# MCUB Inhibits PRKN‐Dependent Mitophagic Degradation of PD‐L1 to Promote Immune Evasion in Bladder Cancer

**DOI:** 10.1002/advs.202514764

**Published:** 2025-11-12

**Authors:** Yuan Huang, Chen Chen, Mingqiang Su, Dongqing Li, Jinge Zhang, Kuangye Long, Wenbin Nie, Shu Wei, Wei Chen, Haiyong Chen, Zhangfeng Zhong, Lina Hou, Wanlong Tan, Fei Li

**Affiliations:** ^1^ Department of Urology Nanfang Hospital Southern Medical University Guangzhou Guangdong 510515 P. R. China; ^2^ Department of Urology Zigong Fourth People's Hospital Zigong Sichuan 643000 P. R. China; ^3^ Department of Urology Baoshan People's Hospital Baoshan Yunnan 678000 P. R. China; ^4^ School of Chinese Medicine LKS Faculty of Medicine The University of Hong Kong R619 3 Sassoon Road, Pokfulam Hong Kong SAR 999077 P. R. China; ^5^ Macao Centre for Research and Development in Chinese Medicine Institute of Chinese Medical Sciences University of Macau Macao SAR 999078 P. R. China; ^6^ Huiqiao Medical Center Nanfang Hospital Southern Medical University Guangzhou Guangdong 510515 P. R. China

**Keywords:** bladder cancer, immune evasion, MCUB, mitophagy, PD‐L1

## Abstract

Muscle‐invasive bladder cancer (MIBC) poses a severe threat to patient survival due to its high invasiveness and metastatic potential. Although immunotherapy has revolutionized treatment strategies for MIBC, immune evasion remains a major obstacle limiting therapeutic efficacy. In this study, the mitochondrial calcium uniporter regulatory subunit (MCUB) is investigated for its role in immune evasion in MIBC. Bulk RNA‐seq, scRNA‐seq, and proteomic analyses revealed a progressive upregulation of MCUB from normal to MIBC tissues, and strong positive correlations are uncovered between MCUB expression and both PD‐L1/PD‐1 signaling and poor outcomes. Spatial transcriptomics and clinical tissue staining confirmed spatial co‐localization of MCUB and PD‐L1. Functional experiments demonstrated that MCUB stabilized PD‐L1 protein by reducing its lysosomal degradation through inhibition of PRKN‐dependent mitophagy. Mechanistically, MCUB suppressed mitochondrial calcium uptake to reduce PRKN activation and physically interacted with the PRKN‐Arg51 residue to inhibit its function. In vivo, MCUB knockdown led to reduced tumor growth, enhanced CD8⁺ T cell infiltration, and improved response to anti‐PD‐1 therapy. This study identified the MCUB‐PRKN‐PD‐L1 axis as a novel driver of immune evasion in MIBC and proposed that targeting the MCUB‐PRKN interaction may serve as a precise therapeutic strategy to overcome immune resistance with minimal toxicity to normal tissues.

## Graphical abstract

In bladder cancer, the overexpression of MCUB not only reduces mitochondrial calcium influx but also facilitates its binding to PRKN at Arg51, both mechanisms contribute to the impairment of PRKN‐mediated mitophagy. Consequently, the degradation of PD‐L1 is suppressed, leading to its stable expression on the tumor cell surface. This subsequently promotes the exhaustion of CD8⁺ T cells and facilitates tumor immune evasion.

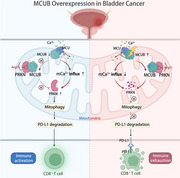

## 1 Introduction

Bladder cancer (BCa) is one of the most prevalent malignant tumors worldwide, with ≈614 000 new cases and 220 000 deaths reported globally in 2022, ranking it ninth among male cancers.^[^ ^1^ ^]^ MIBC, accounting for 20–25% of all BCa cases, is characterized by high aggressiveness and metastatic potential, with a five‐year survival rate of less than 50%, significantly lower than that of non‐muscle‐invasive bladder cancer (NMIBC).^[^ ^2^ ^]^ Standard treatment for MIBC involves surgery, chemotherapy, and radiotherapy. However, high recurrence rates and poor prognosis have driven the search for novel therapeutic strategies.^[^ ^3^ ^]^ Immune checkpoint inhibitors (ICIs), such as pembrolizumab and atezolizumab, have improved outcomes for advanced MIBC patients. For example, pembrolizumab has demonstrated a 21% objective response rate (ORR) in second‐line treatment of advanced urothelial carcinoma, with a subset of patients experiencing prolonged overall survival (OS).^[^ ^4^ ^]^ Nevertheless, the overall response rate to ICIs remains only 20‐30%, largely due to immune evasion mechanisms.^[^ ^5^ ^]^ Therefore, uncovering the molecular mechanisms of immune evasion is essential to optimizing immunotherapy for MIBC.

Immune evasion in MIBC involves multifaceted processes, among which the overexpression of programmed death ligand 1 (PD‐L1) plays a central role. PD‐L1 impairs cytotoxic CD8⁺ T cell function by binding to its receptor PD‐1.^[^ ^6^ ^]^ While PD‐L1 expression is transcriptionally upregulated in inflammatory signaling, its protein stability is also tightly controlled by post‐translational mechanisms. For instance, CMTM6 has been shown to prevent PD‐L1 lysosomal degradation, thereby enhancing its cell surface retention in melanoma.^[^ ^7^ ^]^ GSK3β, on the other hand, modulates PD‐L1 degradation through phosphorylation‐dependent mechanisms in non‐small cell lung cancer and breast cancer.^[^ ^8^ ^]^ These findings highlight the critical role of PD‐L1 post‐translational regulation in immune evasion. However, the full spectrum of regulatory mechanisms remains incompletely understood.

Mitochondria orchestrate essential cellular functions, encompassing energy production, calcium homeostasis, and apoptosis.^[^ ^9^ ^]^ Recent evidence has also linked mitochondrial dynamics and quality control to immune checkpoint regulation. Mitochondrial stress or defective mitophagy has been reported to stabilize PD‐L1 and promote immune evasion.^[^ ^10^ ^]^ This highlights mitochondria as a hub connecting cellular metabolism with immune modulation. Among mitochondrial signals, calcium uptake plays a pivotal role, as it regulates mitophagy and downstream immune pathways. Mitochondrial calcium uptake is primarily mediated by the mitochondrial calcium uniporter (MCU) complex, within which MCUB (also known as CCDC109B) acts as an inhibitory subunit that suppresses calcium influx into the mitochondrial matrix.^[^ ^11^ ^]^ MCUB has been reported to modulate oxidative phosphorylation in cardiomyocytes and influence synaptic transmission in neurons through regulation of mitochondrial calcium levels.^[^ ^12^ ^]^ Elevated expression of MCUB has also been observed in gliomas, where it correlates with enhanced cell proliferation and invasiveness.^[^ ^13^ ^]^ Notably, our analyses of BCa multi‐omics datasets suggested that among the MCU complex members, MCUB uniquely displayed a progressive increase from normal epithelium to NMIBC and MIBC, whereas other components showed no such pattern. This distinctive expression trend, together with its inhibitory role in mitochondrial calcium uptake, made MCUB a particularly compelling candidate for investigation in the context of immune regulation. However, the role of MCUB in BCa, especially in tumor immune modulation, remains undefined. Given the crucial role of mitochondrial calcium signaling in shaping antitumor immunity, it is plausible that MCUB may promote immune evasion by reprogramming mitochondrial dynamics.

Mitophagy, a selective form of autophagy responsible for the clearance of damaged mitochondria,^[^ ^14^, ^15^ ^]^ is governed by the PINK1/PRKN signaling axis and is critically involved in antitumor immune responses.^[^ ^16^ ^]^ It has been reported that mitophagy activates the cGAS‐STING axis and amplifies type I interferon production.^[^ ^17^ ^]^ In addition, PD‐L1 has been shown to undergo degradation via PINK1‐dependent mitophagy, which contributes to improved antitumor immunity.^[^ ^18^ ^]^ Beyond this evidence, recent studies have expanded the link between mitophagy and PD‐L1 regulation. Cui et al. summarized that autophagy, including selective mitophagy, controls PD‐L1 turnover in several tumor contexts.^[^ ^19^ ^]^ Wang et al. reported that autophagic degradation of PD‐L1 enhanced antitumor immunity.^[^ ^20^ ^]^ These findings highlighted the selective mitophagy and mitochondrial quality control as central mechanisms governing PD‐L1 stability and immune escape. Since mitochondrial calcium uptake regulates mitophagy,^[^ ^21^ ^]^ MCUB may suppress mitophagy by limiting calcium influx, leading to stabilization of PD‐L1 and immune evasion. Nevertheless, whether MCUB contributes to immune evasion in MIBC through modulation of mitophagy remains to be elucidated.

Recent advances in multi‐omics technologies have enabled a systematic dissection of tumor heterogeneity and immunological interactions. Single‐cell RNA sequencing (scRNA‐seq) has been applied to delineate cellular compositions and transcriptional dynamics within the tumor microenvironment,^[^ ^22^ ^]^ while bulk RNA sequencing (bulk RNA‐seq) and proteomics provide comprehensive gene and protein expression landscapes.^[^ ^23^ ^]^ Spatial transcriptomics further uncovers the spatial architecture of gene expression within the tumor‐immune niche.^[^ ^24^ ^]^ These tools have offered unprecedented opportunities to elucidate the role of MCUB in immune evasion.

In this study, MCUB was found to drive immune evasion by stabilizing PD‐L1 protein through inhibition of PRKN‐mediated mitophagy. A comprehensive multi‐omics approach was employed, and in vitro and in vivo experiments were conducted to elucidate the underlying mechanism. Mechanistically, MCUB reduced mitochondrial calcium uptake and directly bound to the PRKN‐Arg51 residue, thereby impairing PRKN function and disrupting mitophagy‐dependent degradation of PD‐L1. Knockdown of MCUB enhanced anti‐tumor immunity and improved the efficacy of anti‐PD‐1 therapy. The MCUB‐PRKN‐PD‐L1 axis was thus identified as a critical mediator of immune evasion in MIBC and a potential target for combinational immunotherapy.

## 2 Results

### 2.1 MCUB was Upregulated Along the Epithelial Trajectory During MIBC Progression

**Figure 1:**
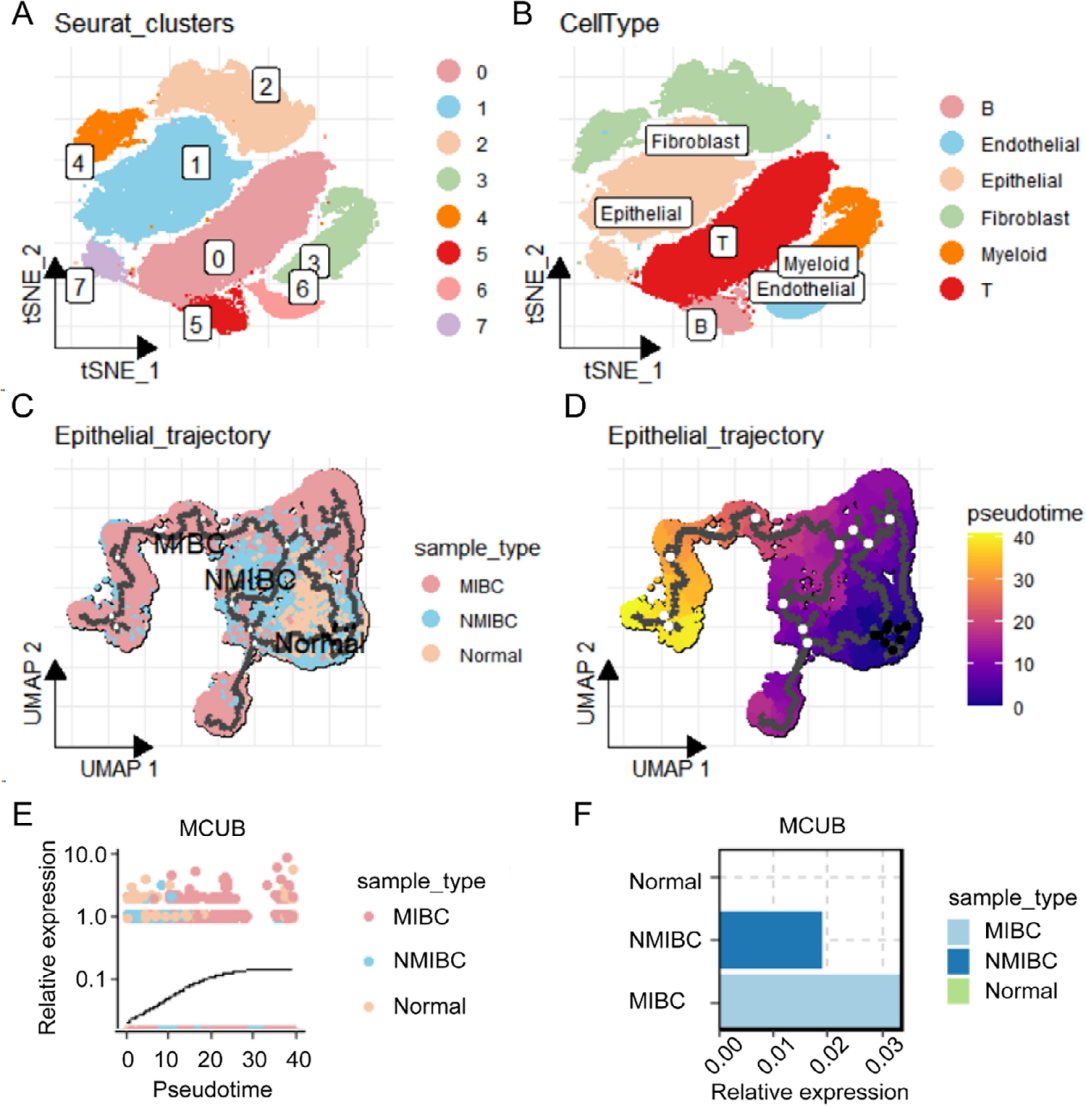
Single‐cell analysis revealed MCUB expression dynamics during MIBC progression. A) 8 clusters were identified from scRNA‐seq data of normal, NMIBC, and MIBC tissues. B) Cell types were annotated using the SingleR algorithm. C) Epithelial cells were extracted and re‐clustered by tissue origin. D) A pseudotime trajectory was constructed using the Monocle3 algorithm, illustrating a continuum from normal to NMIBC to MIBC. E) MCUB expression was visualized along pseudotime in epithelial cells. F) Relative MCUB expression levels were compared among normal, NMIBC, and MIBC‐derived epithelial cells.

scRNA‐seq data from normal, NMIBC, and MIBC tissues were integrated to characterize epithelial dynamics during BCa progression. A total of 8 distinct cell clusters were identified (**Figure** **1**A). Cell types, encompassing epithelial, immune (T cells, B cells, myeloid cells), and stromal (fibroblasts and endothelial cells) compartments, were annotated (Figure 1B).

To further characterize the heterogeneity of epithelial cells, epithelial subsets were isolated and re‐clustered by tissue origin. MIBC‐derived epithelial cells exhibited a distinct distribution pattern, which separated them from NMIBC and normal epithelial cells (Figure 1C). Pseudotime analysis constructed an epithelial trajectory from normal to MIBC tissues. Notably, the trajectory culminated in MIBC‐derived epithelial cells (Figure 1D). Along this epithelial trajectory, MCUB exhibited a gradual and stepwise increase from normal to NMIBC and ultimately to MIBC tissues (Figure 1E,F). These findings indicated that MCUB might play a role in epithelial evolution during BCa progression.

### 2.2 MCUB was Elevated in Advanced BCa and Associated with Poor Prognosis

**Figure 2:**
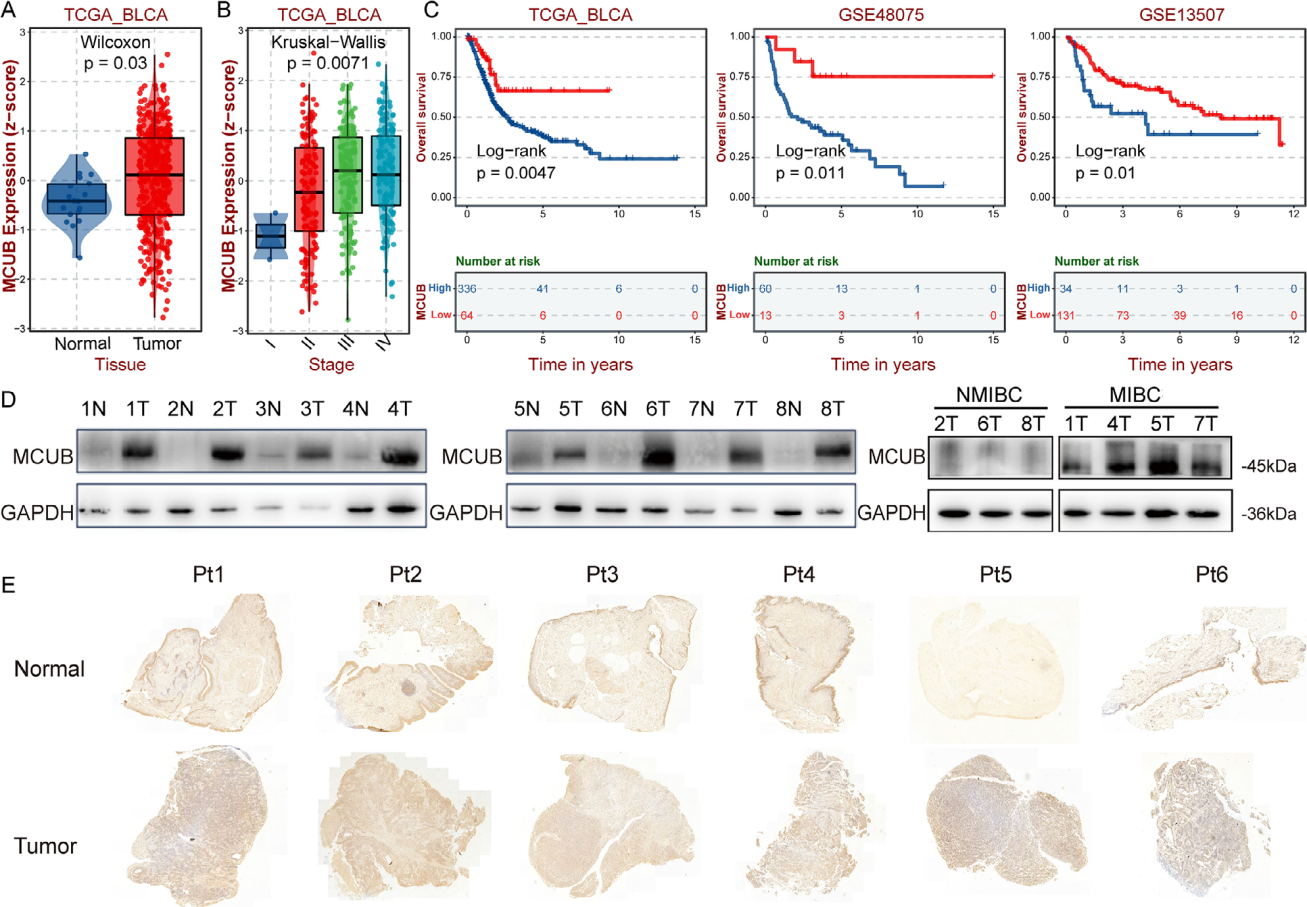
MCUB was upregulated in advanced BCa and associated with poor prognosis. A) The mRNA expression of MCUB in tumor and normal tissues in the TCGA‐BLCA cohort. B) The mRNA expression of MCUB across pathological stages in the TCGA‐BLCA cohort. C) The Kaplan‐Meier overall survival curves in the TCGA, GSE48075, and GSE13507 cohorts. D) Western blotting analysis of MCUB in paired tumor and adjacent normal tissues (left and middle), and in NMIBC versus MIBC tissues (right). E) Representative IHC staining images of MCUB in normal and tumor tissues from 6 clinical BCa patients.

The relationship between MCUB levels and the clinical features of BCa was investigated. The expression of MCUB significantly increased in tumor tissues relative to normal controls in TCGA‐BLCA dataset (*p* = 0.03, **Figure** **2**A). High MCUB expression was positively associated with advanced clinical stage (*p* = 0.0071, Figure 2B). Importantly, Kaplan‐Meier analysis demonstrated that BCa patients with high MCUB expression had poor overall survival in the TCGA, GSE48075, and GSE13507 cohorts (Figure 2C). In line with the above analyses, MCUB protein levels were markedly elevated in MIBC samples compared to NMIBC and normal tissues in BCa proteomic dataset (Figures S1A,B, Supporting Information).

The protein levels of MCUB were validated in 8 clinical samples. MCUB levels were consistently upregulated in tumor tissues relative to paired normal tissues (Figure 2D, left and middle). Notably, the protein levels of MCUB were further elevated in MIBC compared to NMIBC tumors (Figure 2D, right). MCUB was detected in the cytoplasm and nuclei of normal bladder transitional cells and cancerous cells by IHC. Consistently, the staining intensity was stronger in the BCa group than in the corresponding adjacent normal mucosa (Figure 2E). These results indicated that MCUB might play a key role in BCa progression.

In addition, MCUB expression was evaluated across molecular subtypes. In the GSE52219 cohort, a trend toward higher MCUB expression was observed in basal tumors compared with luminal tumors, although the difference was not statistically significant (*p* = 0.67) (Figure S1C, Supporting Information, left). In the TCGA‐BLCA cohort, MCUB expression was significantly higher in non‐papillary than in papillary tumors (*p*<0.01) (Figure S1C, Supporting Information, right). These findings suggested that MCUB might be associated with more aggressive subtypes of BCa.

### 2.3 MCUB was Associated with Immune Signals and PD‐L1 Expression

**Figure 3:**
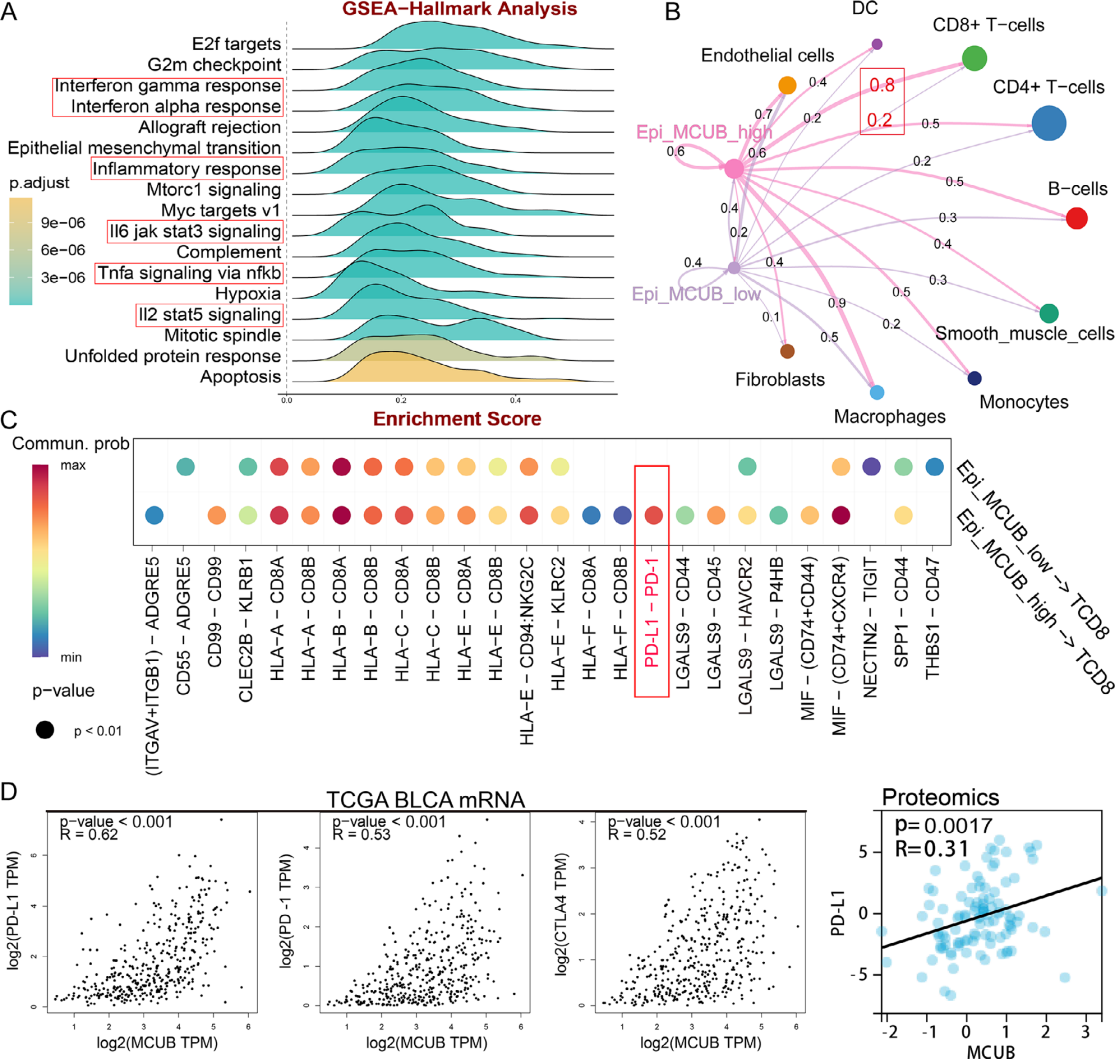
MCUB was associated with immune pathways and checkpoints in BCa. A) GSEA of MCUB in the TCGA‐BLCA dataset. B) Cell‐cell communication between epithelial cells and other compartments in the tumor microenvironment. C) Ligand‐receptor interaction analysis between epithelial cells and CD8⁺ T cells. D) Correlation analyses between MCUB and immune checkpoints (PD‐L1, PD‐1, and CTLA4) were performed in TCGA‐BLCA (left) and proteomic datasets (right).

The mechanisms of MCUB underlying BCa progression were investigated by GSEA. The results revealed enrichments in immune‐related pathways, encompassing IFN, JAK‐STAT, TNF, and NF‐κB signals (**Figure** **3**A). These findings implied an immune‐evasion phenotype associated with MCUB.

To further explore the immunomodulatory role of MCUB, the cell‐cell communication analysis was performed using the CellChat algorithm. MCUB‐high epithelial cells displayed stronger interactions with CD8⁺ T cells than MCUB‐low cells (Figure 3B). Interestingly, PD‐L1/PD‐1 ligand‐receptor pair was detected between MCUB‐high epithelial cells and CD8⁺ T cells (Figure 3C). Correlation analysis revealed strong positive associations between MCUB and key immune checkpoints (PD‐L1, PD‐1, and CTLA4) in TCGA‐BLCA (Figure 3D, left) and BCa proteomic datasets (Figure 3D, right). Notably, PD‐L1 expression showed the strongest correlation with MCUB in TCGA‐BLCA dataset (*r*  0.62, *p* <  0.001). The further analyses revealed that MCUB showed weaker correlations with CD276, CD47, and MHC‐I than PD‐L1 in TCGA‐BLCA dataset (Figure S1D, Supporting Information). These findings demonstrated that MCUB might regulate PD‐L1‐mediated immune evasion in BCa.

In addition, we performed immune deconvolution of bulk transcriptomic data using the ImmuCellAI algorithm (Figure S1E, Supporting Information). Based on the median expression of MCUB, MCUB‐high tumors were enriched in exhausted immune cells. By contrast, naive CD8⁺ T cell subsets were reduced. These results indicated that elevated MCUB was associated with an immunosuppressive tumor microenvironment.

### 2.4 MCUB was Spatially Co‐Localized with PD‐L1 in MIBC Tumors

**Figure 4:**
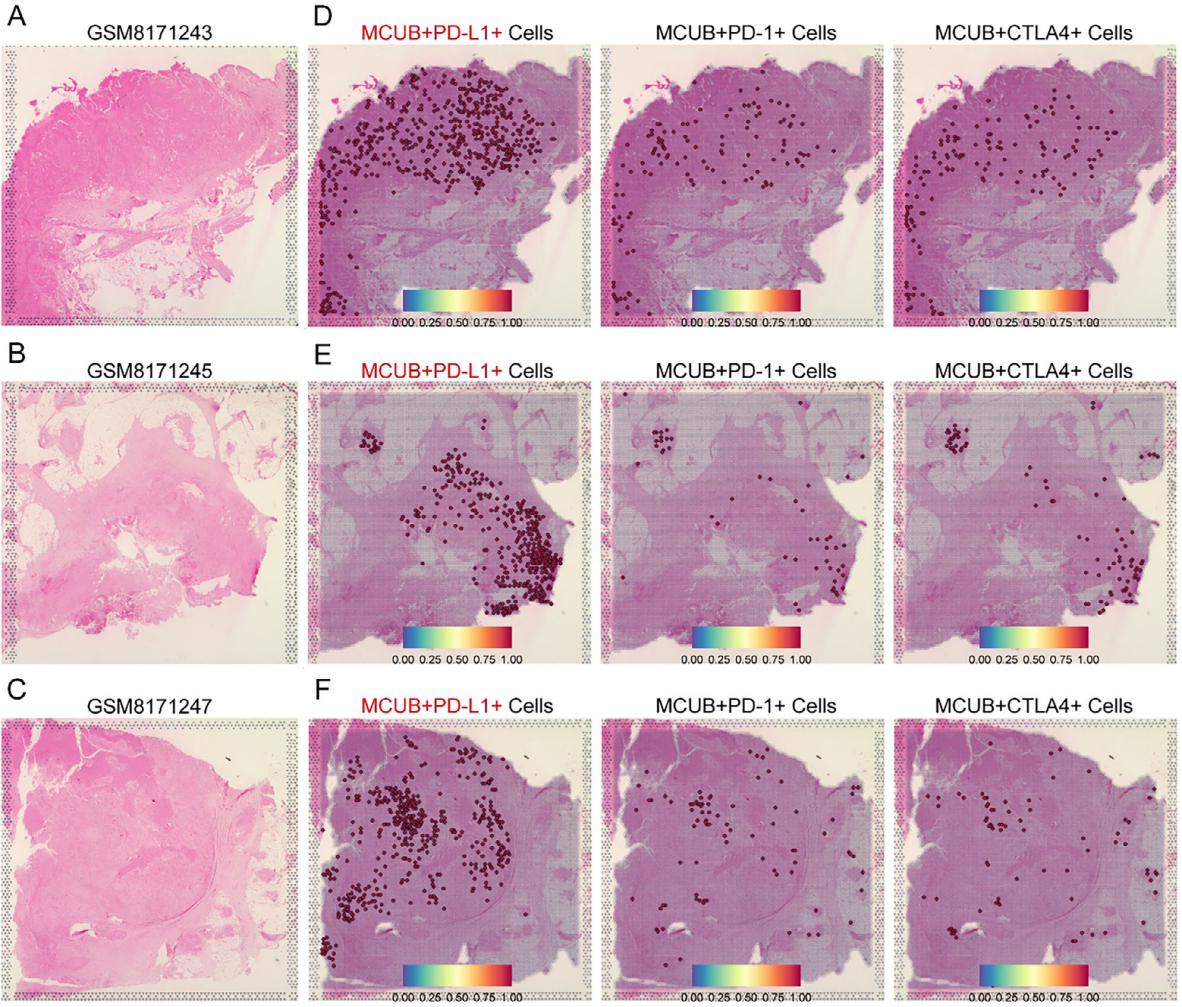
Spatial transcriptomic analysis revealed co‐localization of MCUB and PD‐L1 in MIBC tumors. A–C) H&E staining images of spatial transcriptomic sections from 3 MIBC patients (GSM8171243, GSM8171245, and GSM8171247). D–F) Spatial distributions of MCUB⁺PD‐L1⁺ (left), MCUB⁺PD‐1⁺ (middle), and MCUB⁺CTLA4⁺ (right) double‐positive cells. Spatial co‐expression probabilities were represented by color scales ranging from 0 to 1.

Spatial transcriptomic analysis was performed to assess the distribution of MCUB and immune checkpoints in 3 MIBC tissues (**Figure** **4**A–C). Notably, a pronounced spatial overlap between MCUB and PD‐L1 was observed, with MCUB⁺PD‐L1⁺ double‐positive cells broadly distributed across tumor regions (Figure 4D–F, left). In contrast, MCUB⁺PD‐1⁺ and MCUB⁺CTLA4⁺ double‐positive cells were sparsely detected (Figure 4D–F, middle and right). These findings indicated a preferential spatial association between MCUB and PD‐L1, implicating MCUB in the regulation of PD‐L1 rather than PD‐1 or CTLA4 during immune evasion in MIBC.

**Figure 5:**
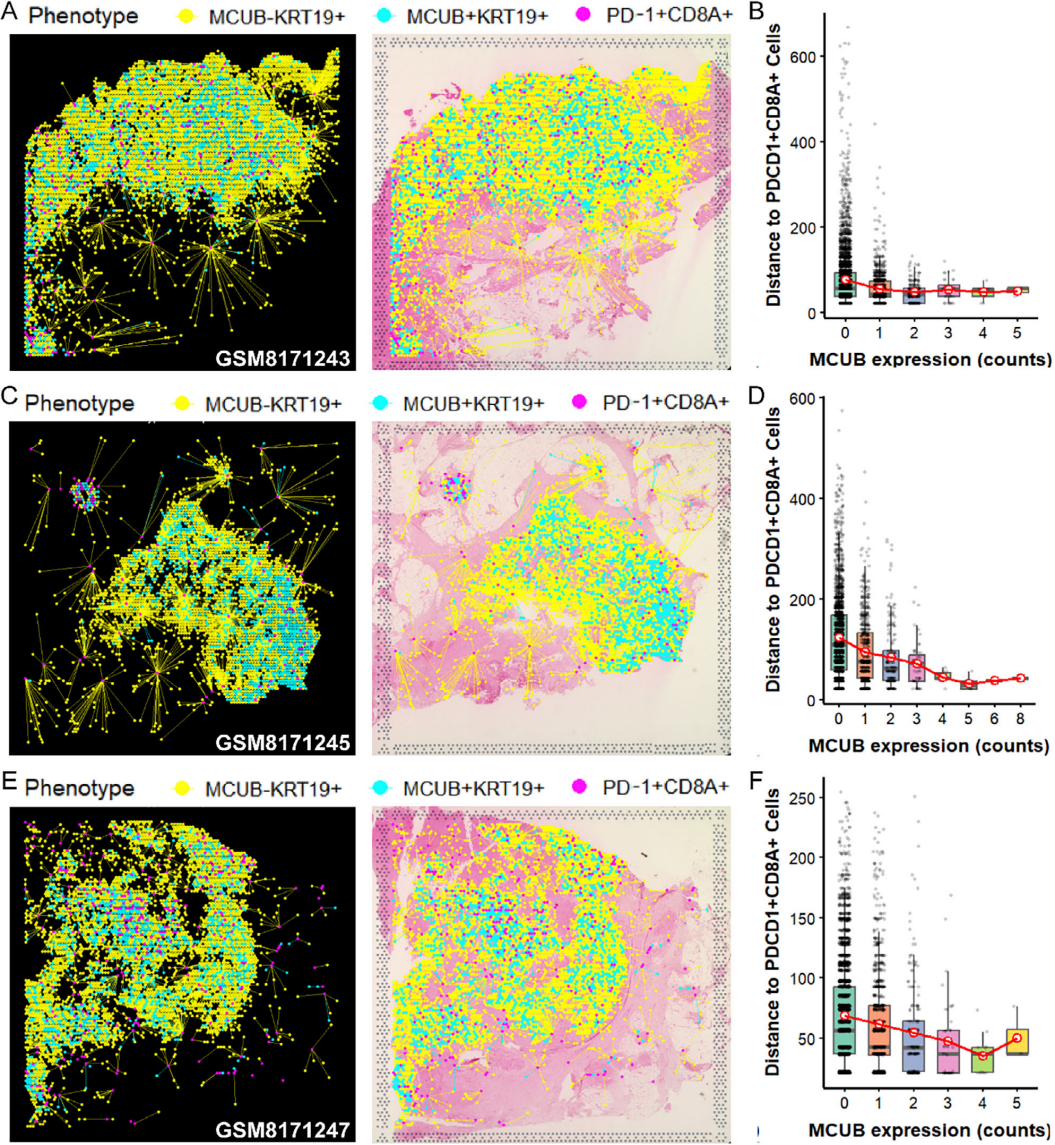
MCUB⁺KRT19⁺ epithelial cells exhibited closer spatial proximity to PD‐1⁺CD8A⁺ T cells in MIBC tissues. A,C,E) Representative spatial transcriptomic plots showing the distribution of MCUB^−^KRT19⁺ epithelial cells (yellow), MCUB⁺KRT19⁺ epithelial cells (cyan), and PD‐1⁺CD8A⁺ T cells (magenta), with lines connecting each epithelial cell to its nearest PD‐1⁺CD8A⁺ T cell. B,D,F) Quantitative analyses of spatial distances between epithelial cells and PD‐1⁺CD8A⁺ T cells, binned by MCUB expression levels in epithelial cells. Boxplots show the median and interquartile ranges, and statistical differences were evaluated by the Mann‐Whitney U test.

Intriguingly, MCUB⁺KRT19⁺ epithelial cells (cyan) were consistently located in closer proximity to PD‐1⁺CD8A⁺ T cells (magenta) than MCUB^−^KRT19⁺ epithelial cells (yellow) across all three tissue sections (**Figure** **5**A,C,E) (Figure S2A–F, Supporting Information). Quantitative distance analysis demonstrated a consistent trend: as MCUB expression levels increased (from low to high counts) in KRT19⁺ epithelial cells, their median spatial distance to PD‐1⁺CD8A⁺ cells decreased (Figure 5B,D,F). The results suggested the potential PD‐L1/PD‐1 crosstalk between MCUB‐expressing epithelial cells and tumor‐infiltrating PD‐1⁺CD8A⁺ T cells.

### 2.5 MCUB Promoted BCa Progression by Enhancing PD‐L1 Expression

**Figure 6:**
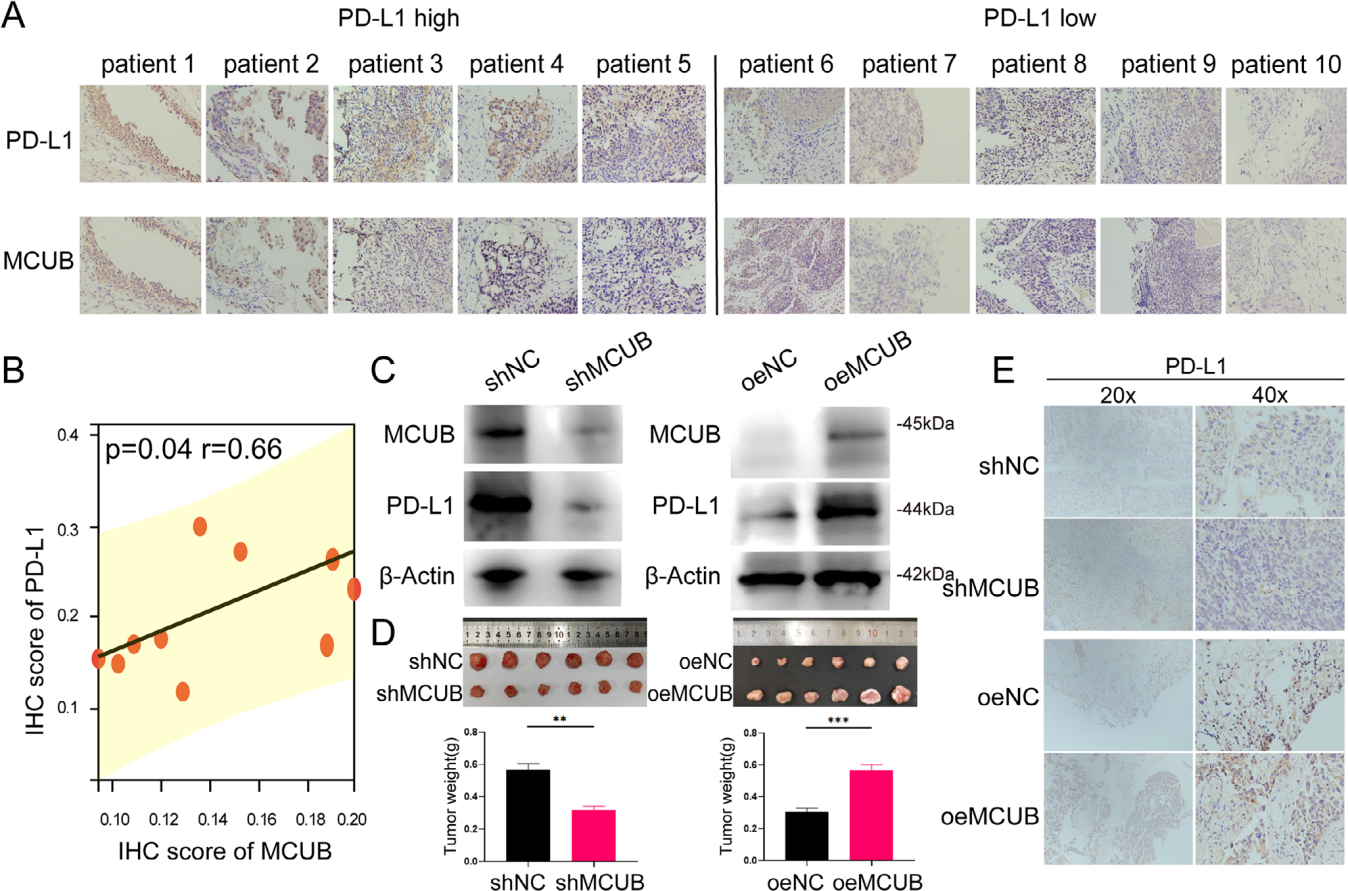
MCUB promoted PD‐L1 expression and tumor progression in BCa. A) IHC staining images of MCUB and PD‐L1 in 10 BCa samples. B) Correlation analysis between MCUB and PD‐L1 IHC scores. C) Western blotting of PD‐L1 expression in MCUB knockdown (shMCUB) and overexpression (oeMCUB) UMUC3 cells. D) Representative tumor images and tumor weights from xenograft models following MCUB knockdown or overexpression in MB49 cells (*N* = 6 per group). E) IHC staining images of PD‐L1 in subcutaneous tumors at 20× and 40× magnifications. Statistical analysis was performed using Student's *t*‐test. ^*^ *p*<0.05, ^**^ *p*<0.01, ^***^ *p*<0.001.

The spatial expression patterns of MCUB and PD‐L1 were validated in our in‐house cohort (*N* = 10) by IHC. Consistent with the previous results of spatial transcriptomic analysis, co‐localization of MCUB and PD‐L1 was observed in epithelial regions (**Figure** **6**A). Notably, quantitative analysis revealed a significant positive correlation between the IHC scores of MCUB and PD‐L1 (*r* =  0.66, *p* =  0.04, Figure 6B).

The regulatory effect of MCUB on PD‐L1 was further evaluated in vitro. Western blotting demonstrated that MCUB knockdown reduced PD‐L1 protein levels, while MCUB overexpression led to a marked increase in PD‐L1 expression both in MB49 (Figure S1F, Supporting Information) and UMUC3 (Figure 6C; Figure S3A, Supporting Information) cells. In syngeneic tumor model, MCUB knockdown significantly decreased tumor burden, while MCUB overexpression promoted tumor growth (Figure 6D). IHC analysis of tumor tissues confirmed that PD‐L1 expression was suppressed in MCUB knockdown tumors and enhanced in MCUB overexpression tumors (Figure 6E). Collectively, these results indicated that MCUB promoted BCa progression by enhancing PD‐L1 expression.

### 2.6 MCUB Stabilized PD‐L1 Protein by Inhibiting Mitophagy‐Dependent Lysosomal Degradation

**Figure 7:**
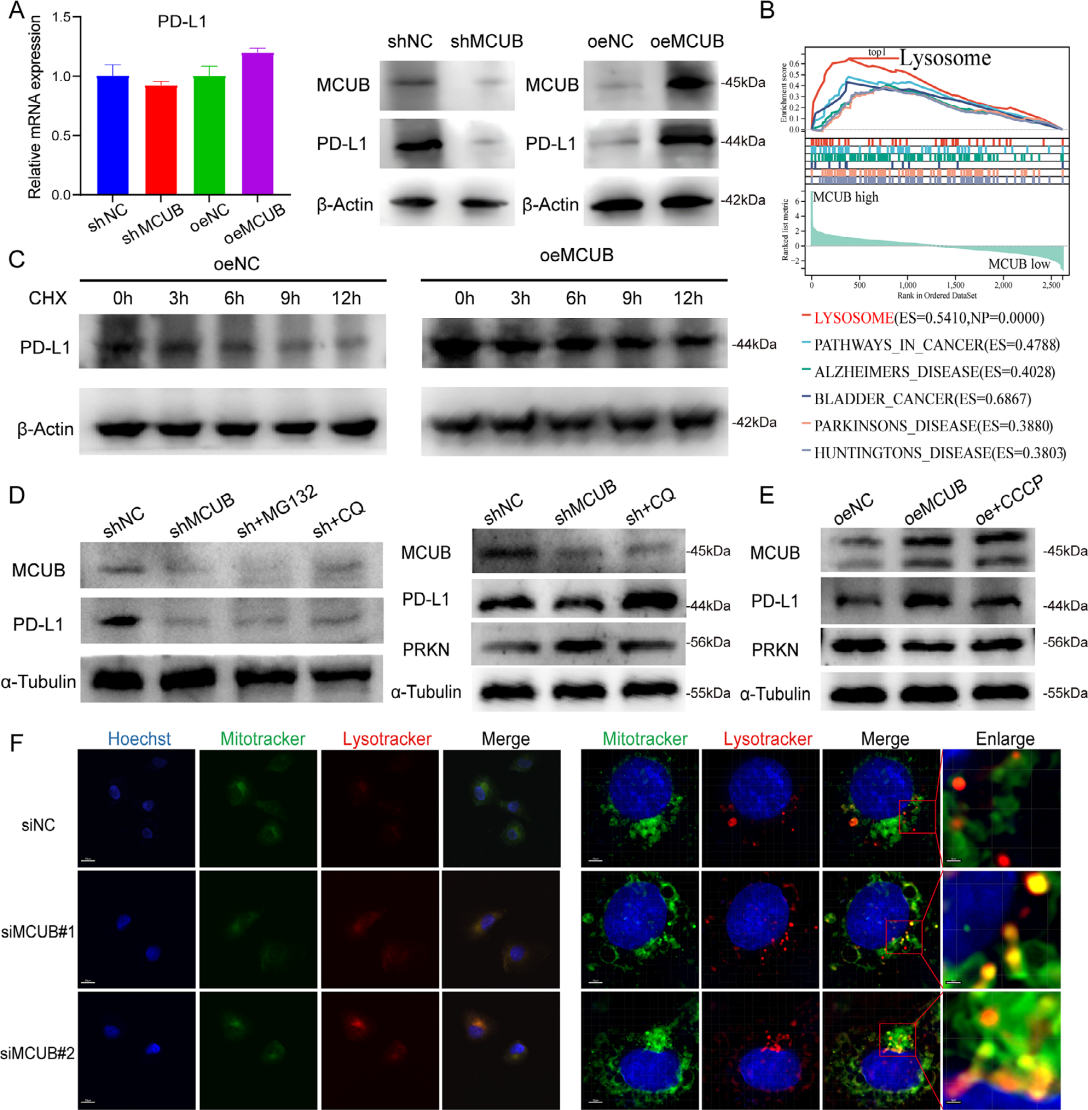
MCUB stabilized PD‐L1 protein by blocking mitophagy‐mediated lysosomal degradation. A) The mRNA and protein levels of PD‐L1 were examined by qRT‐PCR and Western blotting in MCUB knockdown (shMCUB) and overexpression (oeMCUB) BCa cells. B) GSEA was performed based on epithelial scRNA‐seq following virtual knockout of MCUB using the scTenifoldKnk algorithm. C) PD‐L1 protein stability was assessed by Western blotting after CHX treatment at 0, 3, 6, 9, and 12 h in oeMCUB cells. D) Western blotting was performed in shMCUB cells treated with MG132 or CQ to determine the degradation pathway. E) Western blotting was performed in oeMCUB cells treated with or without CCCP. F) Immunofluorescence staining of mitochondria (MitoTracker) and lysosomes (LysoTracker) was performed in siNC and siMCUB‐transfected cells. Scale bar: 20 µm (left), 10 µm (right), 5 µm (right, Enlarge).

To explore the mechanism of MCUB in regulating PD‐L1, we investigated the mRNA and protein levels of PD‐L1 after MCUB knockdown or overexpression. Interestingly, the mRNA levels of PD‐L1 remained unchanged (**Figure** **7**A, left). However, the protein levels of PD‐L1 were increased in the MCUB overexpression group and decreased in the MCUB knockdown group (Figure 7A, right; Figure S3A, Supporting Information). These results supported a post‐transcriptional mode of regulation. Utilizing the scTenifoldKnk algorithm, a virtual knockout of MCUB was conducted in epithelial cells to acquire the downstream genes of MCUB. Crucially, GSEA of downstream genes revealed a significant enrichment in the lysosome pathway (Figure 7B). Based on these results, PD‐L1 was suspected to undergo lysosome‐dependent degradation regulated by MCUB.

The stability of PD‐L1 protein was evaluated by CHX chase assays. In the MCUB overexpression group, PD‐L1 degradation was delayed over time compared to the control group (Figure 7C; Figure S3B, Supporting Information). The MCUB knockdown group was treated with MG132 or CQ to detect the degradation pathway. Surprisingly, CQ treatment restored PD‐L1 protein expression, while MG132 had no detectable effect (Figure 7D; Figure S3C,D, Supporting Information). In addition, CCCP was applied to assess the involvement of mitophagy. Notably, PD‐L1 accumulation was abolished after CCCP‐triggered mitophagy in the MCUB overexpression group (Figure 7E; Figure S3D, Supporting Information, right). Immunofluorescence staining revealed enhanced colocalization between mitochondria and lysosomes in MCUB‐silenced cells (Figure 7F). These results demonstrated that MCUB stabilized PD‐L1 protein by inhibiting mitophagy‐dependent lysosomal degradation of PD‐L1.

### 2.7 MCUB Inhibited PRKN‐Mediated Mitophagy and Stabilized PD‐L1 by Blocking Its Mitophagy‐Dependent Lysosomal Degradation

**Figure 8:**
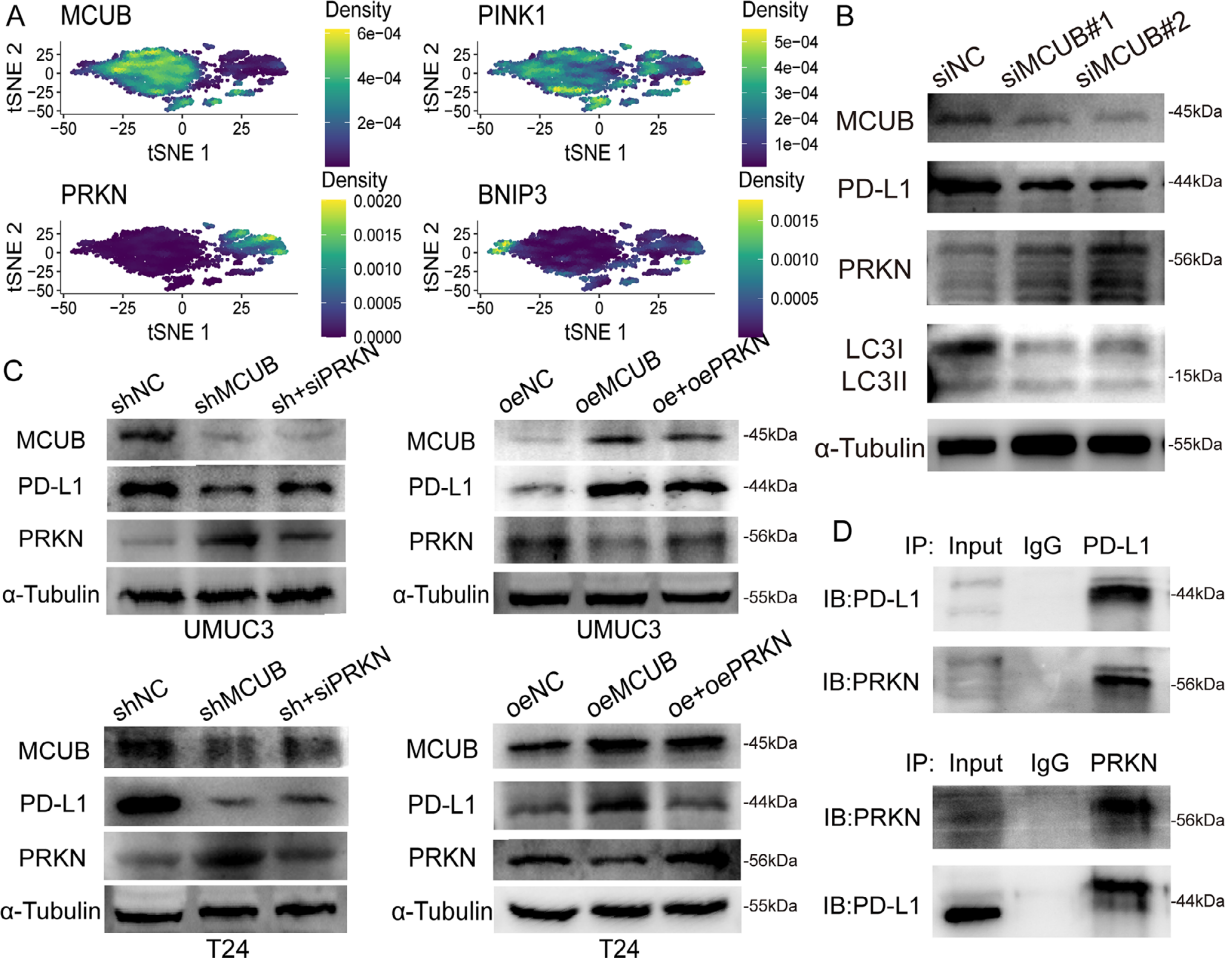
MCUB suppressed mitophagy by inhibiting PRKN and stabilized PD‐L1 protein expression. A) Density maps of MCUB and mitophagy‐related genes were generated from scRNA‐seq‐derived epithelial cells. B) Western blotting was performed in MCUB‐silenced cells to evaluate PRKN expression and LC3II/LC3I ratio. C) Rescue experiments were conducted by co‐silencing or co‐overexpressing PRKN and MCUB to assess the effect on PD‐L1 expression in UMUC3 and T24 cells. D) Co‐IP assays were performed to detect the interaction between PRKN and PD‐L1.

MCUB and mitophagy‐related genes (PINK1, PRKN, and BNIP3) were mapped onto the epithelial cells to reveal the downstream gene through which MCUB inhibited mitophagy. Notably, MCUB was negatively correlated with PRKN (**Figure** **8**A). Western blotting revealed that the expression of PRKN and the ratio of LC3II/LC3I were elevated after MCUB knockdown (Figure 8B). These results implied that MCUB might inhibit PRKN‐mediated mitophagy.

Given that MCUB stabilized PD‐L1 protein by inhibiting mitophagy‐dependent lysosomal degradation, we examined whether PRKN‐mediated mitophagy was essential. Functional rescue assays were performed to assess the role of PRKN in MCUB‐mediated PD‐L1 stabilization. In both UMUC3 and T24 cells, the protein levels of PD‐L1 were restored by PRKN silencing in the MCUB knockdown group, whereas PD‐L1 accumulation was attenuated by PRKN overexpression in the MCUB overexpression group (Figure 8C; Figure S3E,F, Supporting Information). Co‐IP revealed that PRKN might physically interact with PD‐L1 (Figure 8D). Briefly, the above findings demonstrated that MCUB inhibited PRKN‐mediated mitophagy to stabilize PD‐L1 by reducing PD‐L1's mitophagy‐dependent lysosomal degradation.

### 2.8 MCUB Suppressed PRKN Activity by Limiting Mitochondrial Calcium Uptake

**Figure 9:**
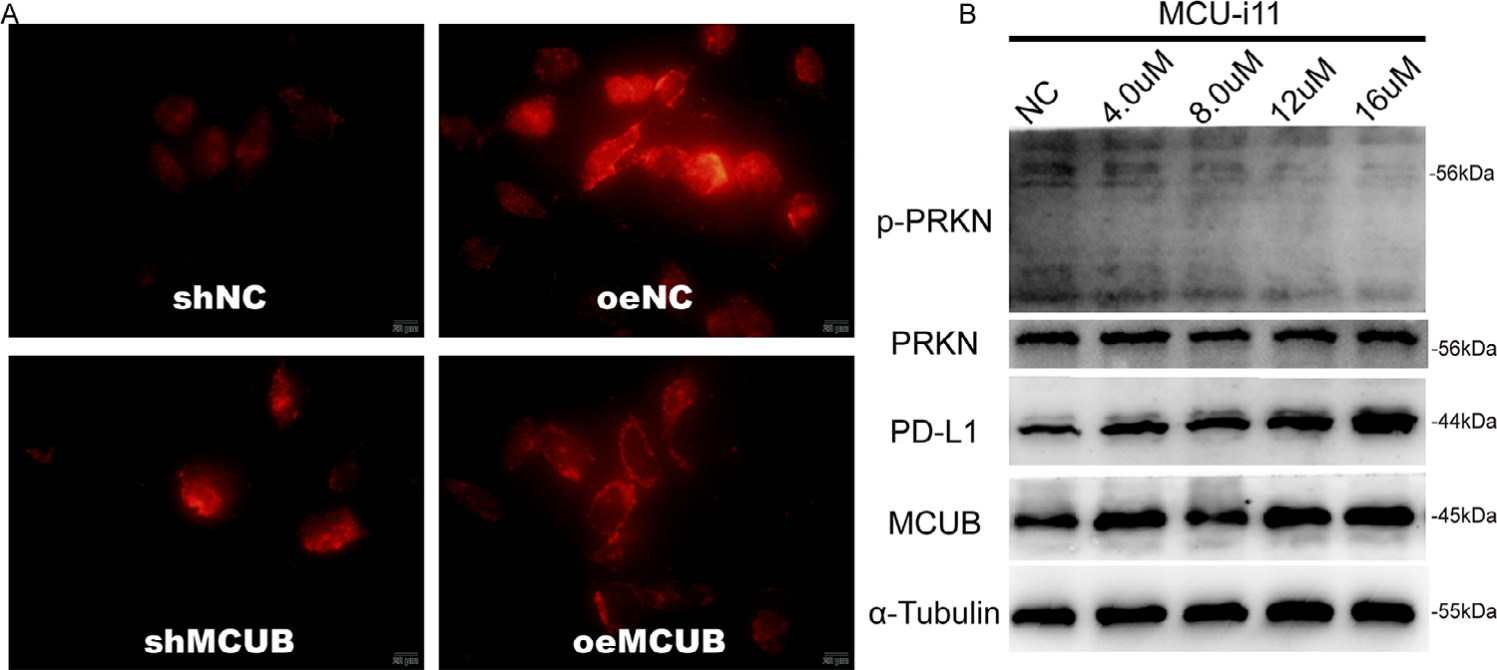
MCUB reduced mitochondrial calcium uptake to inhibit the activation of PRKN. A) Mitochondrial calcium levels were measured in MCUB knockdown or overexpression UMUC3 cells using the calcium fluorescent probe. B) Western blotting was performed to detect the levels of p‐PRKN (Ser65), PRKN, PD‐L1, MCUB, and α‐Tubulin in BCa cells treated with increasing concentrations of MCU‐i11.

As a component of the mitochondrial calcium uniporter complex, MCUB has been reported to suppress mitochondrial calcium uptake.^[^ ^12^ ^]^ To explore whether MCUB inhibited mitochondrial calcium influx in BCa cells, the mitochondrial calcium probe was applied following MCUB knockdown or overexpression (**Figure** **9**A). An increase in mitochondrial calcium fluorescence intensity was observed in the MCUB knockdown group (Figure 9A, left), whereas a decreased fluorescence signal was detected in the MCUB overexpression group (Figure 9A, right). The results demonstrated that MCUB inhibited mitochondrial calcium uptake in BCa cells.

MCU‐i11, a pharmacological inhibitor of the MCU complex, was used to block mitochondrial calcium uptake. This compound does not directly target MCUB and serves to test whether PRKN activation was calcium‐dependent. The protein levels of PRKN and MCUB remained unchanged upon increasing concentrations of MCU‐i11 (Figure 9B). Intriguingly, the level of PRKN phosphorylation at Ser65 (p‐PRKN) progressively decreased, accompanied by a dose‐dependent accumulation of PD‐L1 protein. These results confirmed that MCUB suppressed PRKN activation by reducing mitochondrial calcium influx, thereby stabilizing PD‐L1 protein through inhibiting mitophagy‐dependent degradation.

### 2.9 MCUB Regulated PRKN‐Mediated Mitophagy Through Direct Interaction with the PRKN‐Arg51 Residue

**Figure 10:**
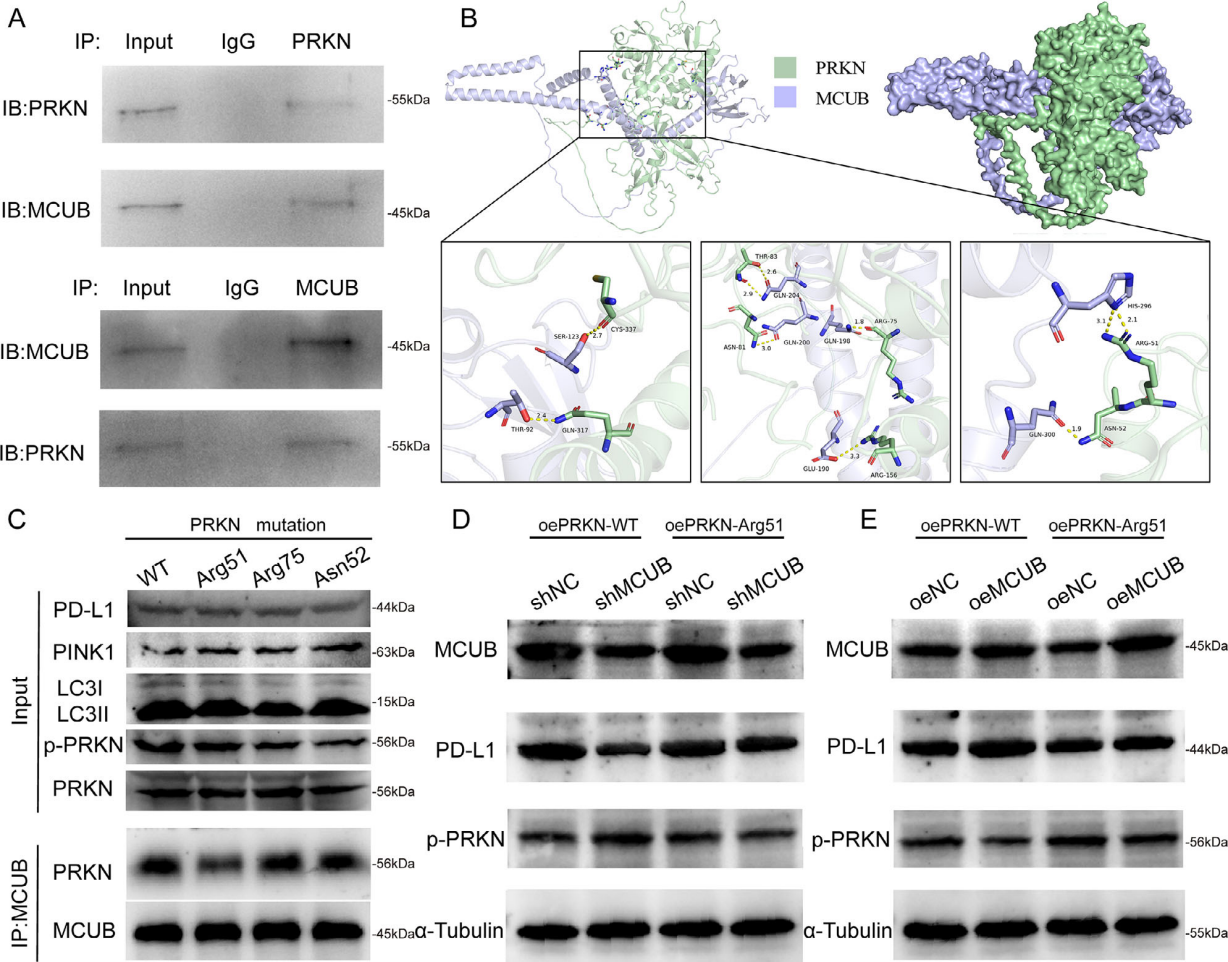
MCUB regulated PD‐L1 expression and mitophagy through interaction with the PRKN‐Arg51 site. A) Co‐IP was performed to detect the physical interaction between MCUB and PRKN. B) Molecular docking analysis was conducted to identify MCUB‐binding residues on PRKN, and Arg51, Asn52, and Arg75 were predicted as candidate sites. C) PRKN wild‐type and site‐directed mutants were analyzed by Co‐IP and Western blotting to assess MCUB binding and downstream expression levels of PINK1, LC3B, and p‐PRKN. D–E) PRKN‐WT and PRKN‐Arg51 constructs were transfected into MCUB‐knockdown D) or MCUB‐overexpressing E) BCa cells, and the levels of PD‐L1 and p‐PRKN were examined by Western blotting.

The Co‐IP assays were used to determine whether MCUB directly modulated PRKN. A physical interaction between MCUB and PRKN was observed (**Figure** **10**A). Notably, 3 candidate residues on PRKN (Arg51, Asn52, and Arg75) were identified as potential MCUB interaction sites by molecular docking analysis (Figure 10B).

The site‐directed mutagenesis of PRKN was performed. The result revealed that the substitution of Arg51 markedly disrupted the interaction of MCUB‐PRKN in the IP group (Figure 10C, bottom), while the levels of PRKN and p‐PRKN remained unaffected in the Input group (Figure 10C, top). Functional assays indicated that mutation of Arg51 attenuated the effect of MCUB knockdown or overexpression on the levels of PD‐L1 and p‐PRKN (Figure 10D,E). These findings demonstrated that MCUB modulated PRKN‐dependent mitophagy and PD‐L1 degradation through direct interaction with the Arg51 residue on PRKN.

### 2.10 MCUB Knockdown Enhanced Antitumor Immunity and Improved PD‐1 Blockade Efficacy

**Figure 11:**
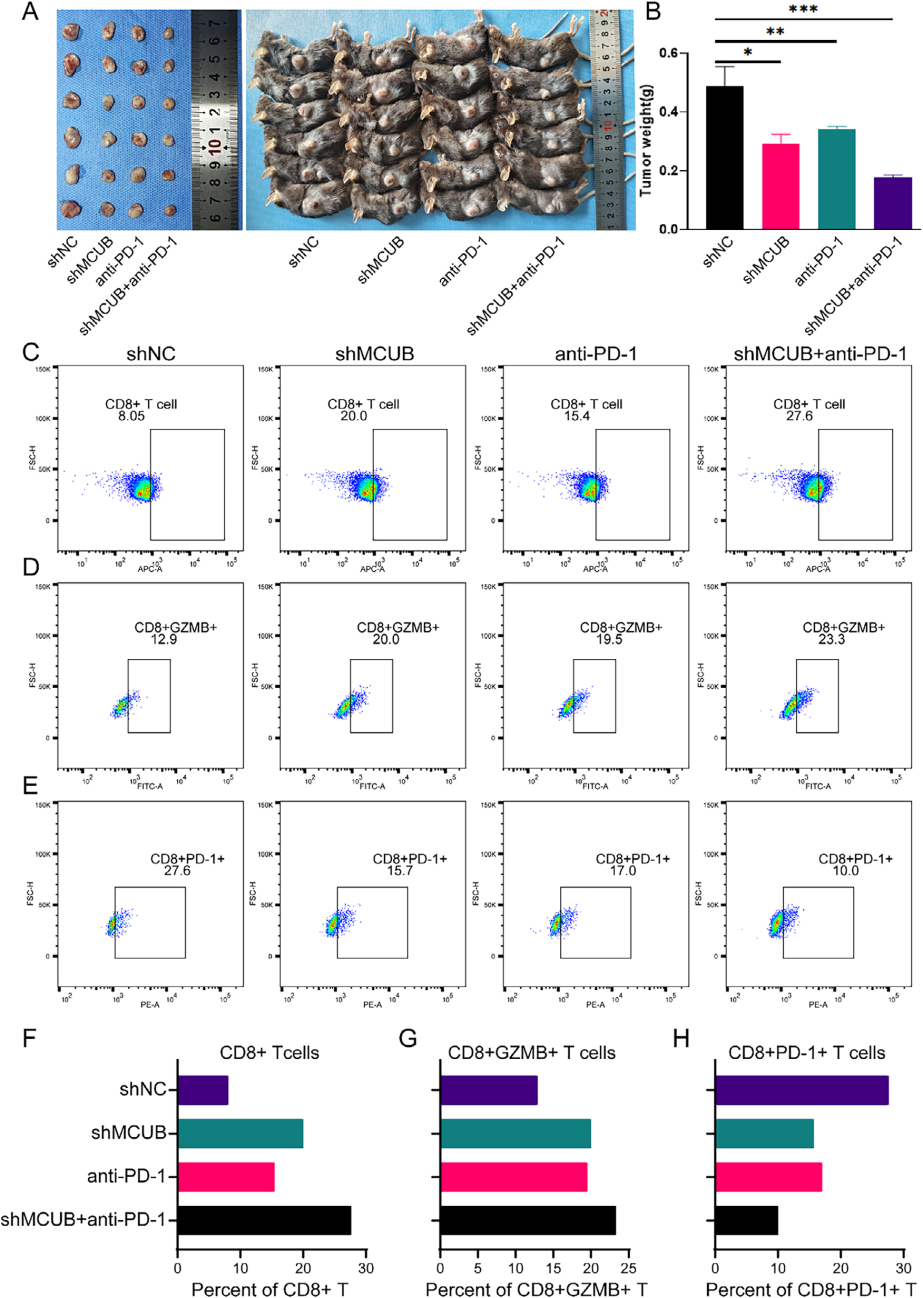
MCUB knockdown enhanced antitumor immunity and improved PD‐1 blockade efficacy. A,B) Representative tumor images A) and tumor weights B) were recorded from four groups: shNC, shMCUB, anti‐PD‐1 antibody, and shMCUB combined with anti‐PD‐1 treatment. C–E) Flow cytometry quantification of the proportions of CD8⁺ T cells C), CD8⁺GZMB⁺ cytotoxic T cells D), and CD8⁺PD‐1⁺ exhausted T cells E) in tumors from each group. F–H) The bar graphs showed the percentages of CD8⁺ T cells F), CD8⁺GZMB⁺ cytotoxic T cells G), and CD8⁺PD‐1⁺ exhausted T cells H) within the total CD8⁺ T cell population. Statistical analysis was performed using Student's *t*‐test. ^*^ *p*<0.05, ^**^ *p*<0.01, ^***^ *p*<0.001.

To assess the impact of MCUB on tumor growth and immune response in vivo, the immunocompetent tumor model was established (**Figure** **11**A). The tumor weight was markedly reduced in both MCUB knockdown and anti‐PD‐1 monotherapy groups and further diminished upon combination treatment (Figure 11B). The results indicated that the knockdown of MCUB inhibited tumor progression and improved PD‐1 blockade efficacy.

The composition of immune cells was analyzed by flow cytometry. The results revealed that the knockdown of MCUB led to the increased infiltration of CD8⁺ T cells (Figure 11C,F), elevated frequencies of cytotoxic CD8⁺GZMB⁺ subsets (Figure 11D,G), and reduced the proportion of exhausted CD8⁺PD‐1⁺ cells (Figure 11E,H). A comparable remodeling of the immune landscape was observed in the anti‐PD‐1 monotherapy group. Notably, the combined treatment with MCUB knockdown and PD‐1 blockade further enhanced the infiltration of cytotoxic CD8⁺ T cells and reduced the exhaustion of T cells. These findings demonstrated that MCUB suppression reprogrammed the immune microenvironment and enhanced the efficacy of immune checkpoint therapy in BCa.

To further investigate the clinical relevance of MCUB in immunotherapy, we analyzed the IMvigor210 cohort of BCa patients treated with anti‐PD‐L1 therapy. The MCUB‐high patients demonstrated longer overall survival than the MCUB‐low patients (Figure S3G, Supporting Information). This trend was consistent with the observation that CD274 (PD‐L1) expression was also positively associated with immunotherapy benefit in the same cohort (Figure S3H, Supporting Information). These findings suggested that although MCUB promoted immune evasion by stabilizing PD‐L1, the MCUB‐high patients were more dependent on PD‐L1 signaling, thereby exhibiting increased sensitivity to PD‐L1 blockade.

## 3 Discussion

Immunotherapy with anti‐PD‐1/PD‐L1 antibodies has revolutionized MIBC treatment. However, low response rates driven by immune evasion have been identified as a critical barrier.^[^ ^25^ ^]^ Novel targets linked to immune evasion are warranted to enhance therapeutic efficacy. As a key regulator of cellular metabolism and immune function, mitochondrial Ca^2^⁺ signaling has been recognized as a pivotal factor in the tumor microenvironment.^[^ ^26^ ^]^ The MCU complex, governing mitochondrial Ca^2^⁺ homeostasis, has been established as a central player in cancer progression and immune modulation.^[^ ^26^, ^27^ ^]^ As an inhibitory component of M8CU complex, MCUB was identified as a potential immune regulator, and its role in MIBC was systematically elucidated, which provided insights into novel immune evasion targets.

Through the multi‐omics integrated analyses, the role of MCUB in the progression and immune evasion was elucidated. Starting with the construction of a single‐cell atlas in BCa, MCUB was identified as a malignancy‐associated gene in MIBC‐related epithelial trajectories. Its progressive upregulation during epithelial differentiation, and significant association with advanced stage and poor prognosis were confirmed by bulk RNA‐seq and proteomic datasets. Besides, spatial transcriptomics and IHC staining further validated the spatial co‐expression and colocalization of MCUB and PD‐L1 within the tumor microenvironment. Moreover, cell‐cell communication analysis revealed an MCUB‐ enhanced PD‐L1/PD‐1 signaling between tumor epithelial cells and CD8^+^ T cells, which established MCUB as a potential driver of immune evasion.

Building upon these findings, our study delineated the molecular mechanism by which MCUB regulates PD‐L1 expression. Functional experiments demonstrated that MCUB did not affect PD‐L1 mRNA levels. Instead, it markedly stabilized PD‐L1 protein by inhibiting its mitophagy‐dependent lysosomal degradation. Mechanically, mitophagy was inhibited by MCUB through two distinct pathways: first, by reducing mitochondrial Ca^2^⁺ uptake, which attenuated PRKN activation, consistent with prior reports where Ca^2^⁺ overload triggered mitochondrial membrane potential disruption and PRKN‐mediated mitophagy^[^ ^21^, ^28^ ^]^; second, through MCUB's direct binding to PRKN at the Arg51 residue, identified as a critical interaction interface via molecular docking and mutagenesis. PRKN mutant with the Arg51→Ala substitution was demonstrated to abolish MCUB‐PRKN binding while preserving PRKN's basal function, which confirmed Arg51 as a pivotal regulatory node. It was hypothesized that MCUB's binding to PRKN at Arg51 disrupts the canonical PRKN activation pathway, specifically PINK1‐mediated phosphorylation at Ser65,^[^ ^29^ ^]^ which is a critical step for PRKN activation. Notably, Arg51 is located in close spatial proximity to Ser65, and previous structural studies have shown that alterations in residues adjacent to Ser65 can sterically influence its phosphorylation status.^[^ ^30^ ^]^ Our findings suggested that MCUB binding to Arg51 may hinder or distort the local conformation surrounding Ser65, thereby interfering with PINK1‐mediated phosphorylation and ultimately suppressing PRKN activation. A similar mechanism has been documented for protein like AMBRA1, where AMBRA1 directly binds PRKN, particularly near its RING domains, including RING0 (where Ser65 and Arg51 reside). This interaction enhances PRKN's recruitment to damaged mitochondria, amplifying PINK1's activation effect. This dual mechanism, uniquely centered on MCUB‐PRKN Arg51 binding, distinguishes MCUB from other regulators like ATAD3A, which interacts with PINK1 and modulates PD‐L1 stability by facilitating its import into mitochondria or promoting its degradation by mitochondrial proteases.^[^ ^18^ ^]^

The translational significance of MCUB targeting was validated in vivo. MCUB knockdown significantly reduced tumor growth and enhanced CD8⁺ T cell infiltration and effector activation. These effects were further augmented by PD‐1 blockade, supporting a synergistic therapeutic effect. Clinical IHC analysis confirmed MCUB and PD‐L1 co‐expression, suggesting its utility as a stratification biomarker. Given MCUB's physiological role in normal cells, directly targeting MCUB may raise safety concerns. Therefore, disrupting the MCUB‐PRKN Arg51 interaction could serve as a more precise and safer therapeutic approach. The development of small‐molecule inhibitors or peptide mimetics targeting this interface may benefit MIBC patients with immune resistance.

Nevertheless, several limitations remain. The sample size for scRNA‐seq and spatial transcriptomics was limited. The in vivo model relied solely on MB49 cells, which may not fully capture the heterogeneity of MIBC. In addition, the role of MCUB in other malignancies remains unexplored, limiting the generalizability of the findings. Beyond the PRKN‐dependent mitophagy pathway, it is also conceivable that MCUB may influence tumor immunity through additional mechanisms, such as mitochondrial ROS generation, metabolic reprogramming, or the cGAS‐STING pathway. Although these possibilities were not addressed in the present study, they represent important avenues for future research. Future studies should expand the sample size and model diversity, validate the prognostic value of MCUB in the local cohort, and explore MCUB‐targeted therapy beyond BCa. Rational design of MCUB‐PRKN inhibitors, particularly Arg51‐specific compounds, may provide a foundation for precision immunotherapy.

## 4 Conclusion 

This study defined the MCUB‐PRKN‐PD‐L1 axis as a critical pathway contributing to immune evasion in MIBC. MCUB was shown to inhibit PRKN‐mediated mitophagy through reduced mitochondrial Ca^2^⁺ uptake and Arg51‐dependent binding, resulting in stabilized PD‐L1 expression. Knockdown of MCUB enhanced antitumor immunity and increased responsiveness to anti‐PD‐1 therapy. Targeting MCUB, especially its interaction with PRKN at Arg51, may overcome immunotherapy resistance while minimizing off‐target toxicity.

## 5 Experimental Section

### Data Sources and Preprocessing

Multi‐omics data from public databases and clinical samples were integrated in this study. scRNA‐seq data were obtained from GEO database (GSE129845, GSE130001, GSE135337, GSE159929, GSE192575, GSE211388, GSE225190), covering normal bladder, NMIBC, and MIBC tissues, as detailed in Supplementary Material 1. Spatial transcriptomics data were sourced from GSE238145, including 12 primary MIBC samples. Bulk transcriptomics data (TPM format) were retrieved from the TCGA‐BLCA database via the GDC portal (https://portal.gdc.cancer.gov/). BCa proteomics data were obtained from Xu et al.,^[^ ^23^ ^]^ comprising 192 normal and tumor samples. Pan‐cancer proteomics data were acquired from the CPTAC database (https://pdc.cancer.gov/pdc/cptac‐pancancer).

### Single‐Cell RNA Sequencing Analysis

scRNA‐seq data were processed using the Seurat package.^[^ ^31^ ^]^ Quality control removed cells with <200 or >7500 genes, >20% mitochondrial gene content, or <1000 UMI counts. Data were normalized using SCTransform, and batch effects were corrected with the Harmony algorithm.^[^ ^32^ ^]^ Cell types were annotated and refined using canonical marker genes, including CD3E for T cells, MS4A1 for B cells, KRT19 for epithelial cells, LYZ for myeloid cells, PECAM1 for endothelial cells, and ACTA2 for fibroblasts. The Annotation results were cross‐validated by the SingleR algorithm^[^ ^33^ ^]^ to ensure robustness and minimize misclassification of stromal, immune, and tumor compartments. Epithelial cell differentiation trajectories (normal→NMIBC→MIBC) were constructed using the Monocle3 package.^[^ ^34^ ^]^ Tumor‐immune cell interactions were analyzed with CellChat.^[^ ^35^ ^]^ Virtual MCUB knockout analysis was conducted using the scTenifoldKnk algorithm.^[^ ^36^ ^]^

### Spatial Transcriptomics, Bulk Transcriptomics, and Proteomics Analysis

Spatial transcriptomics data were preprocessed and visualized using Seurat, assessing spatial co‐localization of MCUB with PD‐L1/PD‐1 and other immune checkpoints. Epithelial cells were classified as MCUB⁺ if their normalized expression value of MCUB was >0, and as MCUB^−^ if the value was ≤0, a uniform thresholding strategy was consistently applied across all samples. For each pair of genes, the co‐expression probability was defined as positive when both genes exhibited normalized expression >0 within the same spot. PD‐1⁺ CD8A⁺ T cells were defined as cells with normalized expression of both PD‐1 (PDCD1) and CD8A >0. Euclidean distances were calculated between the centroids of MCUB⁺KRT19⁺ epithelial cells and the nearest PD‐1⁺CD8A⁺ T cells. Distances were quantified using the “nn2” function from the RANN package and aggregated across all spots to generate distribution plots.

Bulk transcriptomics data were analyzed via the GEPIA2 platform^[^ ^37^ ^]^ (http://gepia2.cancer‐pku.cn/), including Kaplan‐Meier survival analysis for MCUB, correlation analysis of MCUB with PD‐L1/PD‐1/CTLA4, and differential expression analysis of MCUB in normal/tumor tissues and across tumor stages. GSEA pathway enrichment analysis was performed using the BEST tool^[^ ^38^ ^]^ based on KEGG and HALLMARK databases. MCUB protein expression was extracted from BCa proteomics^[^ ^23^ ^]^ data (*N* = 192) and compared across normal, NMIBC, and MIBC tissues.

The protein expression of MCUB and PD‐L1 from pan‐cancer proteomics data was analyzed for Spearman correlation. The pan‐cancer proteomic profiles were downloaded from the National Cancer Institute's Clinical Proteomic Tumor Analysis Consortium (CPTAC) Pan‐Cancer Proteome release (https://pdc.cancer.gov/pdc/cptac‐pancancer). The proteomic profiles (*N* = 1119), which encompassed 11 tumor types‐BRCA, ccRCC, COAD, GBM, HGSC, HNSCC, LSCC, LUAD, PDAC, UCEC, and MB, were harmonized and uniformly processed by the Broad Institute with Spectrum Mill.^[^ ^39^ ^]^

### Cell Culture and Transfection

Human BCa cell lines T24 (RRID: CVCL_0554) and UMUC3 (RRID: CVCL_1783) were sourced from ATCC (Manassas, VA, USA) with catalogue numbers HTB‐4 and CRL‐1749, respectively. The murine BCa cell line MB49 (RRID: CVCL_7076) was obtained from Procell Life Science & Technology Co., Ltd (Wuhan, China) with catalogue number CL‐0733. All cell lines were confirmed to be mycoplasma‐free using a MycoAlert PCR kit (Lonza).

Cells were cultured in DMEM with 10% fetal bovine serum (FBS, Gibco) at 37 °C with 5% CO_2_. Stable MCUB knockdown cell lines were generated via lentiviral shRNA targeting the MCUB 3′UTR (sequence: 5′‐GCTAGCTACGATCGTGAAT‐3′, GeneChem), followed by puromycin selection (2 µg/mL, Sigma‐Aldrich) for 2 weeks. Stable MCUB overexpression was achieved using the pLVX‐MCUB lentiviral vector (Addgene #123456) with similar selection. PRKN overexpression and knockdown were performed using the pLVX‐PRKN plasmid (Addgene #65432) and PRKN‐targeted siRNA (sequence: 5′‐CGAUCGUAACGUUGAUAUU‐3′, GenePharma), respectively. Transient MCUB knockdown used MCUB‐targeted siRNA (sequence: 5′‐GCUAGCUACGAUCGUGAAU‐3′, GenePharma). Transfections were performed using Lipofectamine 3000 (Thermo Fisher).

### Pharmacological Inhibition of Mitochondrial Calcium Uptake

MCU‐i11 (MedChemExpress, catalog #HY‐W194810) is a pharmacological inhibitor of the MCU complex that blocks mitochondrial calcium uptake, which does not alter MCUB protein levels. Cells were treated with the MCU complex inhibitor MCU‐i11 at a final concentration of 10 µm for 6 h to block mitochondrial calcium uptake. MCU‐i11 was dissolved in DMSO (vehicle control). After treatment, cells were subjected to mitochondrial calcium detection and mitophagy assays.

### PD‐L1 Expression and Stability Analysis

PD‐L1 mRNA expression was quantified by qRT‐PCR using SYBR Green (Thermo Fisher) with primers:

PD‐L1 F: 5′‐TGGCATTTGCTGAACGCAT‐3′,

PD‐L1R: 5′‐TGCAGCCAGGTCTAATTGTT‐3′;

GAPDH F: 5′‐GGAGCGAGATCCCTCCAAAAT‐3′,

GAPDH R: 5′‐GGCTGTTGTCATACTTCTCATGG‐3′.

Reaction conditions: 95 °C for 10 min, 40 cycles (95 °C for 15 s, 60 °C for 1 min). Relative expression was calculated using the 2^‐ΔΔCt method. PD‐L1 protein stability was assessed via cycloheximide (CHX, 50 µg/mL, Sigma‐Aldrich) chase assays (0, 3, 6, 9, 12 h), with levels detected by Western blotting.

### Proteasome and Lysosome Inhibition Experiments

Cells were treated with MG132 (10 µm, Selleckchem) or chloroquine (CQ, 20 µm, Sigma–Aldrich) for 6 h. Lysates were collected, and PD‐L1 levels were analyzed by Western blotting.

### Mitophagy Detection

LC3B‐II/I conversion and PRKN levels (Abcam ab109012, 1:1000) were detected by Western blotting. Cells were co‐stained with Lysotracker Red (100 nm, Thermo Fisher) and Mitotracker Green (100 nm, Thermo Fisher) at 37 °C for 30 min, and mitochondrial‐lysosomal co‐localization was assessed via confocal microscopy to evaluate mitophagosome formation.

### Mitochondrial Calcium Detection

Mitochondrial calcium levels were measured using the Rhod‐2 AM fluorescent probe (2 µm, Thermo Fisher). Cells were loaded with the probe (3 °C, 30 min), washed thrice with PBS, and fluorescence intensity (excitation/emission: 552/581 nm) was detected via fluorescence microscopy.

### Co‐Immunoprecipitation Experiments

Interactions between MCUB and PRKN, and PRKN and PD‐L1, were assessed by co‐immunoprecipitation (Co‐IP). Cell lysates (RIPA buffer, Thermo Fisher) were incubated overnight at 4 °C with anti‐MCUB (Abcam ab204259, 1:50), anti‐PRKN (Cell Signaling #4211, 1:50), or anti‐PD‐L1 (Abcam ab213524, 1:50) antibodies. Protein A/G magnetic beads (Thermo Fisher) were added and incubated at 4 °C for 2 h. Beads were washed four times, and immune complexes were analyzed by Western blotting.

### Molecular Docking

MCUB (Uniprot ID: Q9NWR8) and PRKN (Uniprot ID: O60260) structures were retrieved from the AlphaFold Protein Structure Database (https://alphafold.ebi.ac.uk/). Molecular docking was performed using AutoDock Vina v1.2.0, focusing on the MCUB‐PRKN interaction interface, identifying key residues (Arg51, Asn52, Arg75). Binding free energy and interaction patterns were visualized using PyMOL.

### Site‐Directed Mutagenesis

PRKN mutants (R51A, R75E, N52Q) were generated on the pLVX‐PRKN plasmid using the Q5 Site‐Directed Mutagenesis Kit (NEB) with primers from Sangon Biotech, verified by Sanger sequencing. Mutant plasmids were transfected using Lipofectamine 3000, with efficiency confirmed by qRT‐PCR and Western blotting. Effects on PD‐L1 stability and PRKN activity were assessed by Western blotting for PD‐L1 and phosphorylated PRKN (p‐PRKN, Abcam ab92505, 1:1000).

### Flow Cytometry Analysis

Murine tumor tissues were digested with 0.1% collagenase IV (37 °C, 30 min) to prepare single‐cell suspensions. Cells were stained with anti‐CD8 (BioLegend #100712, 1:100), anti‐GZMB (BioLegend #515403, 1:100), and anti‐PD‐1 (BioLegend #135205, 1:100) antibodies at 4 °C in the dark for 30 min. Data were acquired using a BD FACSCanto II and analyzed with FlowJo v10.8.1.

### In Vivo Tumor Model

MB49 cells (5 × 10⁵ in 100 µL PBS) with stable MCUB knockdown or overexpression were subcutaneously injected into C57BL/6 female mice (6‐8 weeks, n = 6/group, Jackson Laboratory). Anti‐PD‐1 antibody (BioXCell, clone RMP1‐14, 10 mg kg^−1^) or isotype control IgG (BioXCell, clone 2A3) was administered intraperitoneally weekly for 4 weeks. Tumor volume was measured every 3 days (volume = 0.5 × length × width^2^). Tumors were harvested for immunohistochemical (IHC) analysis of PD‐L1 expression (anti‐PD‐L1, Abcam ab213524, 1:200). All animal experiments were approved by the Medical Ethics Committee of Nanfang Hospital, Southern Medical University (IACUC‐LAC‐20240920‐35).

### Clinical Sample Validation

Formalin‐fixed paraffin‐embedded (FFPE) samples from 10 BCa patients (Nanfang Hospital, 2019–2023) were analyzed by IHC for MCUB (anti‐MCUB, Abcam ab204259, 1:200) and PD‐L1 (anti‐PD‐L1, Abcam ab213524, 1:200) expression. Tissue sections (4 µm) were deparaffinized, rehydrated, and subjected to antigen retrieval in citrate buffer (pH 6.0) at 95 °C for 15 min. Primary antibodies were incubated overnight at 4 °C, followed by DAB staining. PD‐L1 expression was evaluated by 3 independent pathologists using the Combined Positive Score (CPS), calculated as the number of PD‐L1‐positive tumor cells, lymphocytes, and macrophages divided by the total number of viable tumor cells and multiplied by 100. A CPS ≥ 10 was defined as high PD‐L1 expression, while CPS < 10 was defined as low expression. Staining intensity was quantified using ImageJ. MCUB and PD‐L1 expression correlation was assessed by Pearson analysis (*p*<0.05). MCUB expression in 8 patient samples was analyzed by Western blotting, and in 6 samples by IHC, comparing normal and tumor tissues. This project was approved by the Medical Ethics Committee of Nanfang Hospital, Southern Medical University (NFEC‐2025‐072). All patients provided written informed consent prior to sample collection.

### Western Blotting

Cell or tissue lysates (RIPA buffer with 1% protease inhibitor, Thermo Fisher) were separated by 10% SDS‐PAGE and transferred to PVDF membranes (Millipore). Membranes were blocked with 5% non‐fat milk in TBST for 1 h and incubated overnight at 4 °C with primary antibodies: anti‐MCUB (Abcam ab204259, 1:1000), anti‐PD‐L1 (Abcam ab213524, 1:1000), anti‐PRKN (Cell Signaling #4211, 1:1000), anti‐p‐PRKN (Abcam ab92505, 1:1000), anti‐LC3B (Cell Signaling #3868, 1:1000), anti‐PINK1 (Abcam ab23707, 1:1000), anti‐β‐Actin (Cell Signaling #4970, 1:2000), anti‐α‐Tubulin (Abcam ab7291, 1:2000). After TBST washing, membranes were incubated with HRP‐conjugated secondary antibody (Cell Signaling #7074, 1:5000) for 1 h at room temperature, followed by ECL detection (Thermo Fisher).

### Statistical Analysis

Data were analyzed using GraphPad Prism v9.0 (GraphPad Software, San Diego, CA, USA) and R v4.2.2 (R Foundation for Statistical Computing, Vienna, Austria), with relevant packages including Seurat, Monocle3, CellChat, and DESeq2. For high‐throughput data, normalization and batch correction were conducted using the SCTransform and Harmony algorithms implemented in Seurat. Outliers were identified by evaluating the distribution of normalized expression values and mitochondrial gene percentages and excluded before downstream analysis. For spatial transcriptomic data, expression matrices were log‐normalized and scaled prior to visualization.

Continuous variables are presented as mean ± standard deviation (SD) for normally distributed data or as median (interquartile range) for non‐normally distributed data. Categorical variables are expressed as counts (n) and percentages (%). Each experiment was independently repeated at least three times, unless otherwise indicated. The sample size (n) for each statistical analysis or experiment is specified in the corresponding figure legend.

Comparisons between two groups were assessed using two‐tailed Student's t‐tests, while comparisons among multiple groups were performed by one‐way ANOVA followed by Tukey's post hoc test. Non‐parametric data were analyzed using the Mann–Whitney U test or Kruskal–Wallis test as appropriate. For spatial distance analyses, non‐parametric Mann–Whitney U tests were applied. Survival analyses were conducted by the Kaplan–Meier method with log‐rank testing. Correlations between continuous variables were examined using Pearson's or Spearman's correlation coefficients, as specified in figure legends.

For high‐dimensional transcriptomic or proteomic analyses, P‐values were adjusted for multiple testing using the Benjamini–Hochberg false discovery rate (FDR) method. All statistical tests were two‐sided, and a *p*<0.05 was considered statistically significant, with significance levels indicated as follows: ^*^ *p*<0.05, ^**^ *p*<0.01, ^***^ *p*<0.001, ^****^ *p*<0.0001, ns: not significant.

## Conflict of Interest

The authors declare no conflict of interest.

## Declaration of Generative AI and AI‐Assisted Technologies in the Writing Process

During the preparation of this work, the author(s) used ChatGPT 3.5 in order to improve language and readability. After using this tool/service, the author(s) reviewed and edited the content as needed and take(s) full responsibility for the content of the publication.

## Author Contributions

Y.H., C.C., and M.S. contributed equally to this work. Y.H. contributed to writing—original draft, writing—review and editing, validation, methodology, data curation, formal analysis, and conceptualization. C.C. was responsible for software development, investigation, visualization, and methodology. M.S. contributed to writing—original draft, writing—review and editing, validation, and methodology. D.L. and J.Zh. both contributed to validation, software, and data curation. K.L. participated in the investigation and methodology. W.N. and S.W. contributed to software, investigation, visualization, and methodology. W.C. was involved in investigation, visualization, and methodology. H.C. contributed to software, investigation, and visualization. Z.Z. participated in software and investigation. L.H. contributed to visualization, software, formal analysis, and resources. W.T. provided supervision, validation, and funding acquisition. Finally, F.L. contributed to resources, project administration, methodology, investigation, formal analysis, data curation, conceptualization, and funding acquisition.

## Supporting information



Supporting Information

Supporting Information

## Data Availability

The data that support the findings of this study are available from the corresponding author upon reasonable request.
