# Supplementary material for: MCUB Inhibits PRKN‐Dependent Mitophagic Degradation of PD‐L1 to Promote Immune Evasion in Bladder Cancer
Source: Adv Sci (Weinh). 2025 Nov 12;13(5):e14764. doi: 10.1002/advs.202514764 (PMC12849890; doi:10.1002/advs.202514764)
Supplement: Supplementary file 1 — Supporting Information [file ADVS-13-e14764-s001.docx]

**Supplementary figures**

**
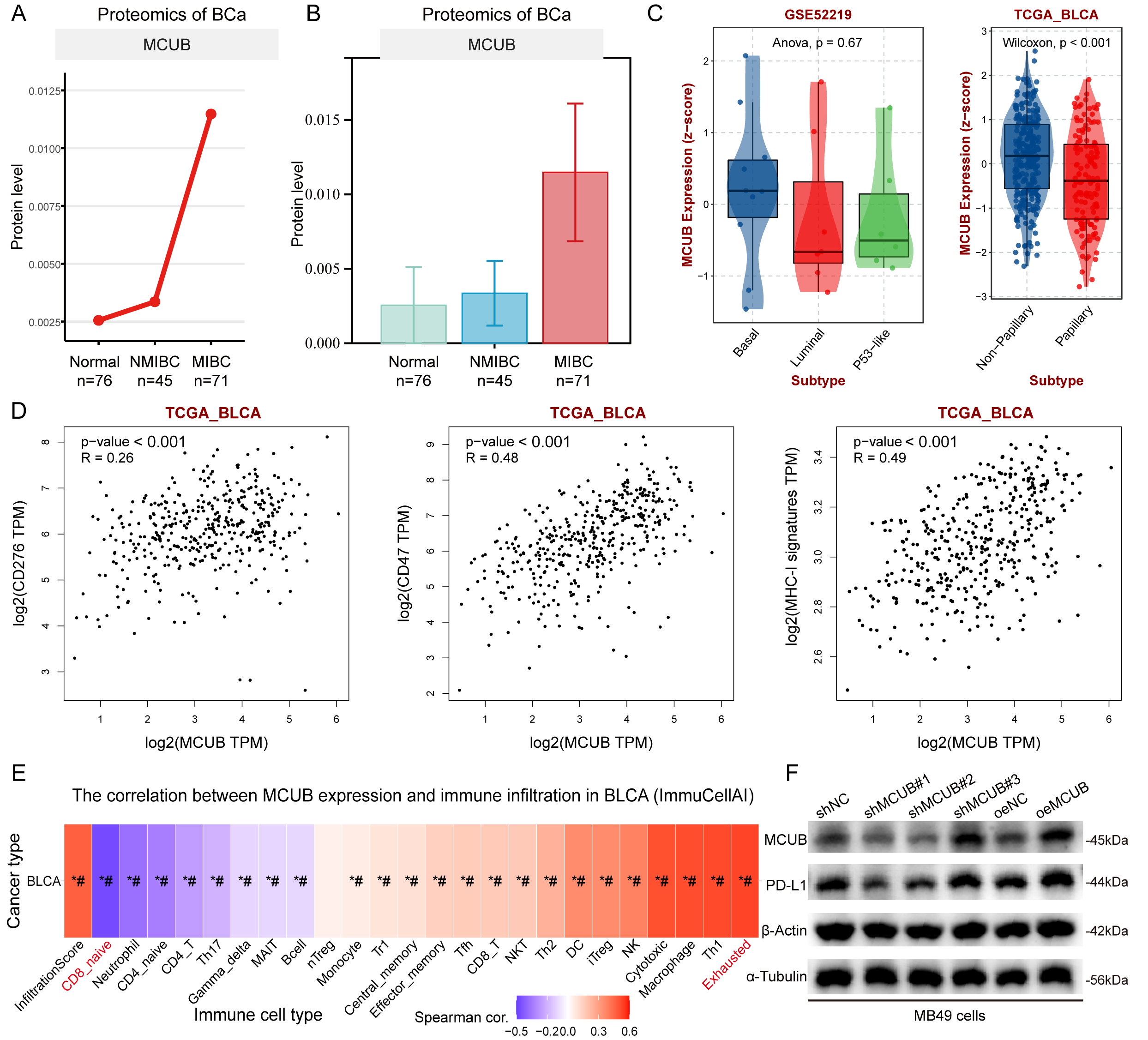
**

**Figure S1. Progressive upregulation of MCUB in MIBC and its correlation with PD-L1 and an immunosuppressive microenvironment.** (A-B) MCUB protein levels were analyzed in proteomic data from 192 bladder tissue samples, including normal bladder mucosa (n=76), NMIBC (n=45), and MIBC (n=71), and were visualized as a line plot (A) and bar graph (B), both showing a marked increase in MCUB expression from normal tissue to MIBC. (C) The expression of MCUB across BCa molecular subtypes. (D) Correlation analysis in TCGA-BLCA revealed that MCUB expression was positively associated with CD276, CD47, and MHC-I signatures. (E) The immune infiltration analysis in TCGA-BLCA dataset using ImmuCellAI algorithm. The results showed that MCUB expression correlated positively with exhausted cells and negatively with naïve CD8+ T cells, suggesting an immunosuppressive landscape in MCUB-high tumors. (F) Western blotting was performed in MB49 cells stably expressing MCUB-targeting shRNAs (shMCUB#1-#3), control shRNA (shNC), MCUB overexpression construct (oeMCUB), or empty vector (oeNC). The MCUB knockdown decreased PD-L1 protein expression, whereas MCUB overexpression elevated PD-L1 levels. β-Actin and α-Tubulin were used as loading controls. **p*<0.05.

**
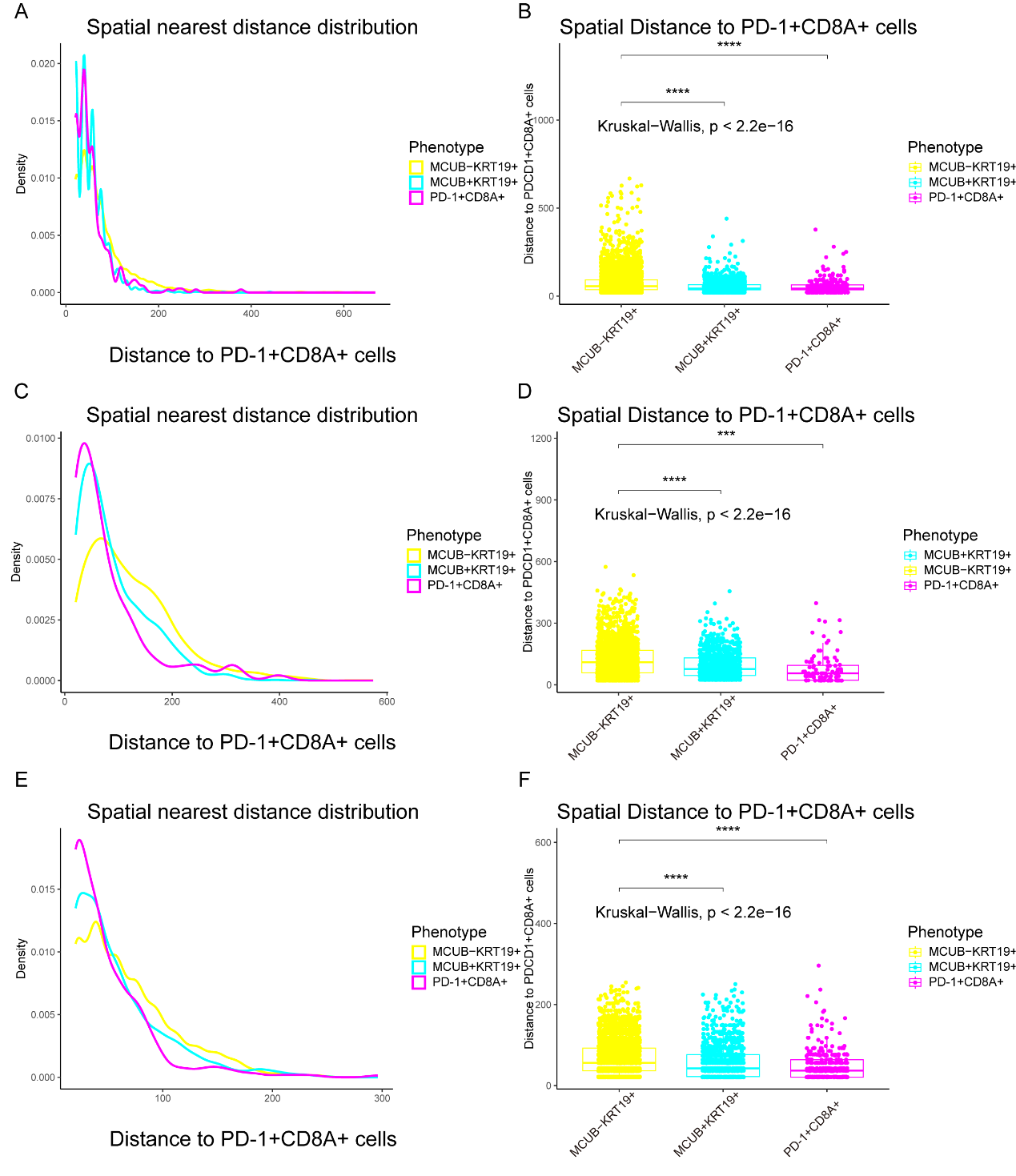
**

**Figure S2. MCUB⁺KRT19⁺ epithelial cells were spatially positioned closer to PD-1⁺CD8A⁺ T cells in MIBC tissues.** (A-F) Spatial distance analyses were performed across three spatial transcriptomic samples to assess the proximity of epithelial subtypes to PD-1⁺CD8A⁺ T cells. (A, C, E) Density distribution plots of the nearest spatial distances to PD-1⁺CD8A⁺ cells from three cell phenotypes: MCUB⁻KRT19⁺ (yellow), MCUB⁺KRT19⁺ (cyan), and PD-1⁺CD8A⁺ (magenta), derived from sections GSM8171243 (A), GSM8171245 (C), and GSM8171247 (E). (B, D, F) Boxplots comparing the spatial distances to PD-1⁺CD8A⁺ cells among the three phenotypes in the corresponding sections, showing that MCUB⁺KRT19⁺ epithelial cells were significantly closer to PD-1⁺CD8A⁺ T cells than MCUB⁻KRT19⁺ cells. Statistical significance was assessed using the Kruskal-Walli’s test. **p*<0.05, ***p*<0.01, ****p*<0.001, *****p*<0.0001.


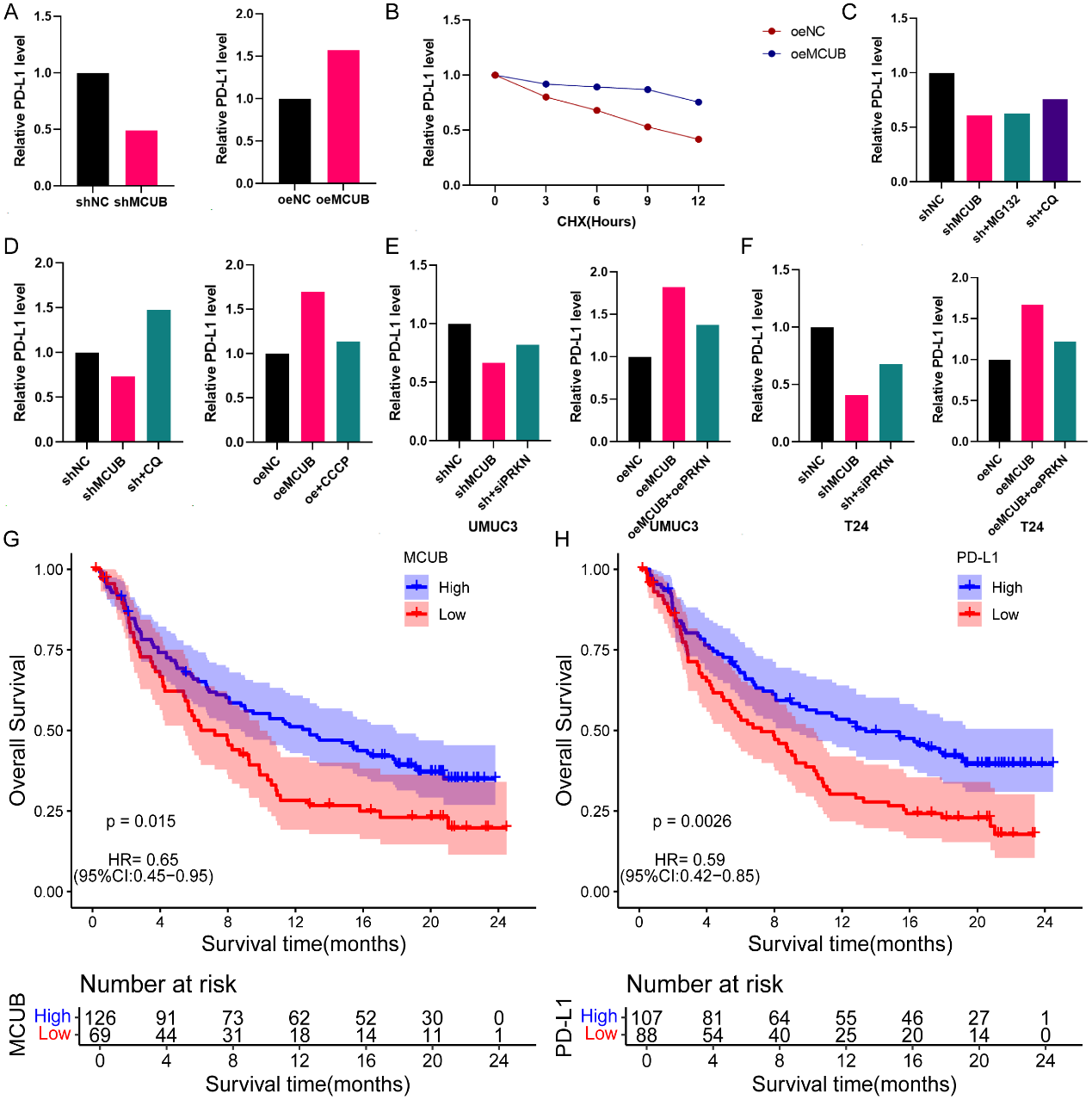


**Figure S3. MCUB regulated PD-L1 expression and predicted clinical outcomes in BCa patients receiving anti-PD-L1 therapy (IMvigor210 cohort).** (A) Western blotting quantification showing that MCUB knockdown decreased PD-L1 protein levels, whereas MCUB overexpression increased PD-L1 levels. (B) Cycloheximide (CHX) chase assays showing that MCUB overexpression prolonged PD-L1 protein half-life. (C) Treatment with MG132 (proteasome inhibitor) or chloroquine (lysosome inhibitor) confirmed that PD-L1 degradation was primarily lysosome-dependent. (D) PD-L1 expression changes upon MCUB modulation combined with chloroquine (CQ) or CCCP treatment, respectively. (E-F) PD-L1 expression changes upon MCUB modulation combined with PRKN knockdown or overexpression in UMUC3 (E) and T24 (F) cells. (G) The Kaplan-Meier survival analysis showing that MCUB-high patients had significantly longer overall survival compared with MCUB-low patients (*p*=0.015, HR=0.65, 95% CI: 0.45-0.95). (H) The Kaplan-Meier survival analysis showing that patients with PD-L1-high tumors also exhibited longer overall survival compared with PD-L1-low patients (*p*=0.0026, HR=0.59, 95% CI: 0.42-0.85). Numbers at risk are indicated below each plot.
