# Supplementary material for: MCUB Inhibits PRKN‐Dependent Mitophagic Degradation of PD‐L1 to Promote Immune Evasion in Bladder Cancer
Source: Adv Sci (Weinh). 2025 Nov 12;13(5):e14764. doi: 10.1002/advs.202514764 (PMC12849890; doi:10.1002/advs.202514764)

**Figure2:**

**
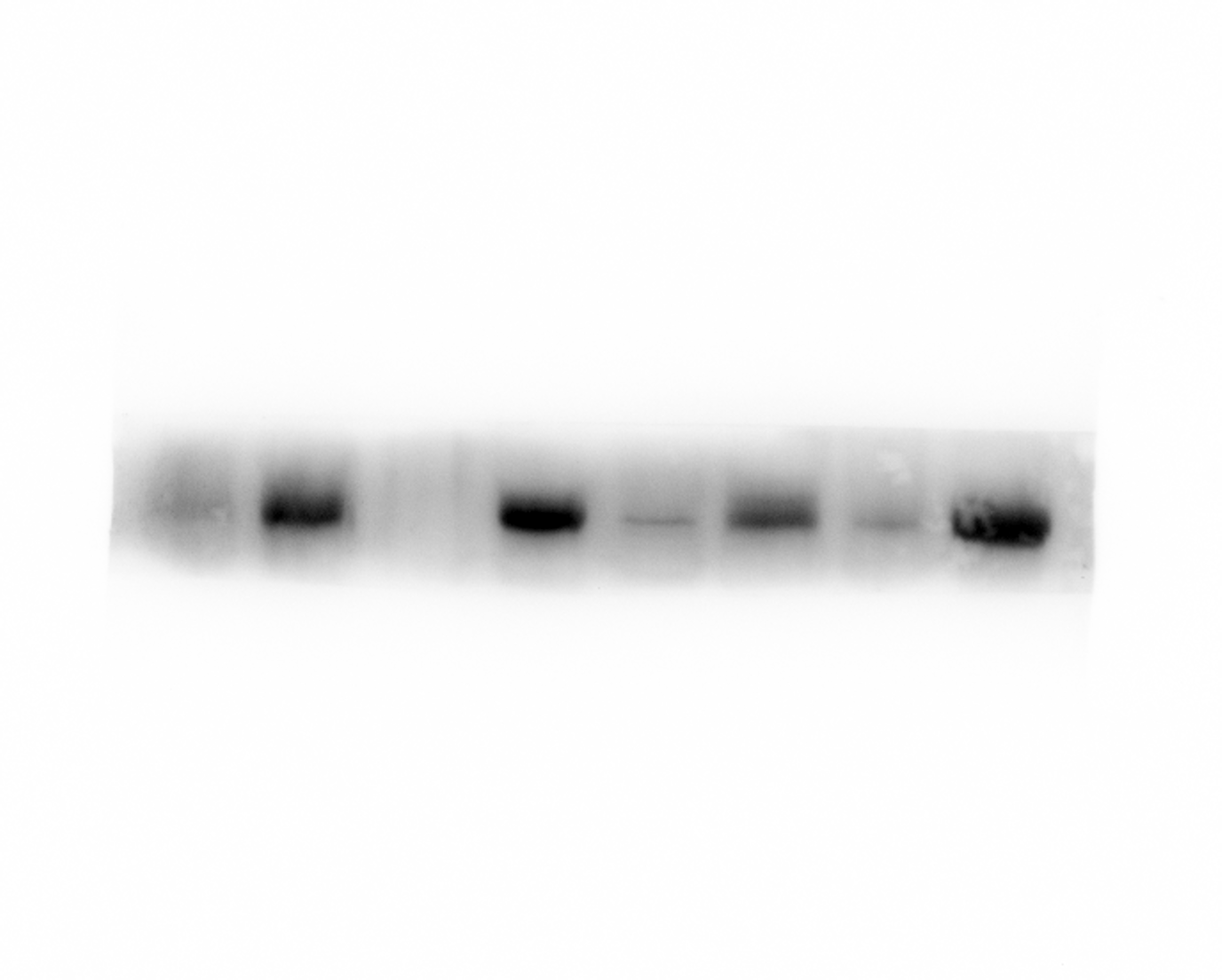
Figure2 D MCUB**

**
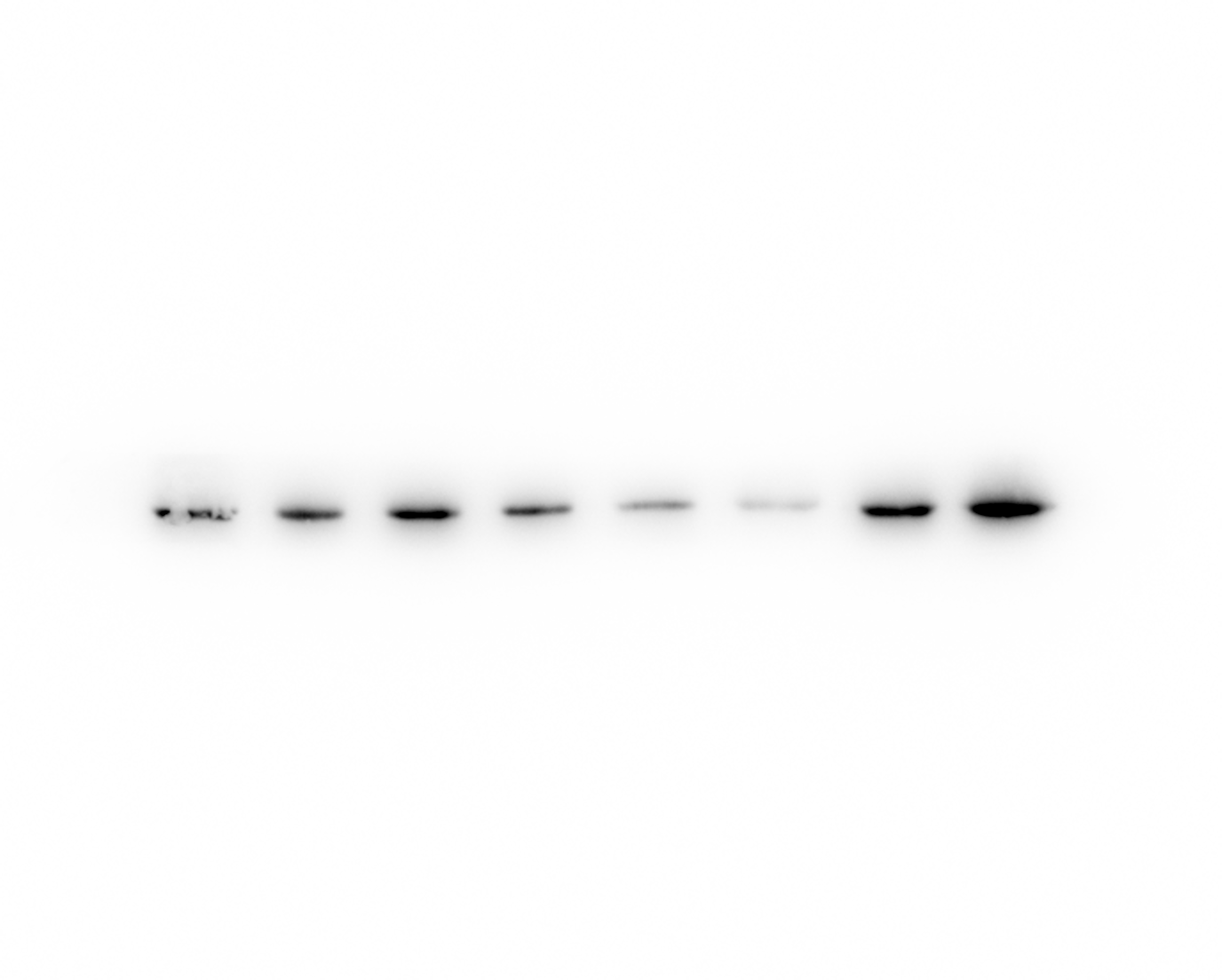
Figure2 D: GAPDH**

**
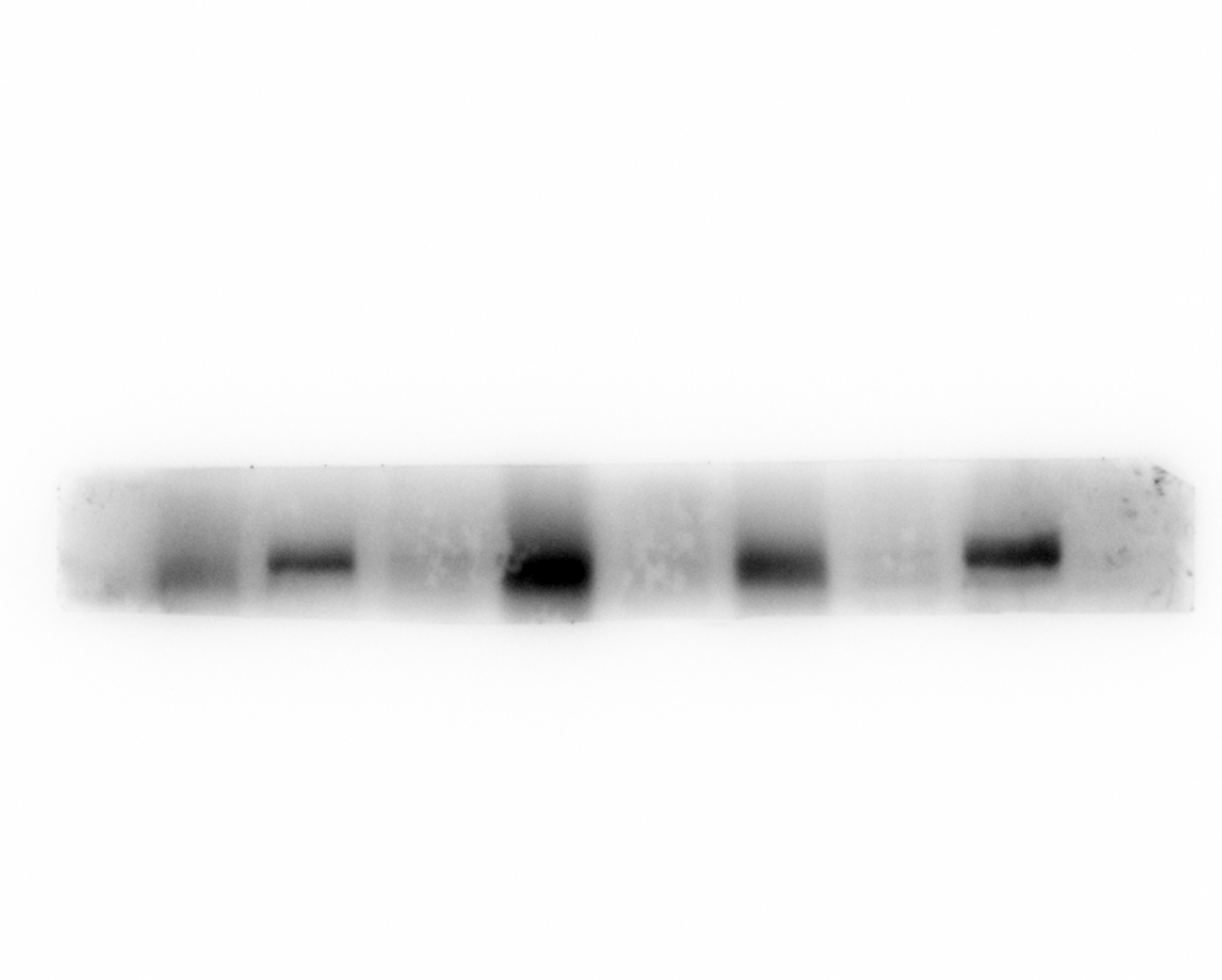
Figure2 D: MCUB**

**
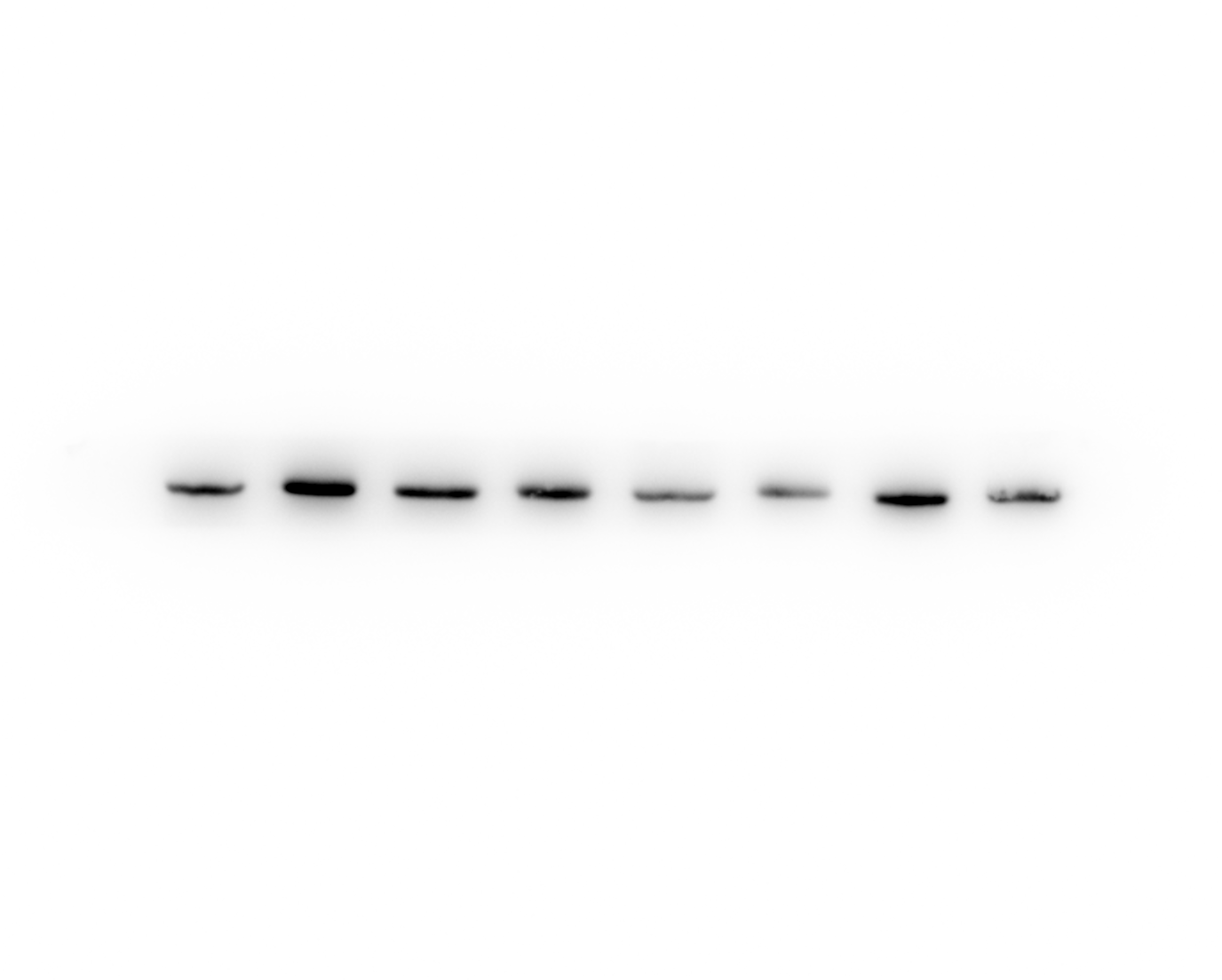
Figure2 D: GAPDH**


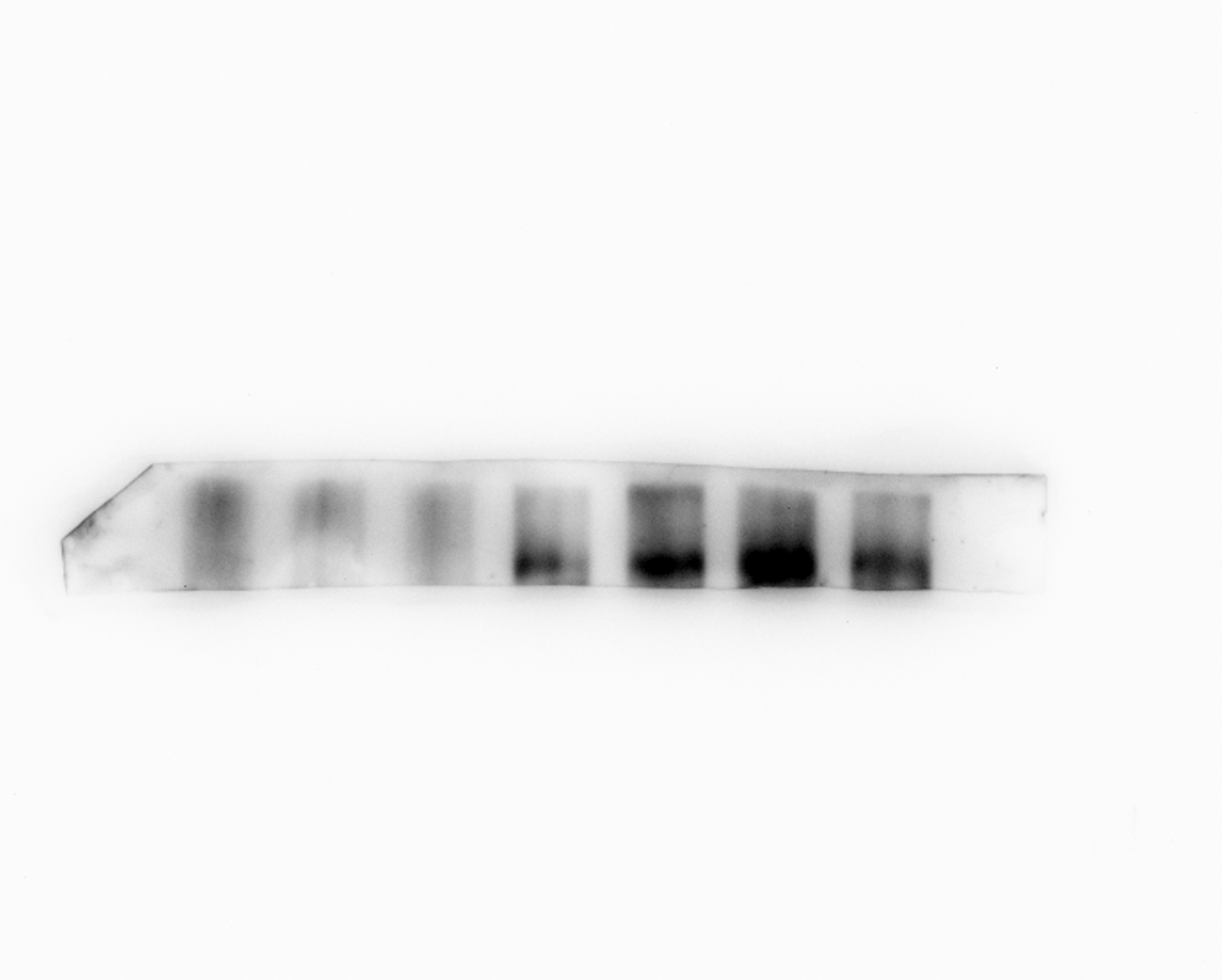
**Figure2 D: MCUB**

**
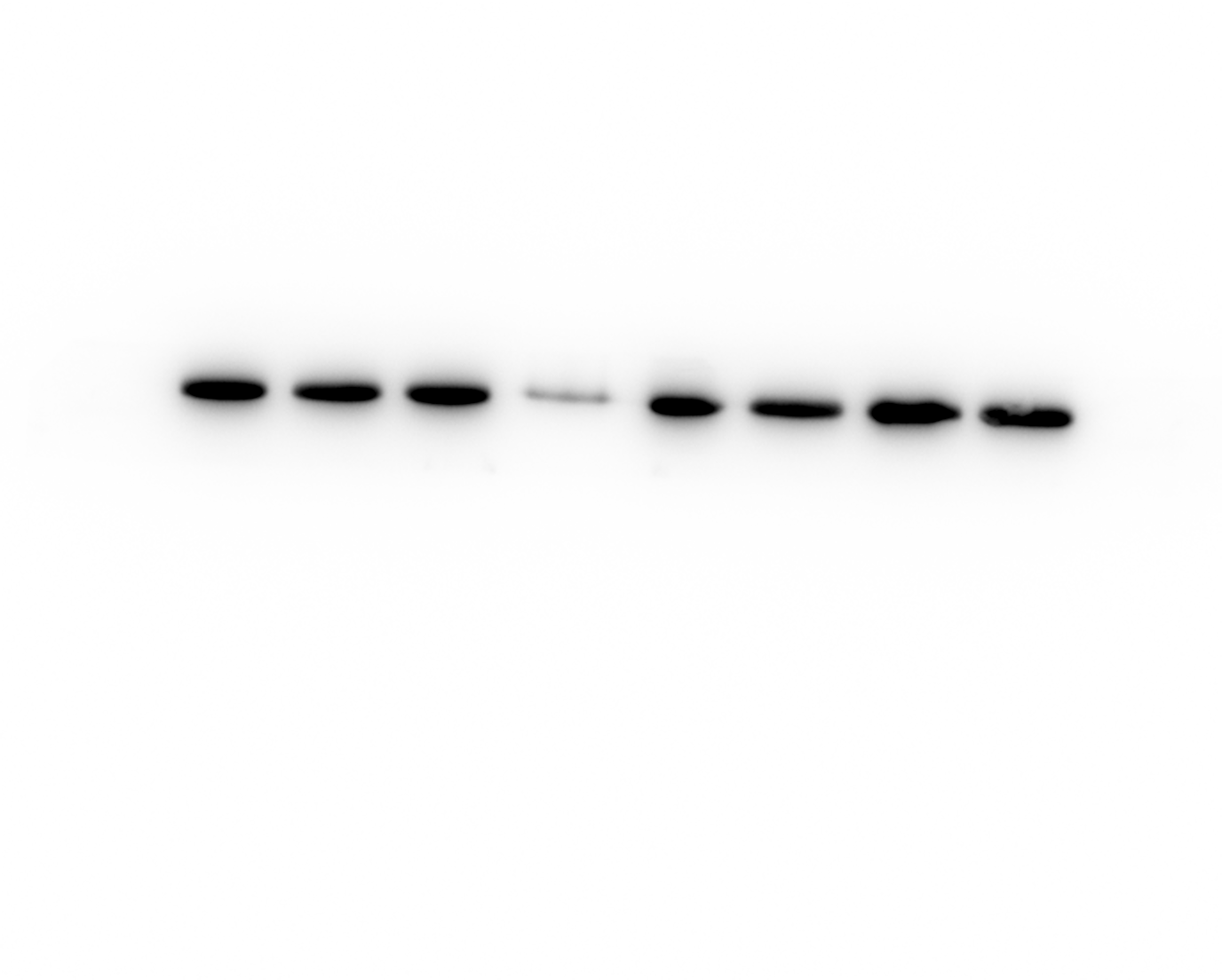
Figure2 D: GAPDH**

**Group: 3T 4T 5T Blank 11T 1T 2T 13T**

**Figure2 E:**

**Pt1 normal**


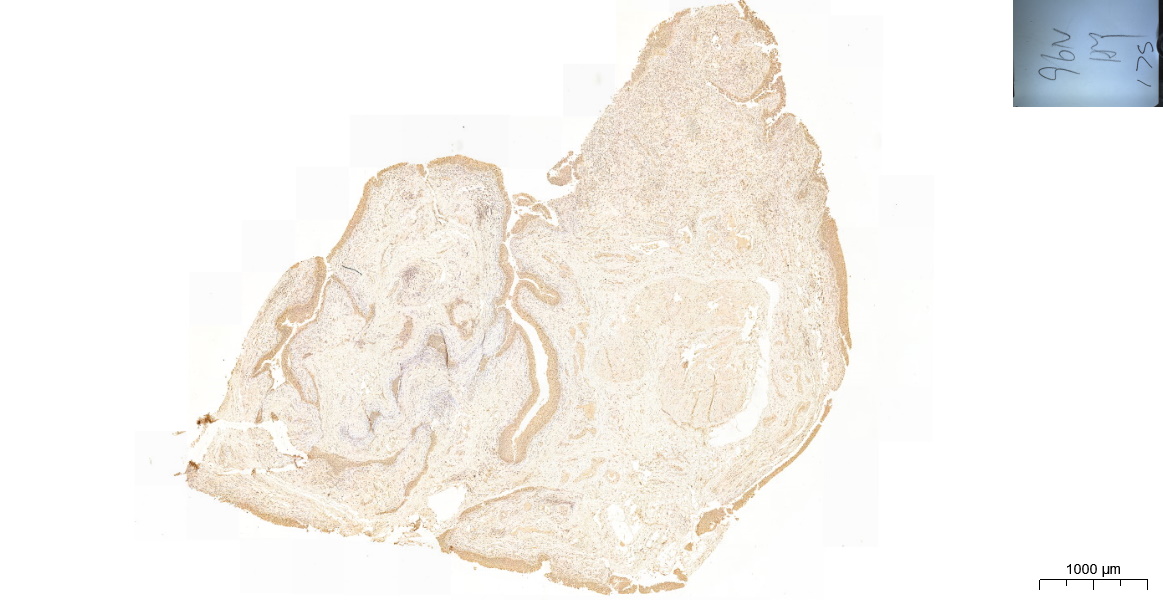


**Figure2 E:**

**Pt1 tumor**


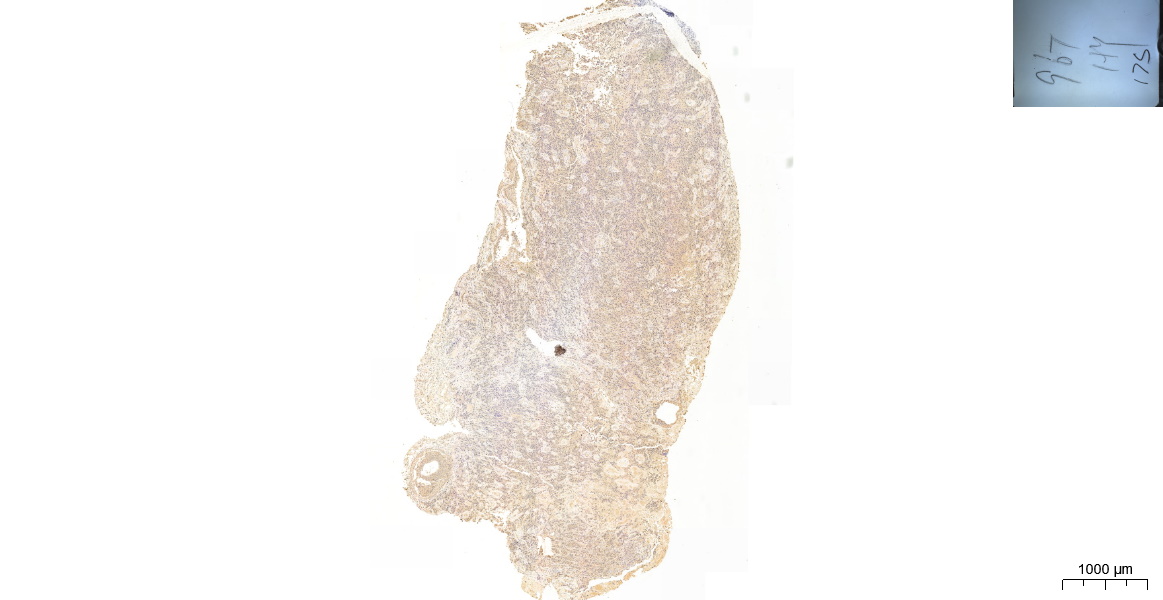


**Figure2 E:**

**Pt2 normal**


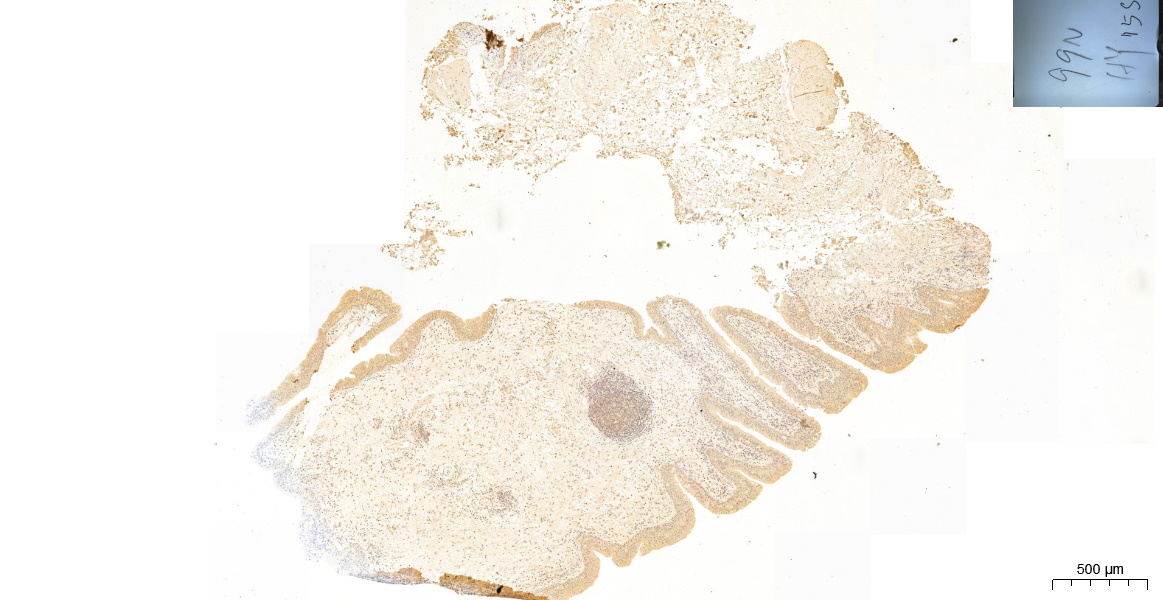


**Figure2 E:**

**Pt2 tumor**


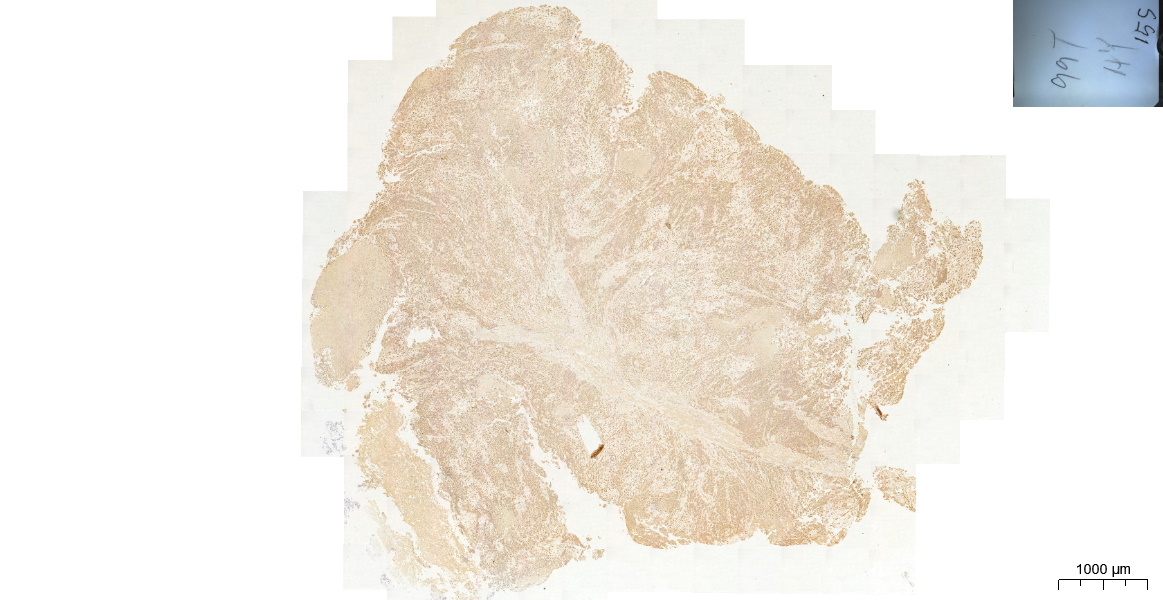


**Figure2 E:**

**Pt3 normal**


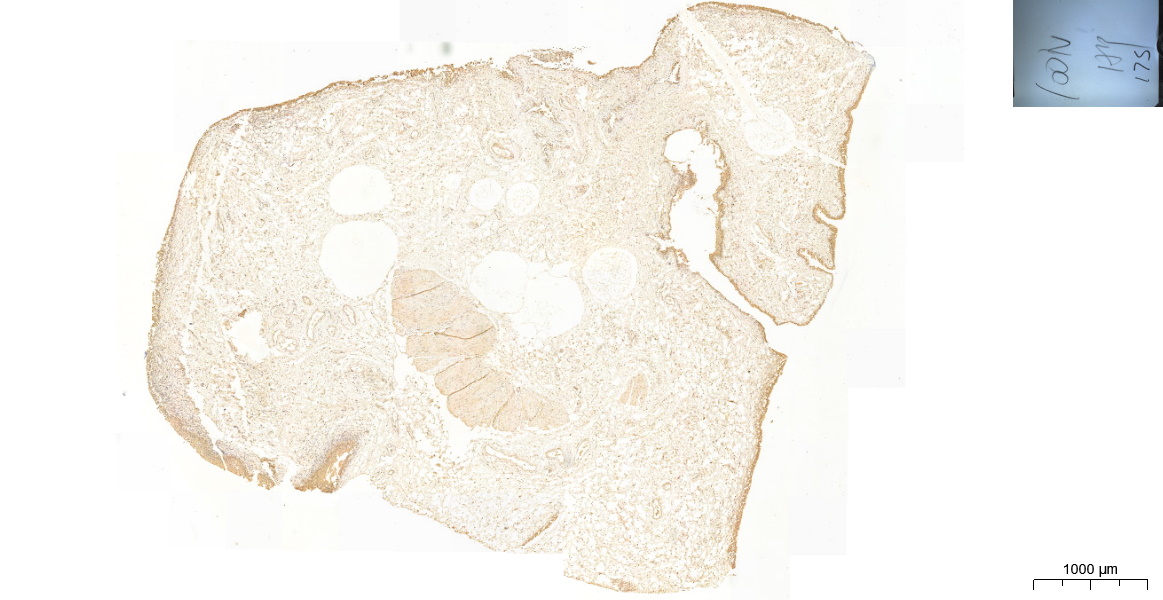


**Figure2 E:**

**Pt3 tumor**


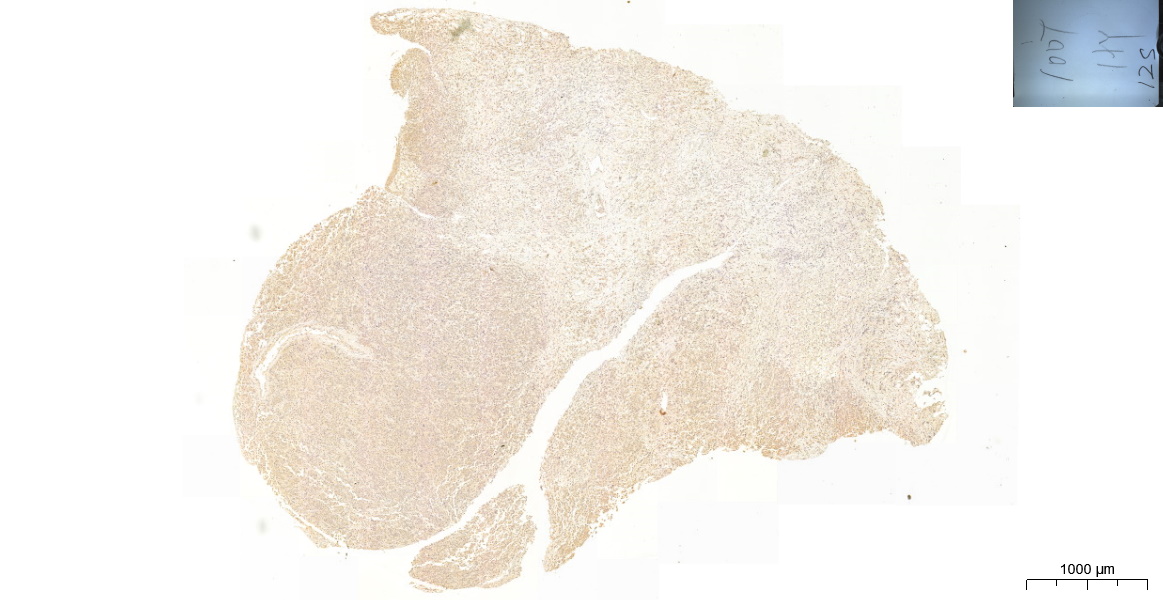


**Figure2 E:**

**Pt4 normal**


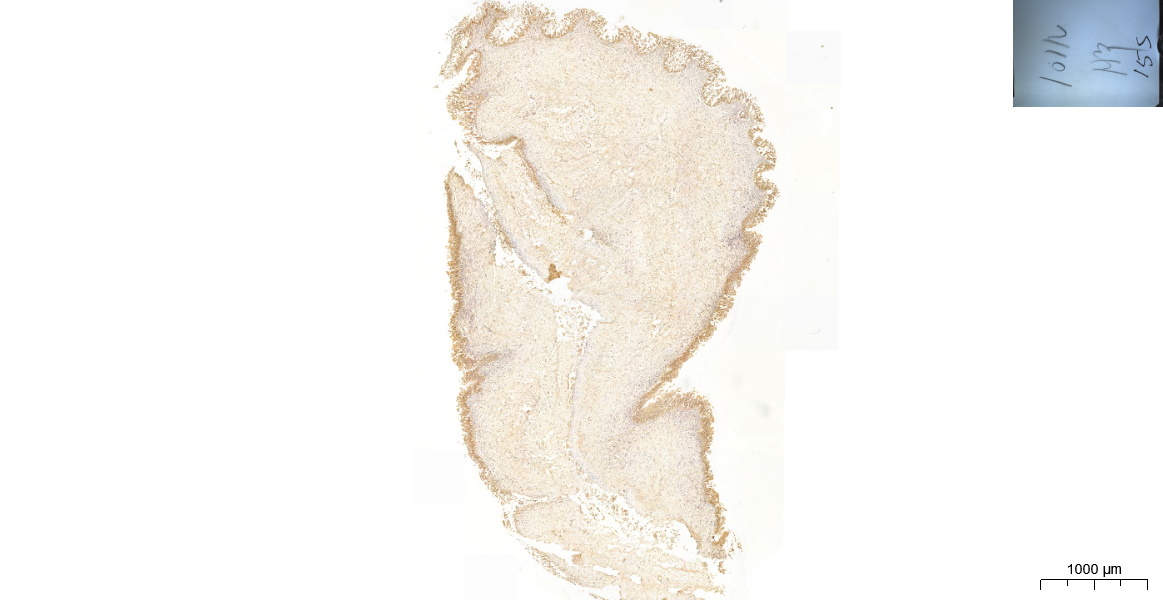


**Figure2 E:**

**Pt4 tumor**


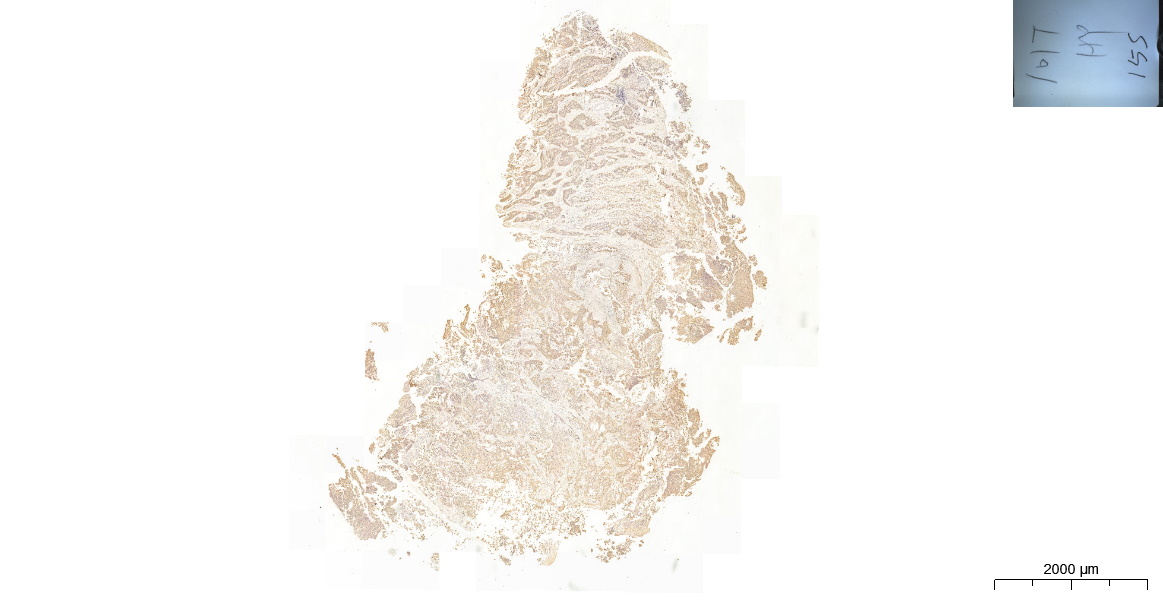


**Figure2 E:**

**Pt5 normal**


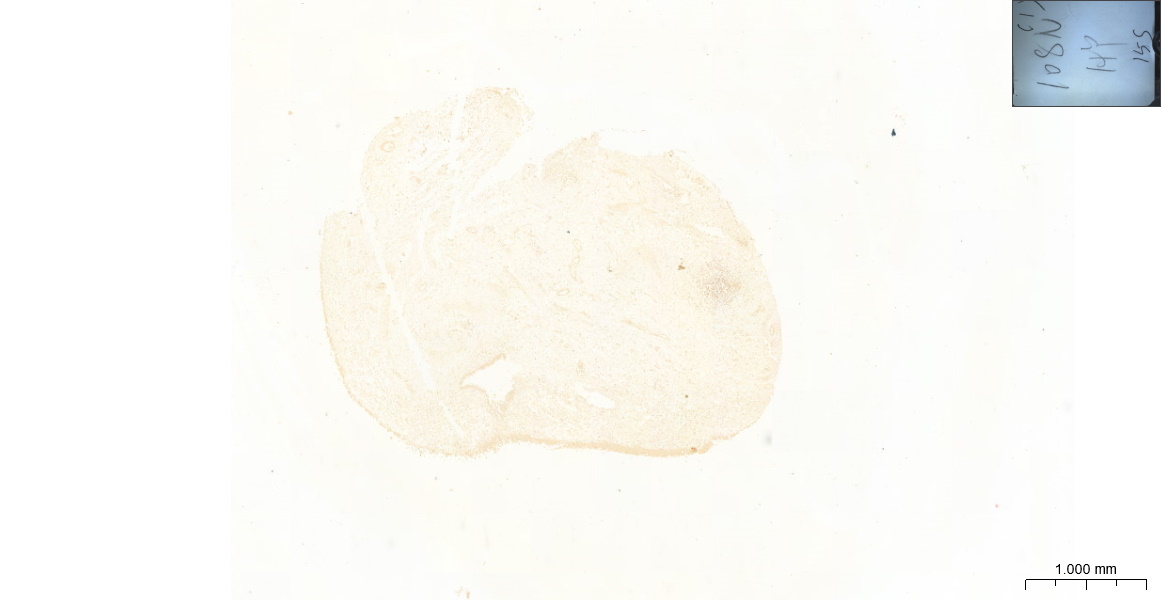


**Figure2 E:**

**Pt5 tumor**


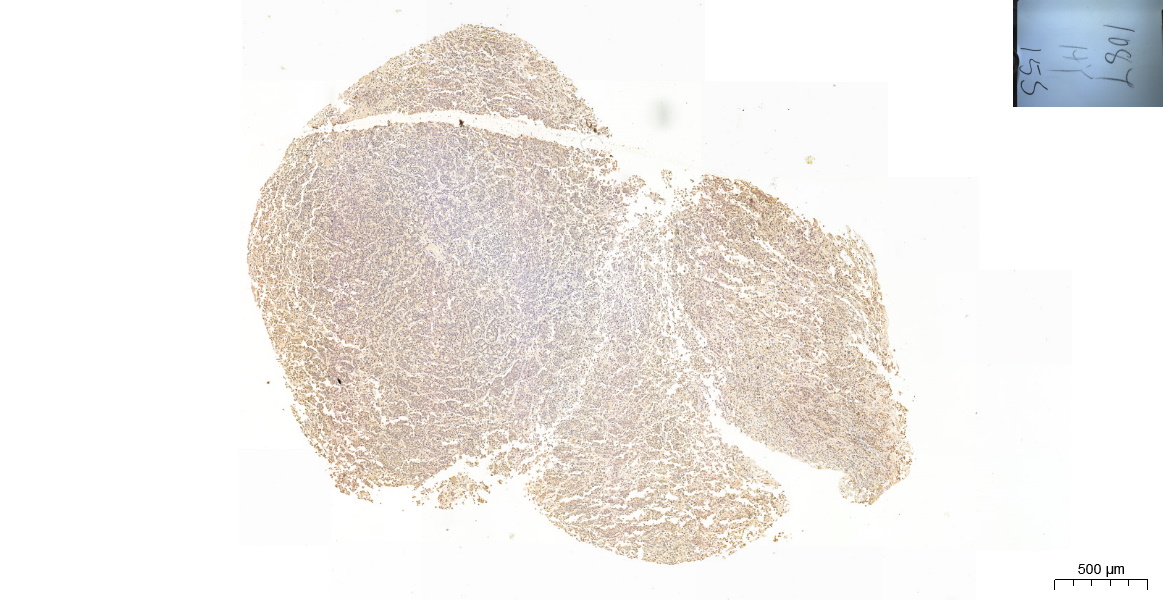


**Figure2 E:**

**Pt6 normal**


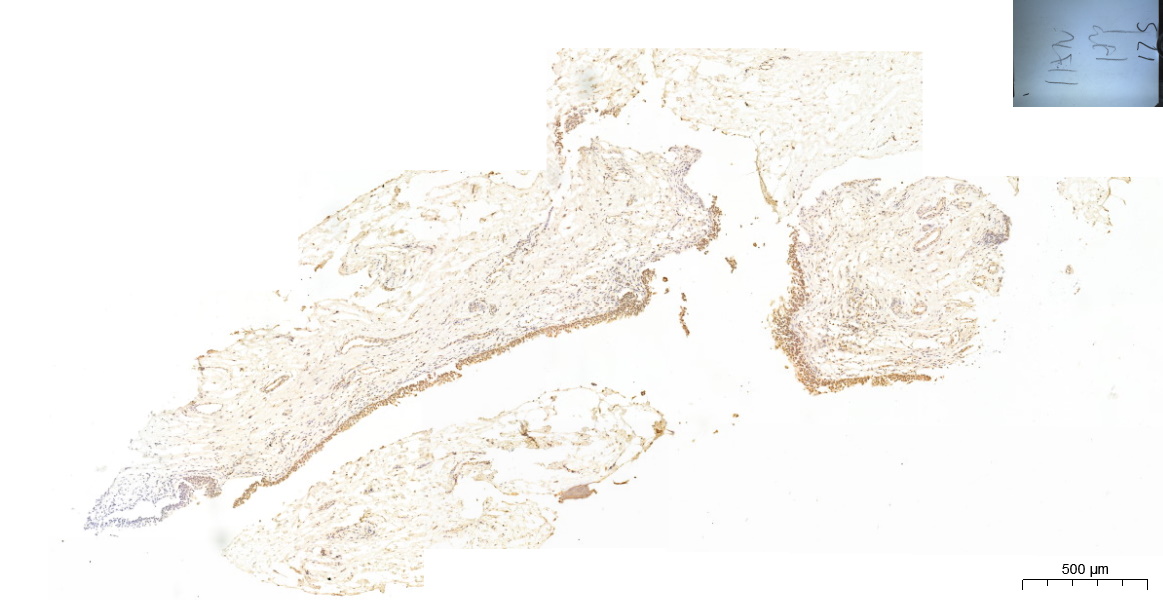


**Figure2 E:**

**Pt6 tumor**


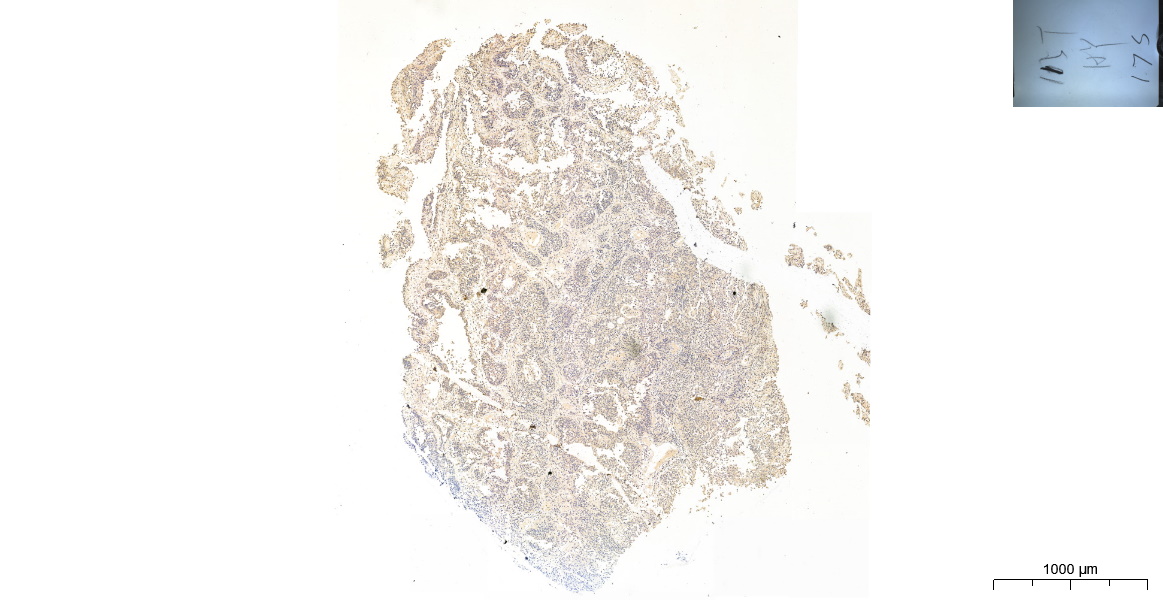

Supplement: Supplementary file 2 — Supporting Information [file ADVS-13-e14764-s002.zip › Figure2.docx]
