# Supplementary material for: MCUB Inhibits PRKN‐Dependent Mitophagic Degradation of PD‐L1 to Promote Immune Evasion in Bladder Cancer
Source: Adv Sci (Weinh). 2025 Nov 12;13(5):e14764. doi: 10.1002/advs.202514764 (PMC12849890; doi:10.1002/advs.202514764)

**Figure6 A:**

**Patient1 PD-L1**


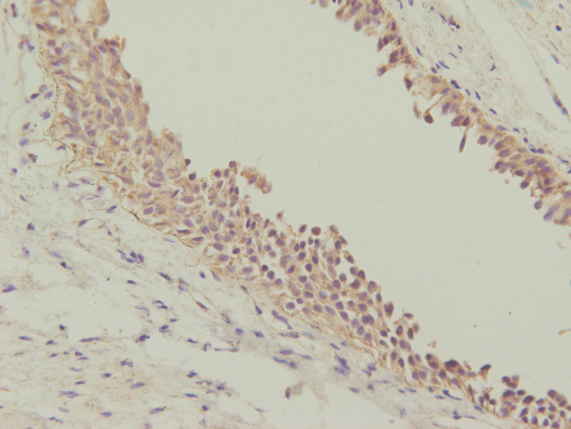


**Figure6 A:**

**Patient1 MCUB**


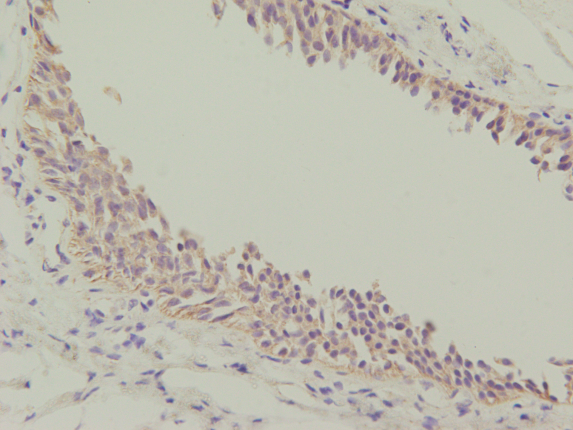


**Figure6 A:**

**Patient2 PD-L1**


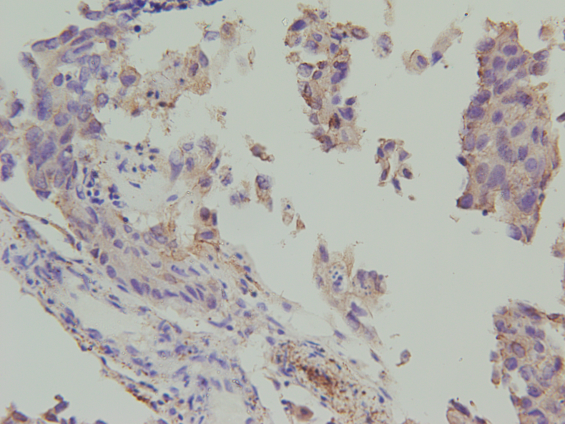


**Figure6 A:**

**Patient2 MCUB**


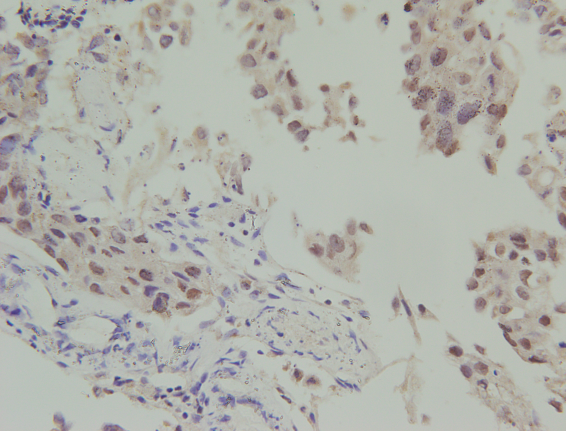


**Figure6 A:**

**Patient3 PD-L1**


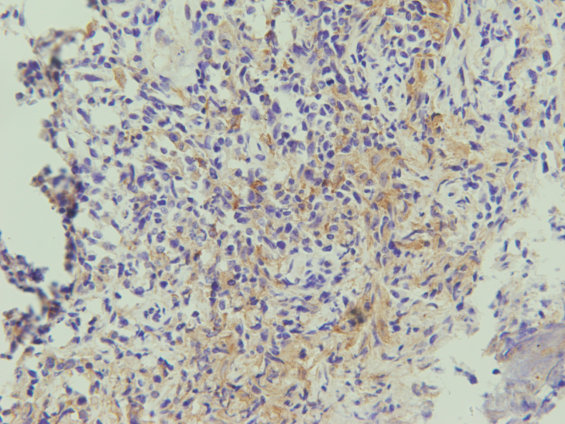


**Figure6 A:**

**Patient3 MCUB**


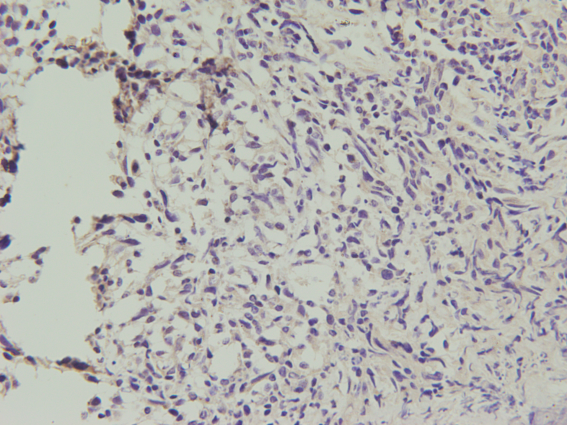


**Figure6 A:**

**Patient4 PD-L1**


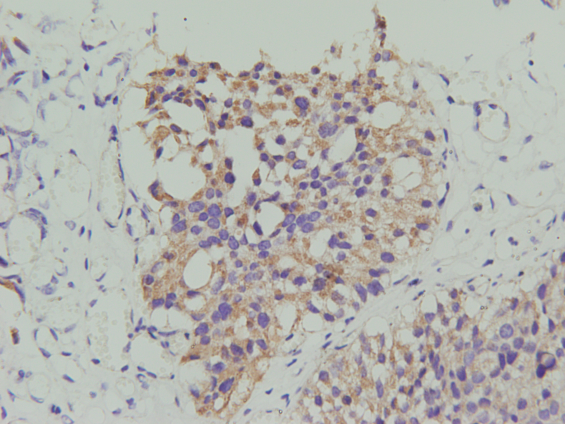


**Figure6 A:**

**Patient4 MCUB**


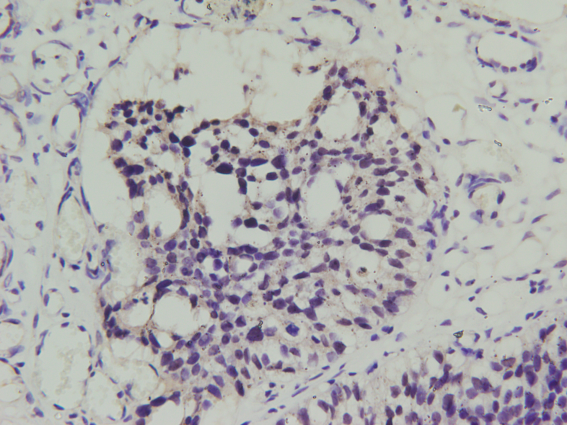


**Figure6 A:**

**Patient5 PD-L1**


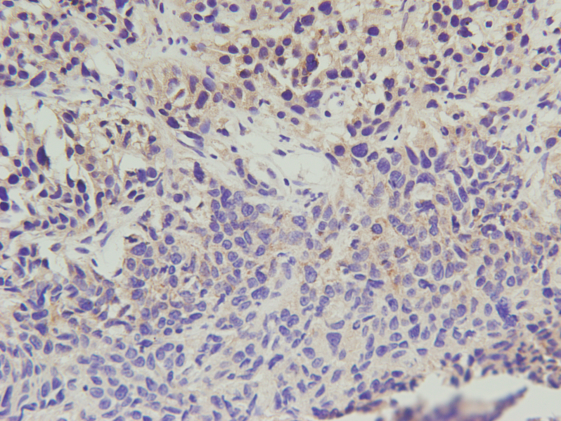


**Figure6 A:**

**Patient5 MCUB**


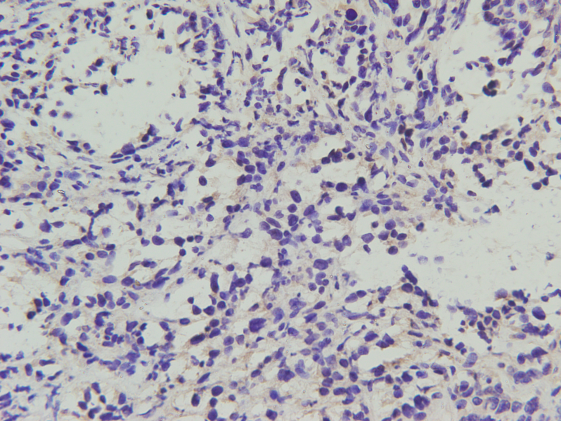


**Figure6 A:**

**Patient6 PD-L1**


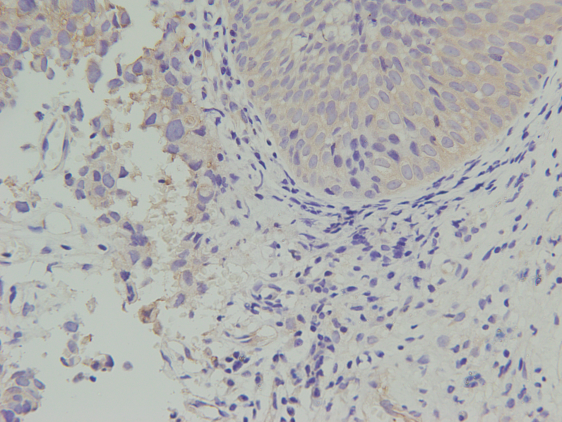


**Figure6 A:**

**Patient6 MCUB**


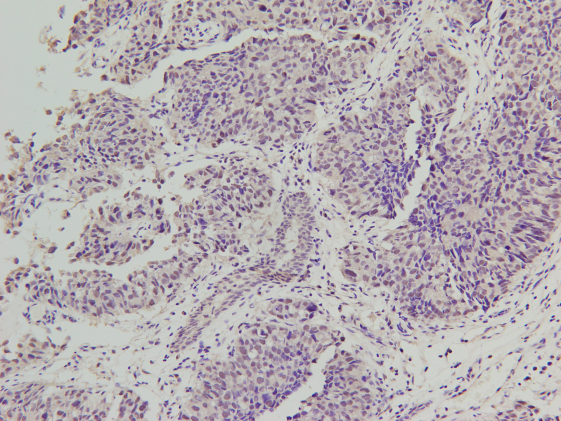


**Figure6 A:**

**Patient7 PD-L1**


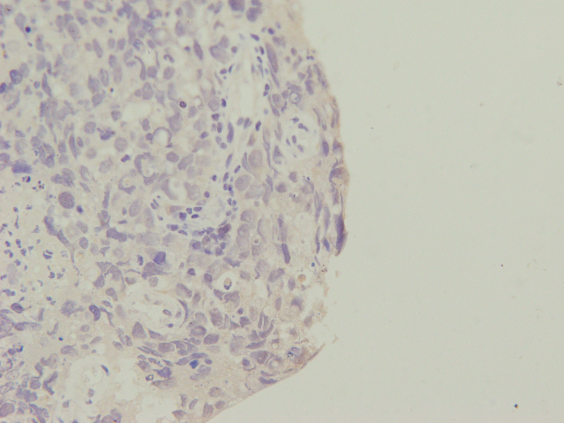


**Figure6 A:**

**Patient7 MCUB**


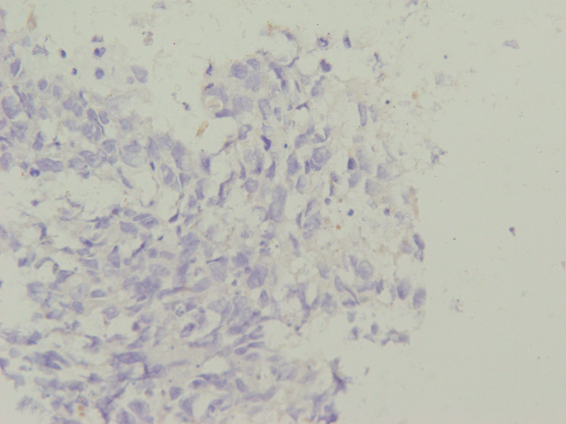


**Figure6 A:**

**Patient8 PD-L1**


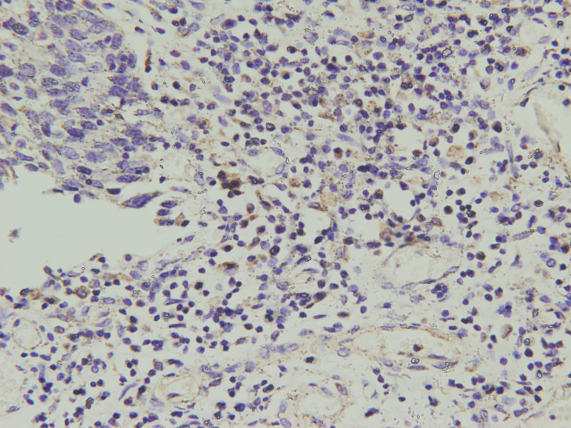


**Figure6 A:**

**Patient8 MCUB**


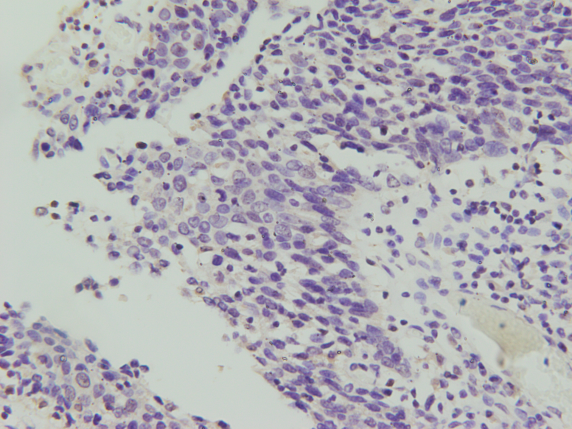


**Figure6 A:**

**Patient9 PD-L1**


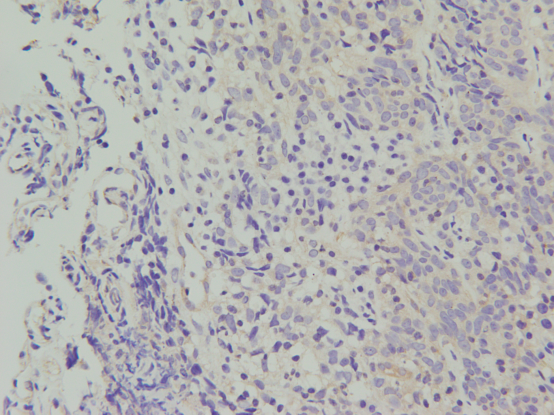


**Figure6 A:**

**Patient9 PD-L1**


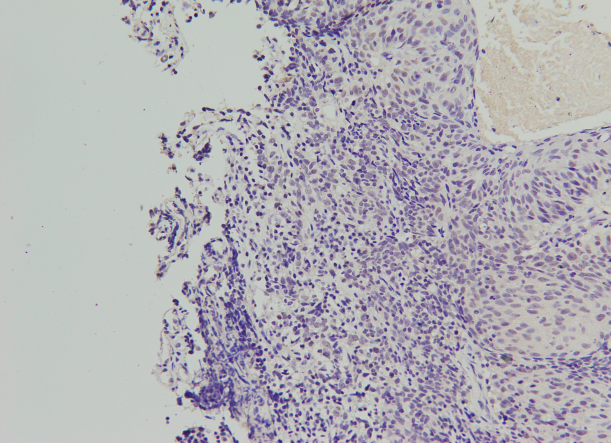


**Figure6 A:**

**Patient10 PD-L1**


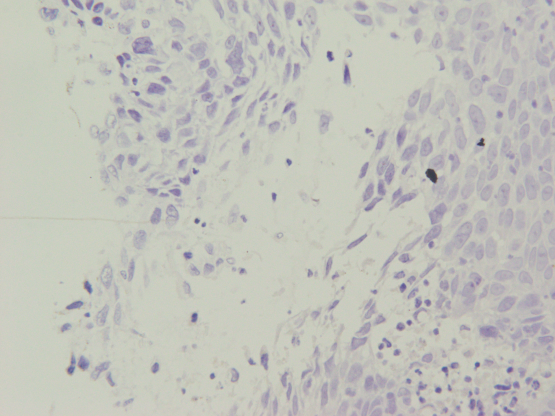


**Figure6 A:**

**Patient10 MCUB**


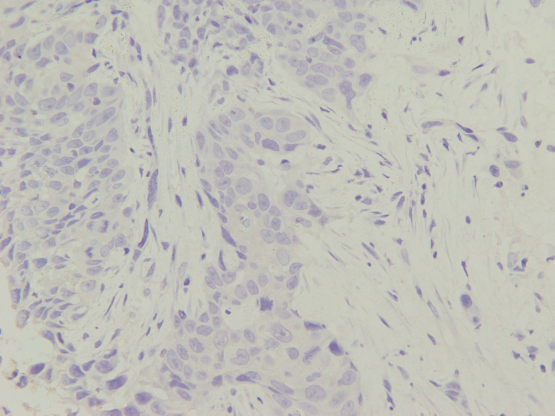


**Figure6 C, left: MCUB**

**Group：(1) shNC (2) shMCUB**

**
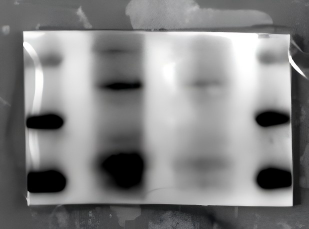
**

**Figure6 C, left: PD-L1**

**Group：(1) shNC (2) shMCUB (3) shMCUB+MG132 (4) shMCUB+CQ**

**
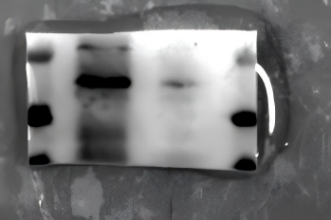
**

**Figure6 C, left: β-Actin**

**Group：(1) shNC (2) shMCUB (3) shMCUB+MG132 (4) shMCUB+CQ**

**
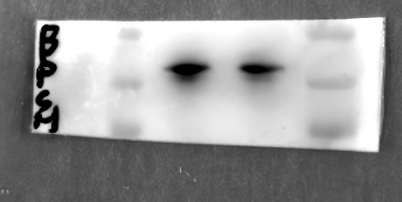
**

**Figure6 C, right: MCUB**

**Group：(1) oeNC (2) oeMCUB**

**
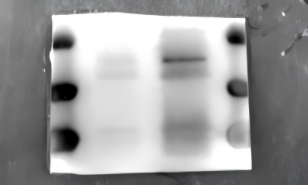
**

**Figure6 C, right: PD-L1**

**Group：(1) oeNC (2) oeMCUB**

**
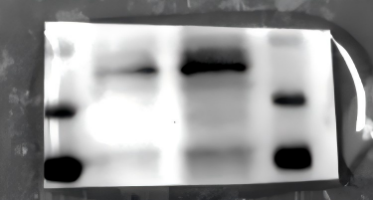
**

**Figure6 C, right:β-Actin**

**Group：(1) oeNC (2) oeMCUB**

**
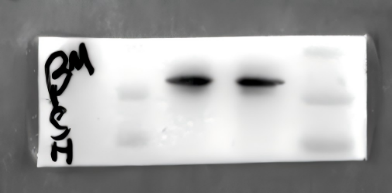
**

**Figure6 D, left**


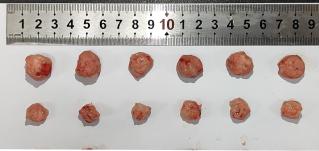


**Figure6 D, right**


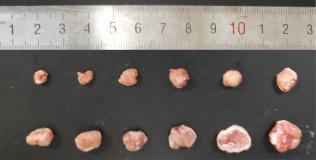


**Figure6 E:**

**shNC:**


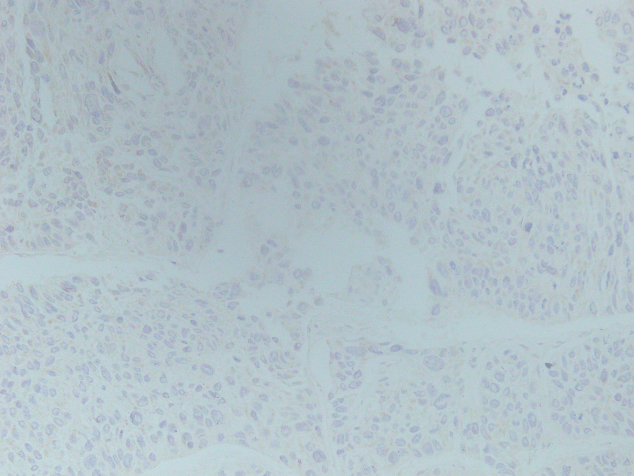

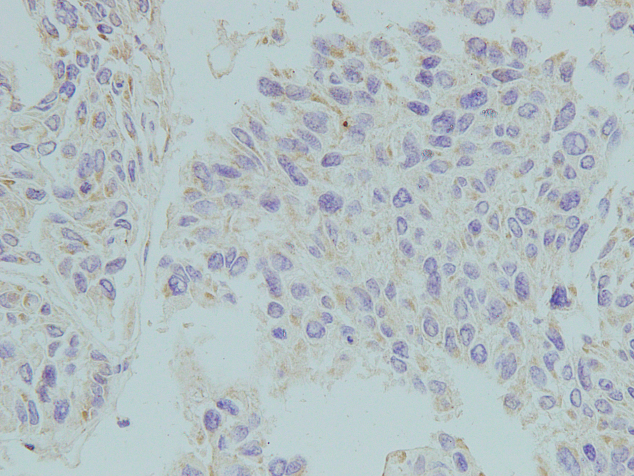


**Figure6 E:**

**shMCUB:**


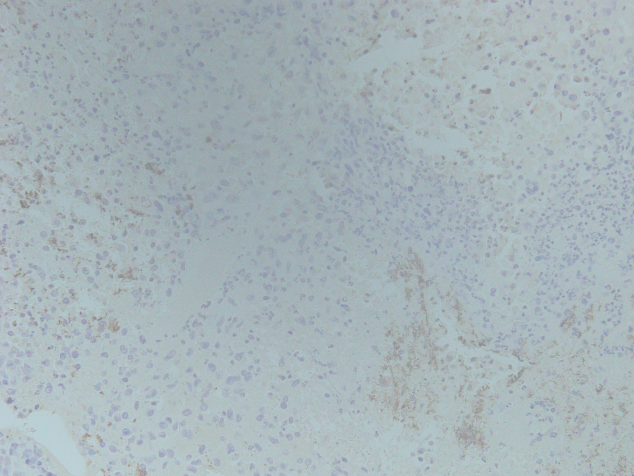


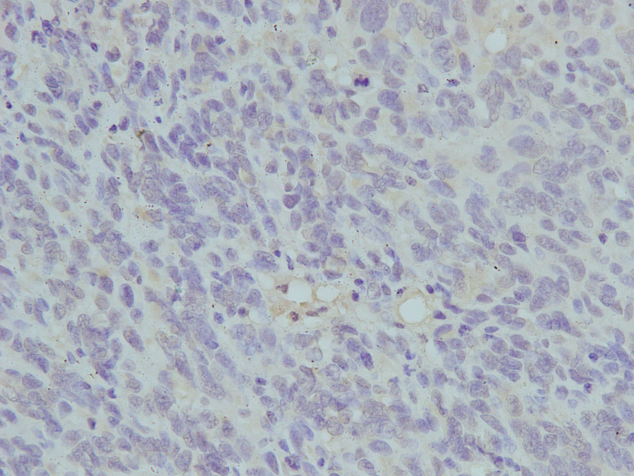


**Figure6 E:**

**oeNC:**


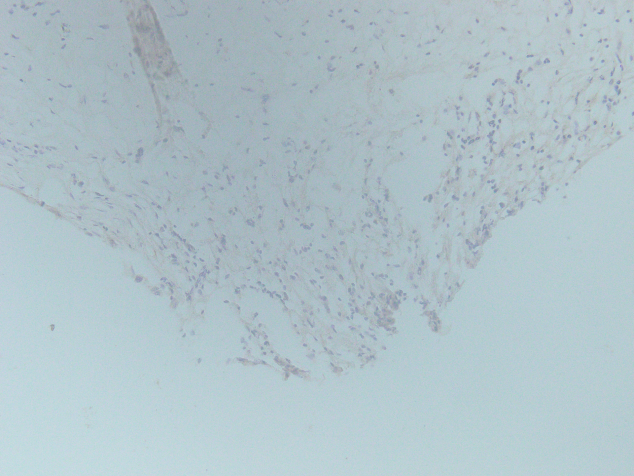

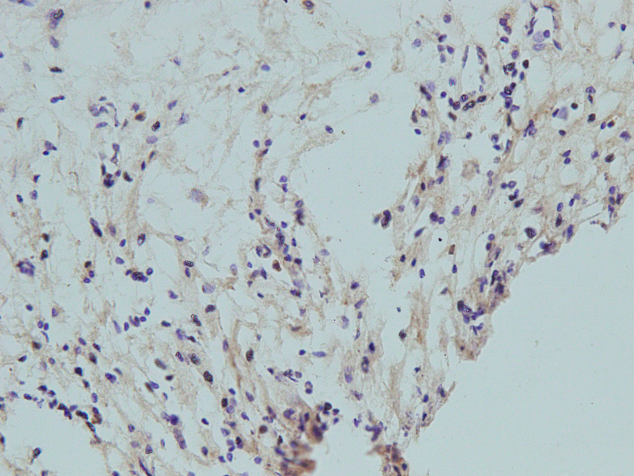


**Figure6 E:**

**oeMCUB:**


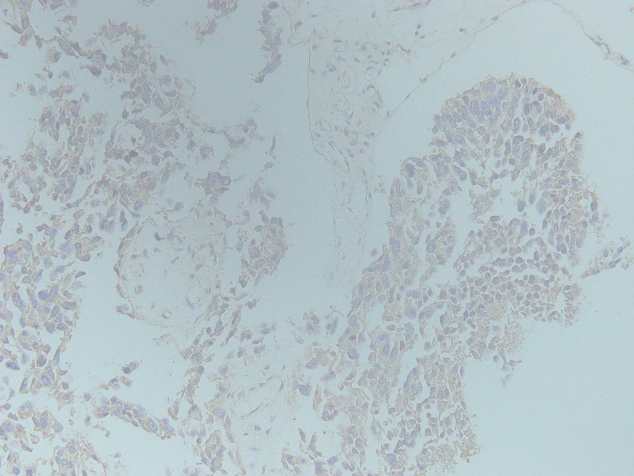

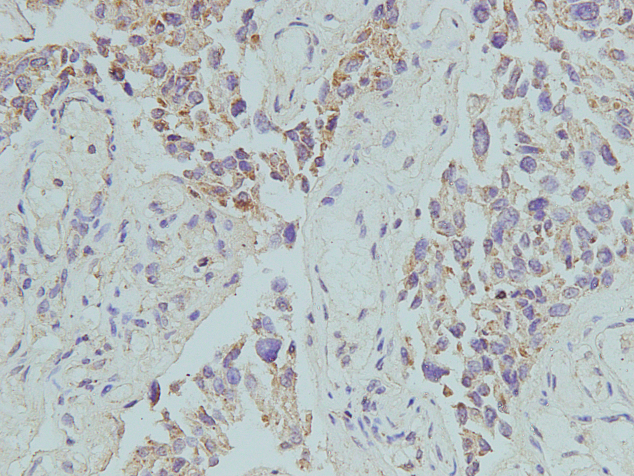

Supplement: Supplementary file 2 — Supporting Information [file ADVS-13-e14764-s002.zip › Figure6.docx]
