# Supplementary material for: MCUB Inhibits PRKN‐Dependent Mitophagic Degradation of PD‐L1 to Promote Immune Evasion in Bladder Cancer
Source: Adv Sci (Weinh). 2025 Nov 12;13(5):e14764. doi: 10.1002/advs.202514764 (PMC12849890; doi:10.1002/advs.202514764)
Supplement: Supplementary file 2 — Supporting Information [file ADVS-13-e14764-s002.zip › Figure7.docx]

**Figure7**

**Figure7 A, left: MCUB**

**Group：(1) shNC (2) shMCUB**

**
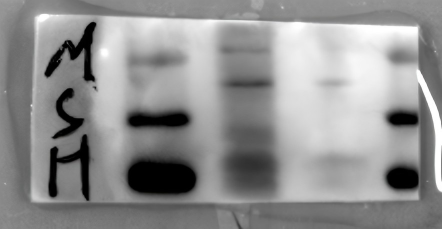
**

**Figure7 A, left: PD-L1**

**Group：(1) shNC (2) shMCUB (3) shMCUB+MG132 (4) shMCUB+CQ**

**
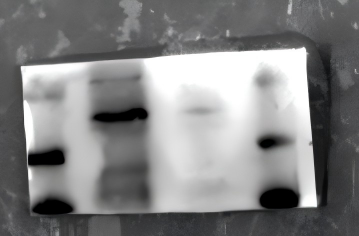
**

**Figure7 A, left: β-Actin**

**Group：(1) shNC (2) shMCUB (3) shMCUB+MG132 (4) shMCUB+CQ**

**
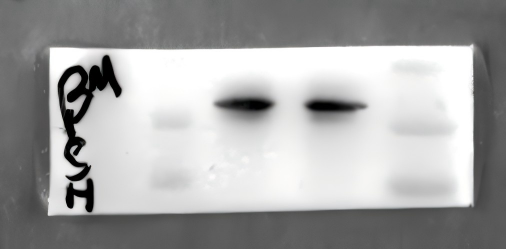
**

**Figure7 A, right: MCUB**

**Group：(1) oeNC (2) oeMCUB**

**
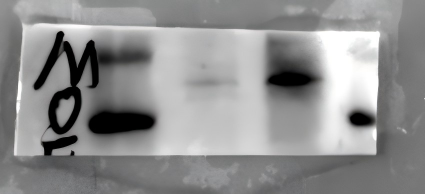
**

**Figure7 A, right: PD-L1**

**Group：(1) oeNC (2) oeMCUB**

**
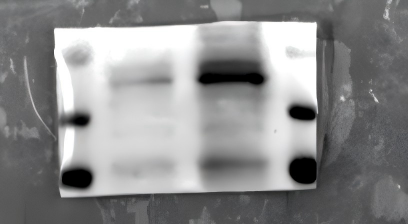
**

**Figure7 A, right: β-Actin**

**Group：(1) oeNC (2) oeMCUB**


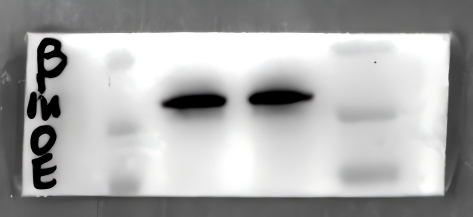


**
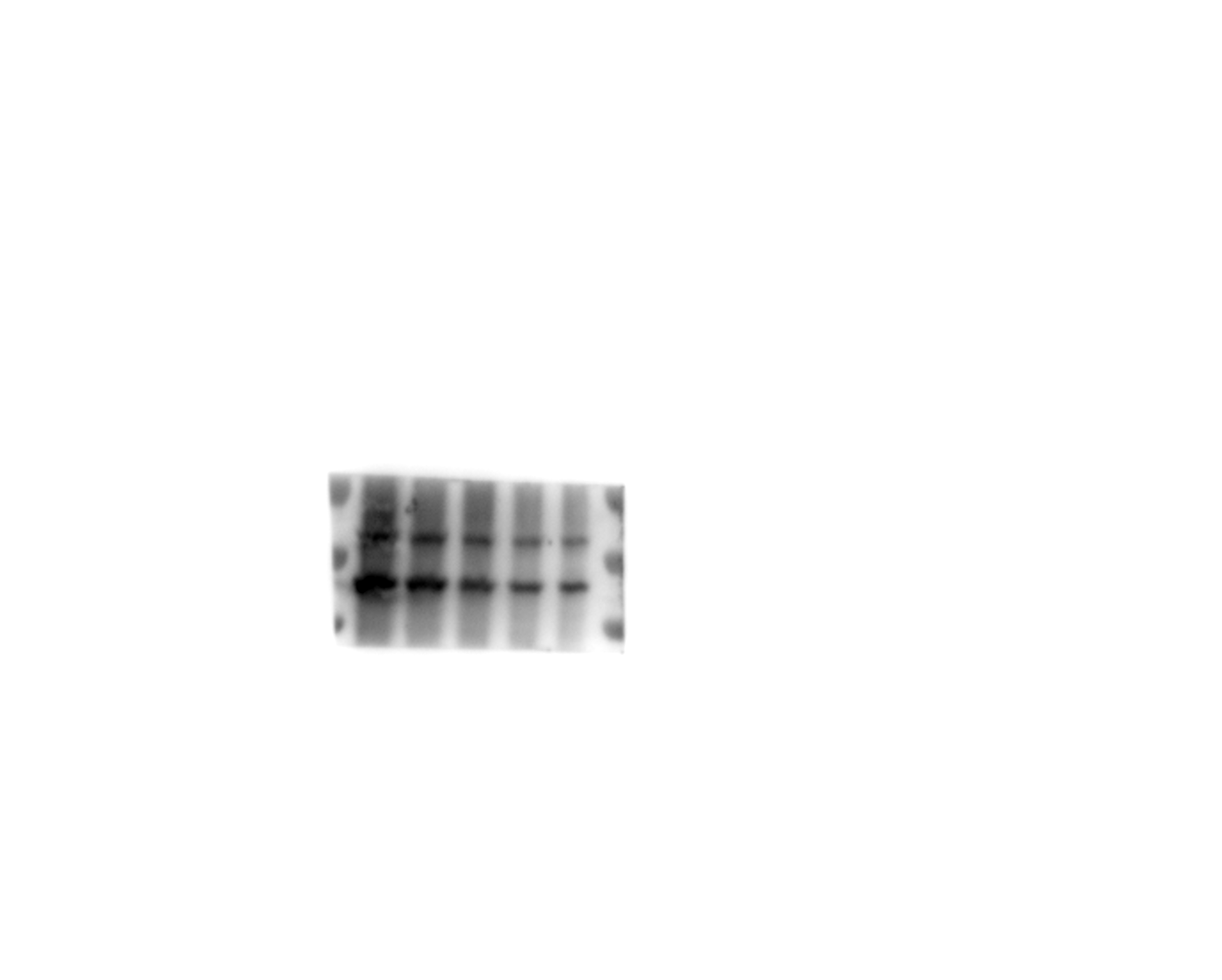
 Figure7 C, left: PD-L1**

**
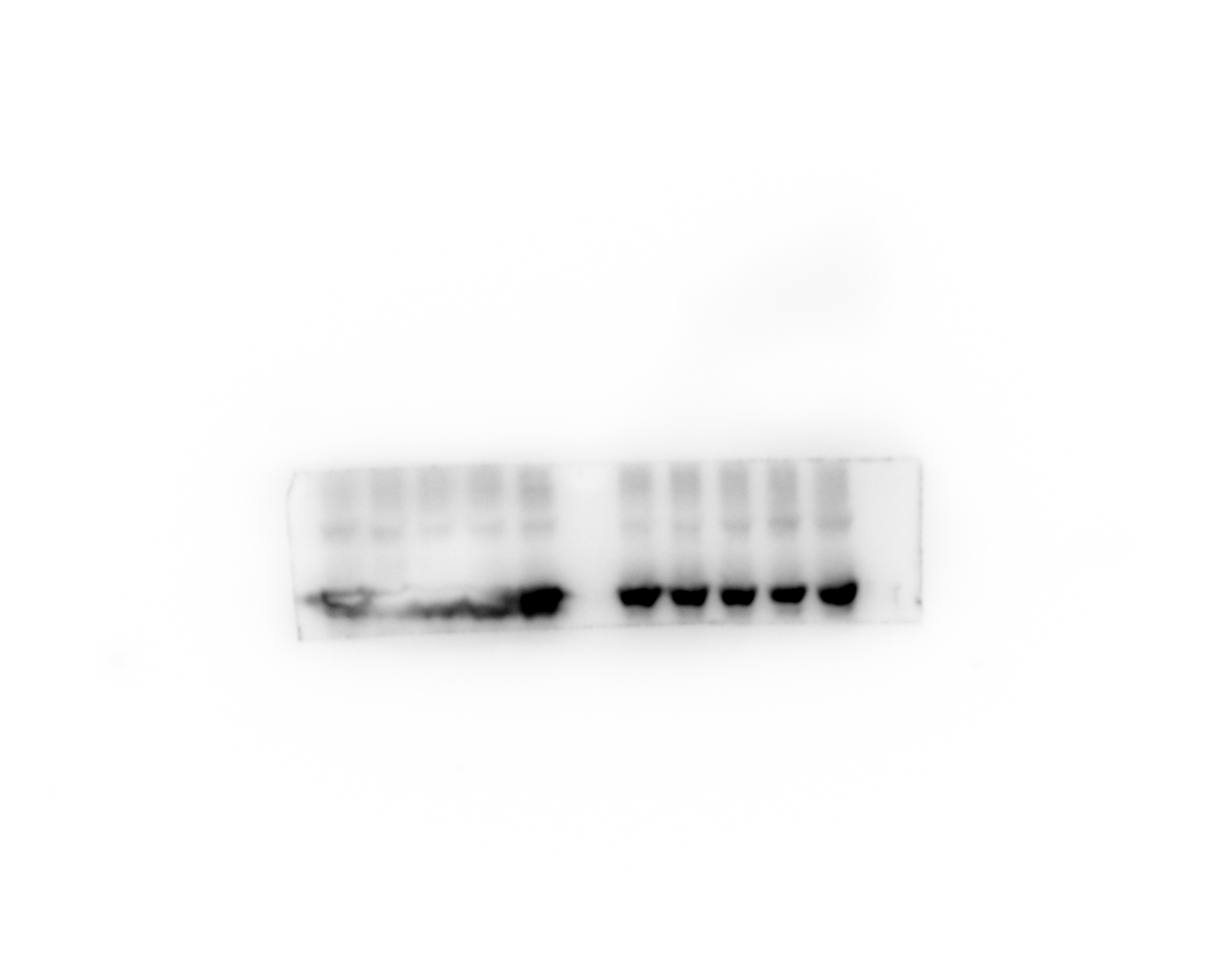
 Figure7 C, left:β-Actin**

**
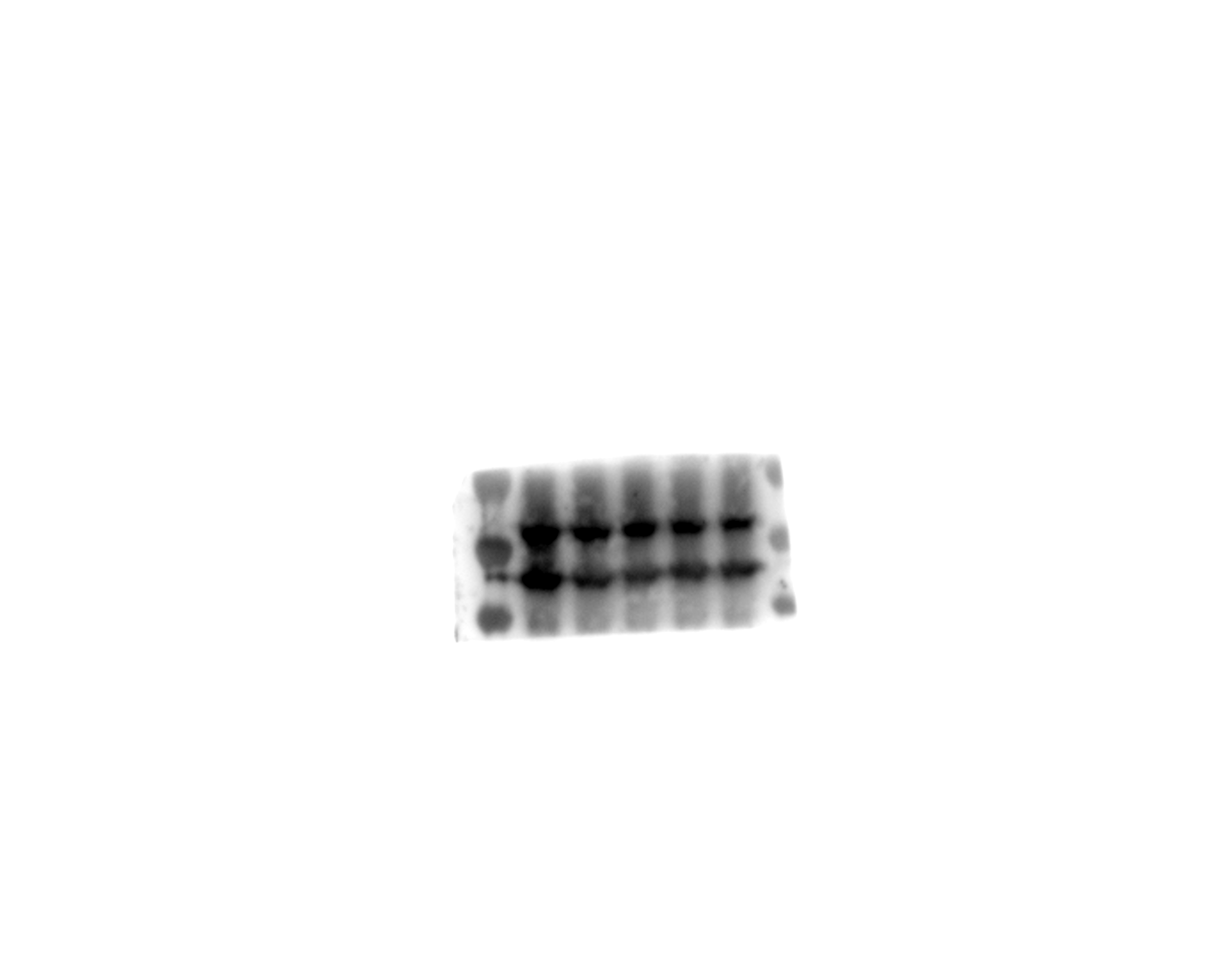
 Figure7 C, right: PD-L1**

**
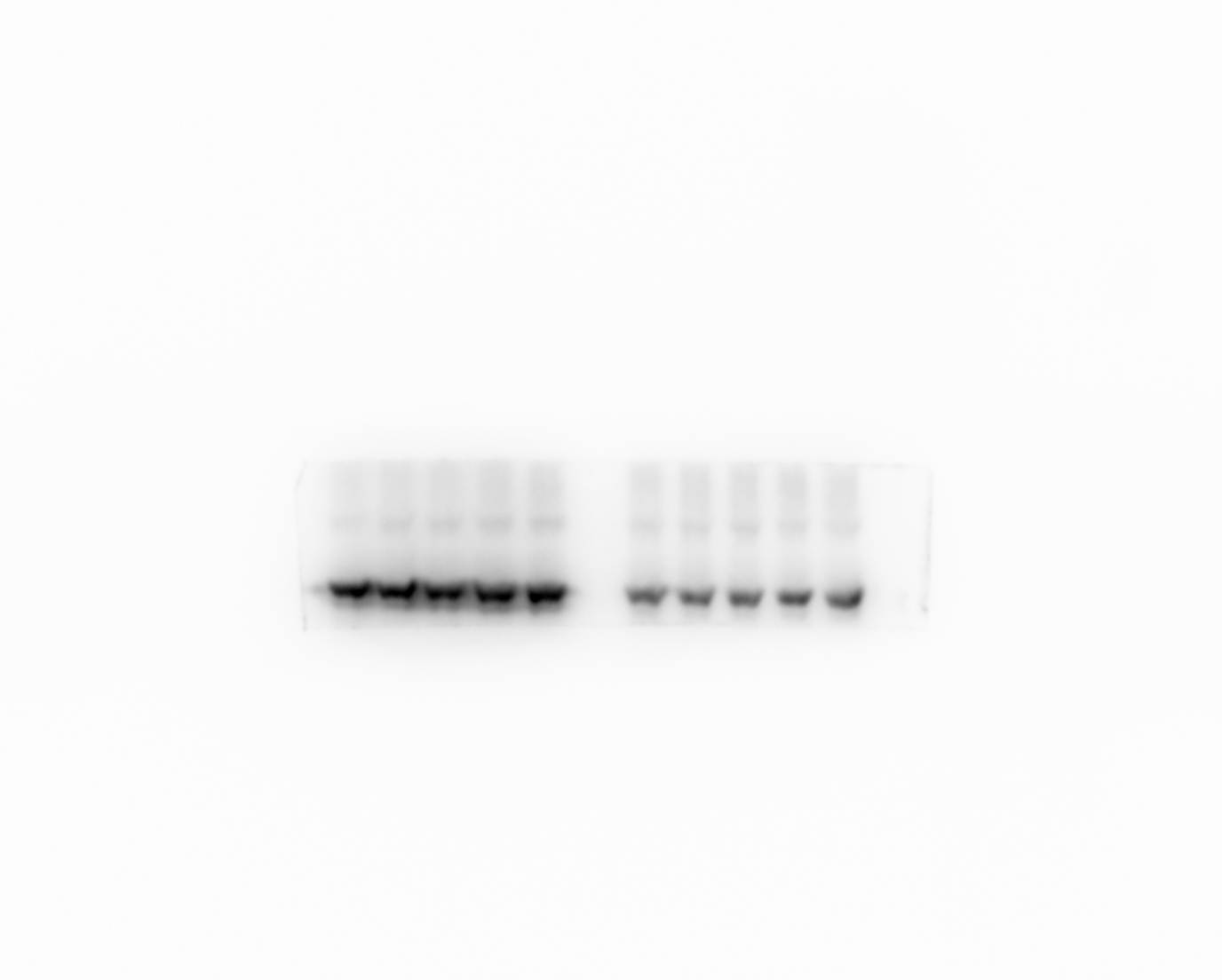
 Figure7 C, right: β-Actin**

**Figure7 D, left: MCUB**

**Group：(1) shNC (2) shMCUB (3) shMCUB+MG132 (4) shMCUB+CQ**

**
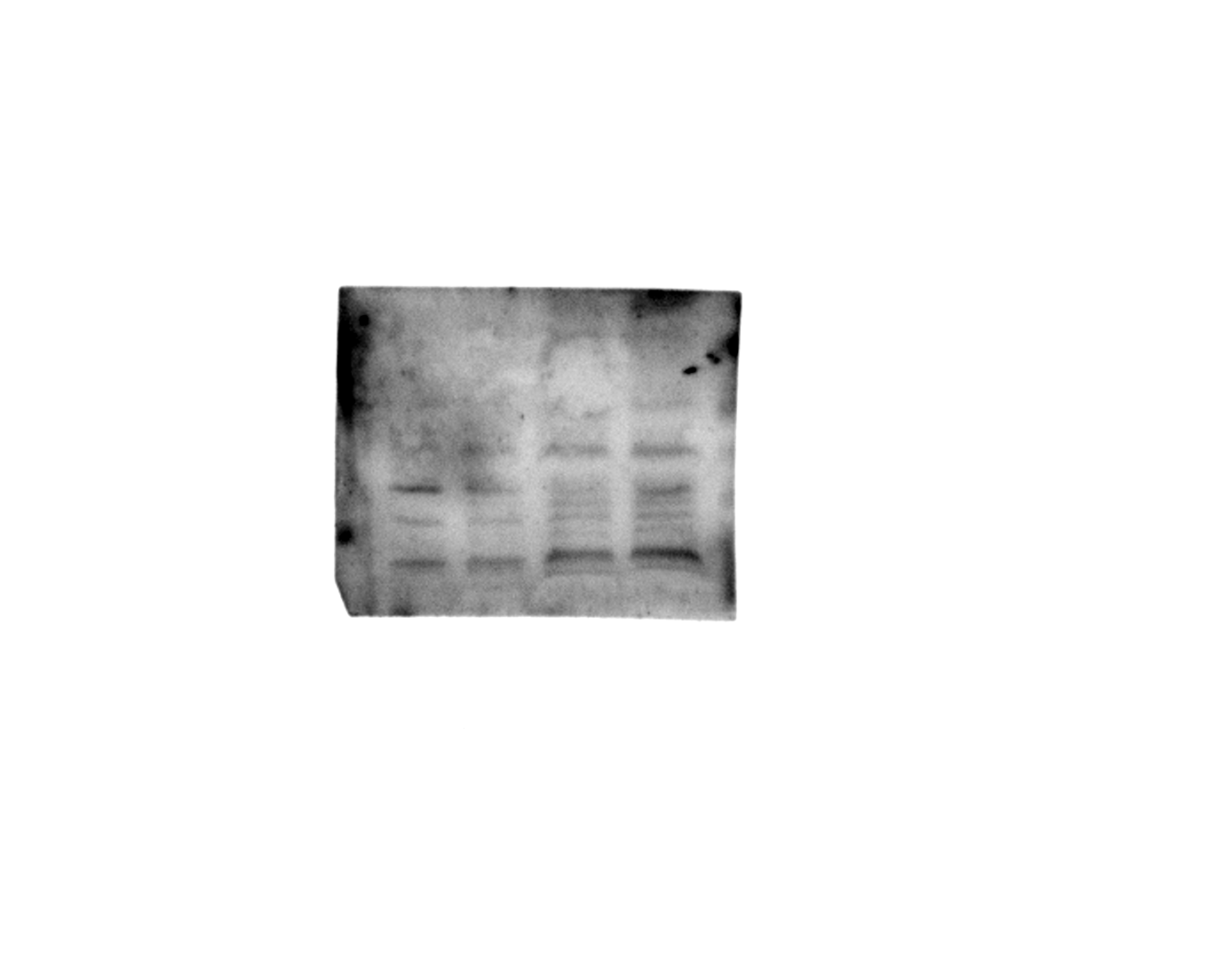
**

**Figure7 D, left: PD-L1**

**Group：(1) shNC (2) shMCUB (3) shMCUB+MG132 (4) shMCUB+CQ**

**
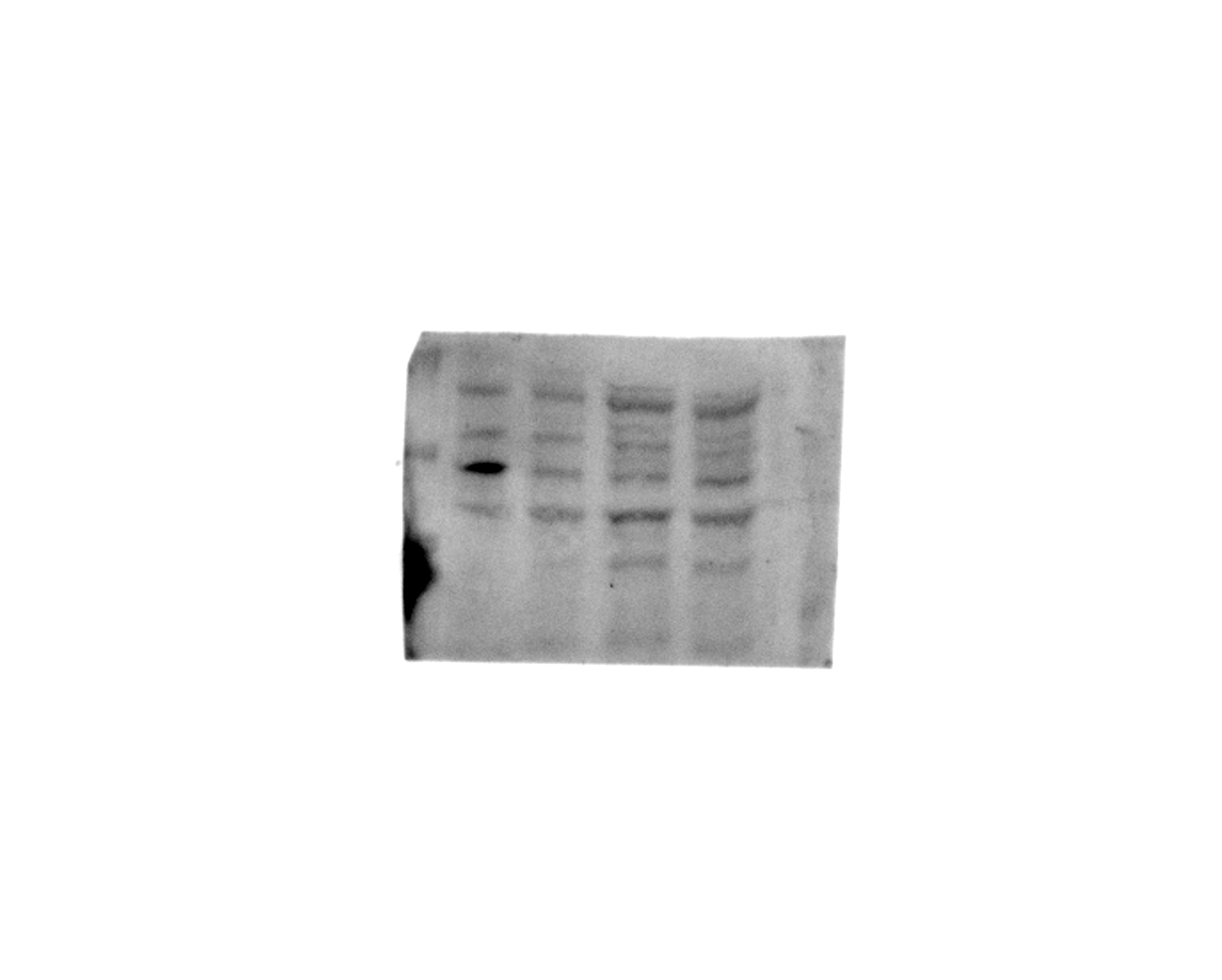
**

**Figure7 D, left: α-Tubulin**

**Group：(1) shNC (2) shMCUB (3) shMCUB+MG132 (4) shMCUB+CQ**

**
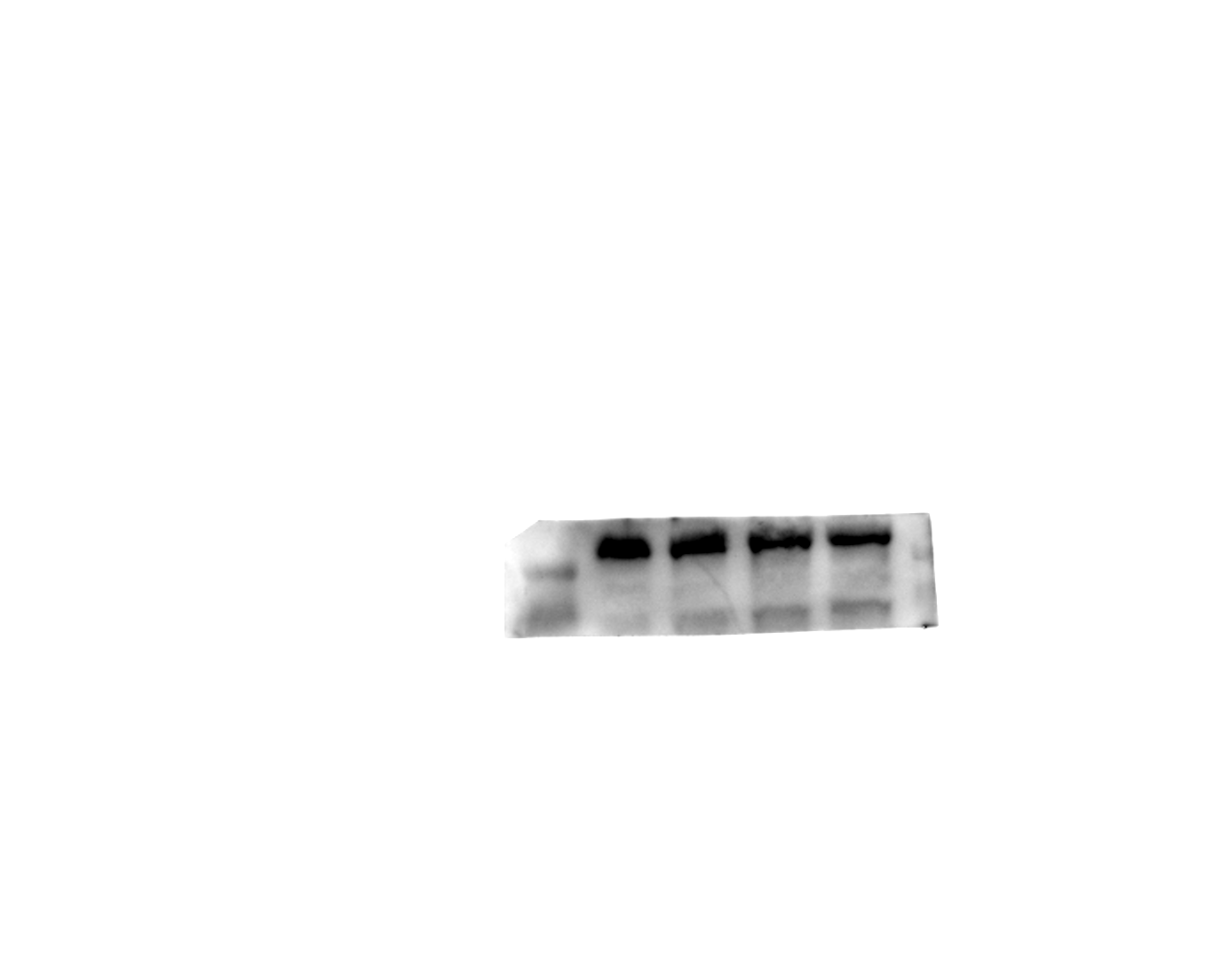
**


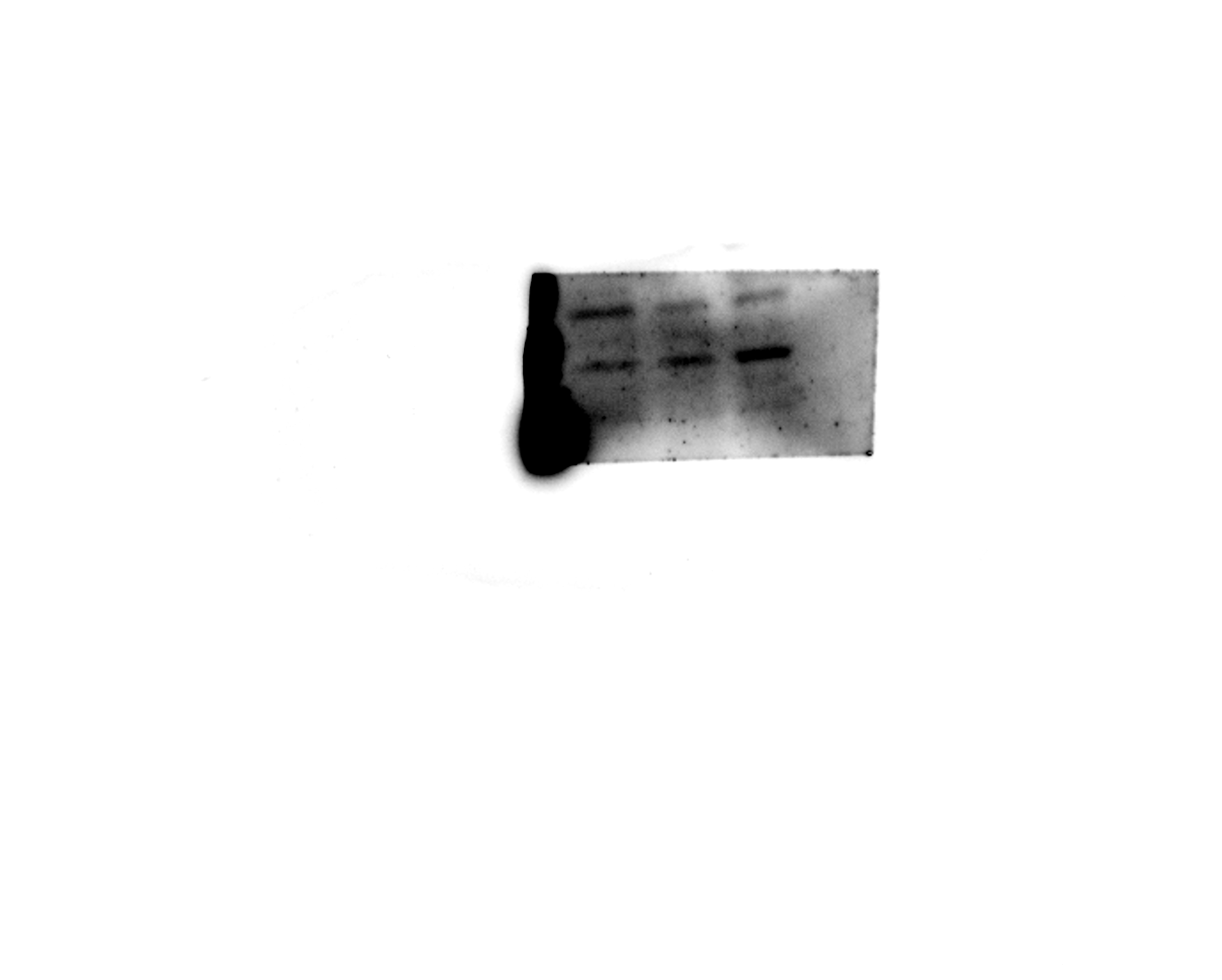
 **Figure7 D, right: MCUB**

**
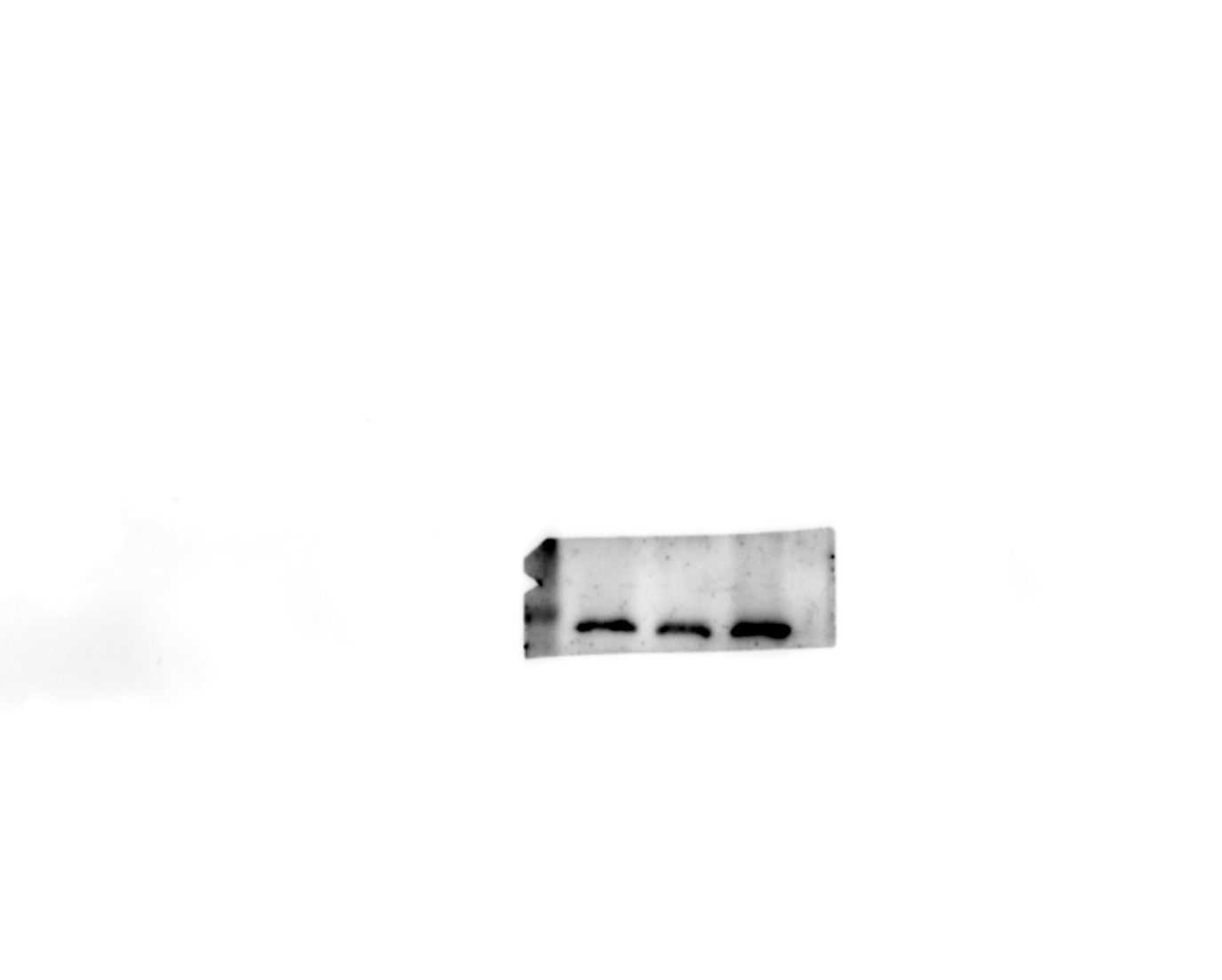
 Figure7 D, right: PD-L1**

**
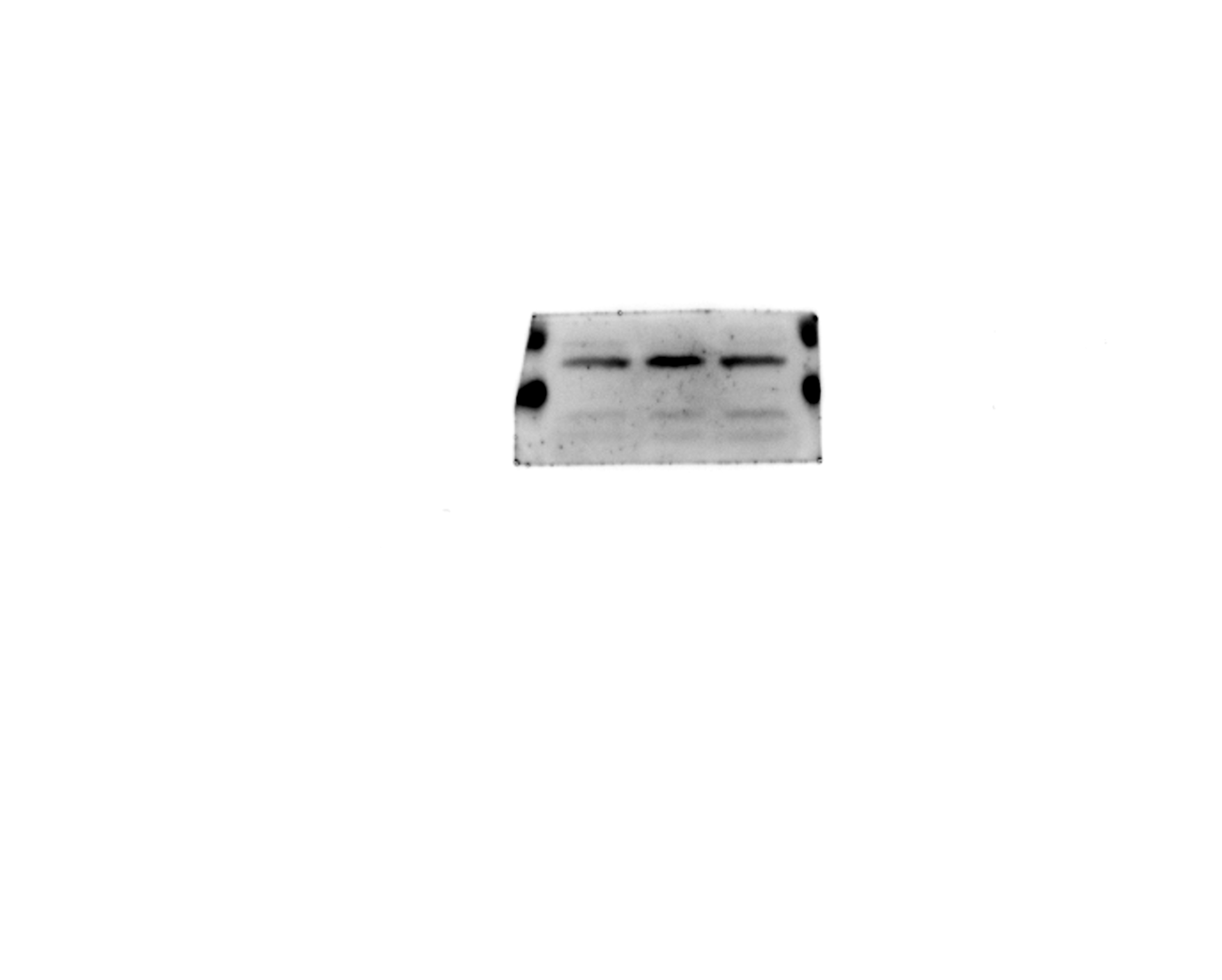
 Figure7 D, right: PRKN**

**
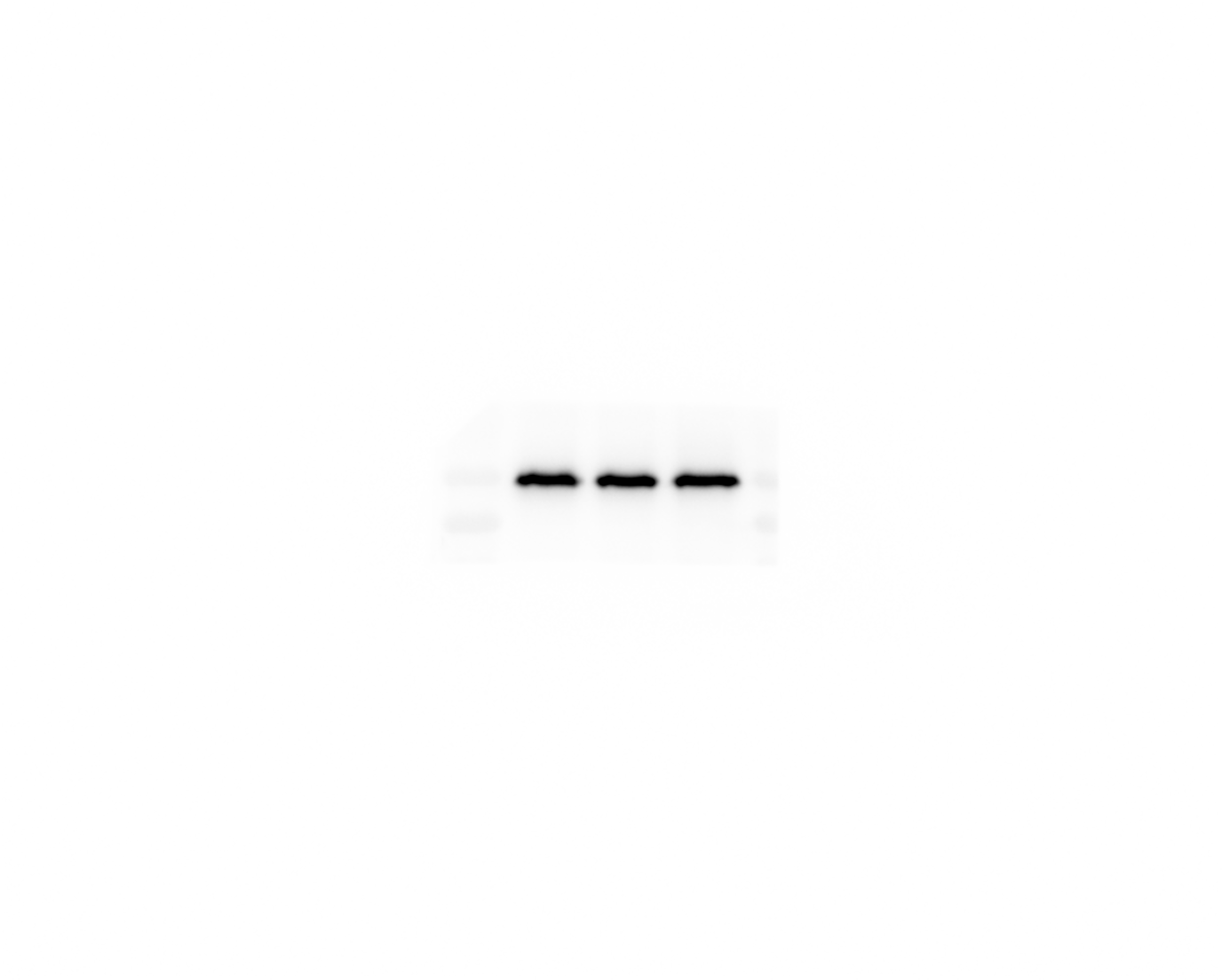
 Figure7 D, right: α-Tubulin**

**
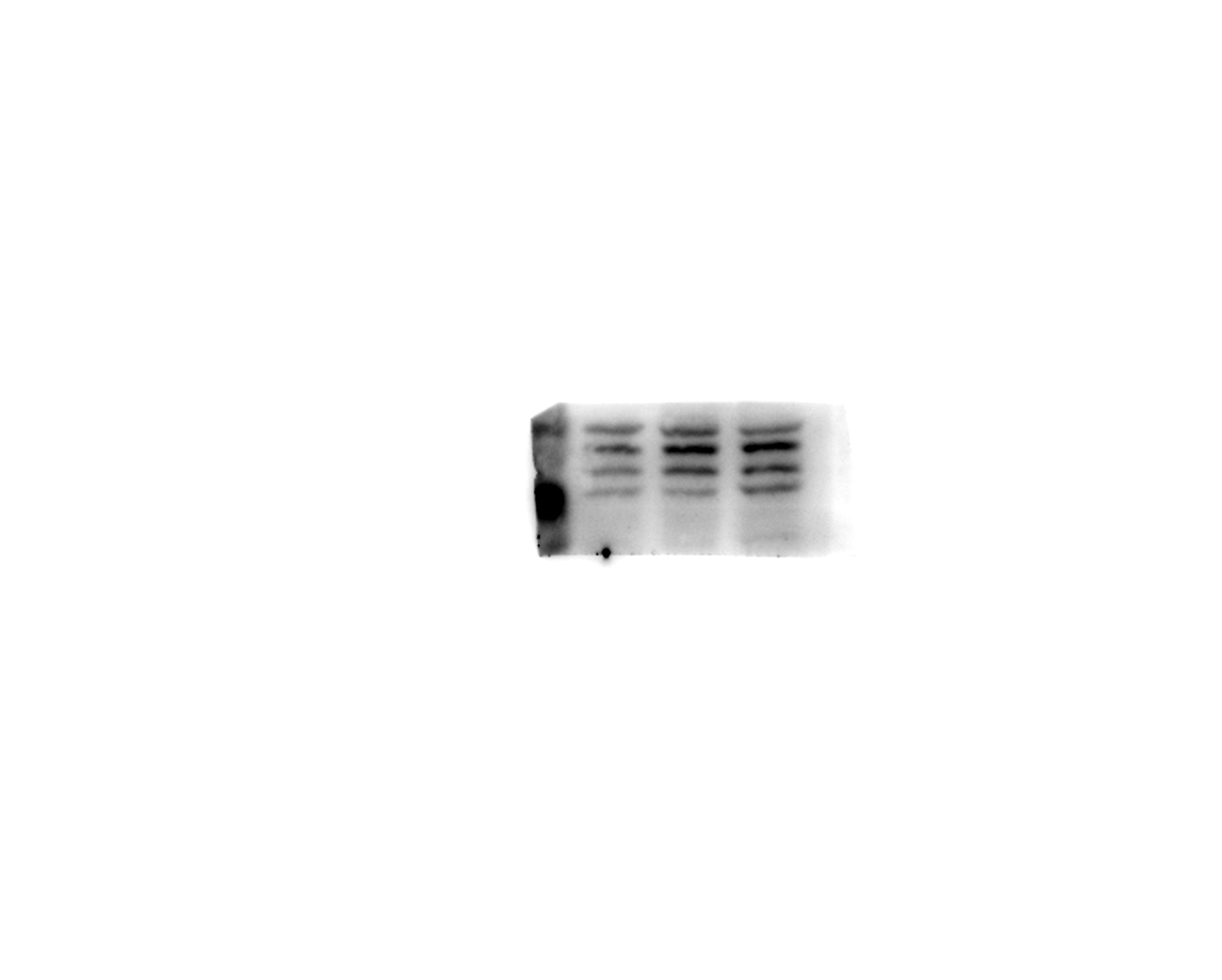
 Figure7 E: MCUB**

**
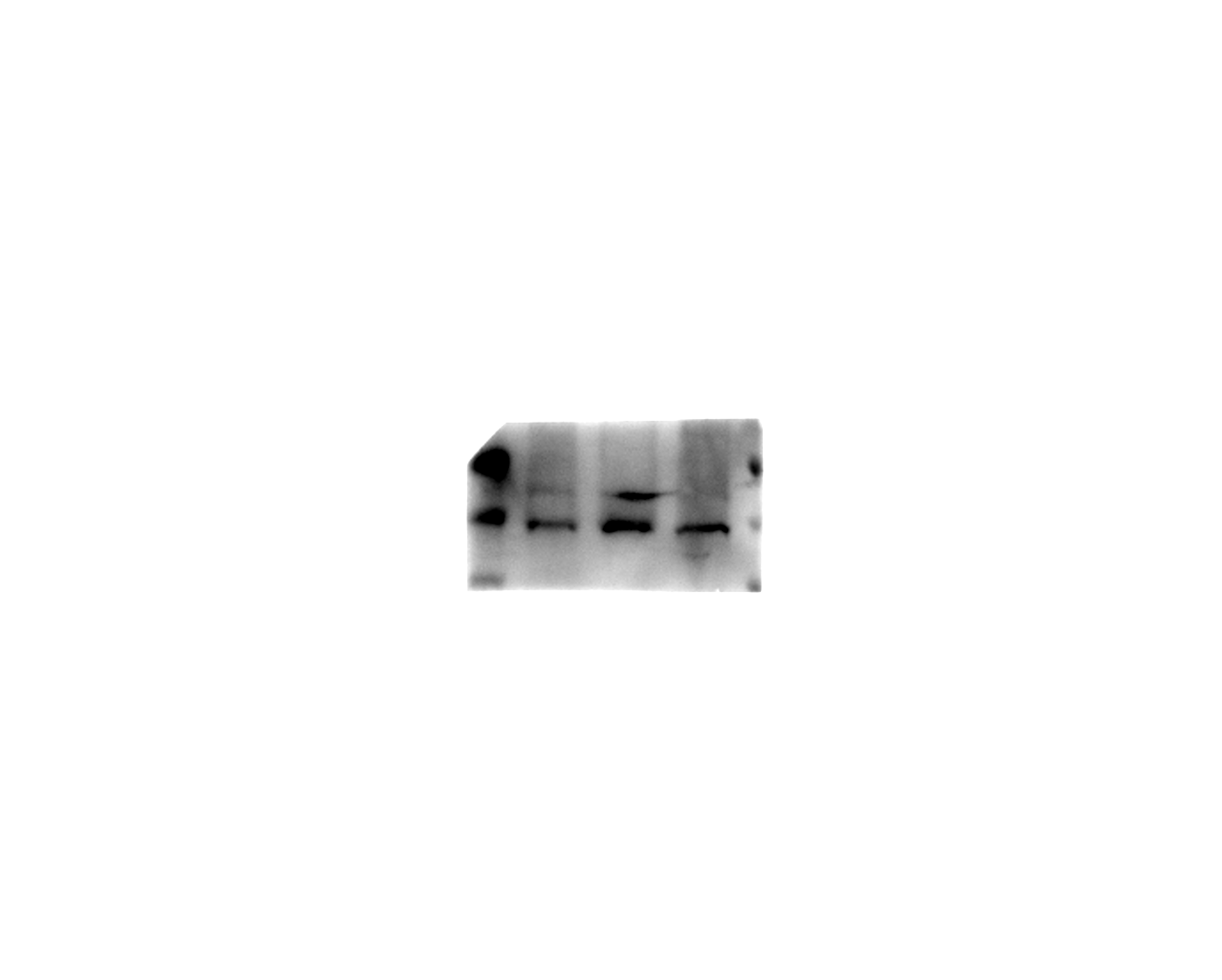
 Figure7 E: PD-L1**

**
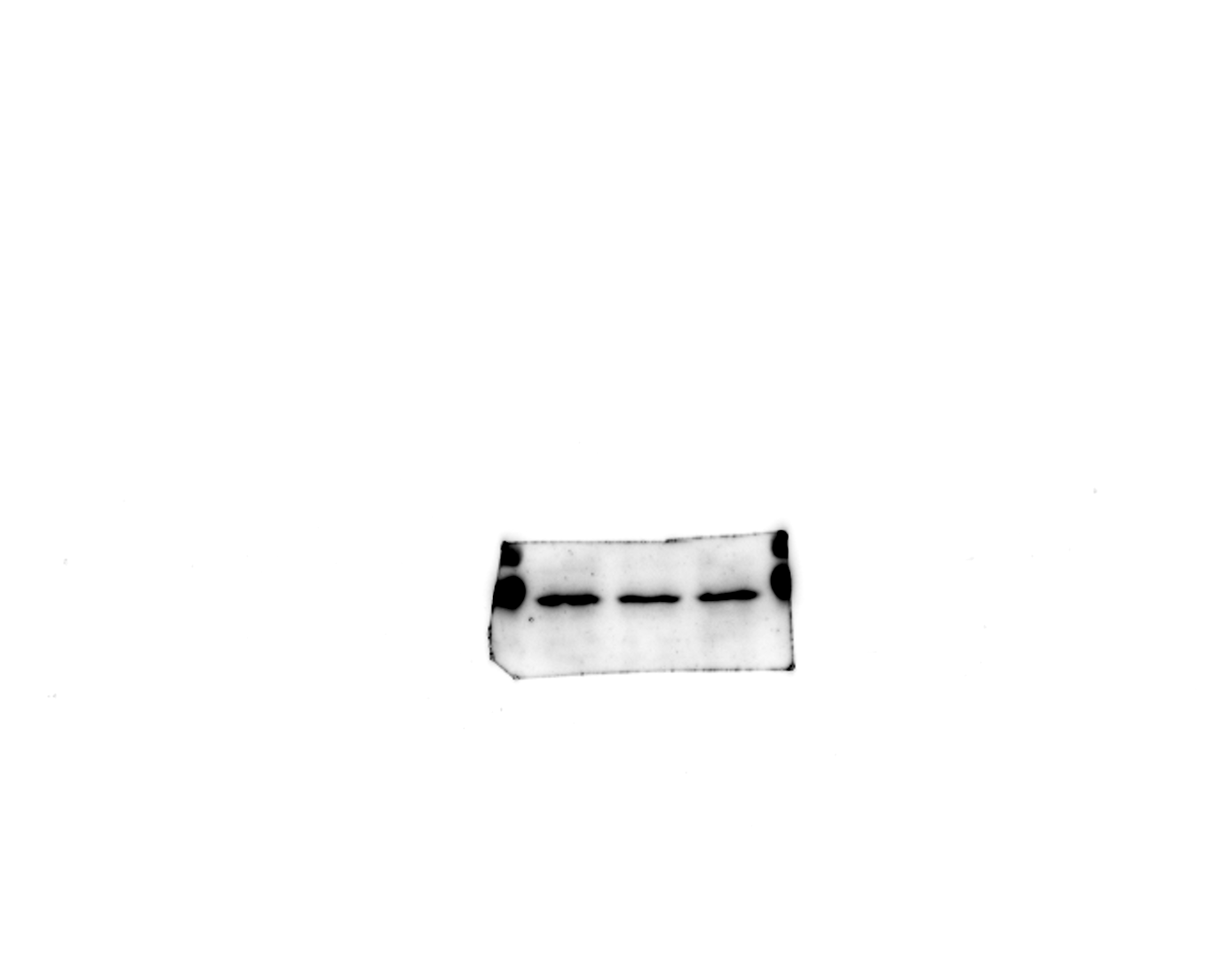
 Figure7 E: PRKN**

**
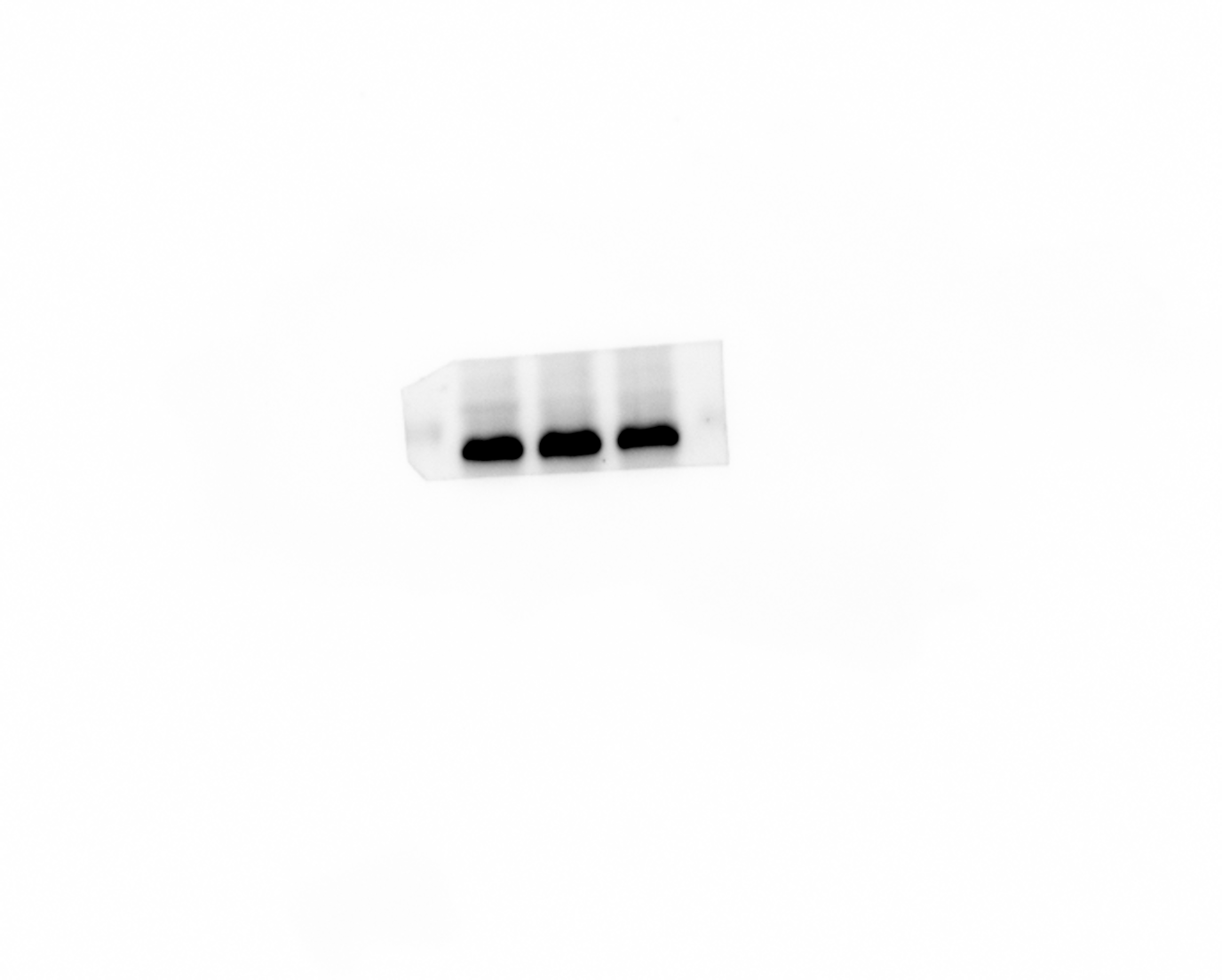
 Figure7 E: α-Tubulin**

**Figure7 F, left:**

**siNC Hoechst**

**
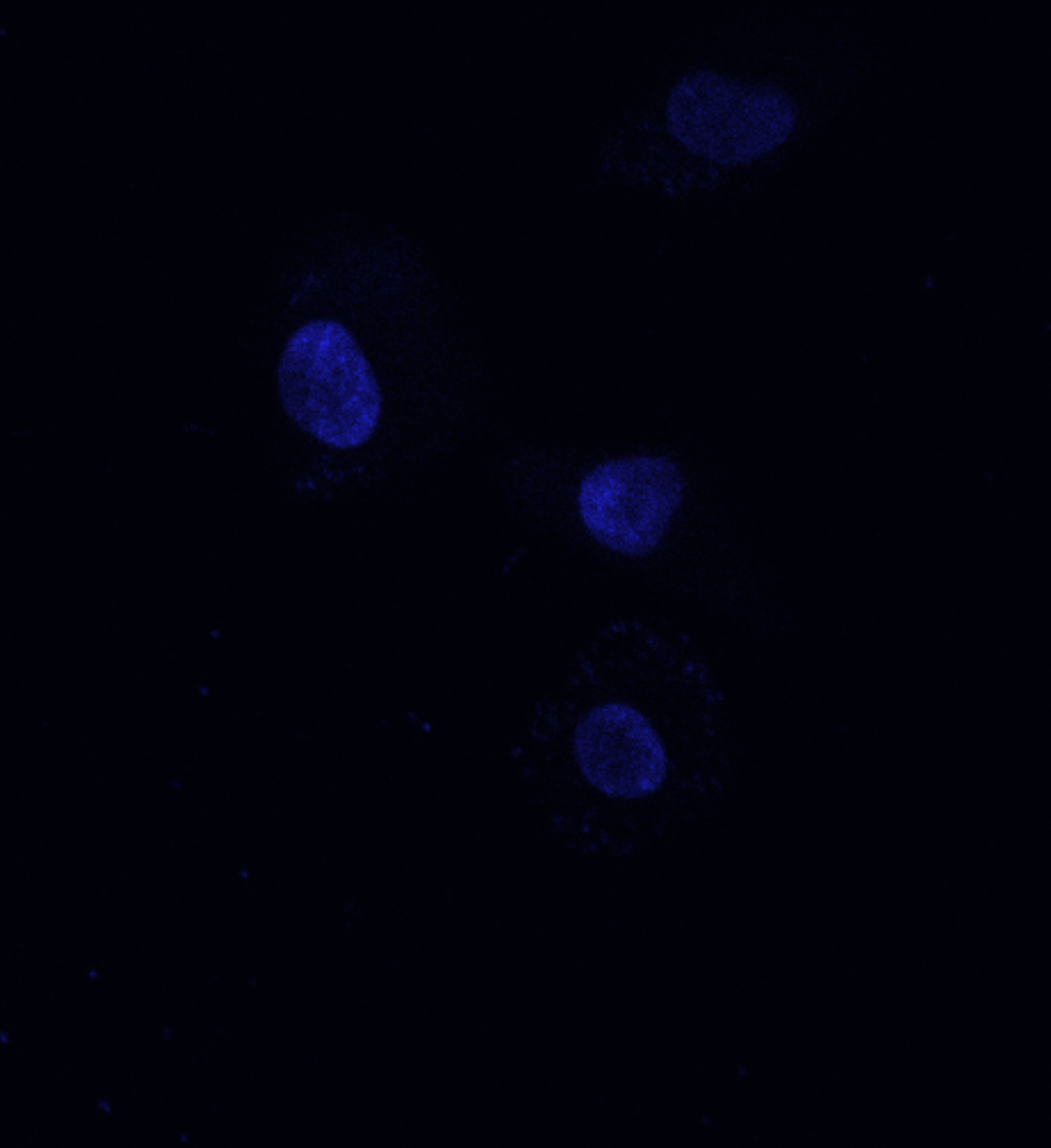
**

**Figure7 F, left:**

**siNC Mitotracker**

**
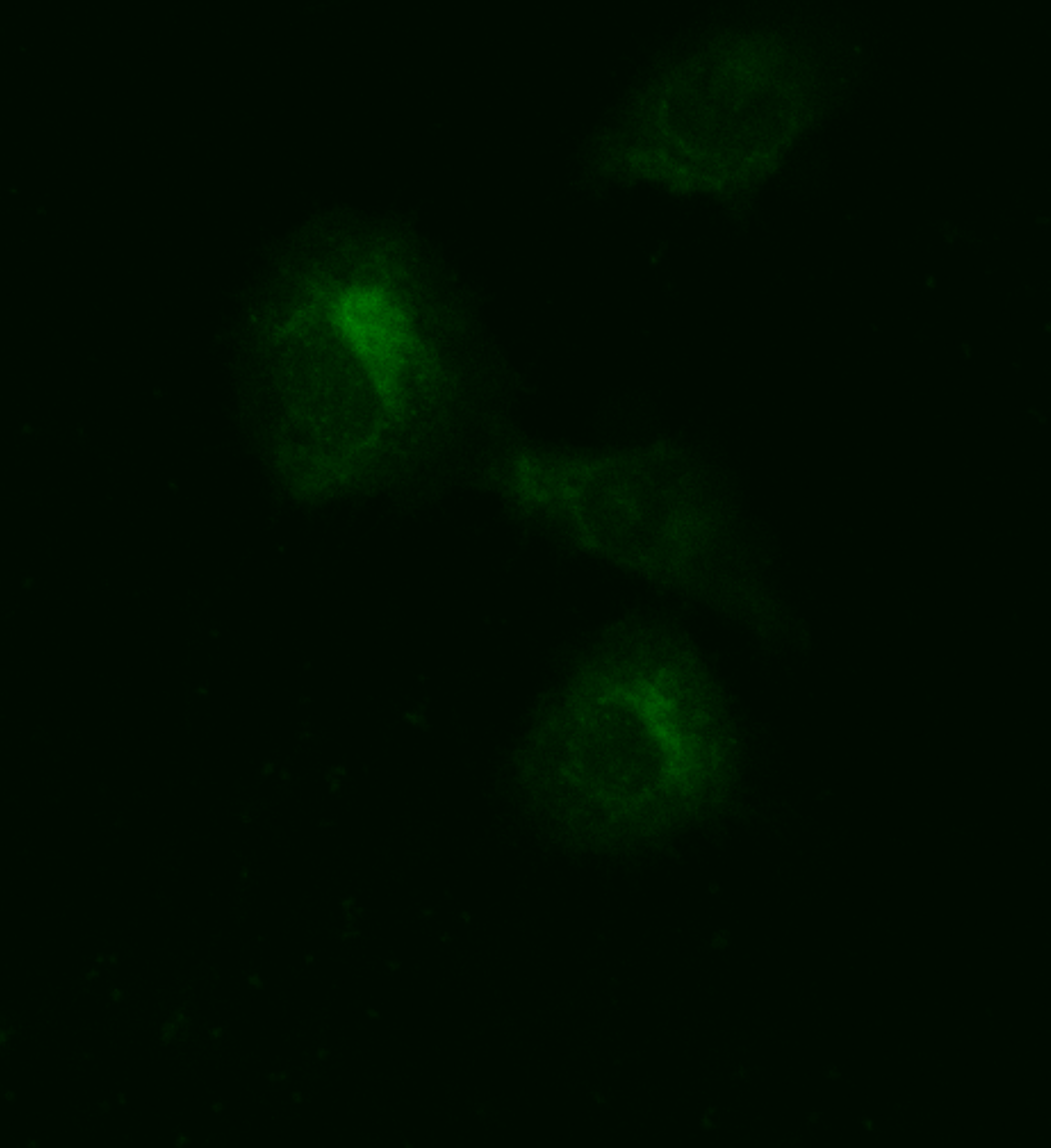
**

**Figure7 F, left:**

**siNC Lysotracker**

**
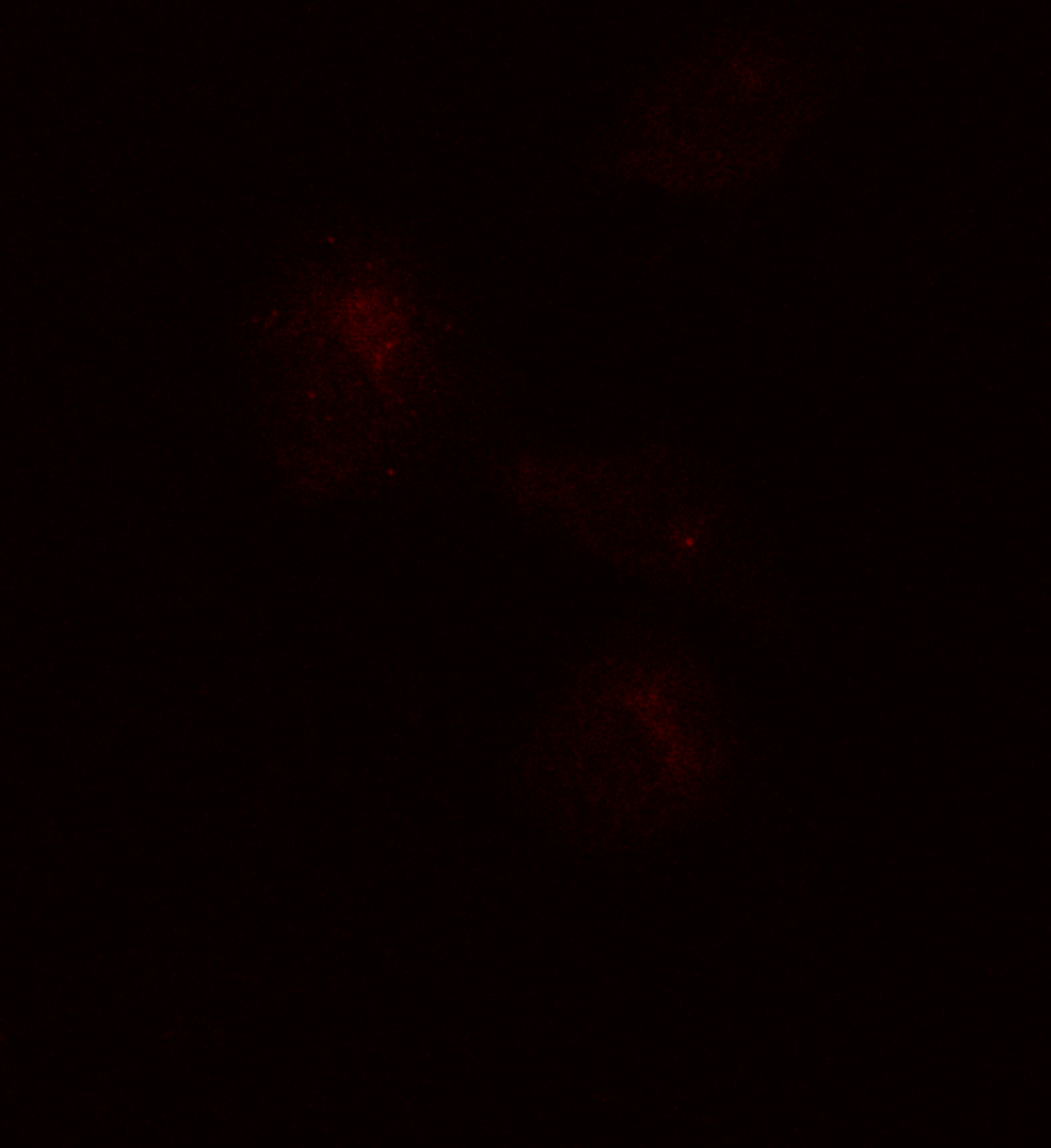
**

**Figure7 F, left:**

**siNC Merge**

**
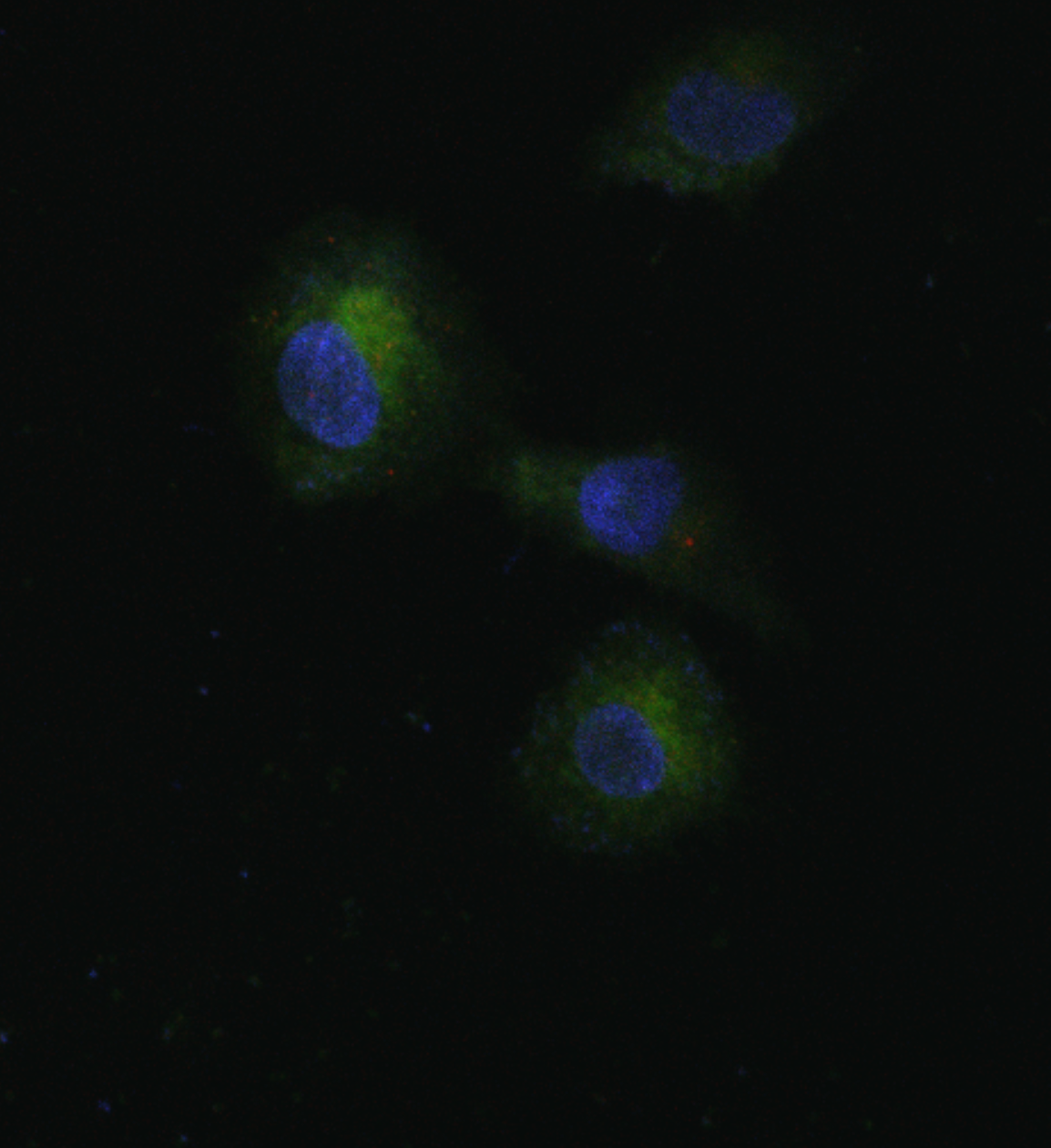
**

**Figure7 F, left:**

**siMCUB#1 Hoechst**

**
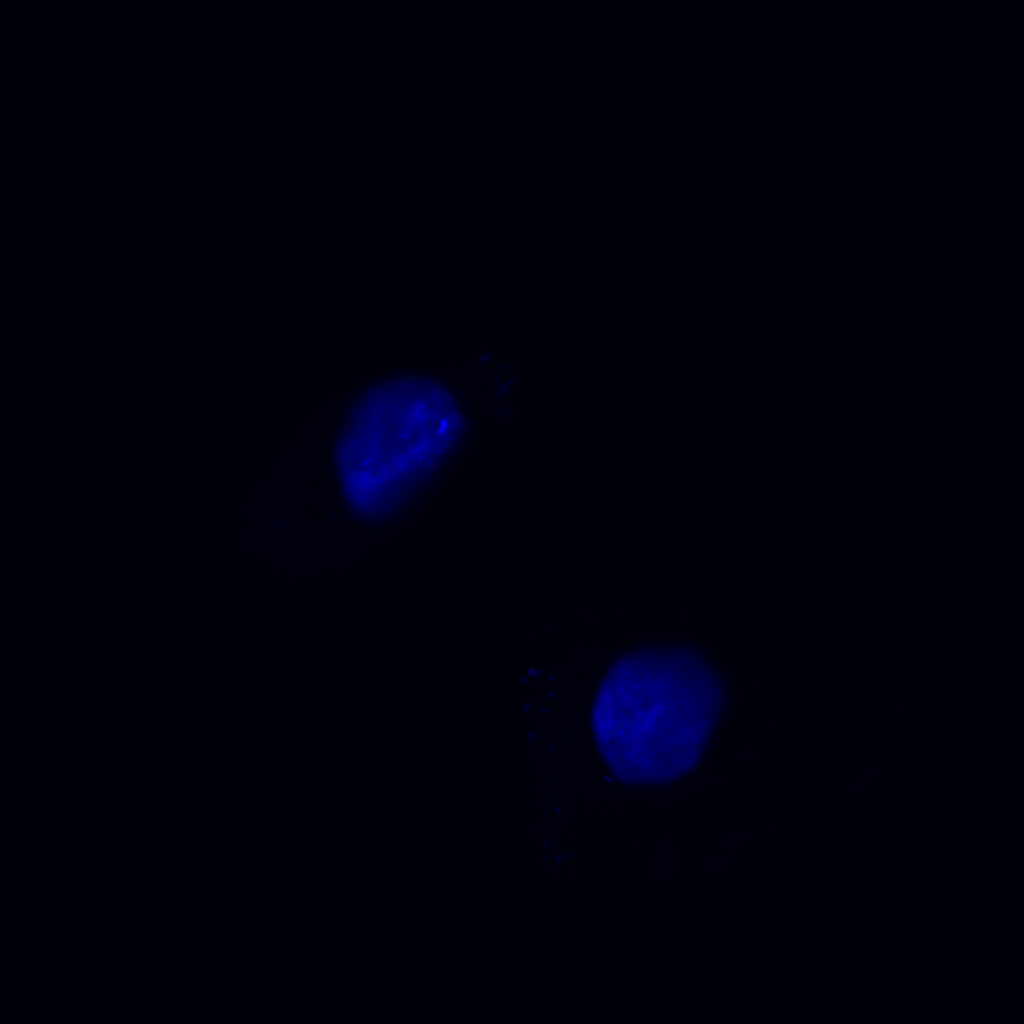
**

**Figure7 F, left:**

**siMCUB#1 Mitotracker**

**
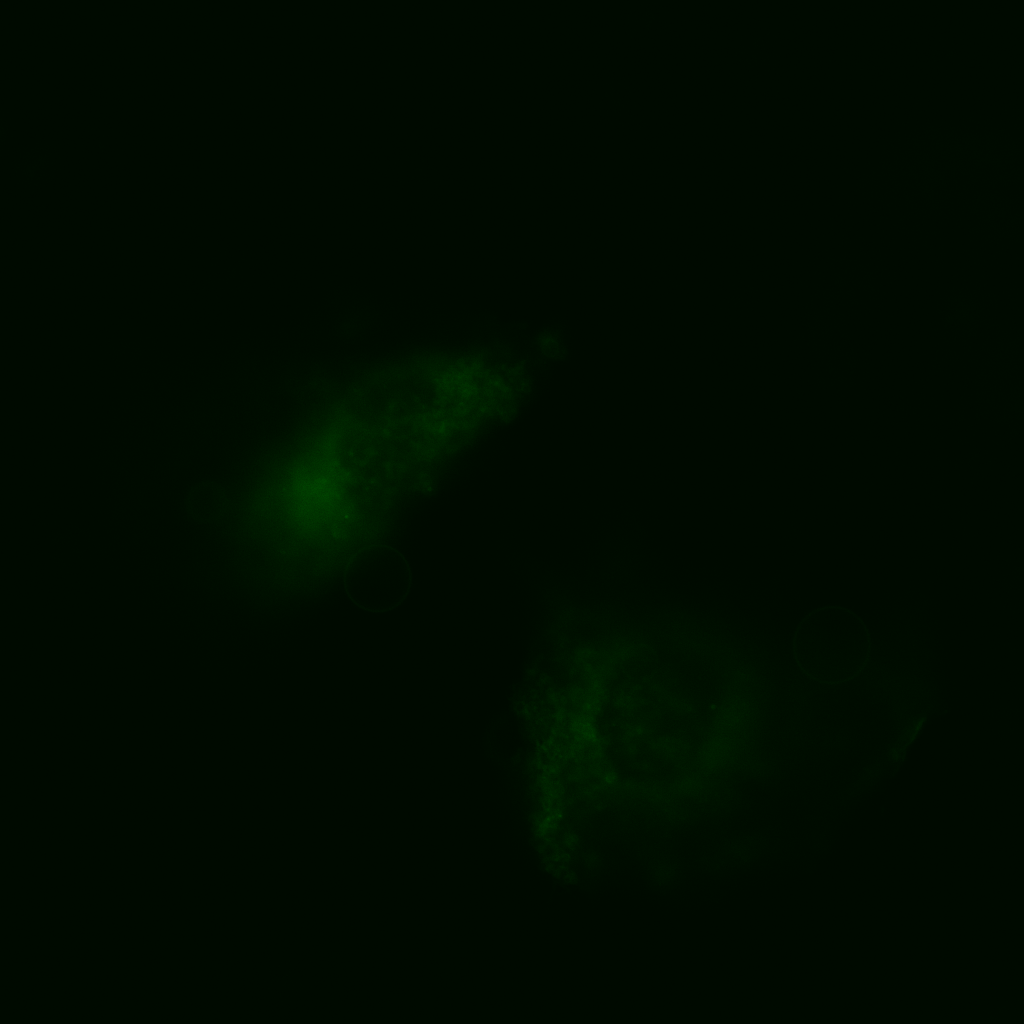
**

**Figure7 F, left:**

**siMCUB#1 Lysotracker**

**
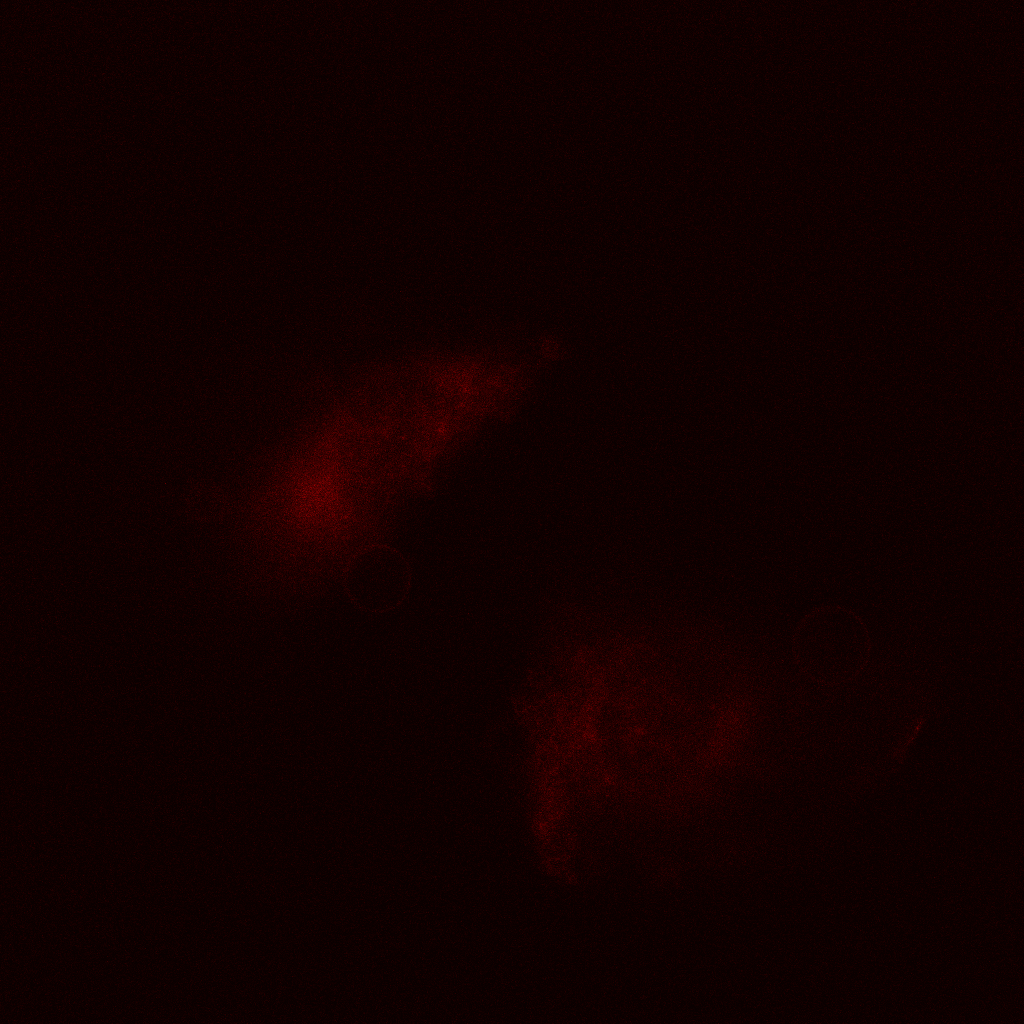
**

**Figure7 F, left:**

**siMCUB#1 Merge**

**
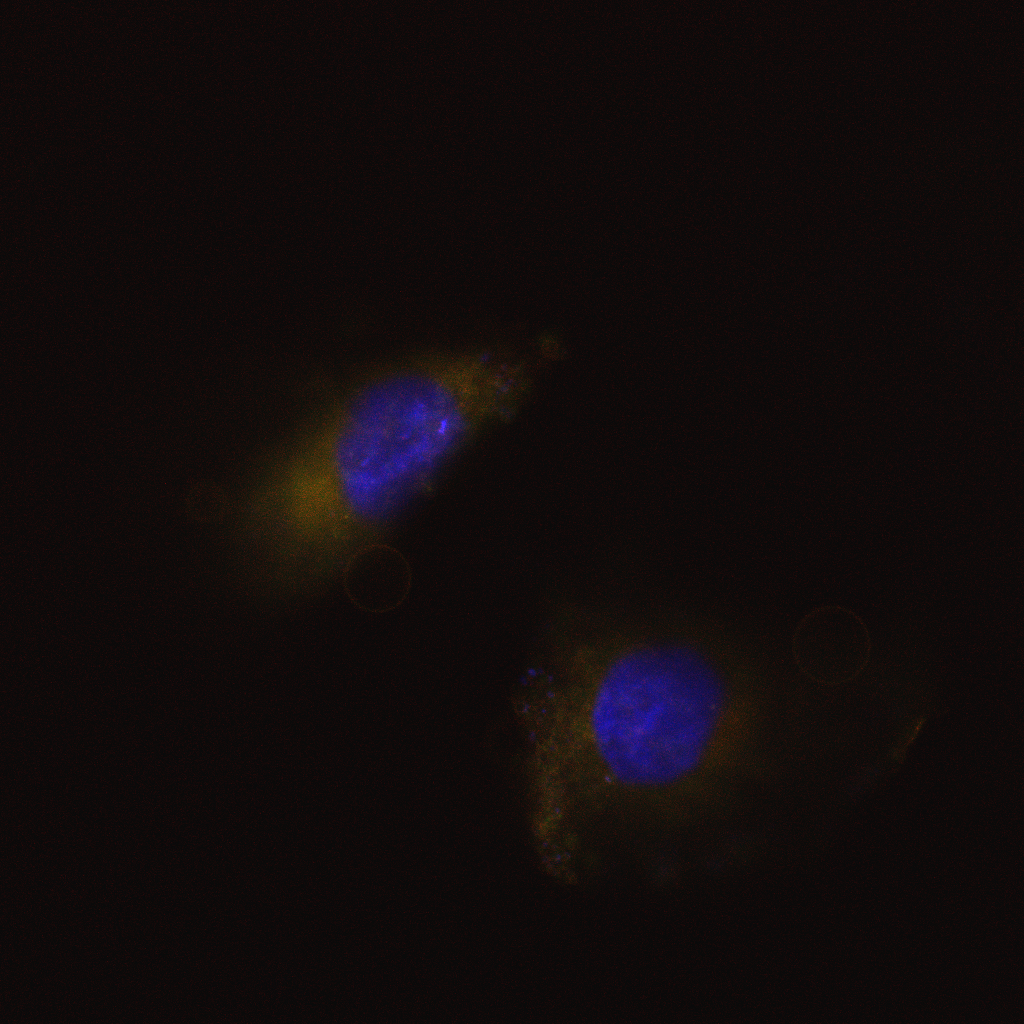
**

**Figure7 F, left:**

**siMCUB#2 Hoechst**

**
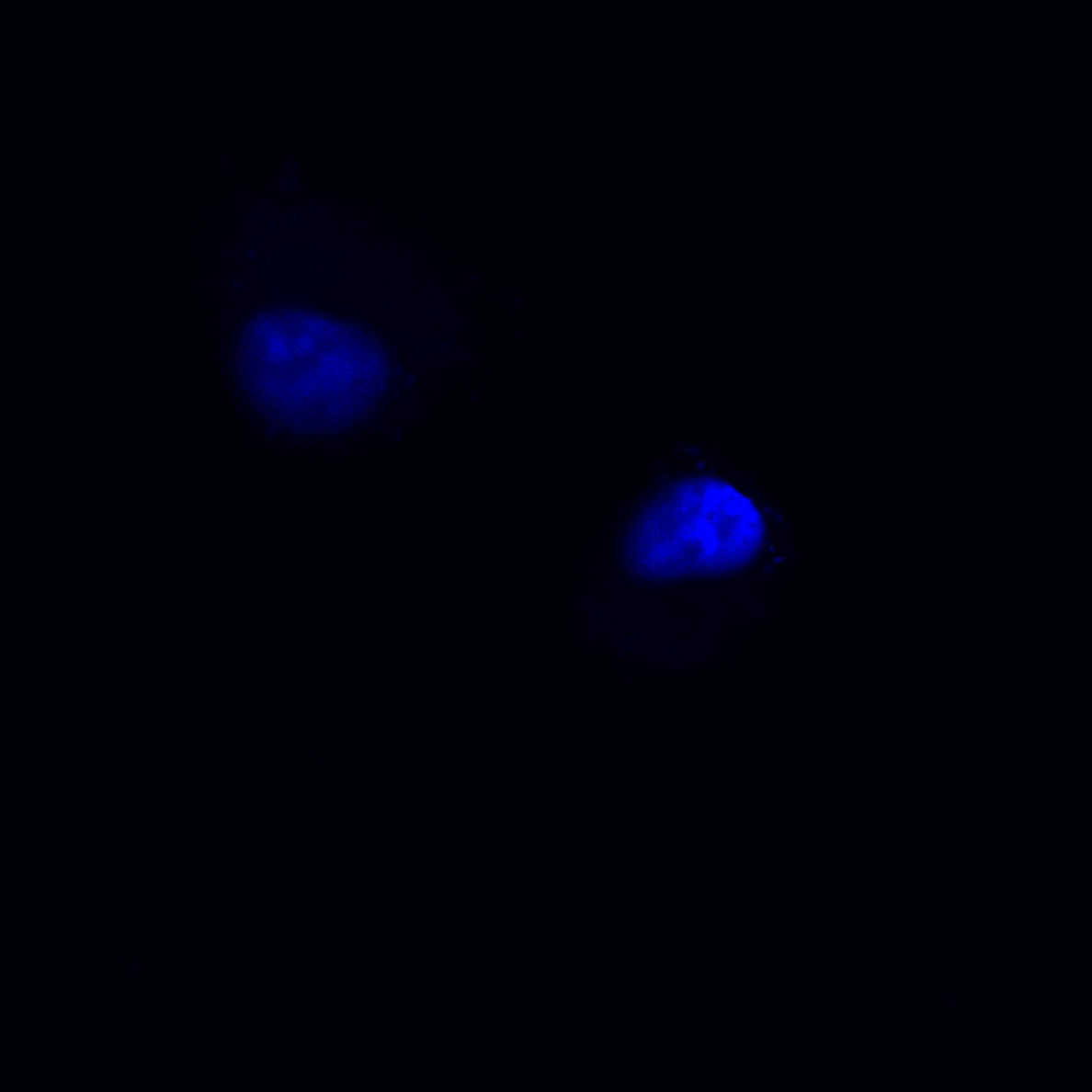
**

**Figure7 F, left:**

**siMCUB#2 Mitotracker**

**
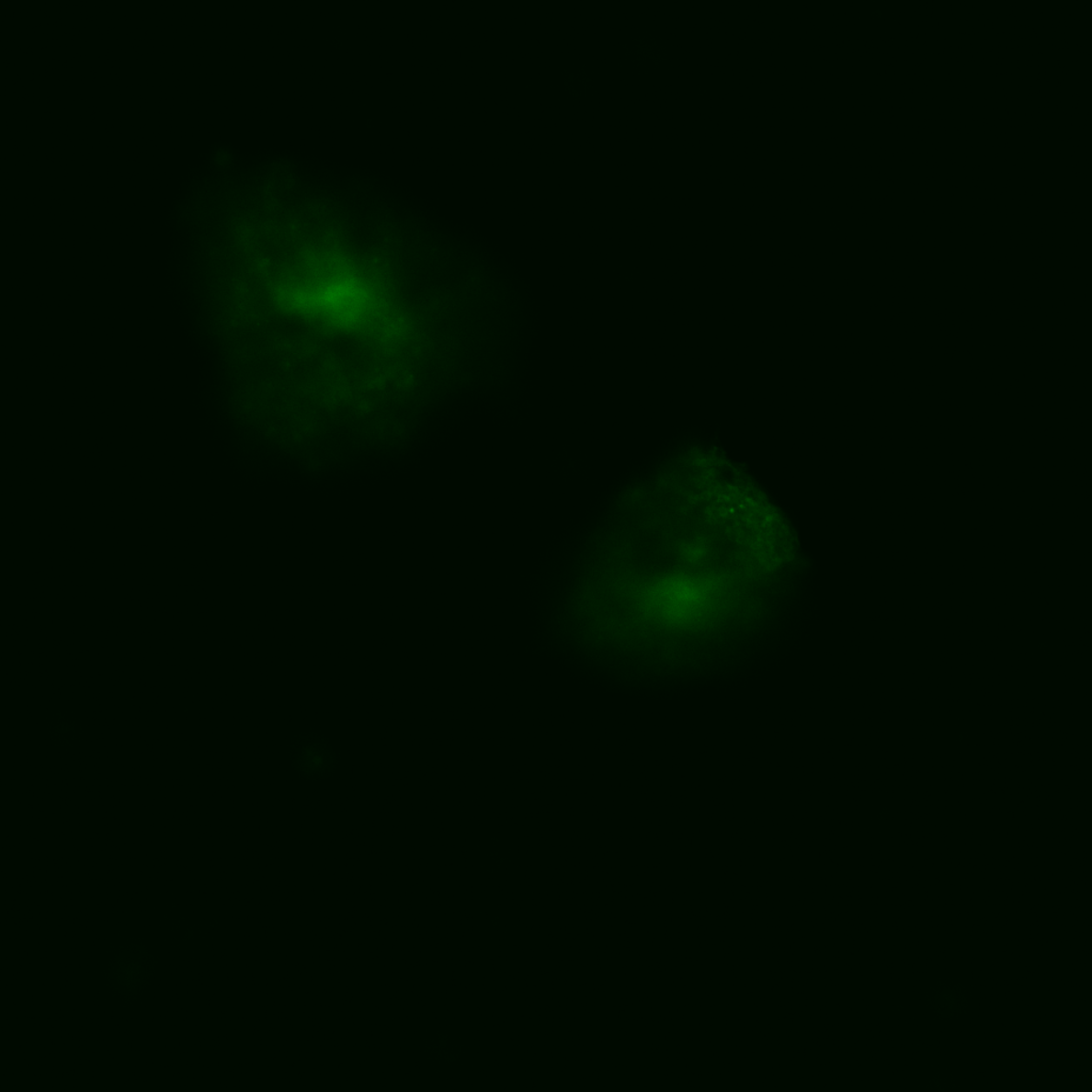
**

**Figure7 F, left:**

**siMCUB#2 Lysotracker**

**
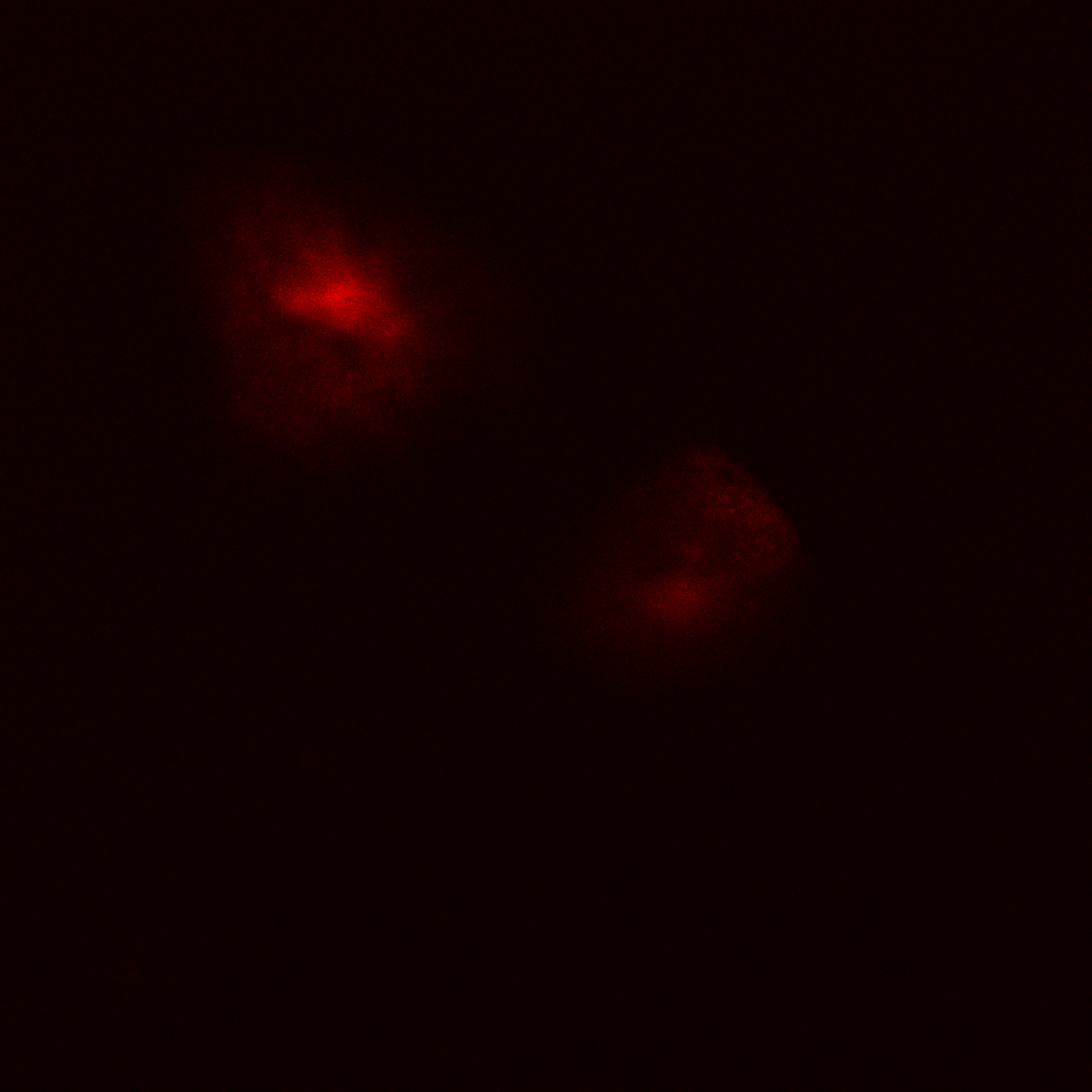
**

**Figure7 F, left:**

**siMCUB#2 Merge**

**
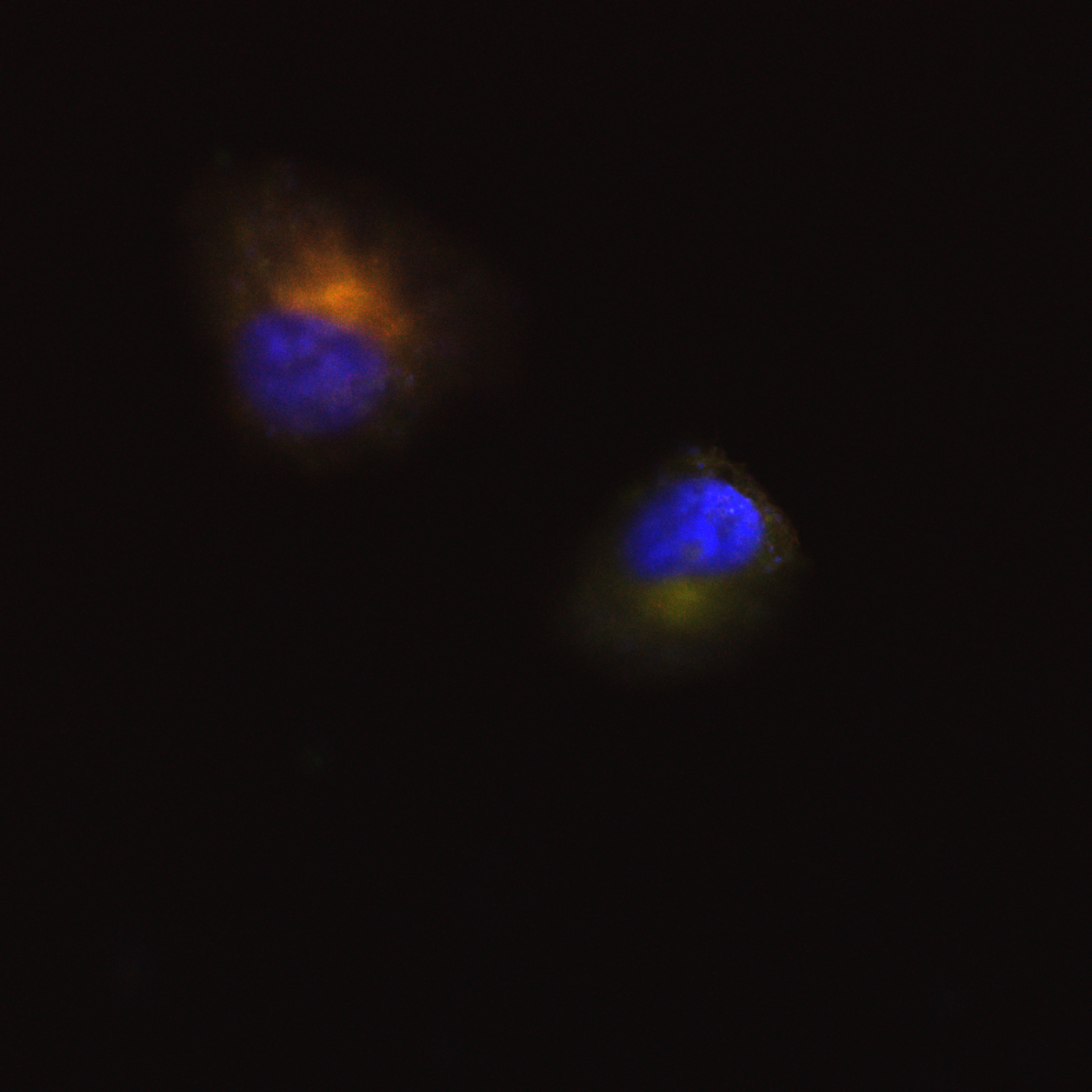
**

**Figure7 F, right:**

**siNC Mitotracker**

**
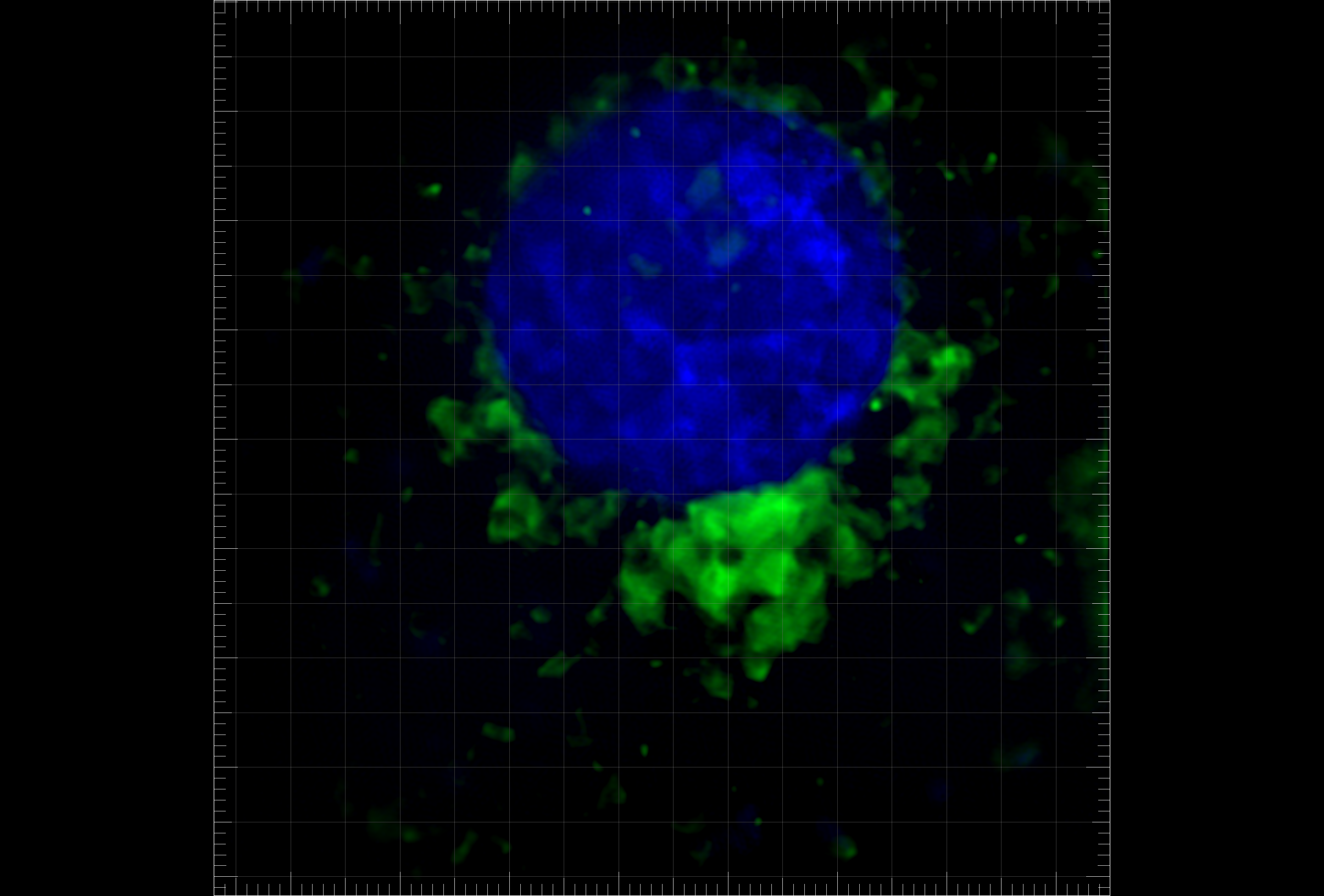
**

**Figure7 F, right:**

**siNC Lysotracker**

**
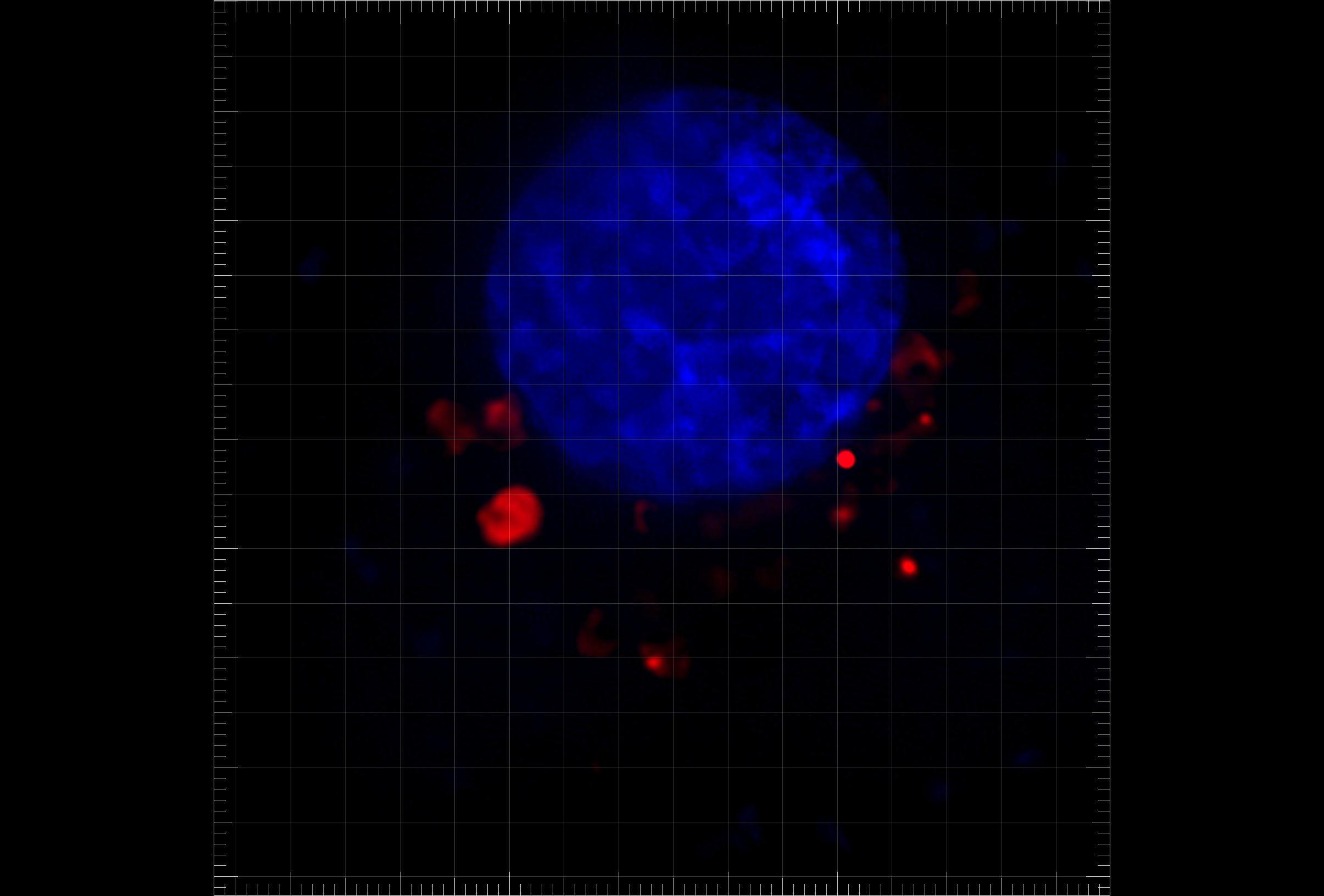
**

**Figure7 F, right:**

**siNC Merge**

**
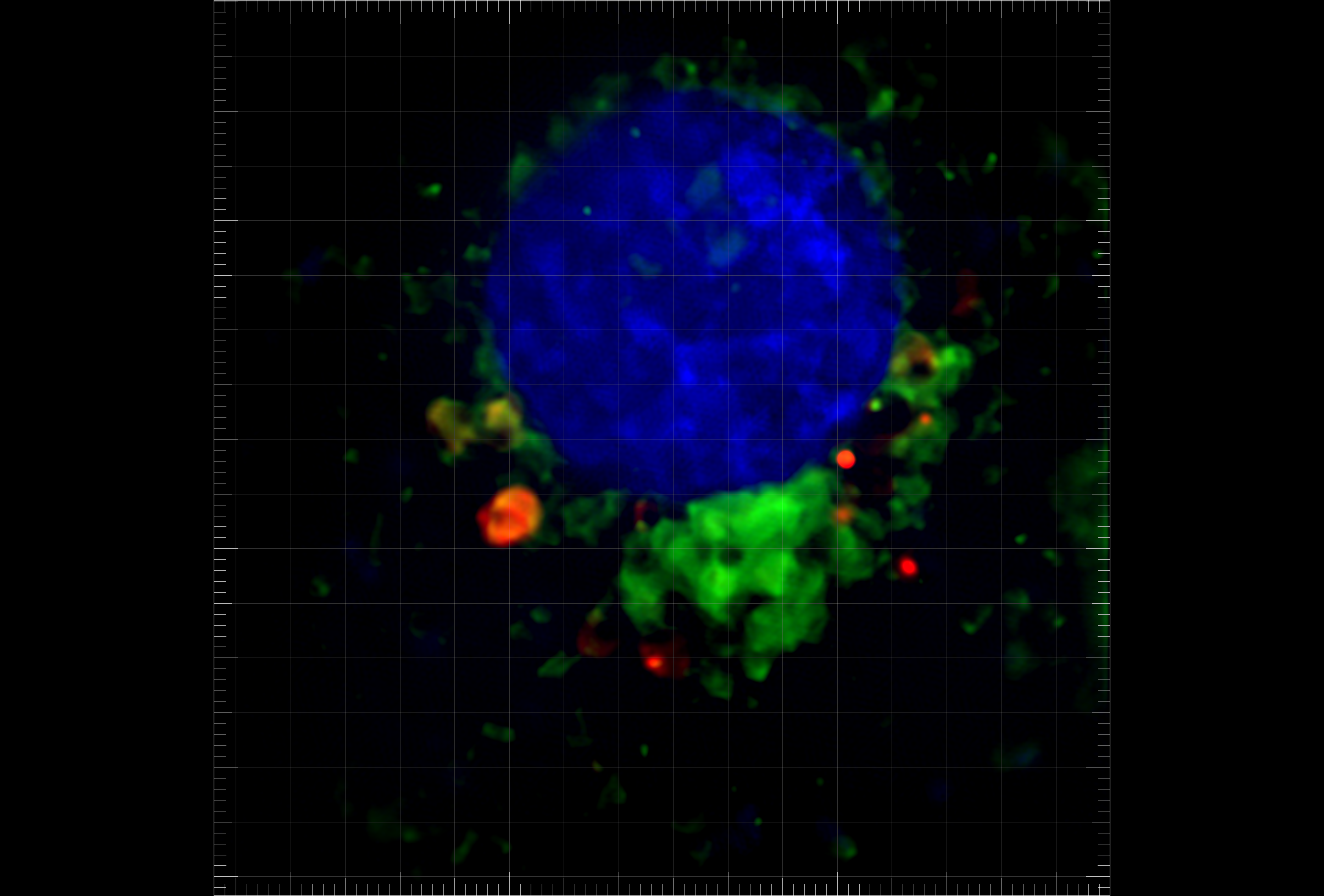
**

**Figure7 F, right:**

**siNC Enlarge**

**
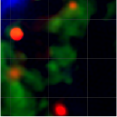
**

**Figure7 F, right:**

**siMCUB#1 Mitotracker**

**
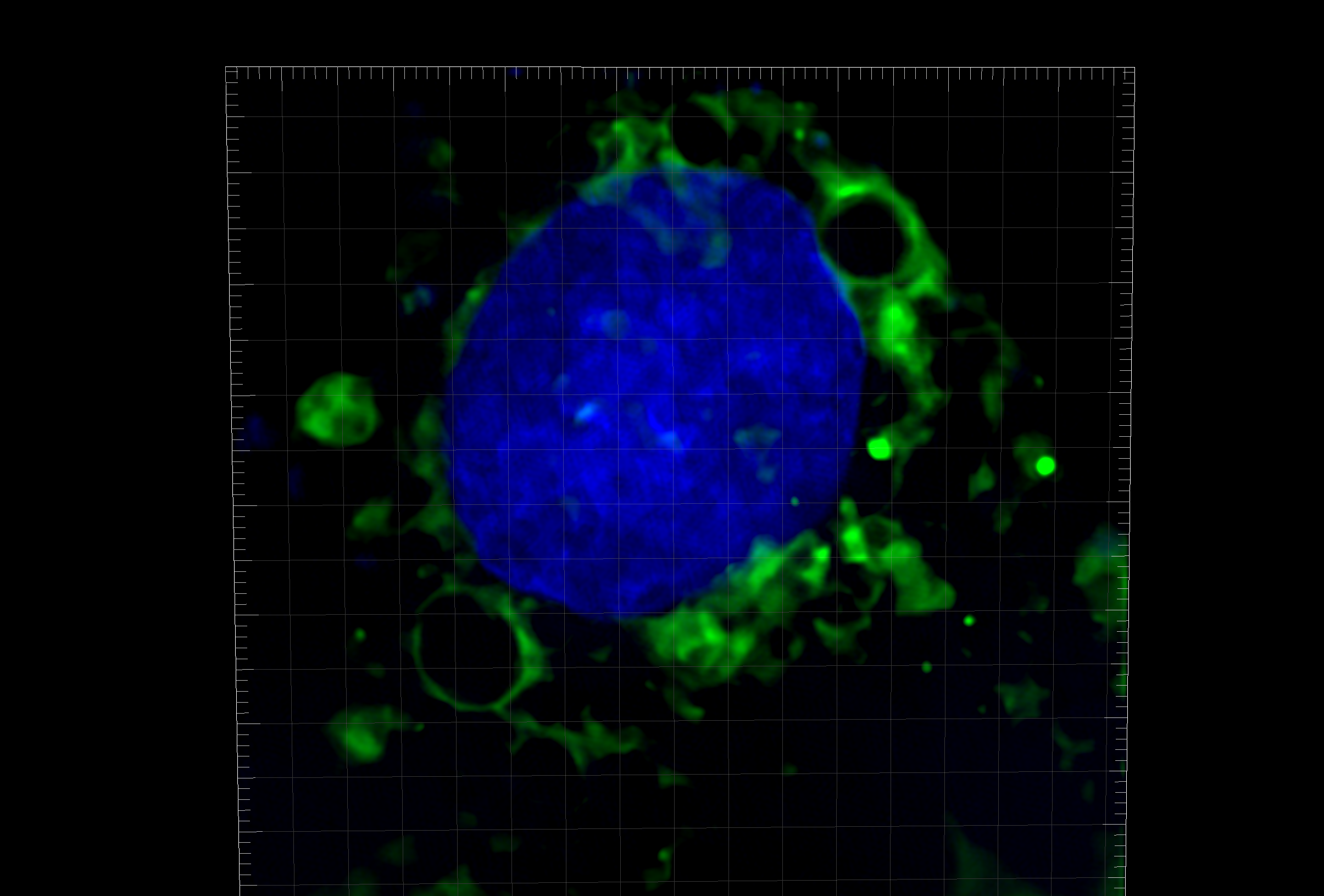
**

**Figure7 F, right:**

**siMCUB#1 Lysotracker**

**
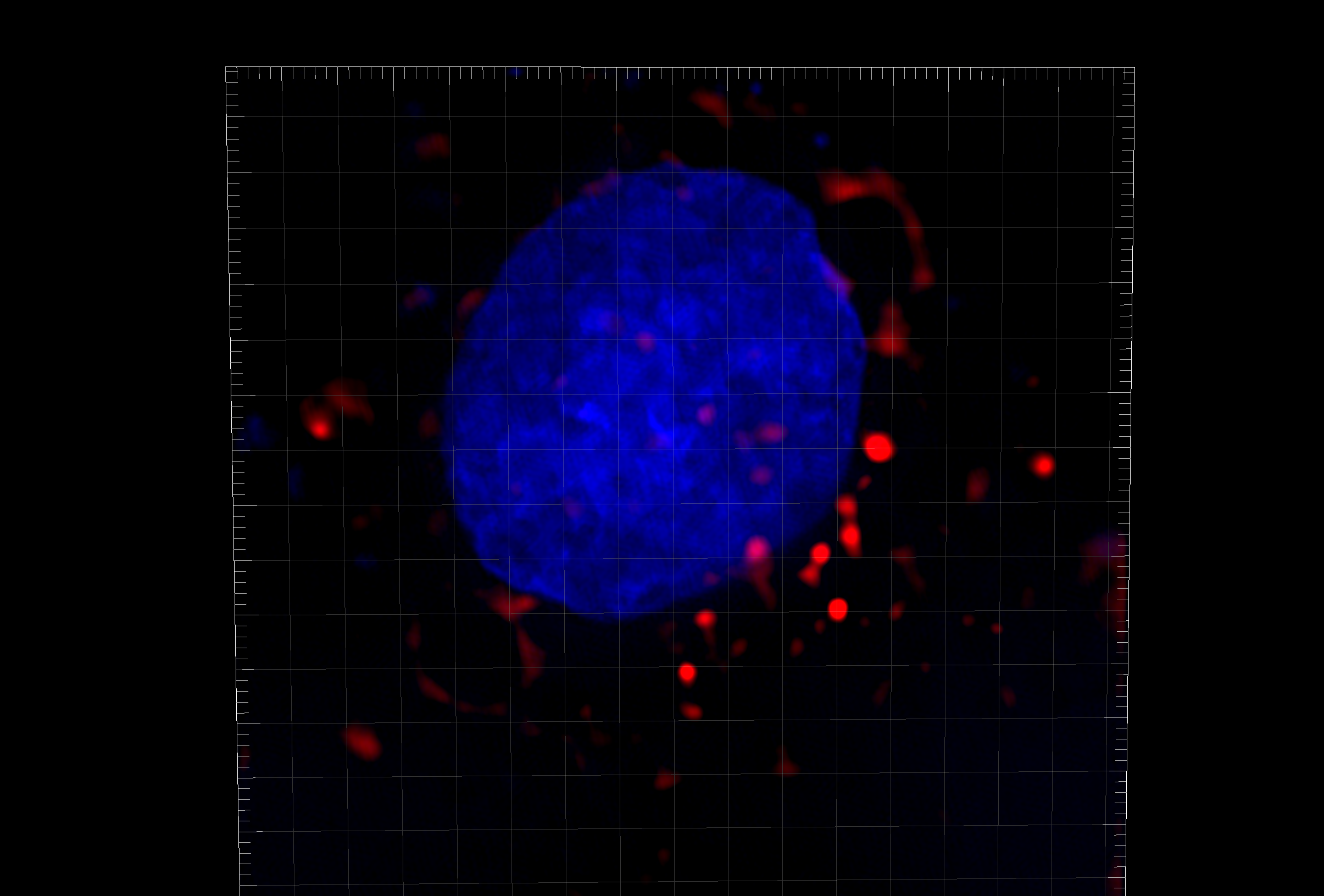
**

**Figure7 F, right:**

**siMCUB#1 Merge**

**
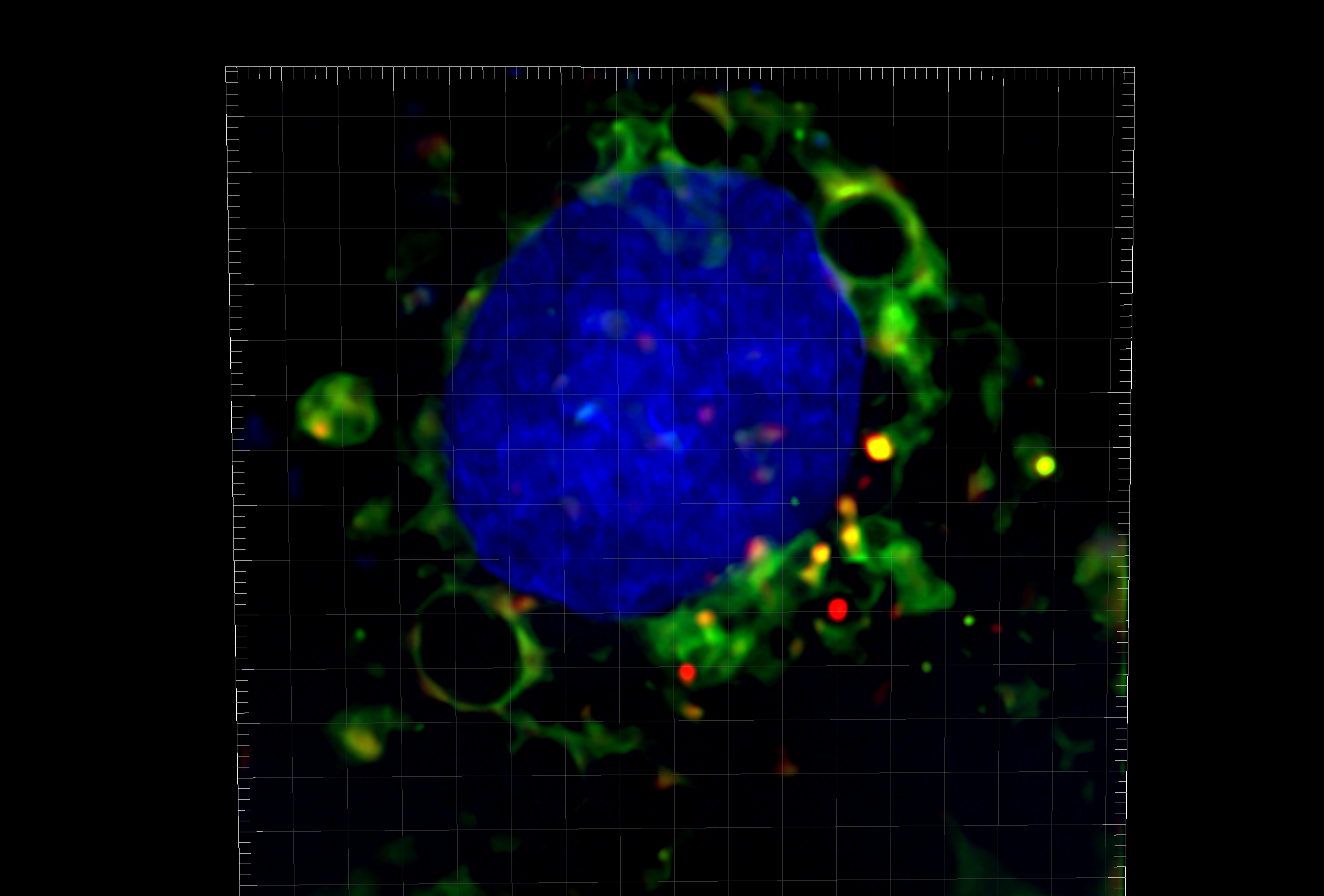
**

**Figure7 F, right:**

**siMCUB#1 Enlarge**

**
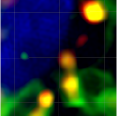
**

**Figure7 F, right:**

**siMCUB#2 Mitotracker**

**
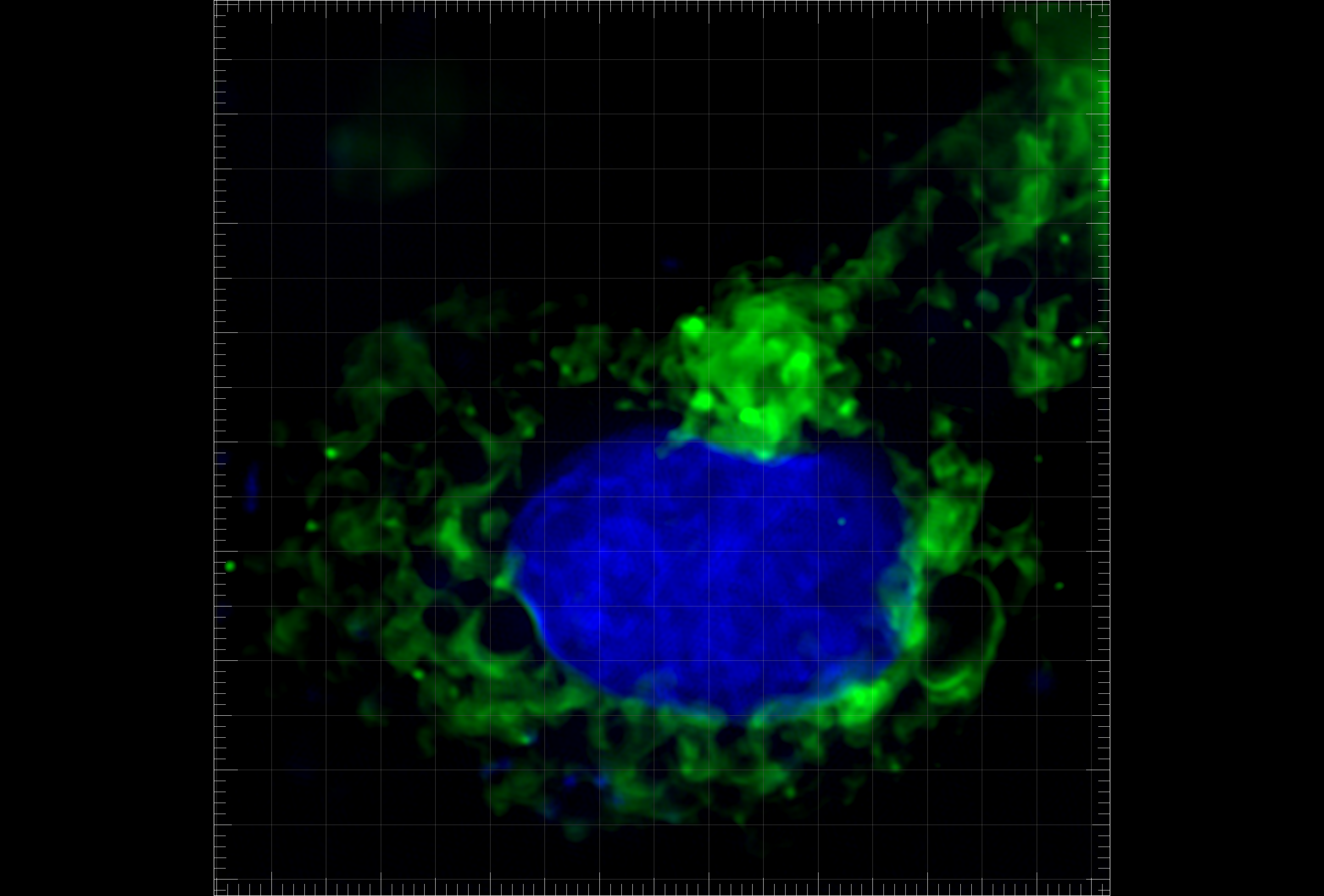
**

**Figure7 F, right:**

**siMCUB#2 Lysotracker**

**
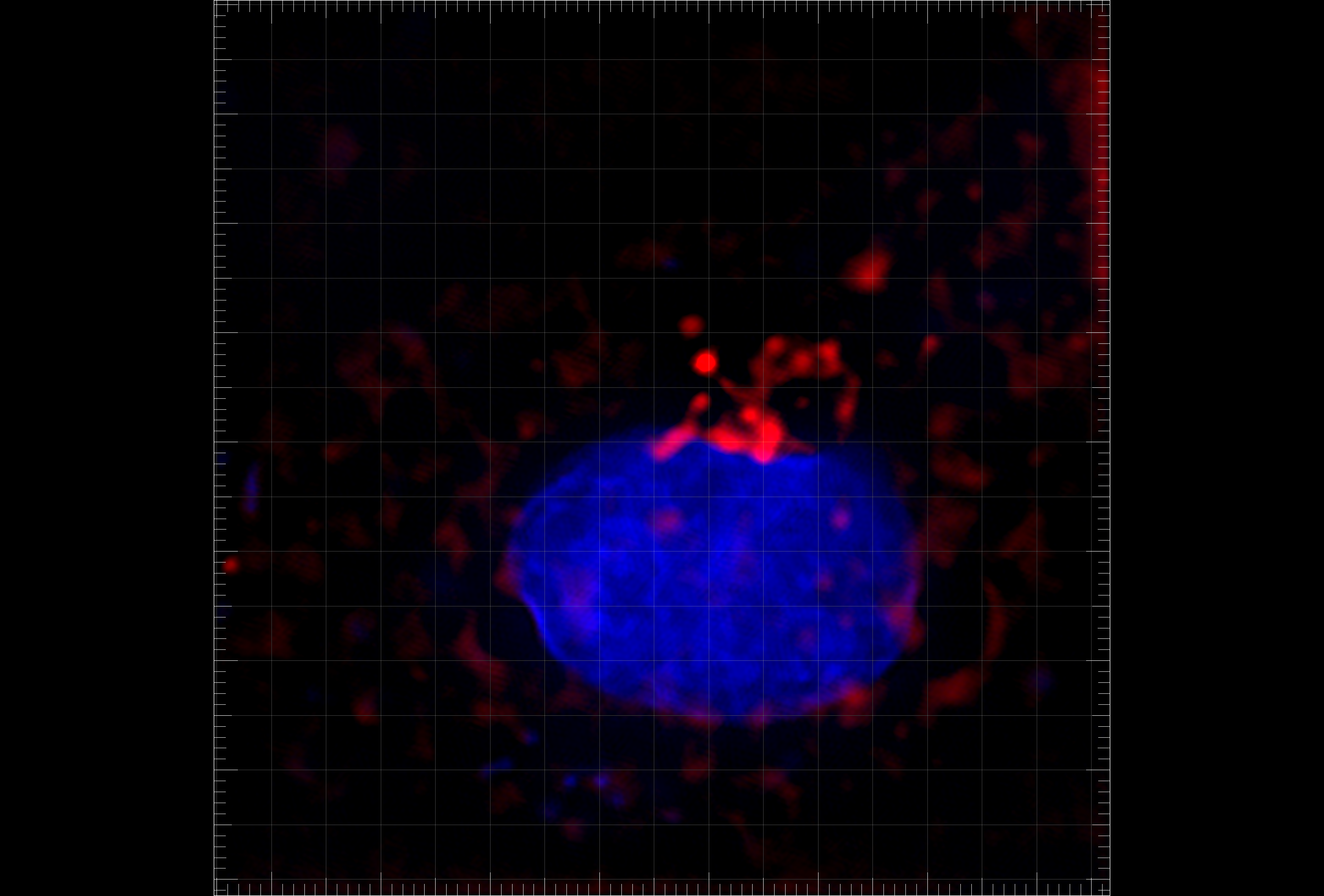
**

**Figure7 F, right:**

**siMCUB#2 Merge**

**
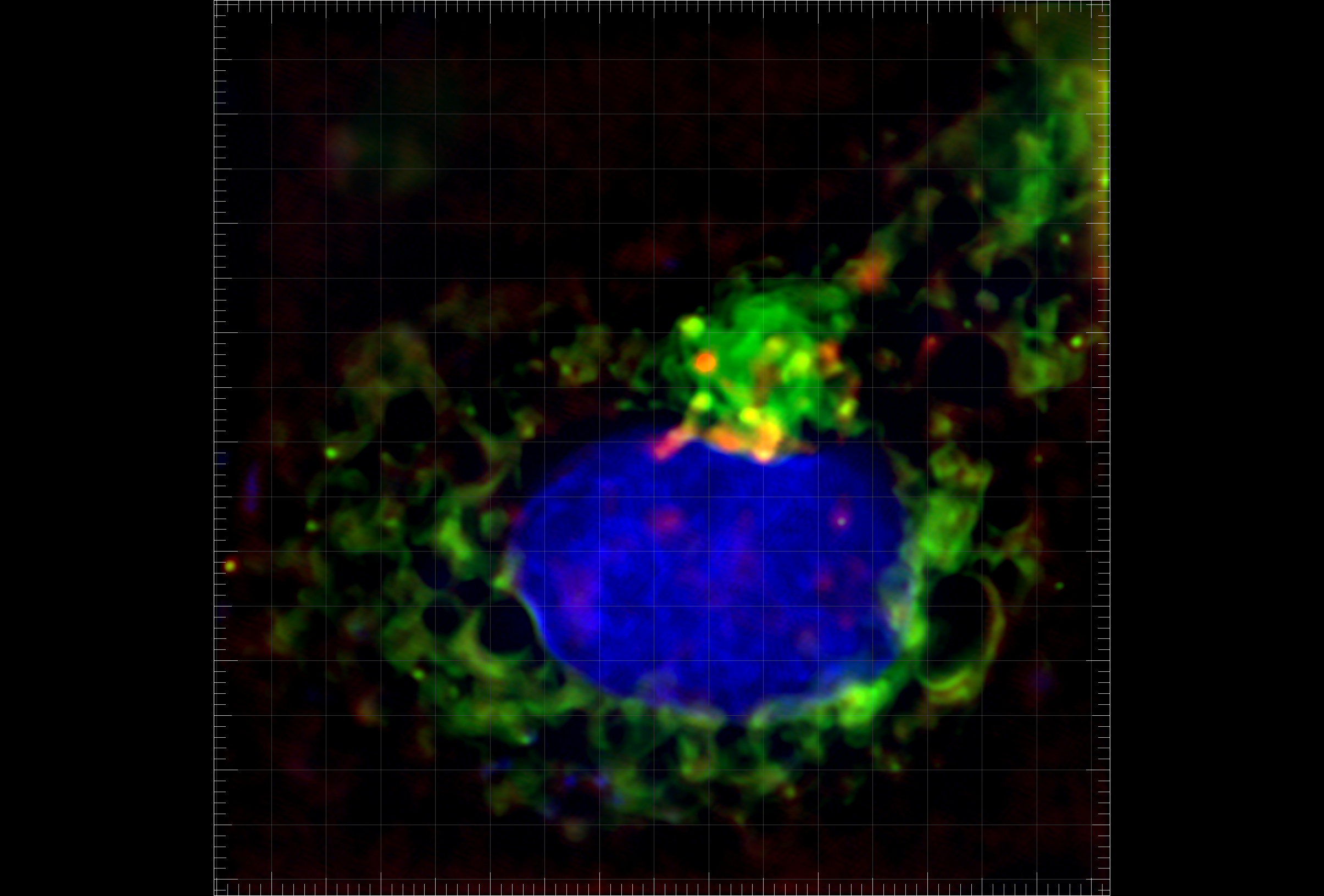
**

**Figure7 F, right:**

**siMCUB#2 Enlarge**

**
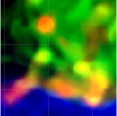
**
