# Supplementary material for: MCUB Inhibits PRKN‐Dependent Mitophagic Degradation of PD‐L1 to Promote Immune Evasion in Bladder Cancer
Source: Adv Sci (Weinh). 2025 Nov 12;13(5):e14764. doi: 10.1002/advs.202514764 (PMC12849890; doi:10.1002/advs.202514764)
Supplement: Supplementary file 2 — Supporting Information [file ADVS-13-e14764-s002.zip › Figure8.docx]

**Figure8:**

**Figure8 B: MCUB**

**Group：(1) siNC (2) siMCUB#1 (3) siMCUB#2 (4) siMCUB#3**


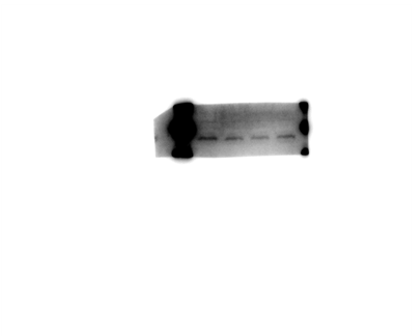


**Figure8 B: PD-L1**

**Group：(1) siNC (2) siMCUB#1 (3) siMCUB#2 (4) siMCUB#3**

**
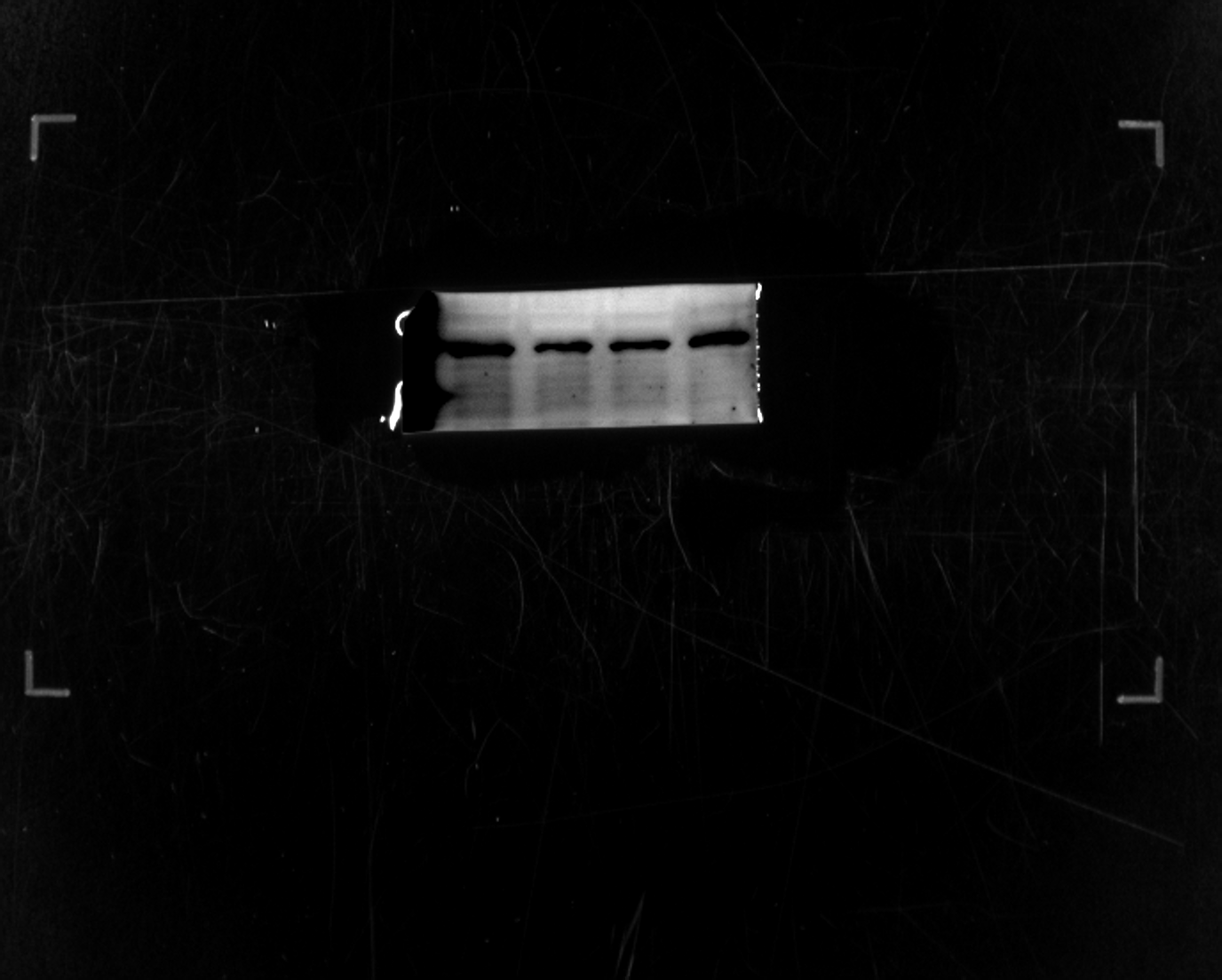
**

**Figure8 B: PRKN**

**Group：(1) siNC (2) siMCUB#1 (3) siMCUB#2 (4) siMCUB#3**

**
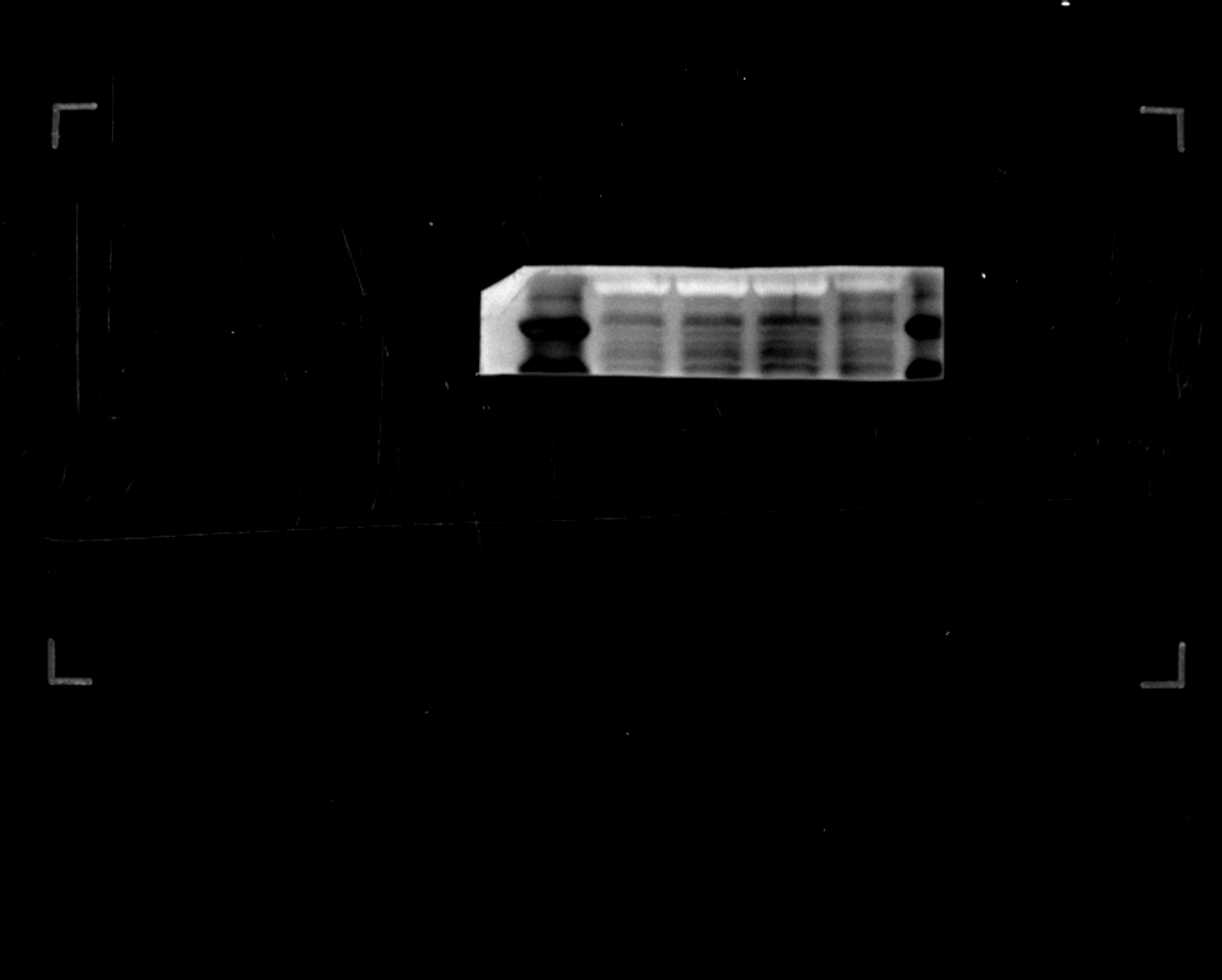
**

**Figure8 B: LC3**

**Group：(1) siNC (2) siMCUB#1 (3) siMCUB#2 (4) siMCUB#3**

**
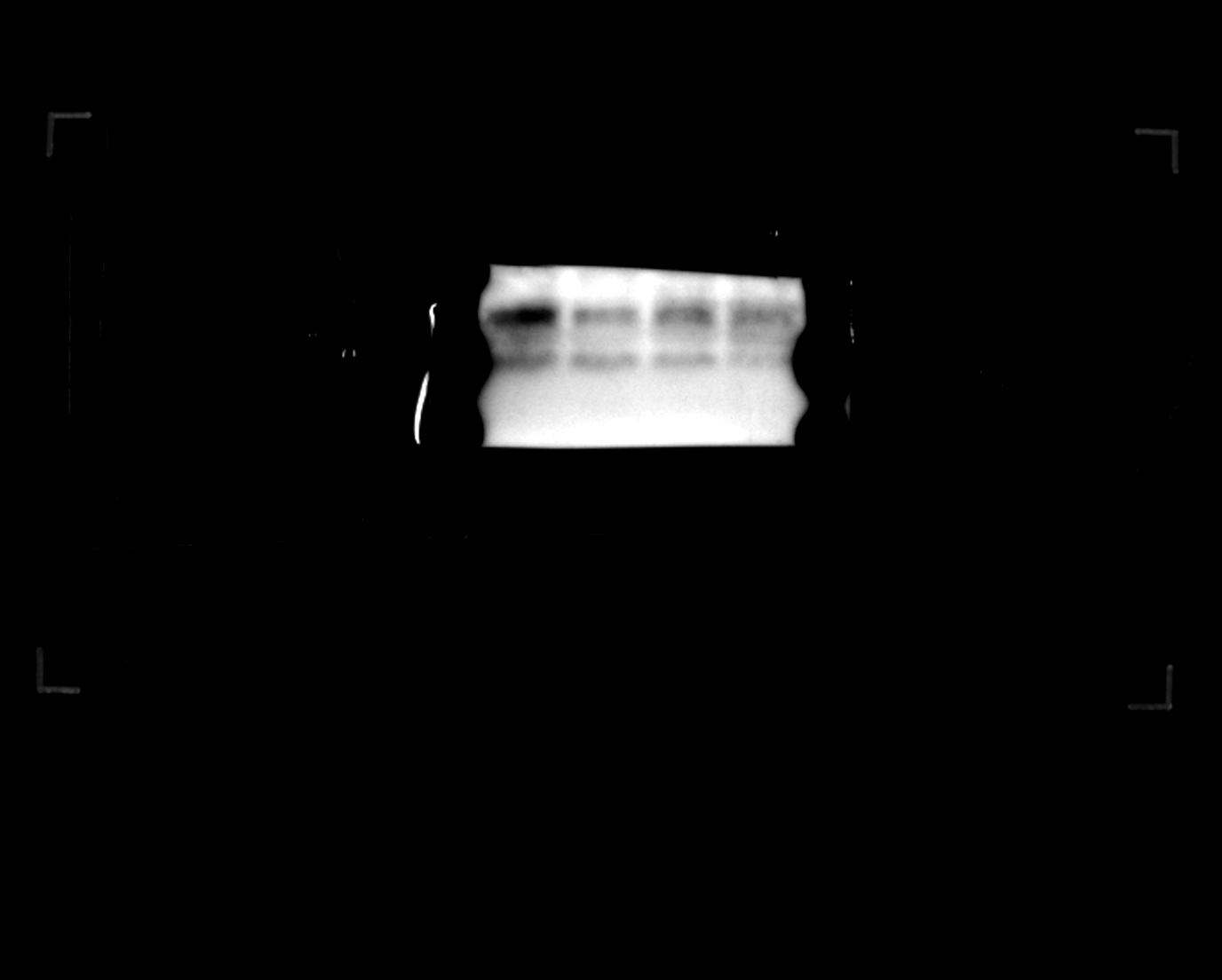
**

**Figure8 B: α-Tubulin**

**Group：(1) siNC (2) siMCUB#1 (3) siMCUB#2 (4) siMCUB#3**

**
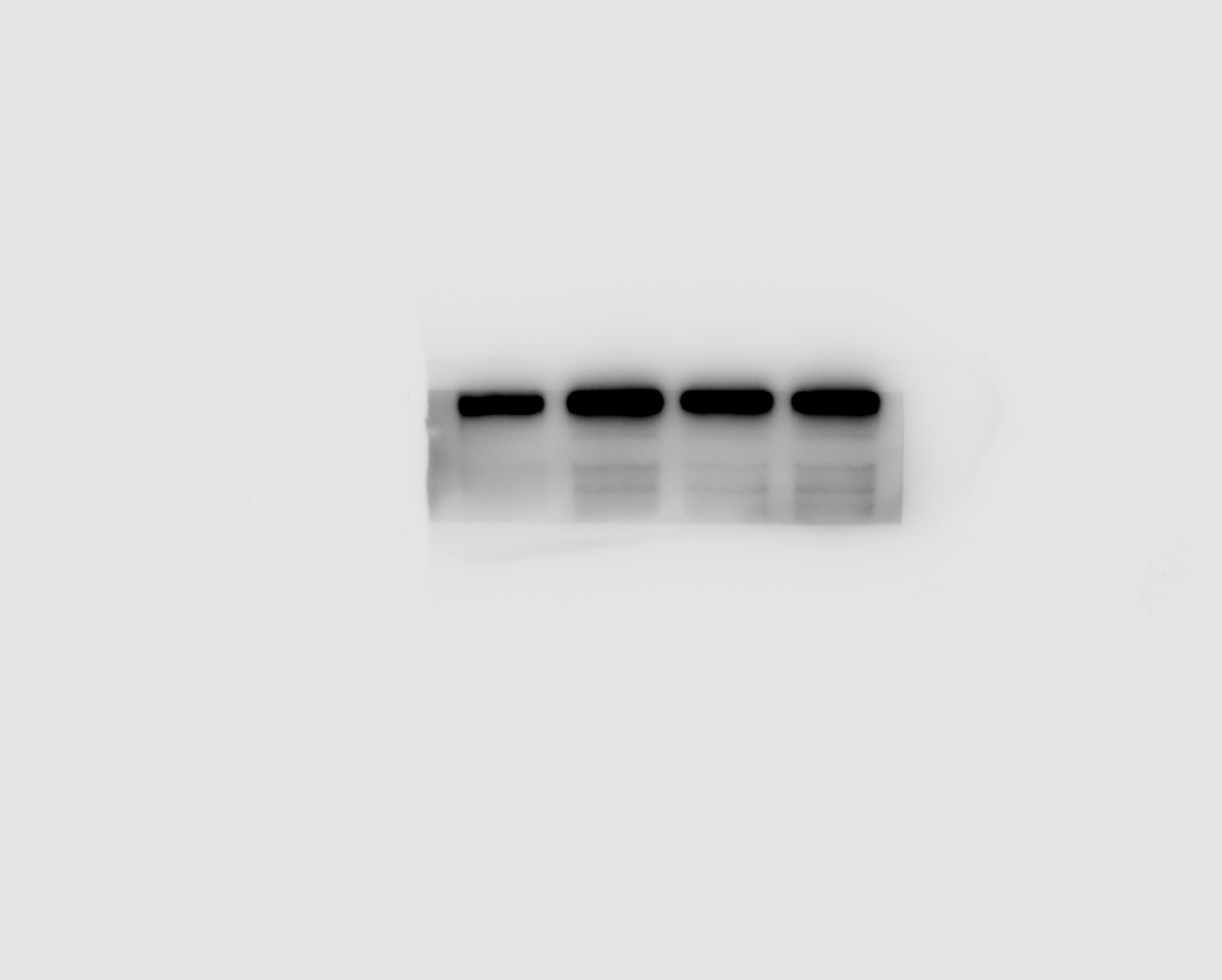
**

**
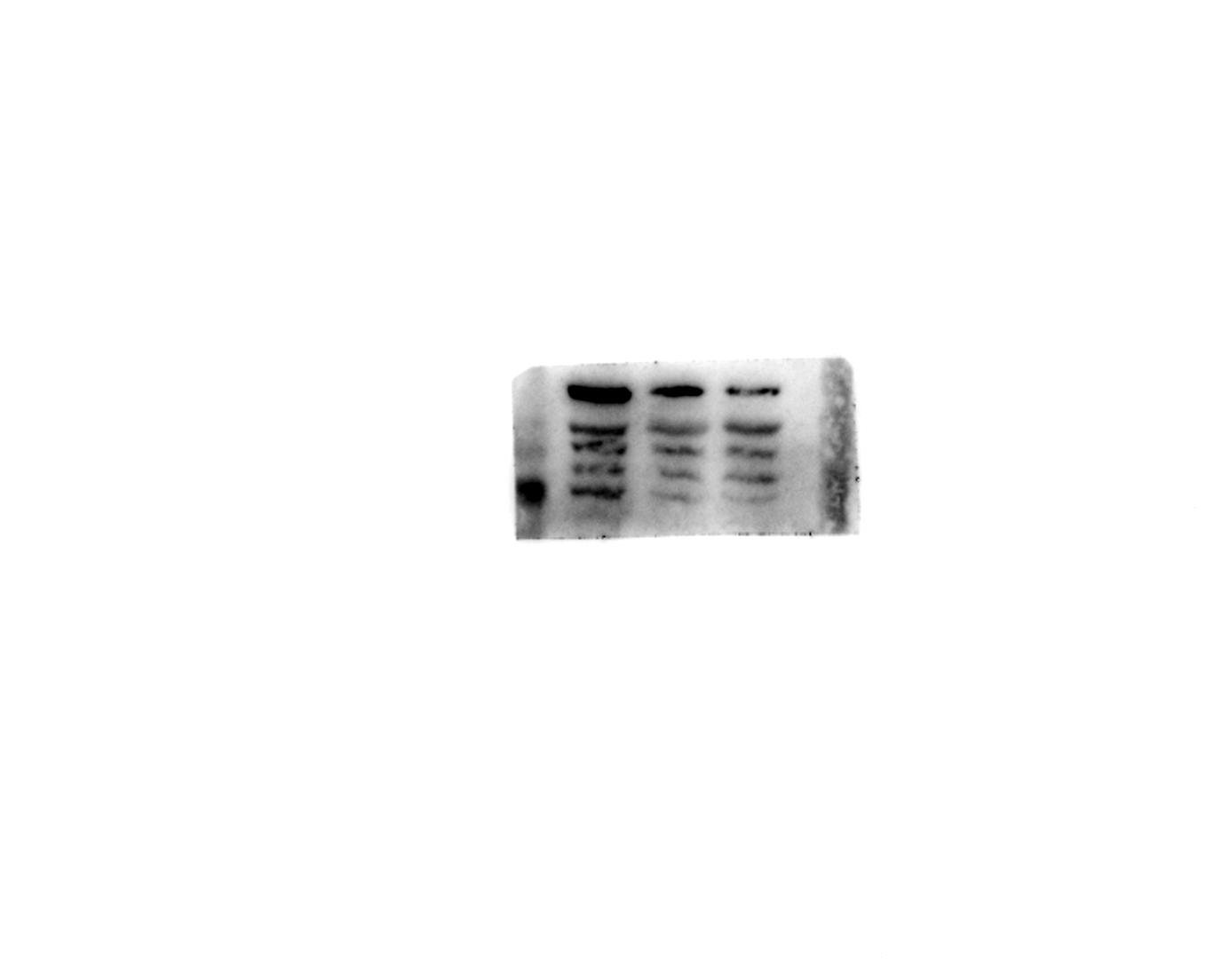
Figure8 C, left, UMUC3: MCUB**

**
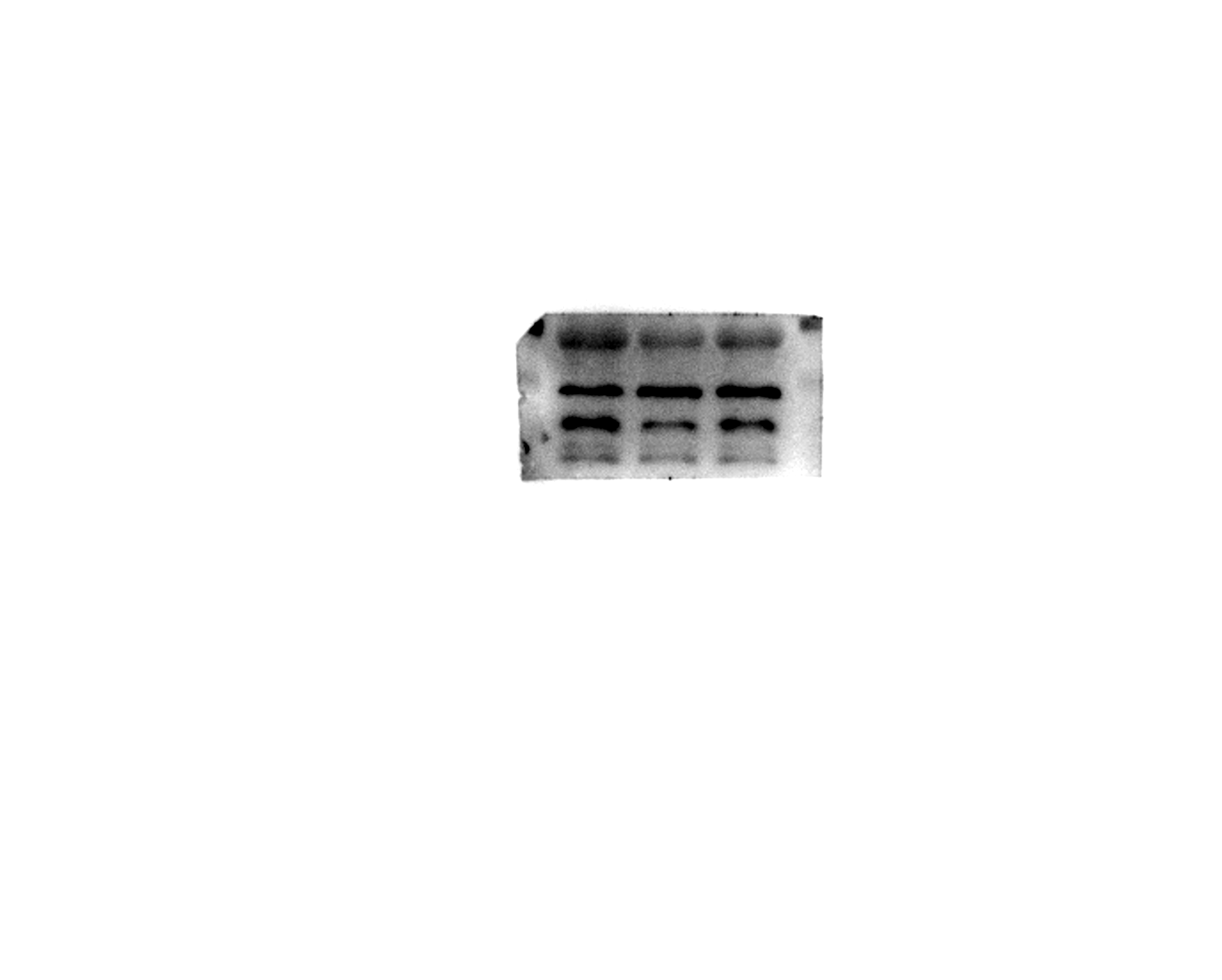
Figure8 C, left, UMUC3: PD-L1**

**
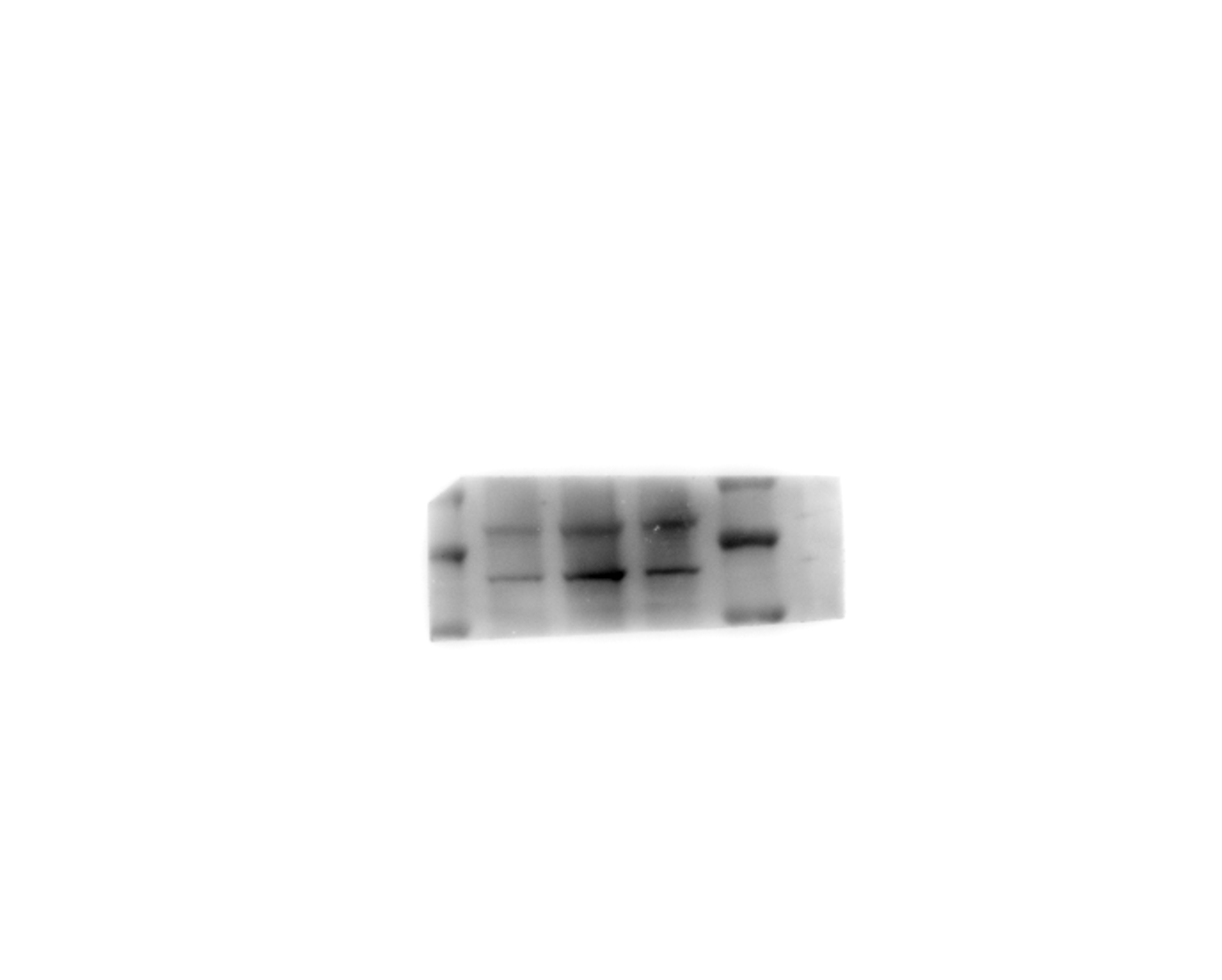
Figure8 C, left, UMUC3: PRKN**


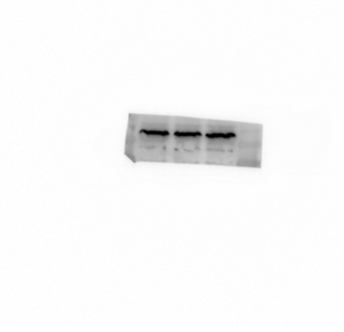
**Figure8 C, left, UMUC3: α-Tubulin**

**
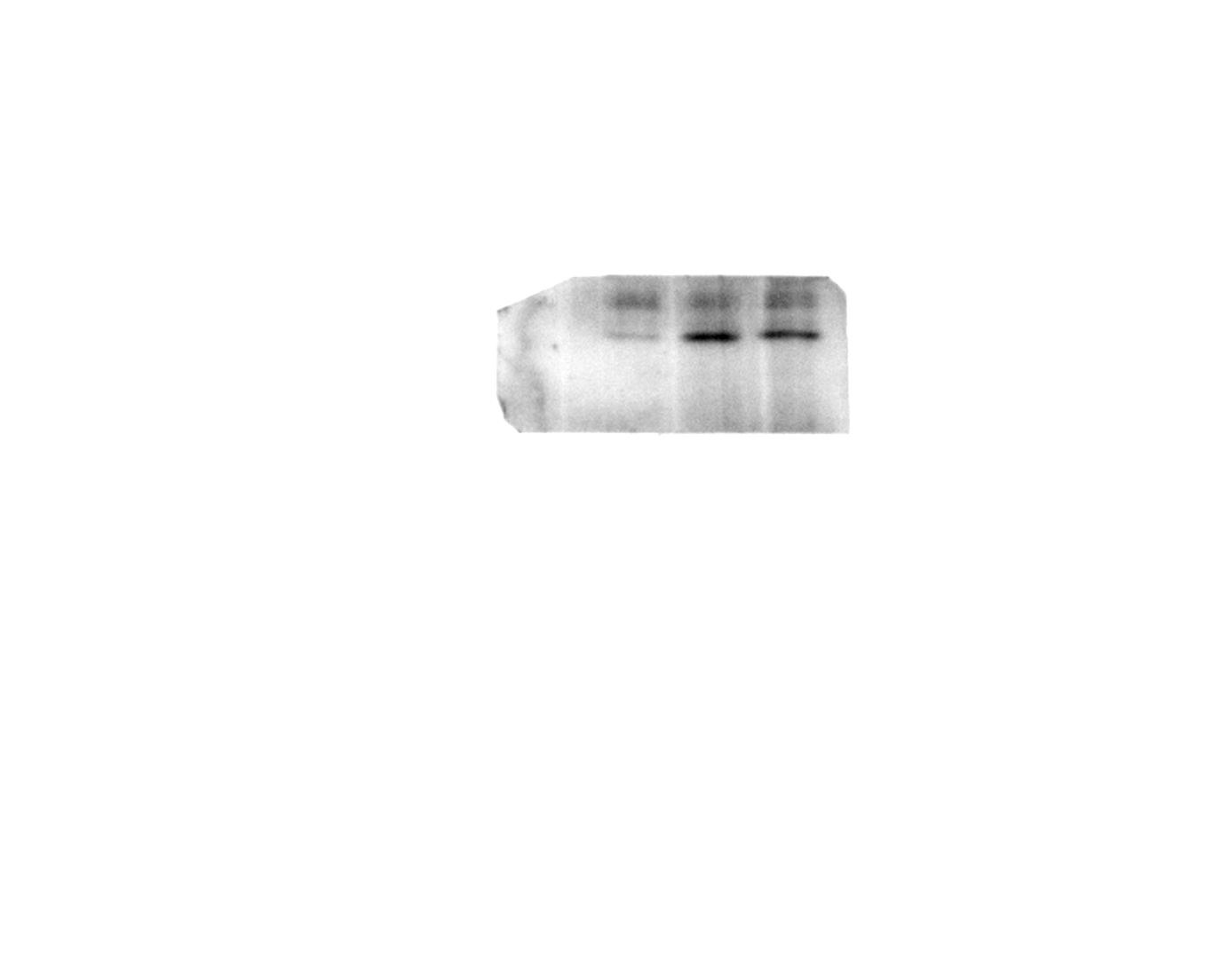
Figure8 C, right, UMUC3: MCUB**

**
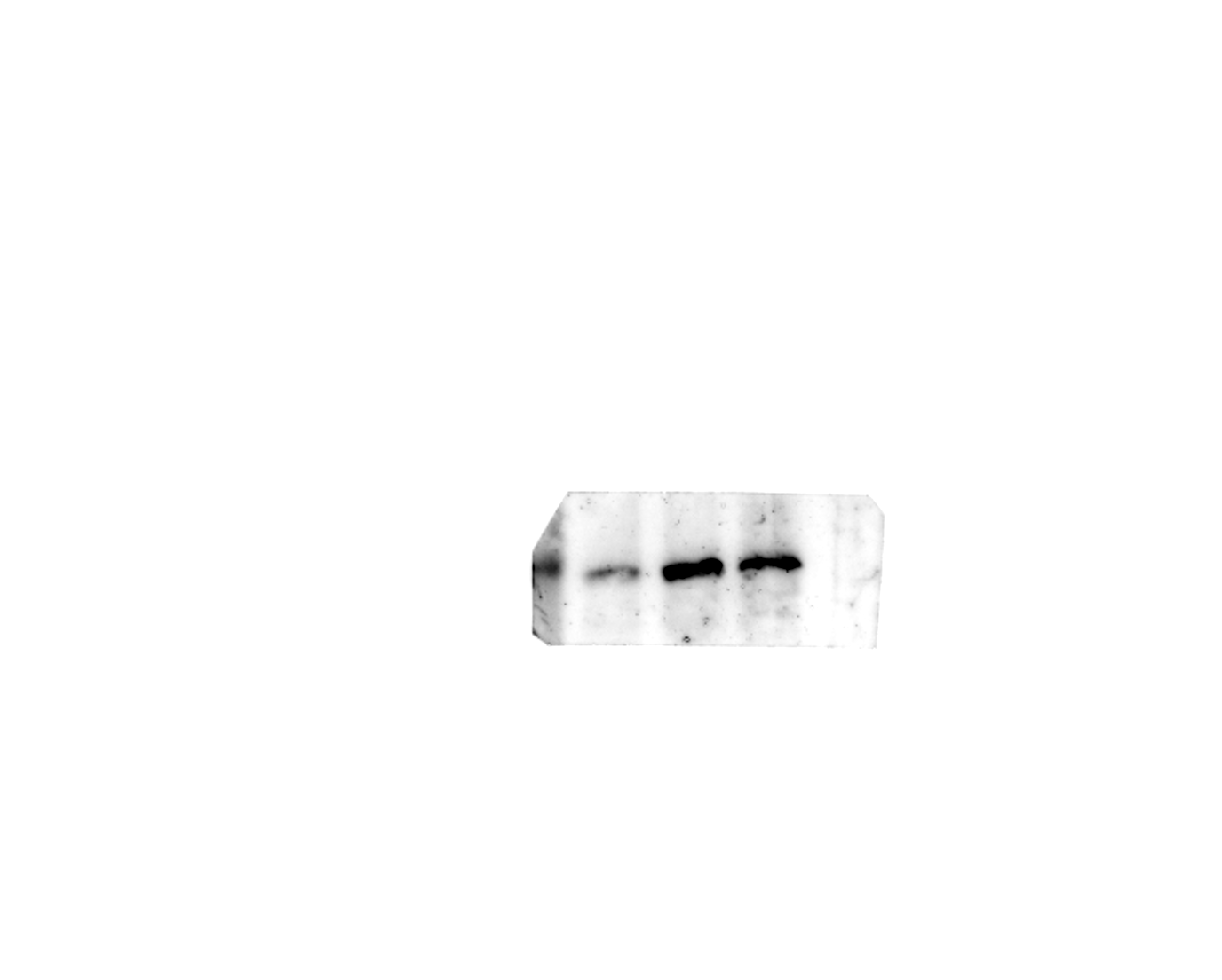
 Figure8 C, right, UMUC3: PD-L1**

**
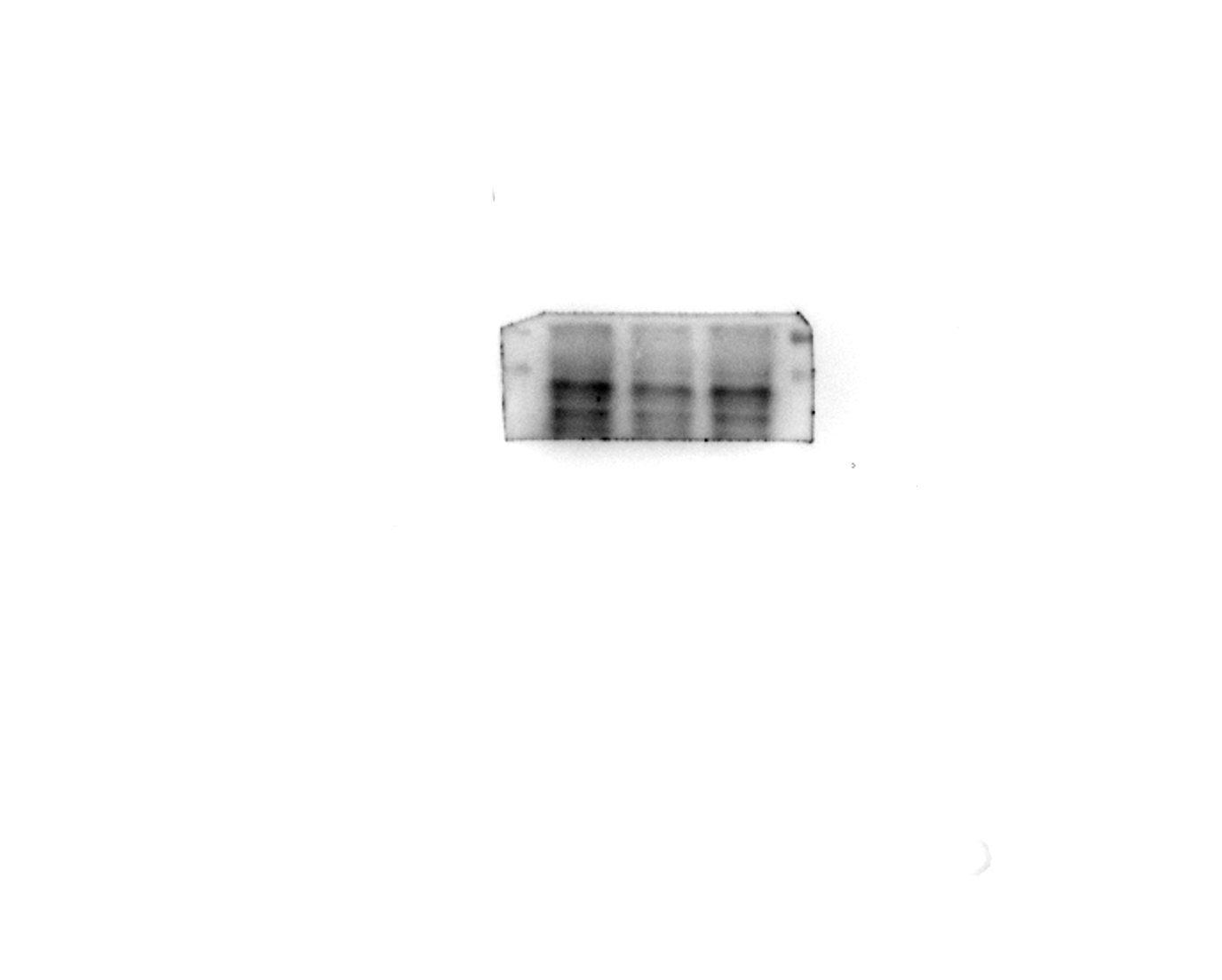
 Figure8 C, right, UMUC3: PRKN**

**
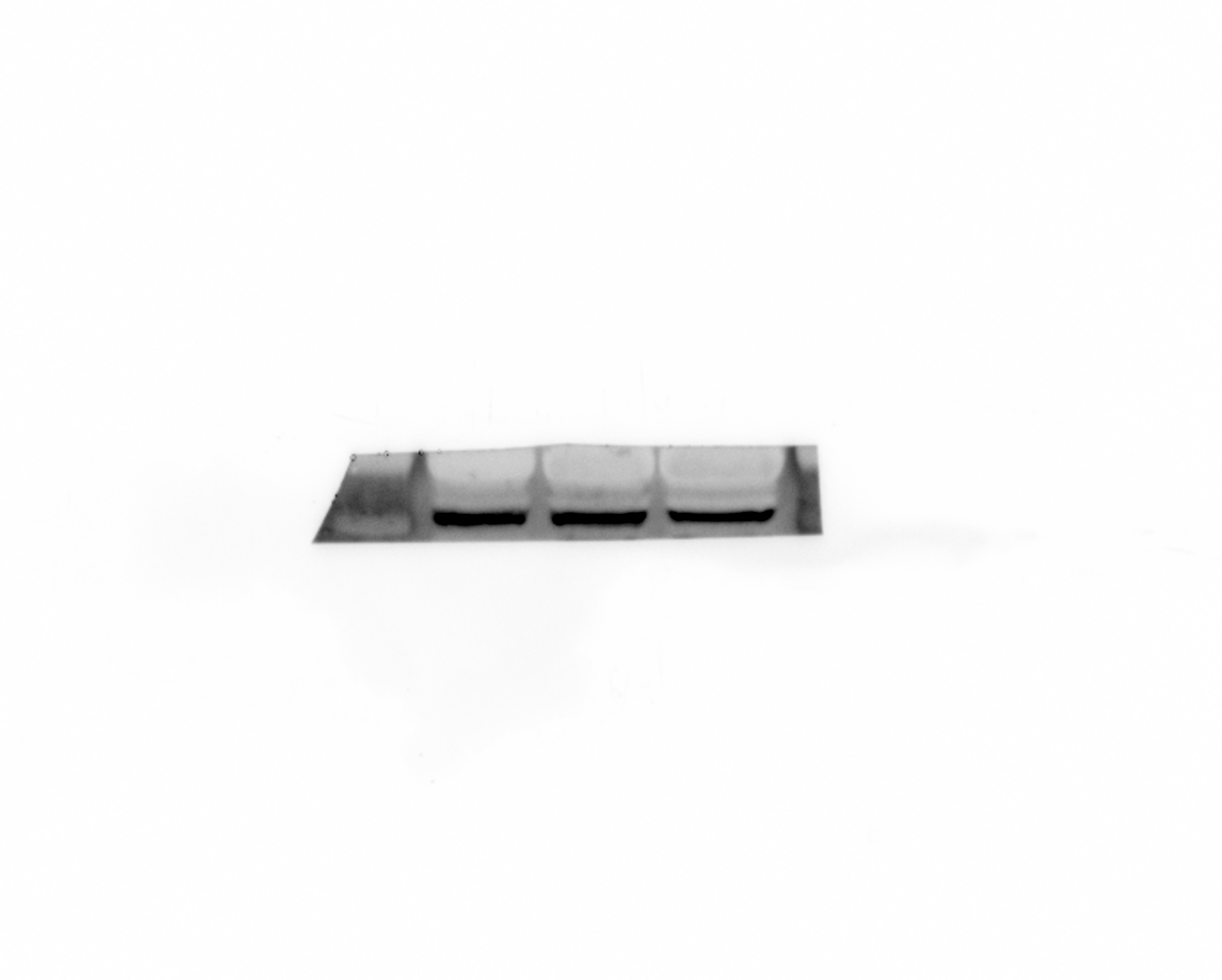
 Figure8 C, right, UMUC3: α-Tubulin**

**
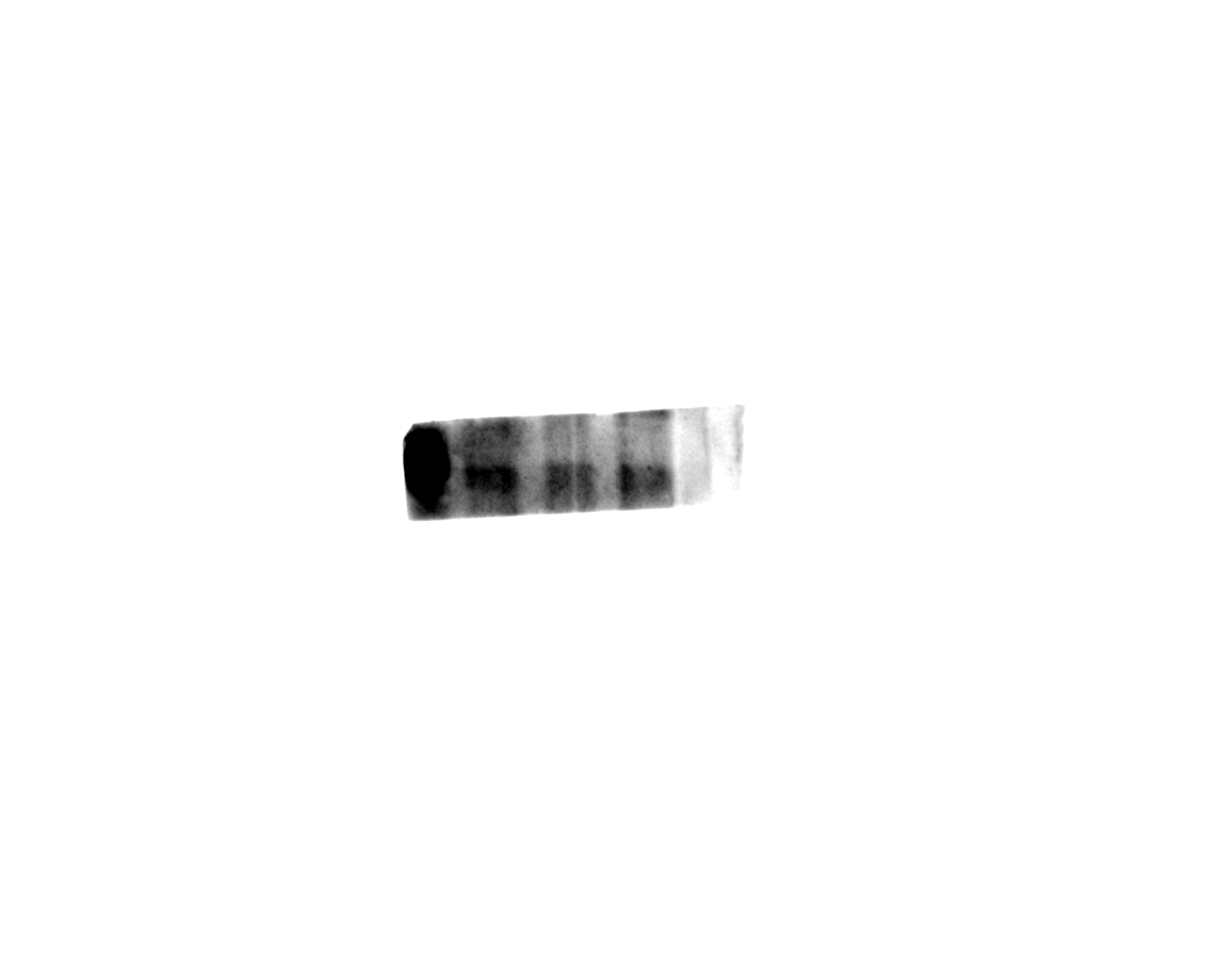
 Figure8 C, left, T24: MCUB**

**
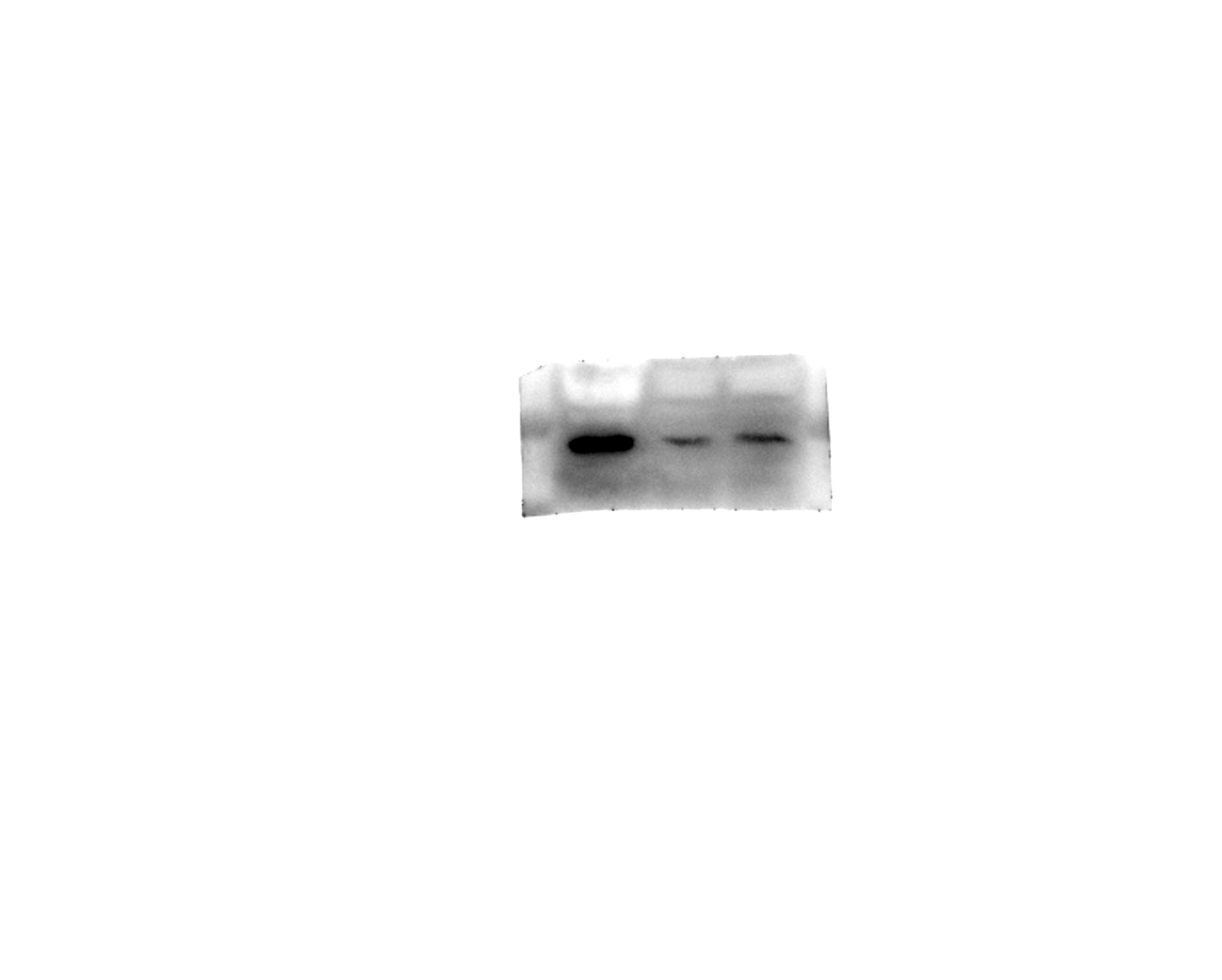
Figure8 C, left, T24: PD-L1**

**
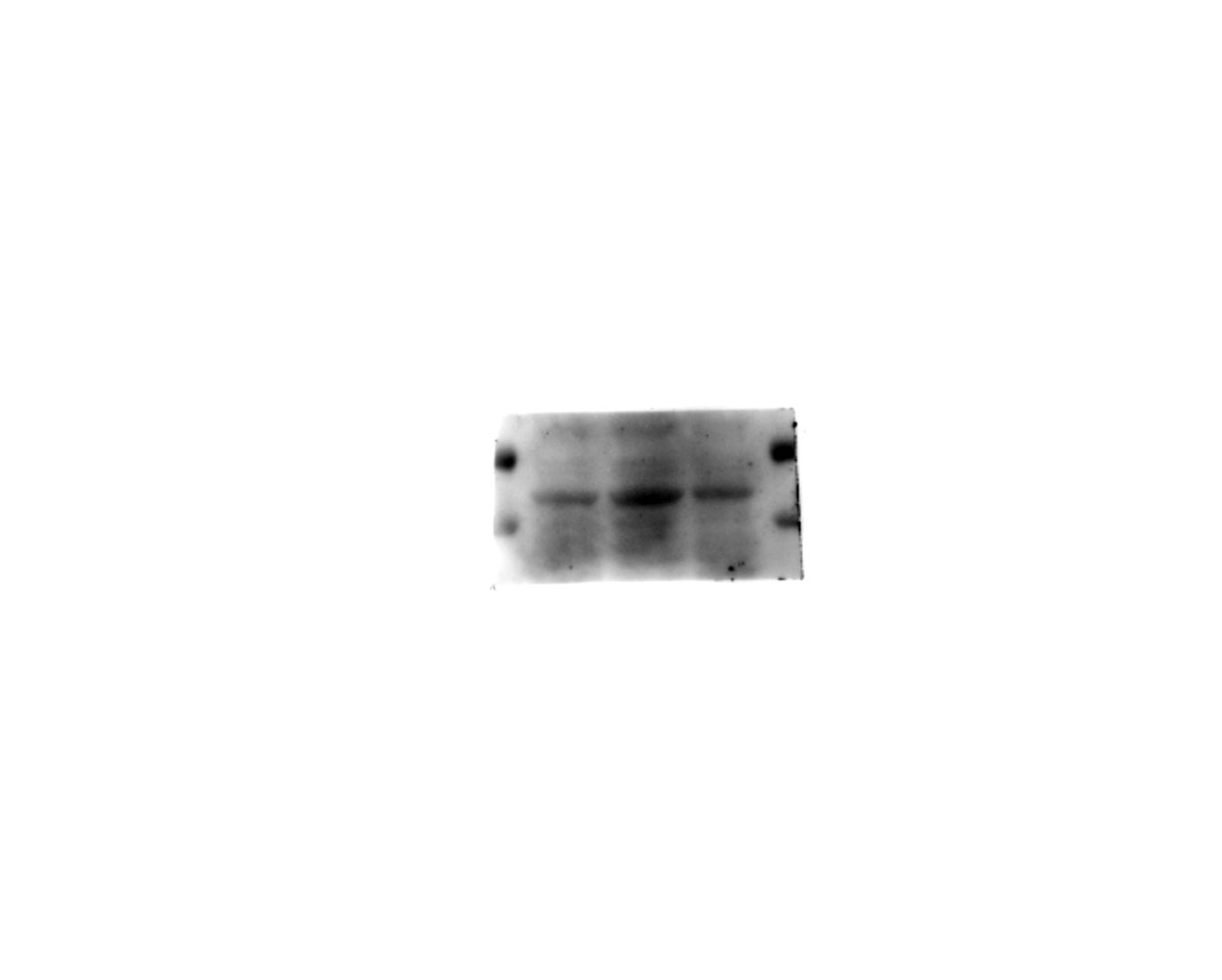
 Figure8 C, left, T24: PRKN**

**
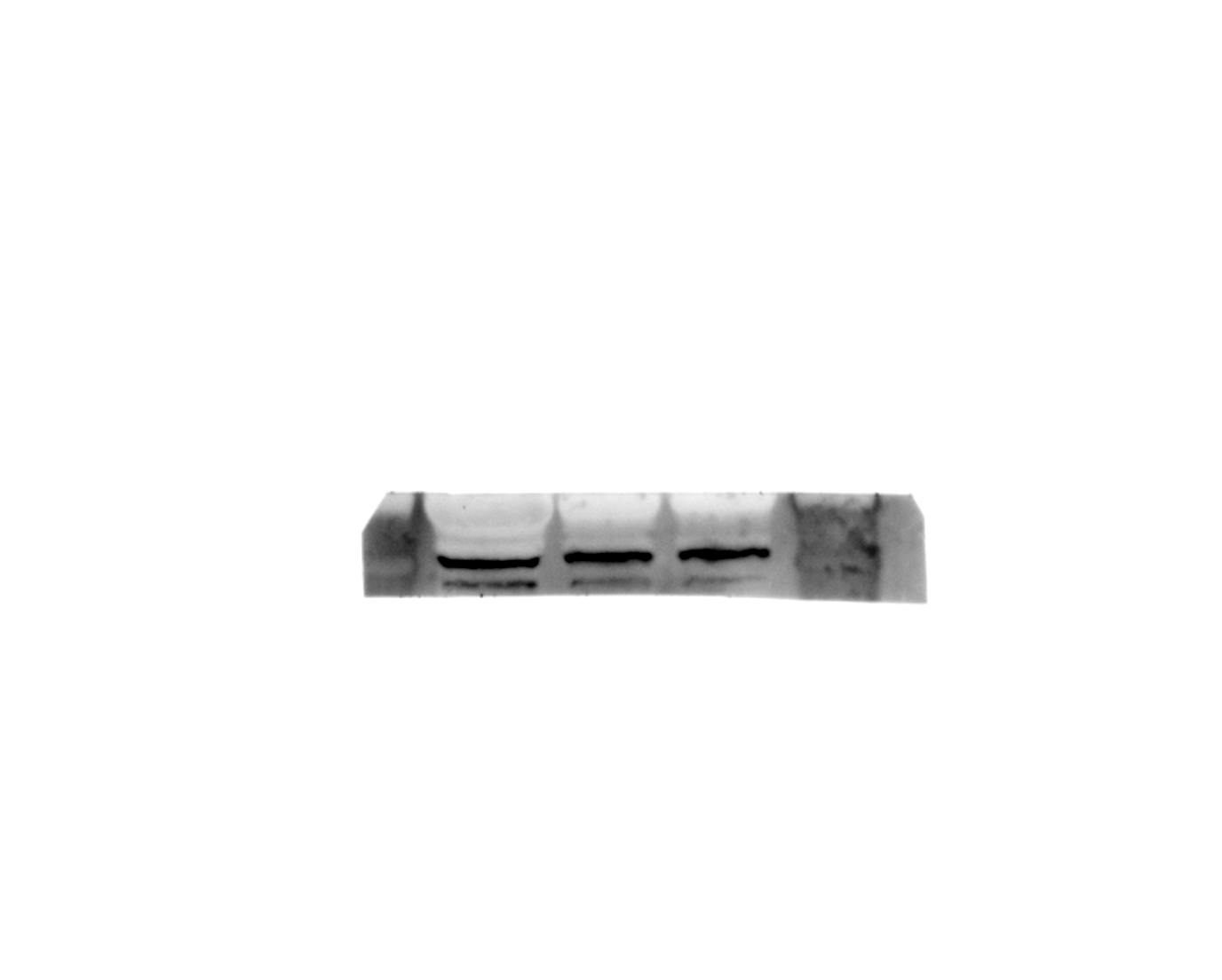
 Figure8 C, left, T24: α-Tubulin**

**
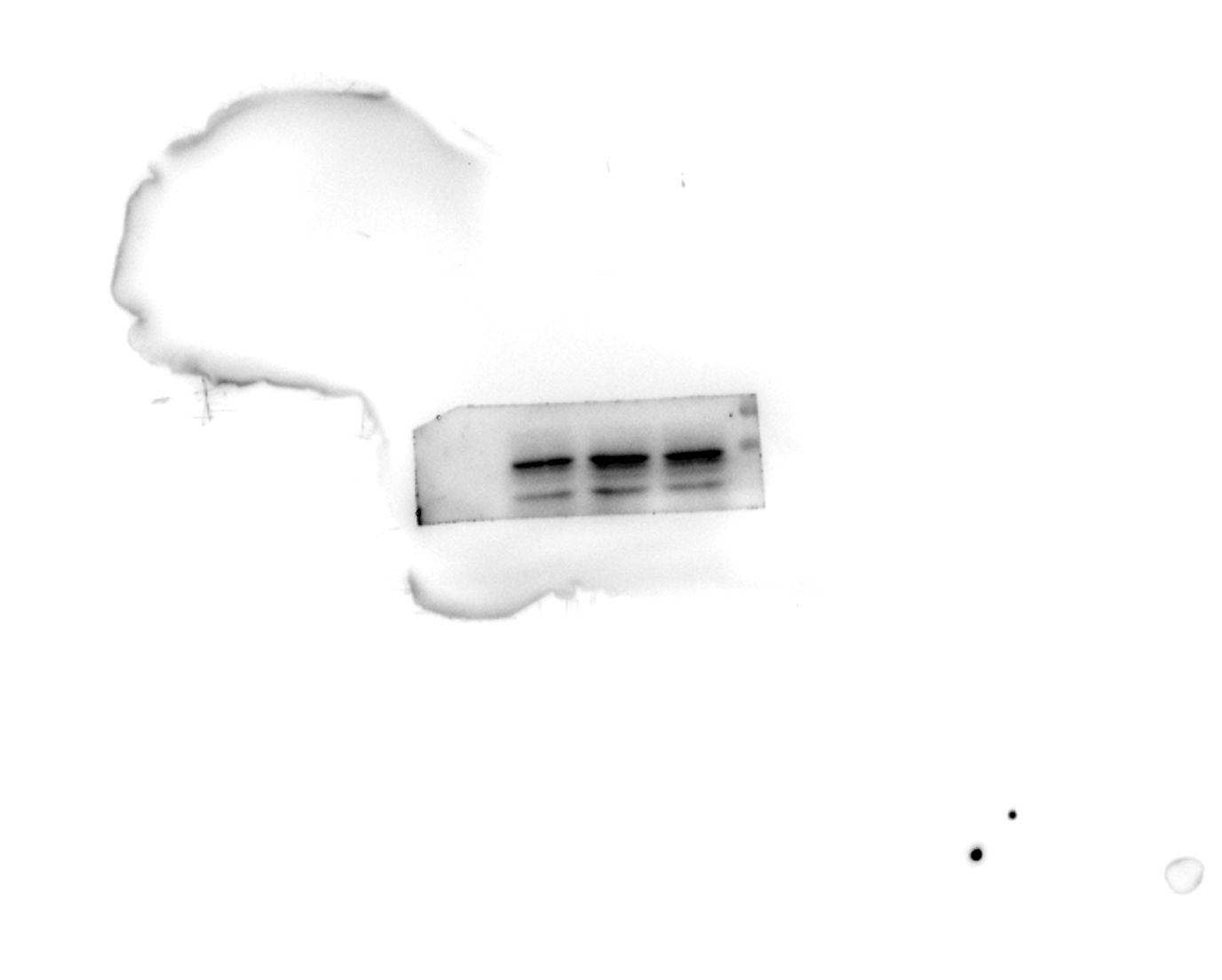
 Figure8 C, right, T24: MCUB**

**
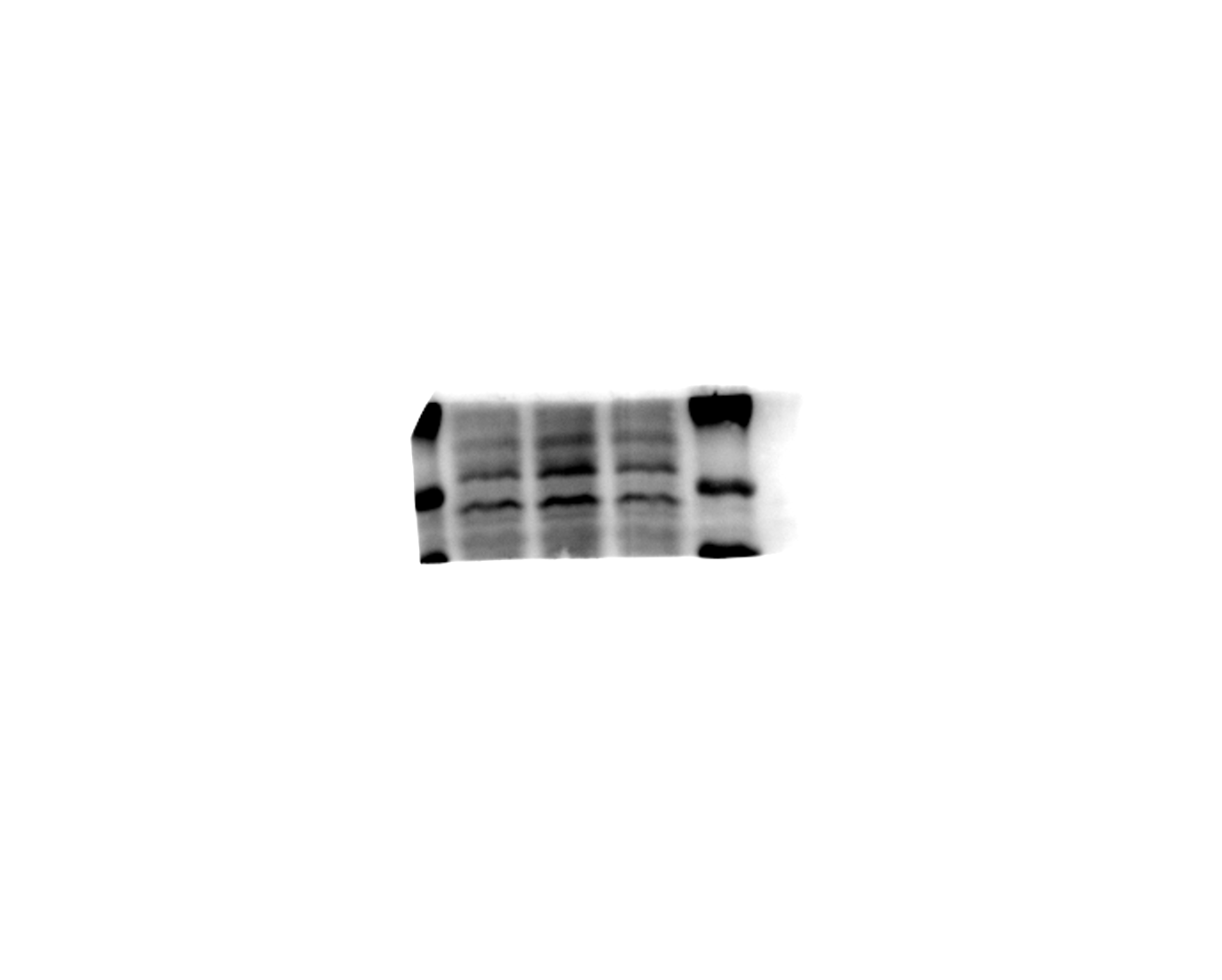
 Figure8 C, right, T24: PD-L1**


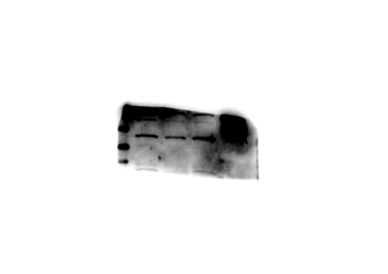
**Figure8 C, right, T24: PRKN**

**
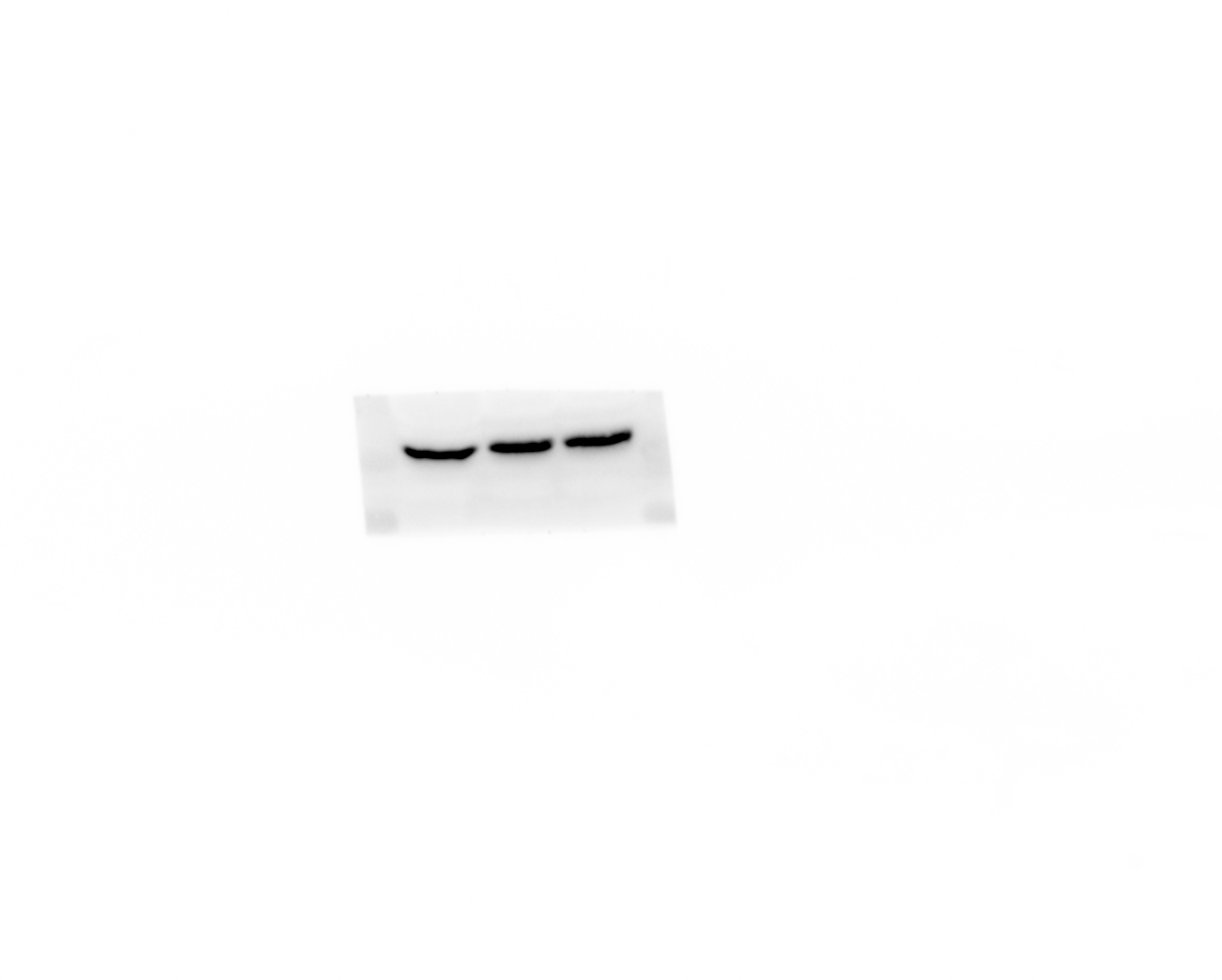
Figure8 C, right, T24: α-Tubulin**


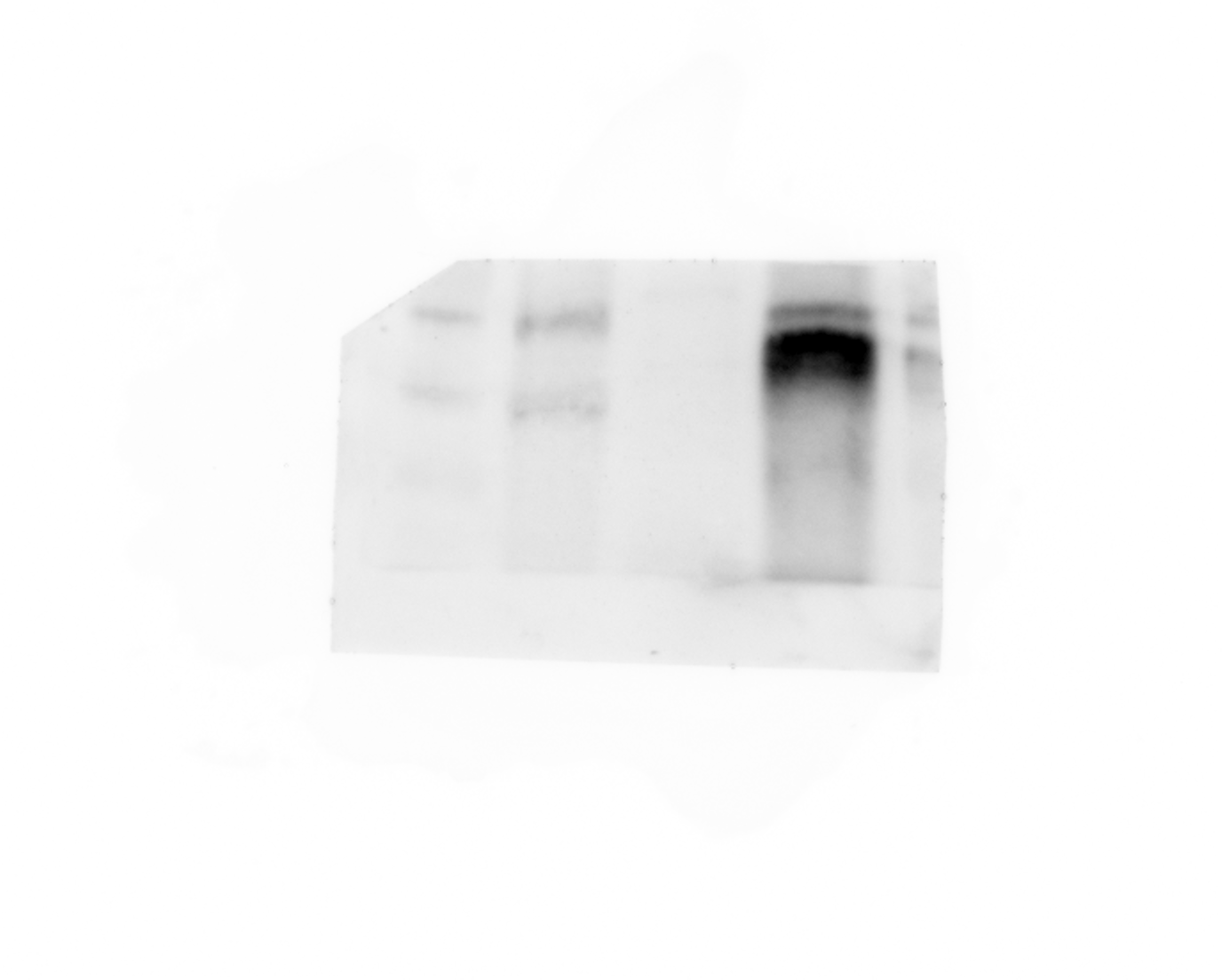
 **Figure8 D: PD-L1**


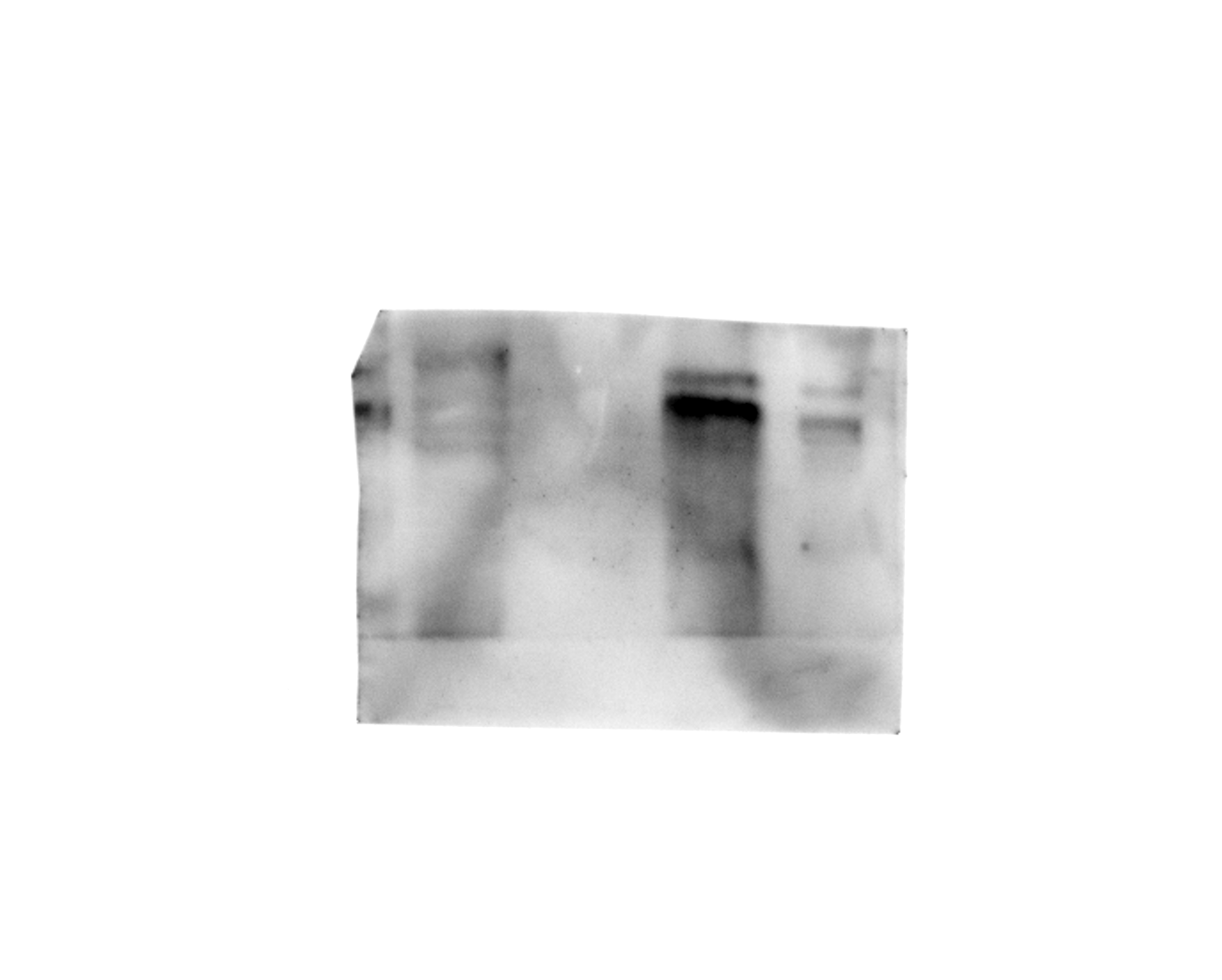
**Figure8 D: PRKN**


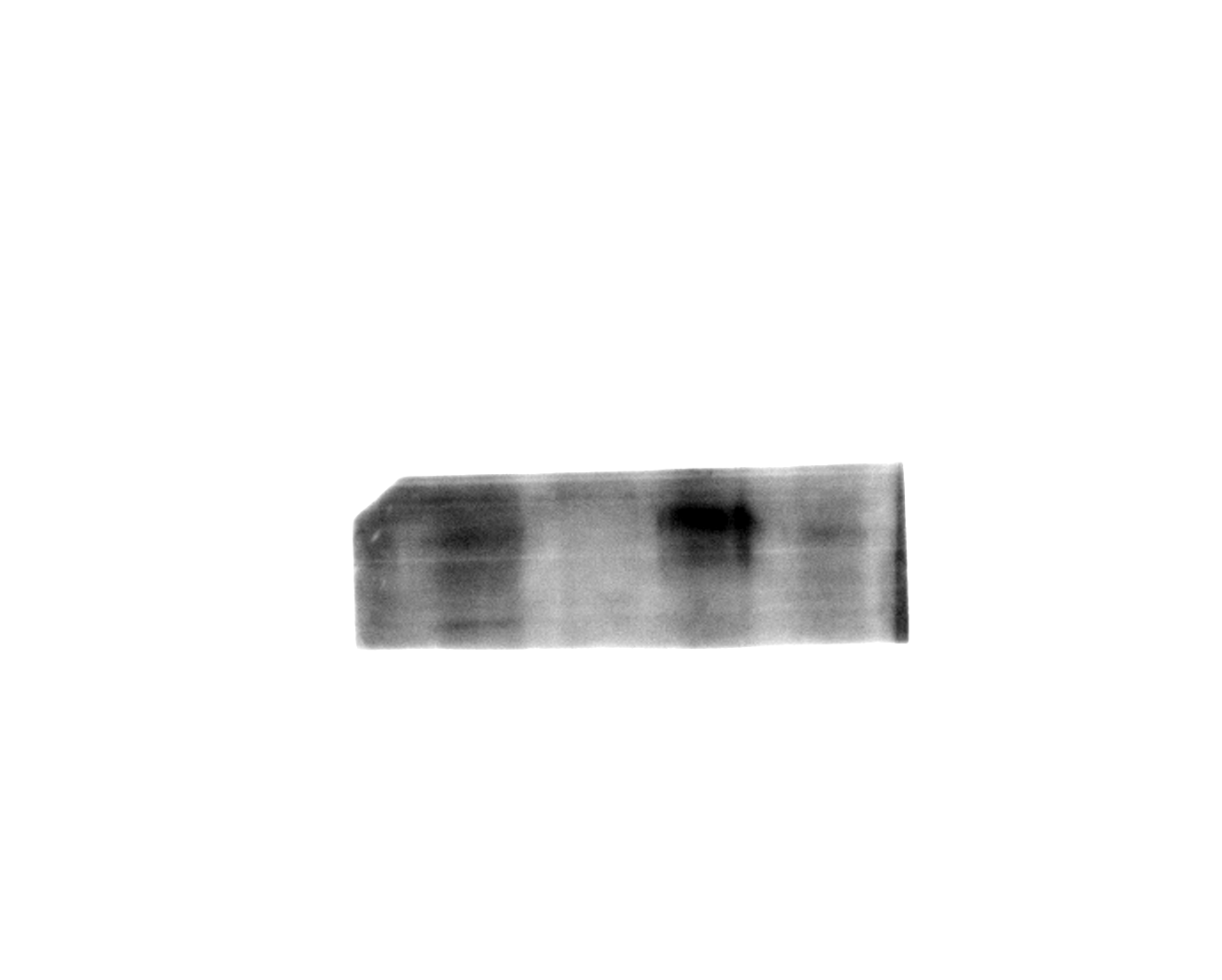
 **Figure8 D: PRKN**


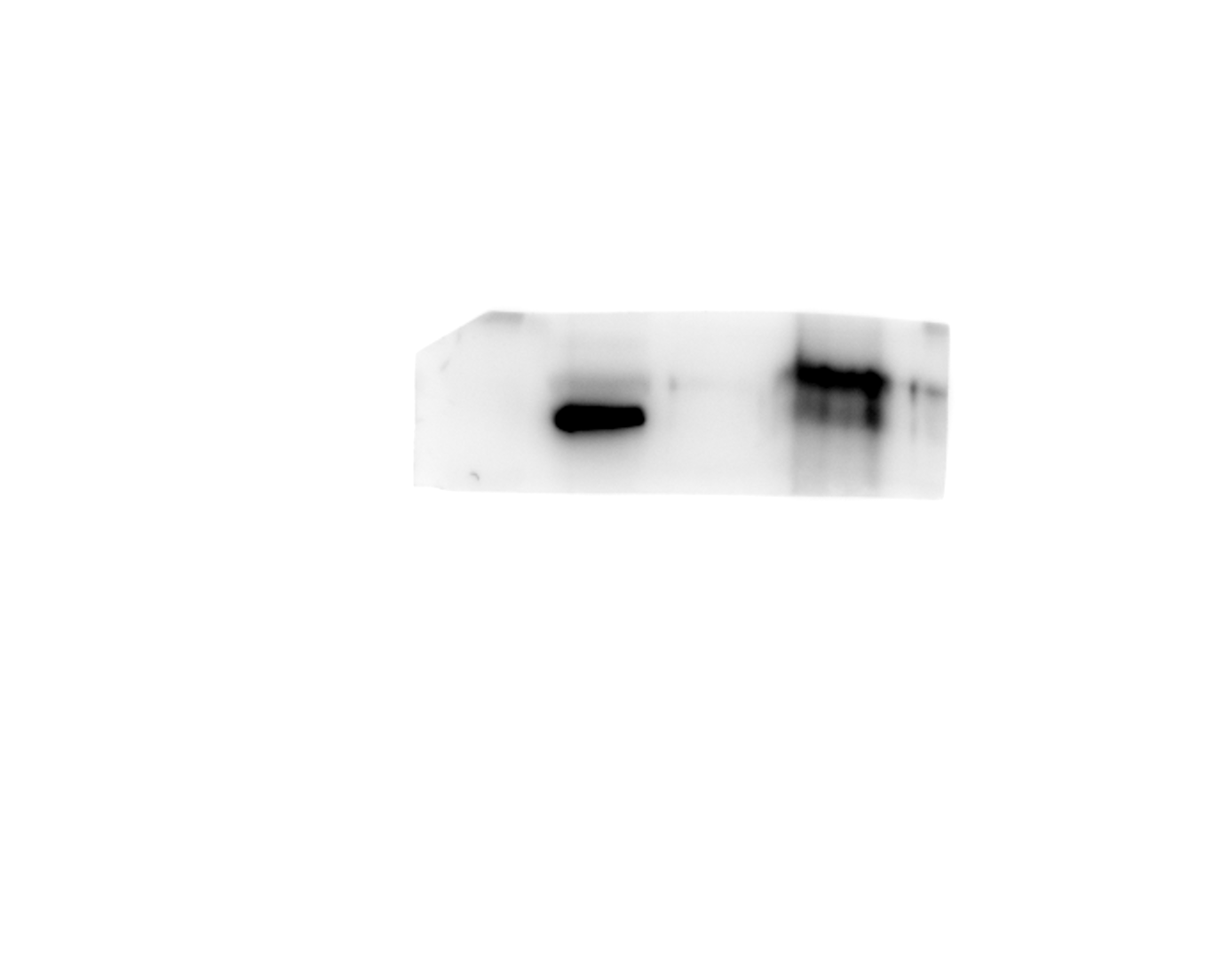
 **Figure8 D: PD-L1**
