# Supplementary material for: MCUB Inhibits PRKN‐Dependent Mitophagic Degradation of PD‐L1 to Promote Immune Evasion in Bladder Cancer
Source: Adv Sci (Weinh). 2025 Nov 12;13(5):e14764. doi: 10.1002/advs.202514764 (PMC12849890; doi:10.1002/advs.202514764)

**Figure9:**

**Figure9 A: shNC**


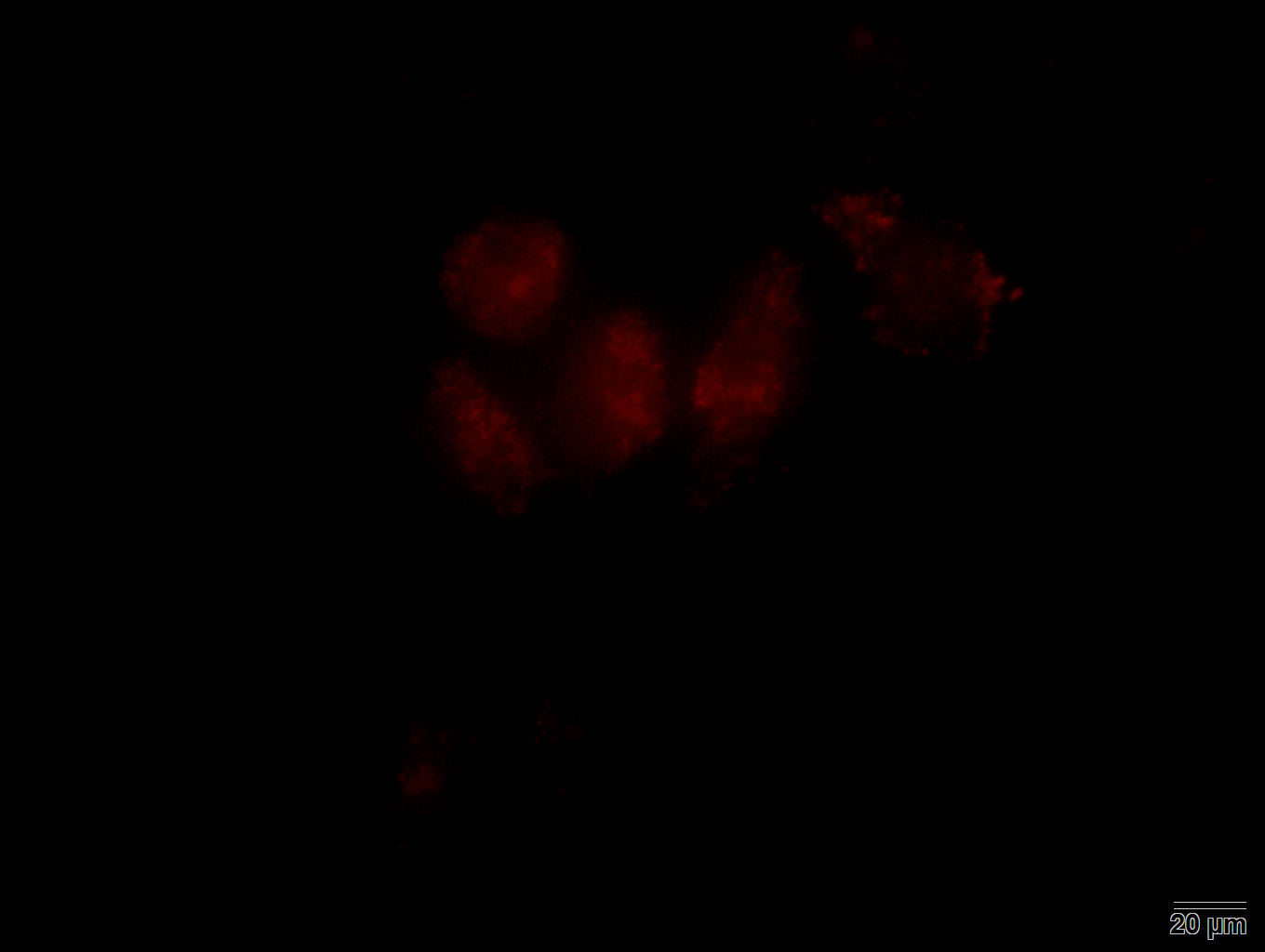


**Figure9 A: shMCUB**


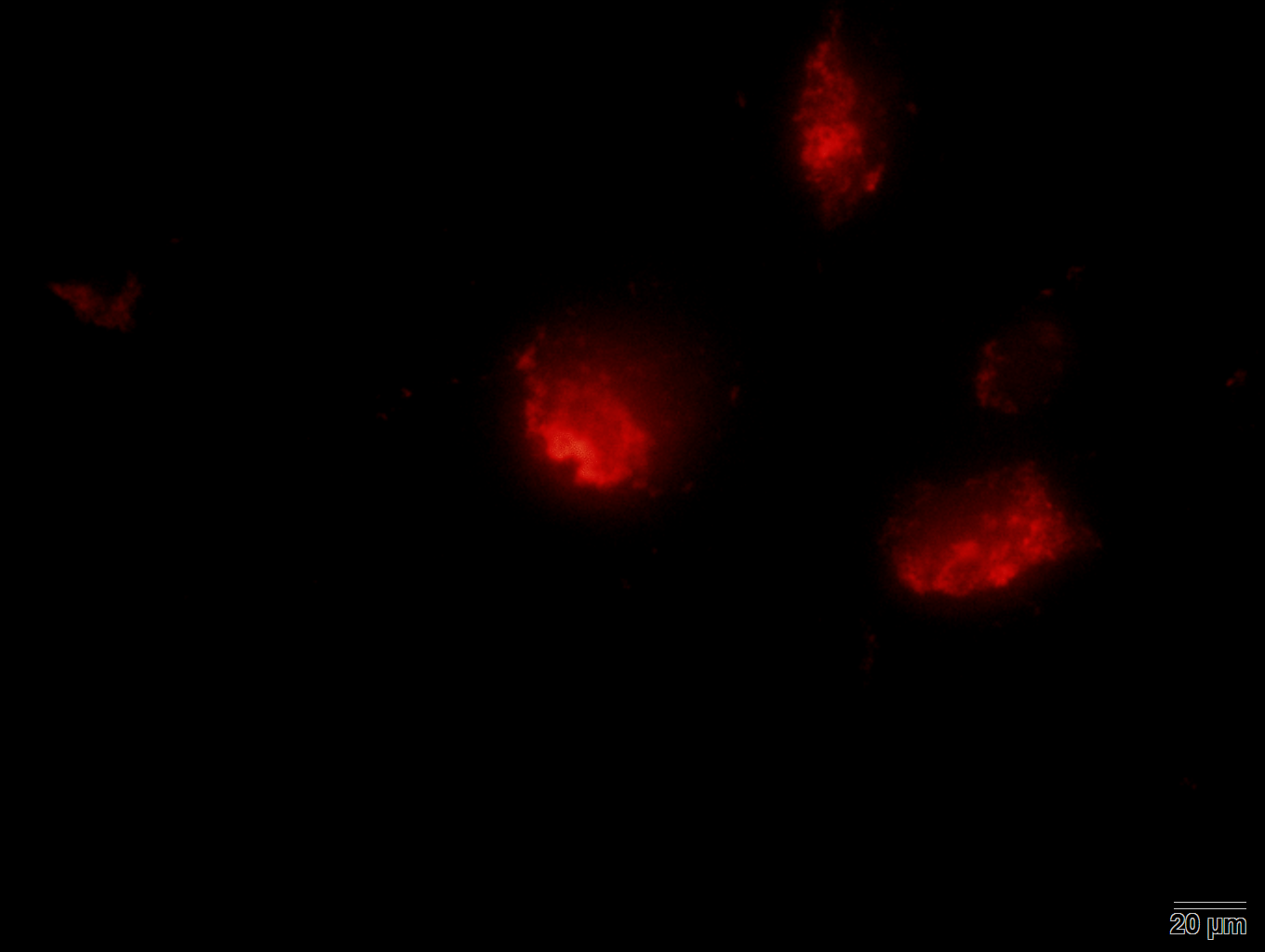


**Figure9 A: oeNC**


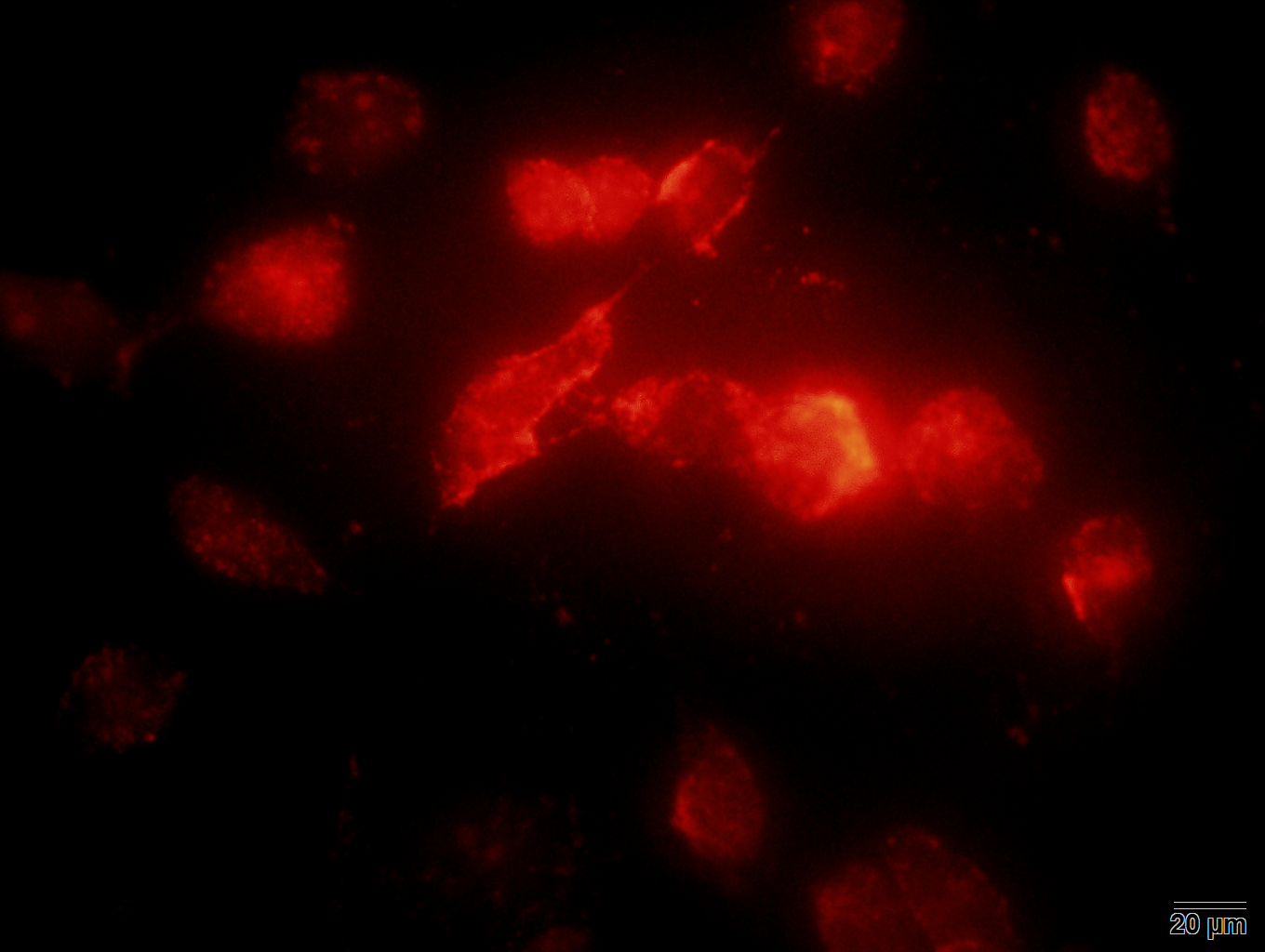


**Figure9 A: oeMCUB**


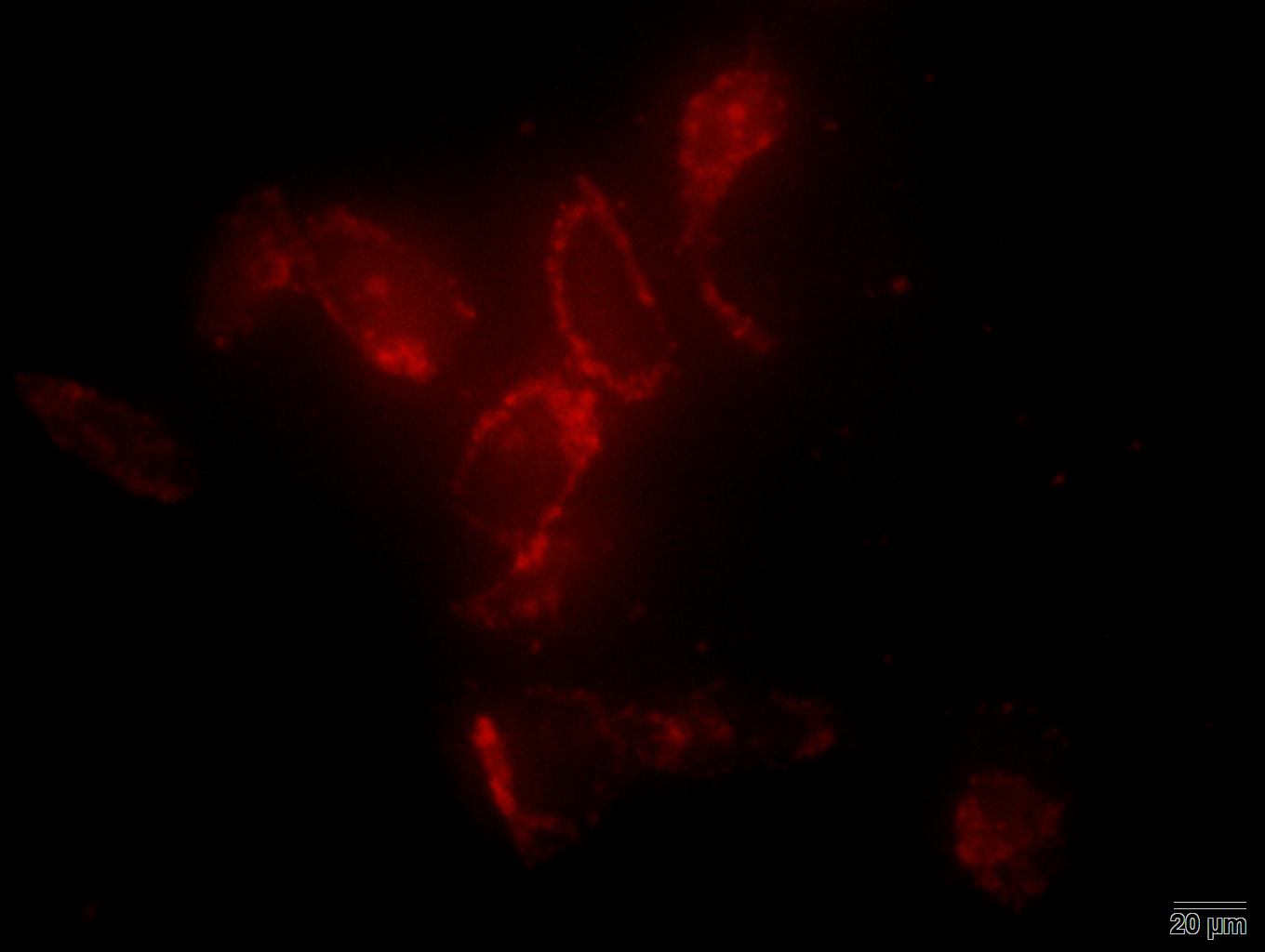


**Figure9 B:**

**Figure9 B: p-PRKN**

**Group: (1) NC (2) 4.0uM (3) 8.0uM (4) 12uM (5) 16uM (6) 20uM**


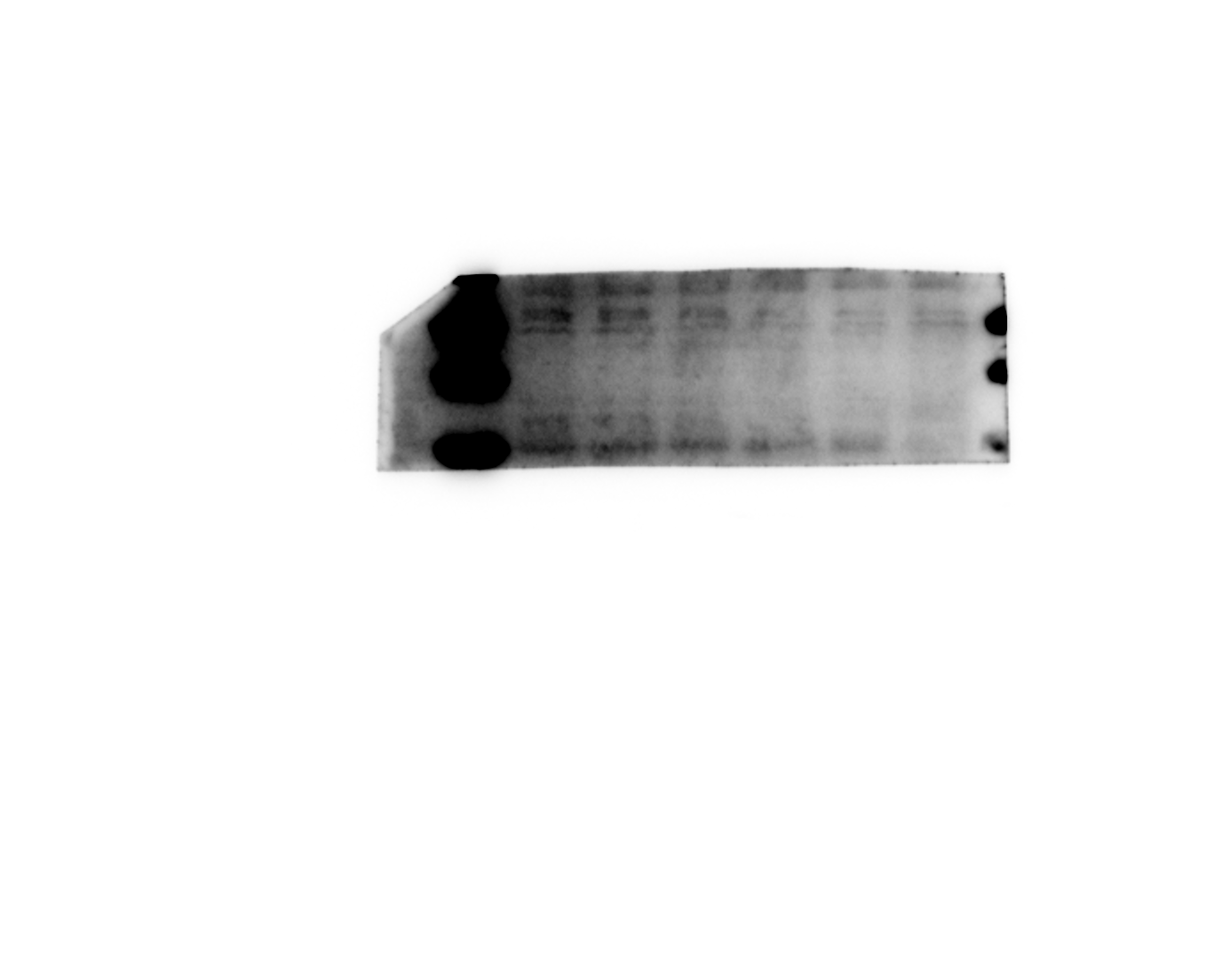


**Figure9 B: PRKN**

**Group: (1) NC (2) 4.0uM (3) 8.0uM (4) 12uM (5) 16uM**


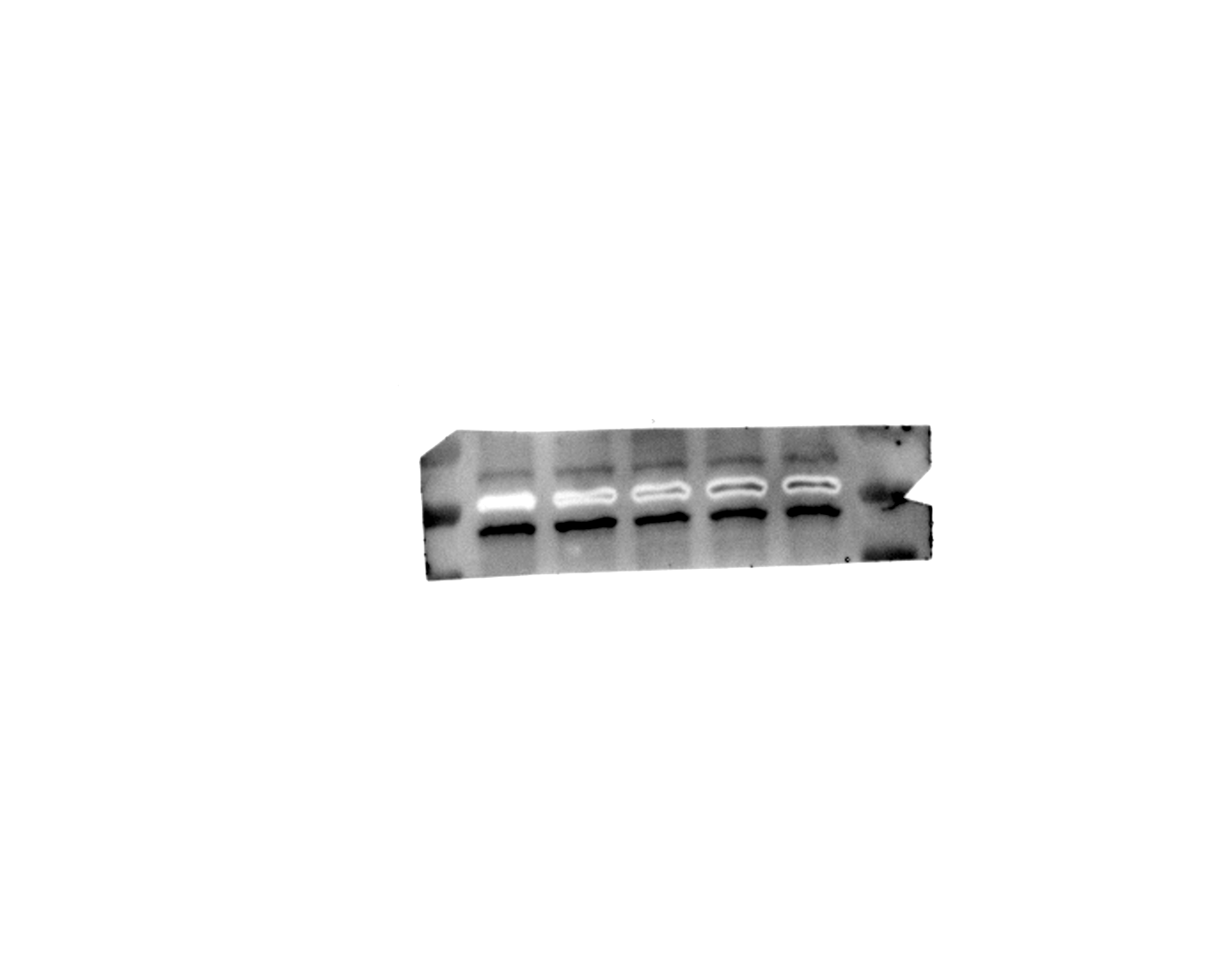


**Figure9 B: PD-L1**

**Group: (1) NC (2) 4.0uM (3) 8.0uM (4) 12uM (5) 16uM (6) 20uM**


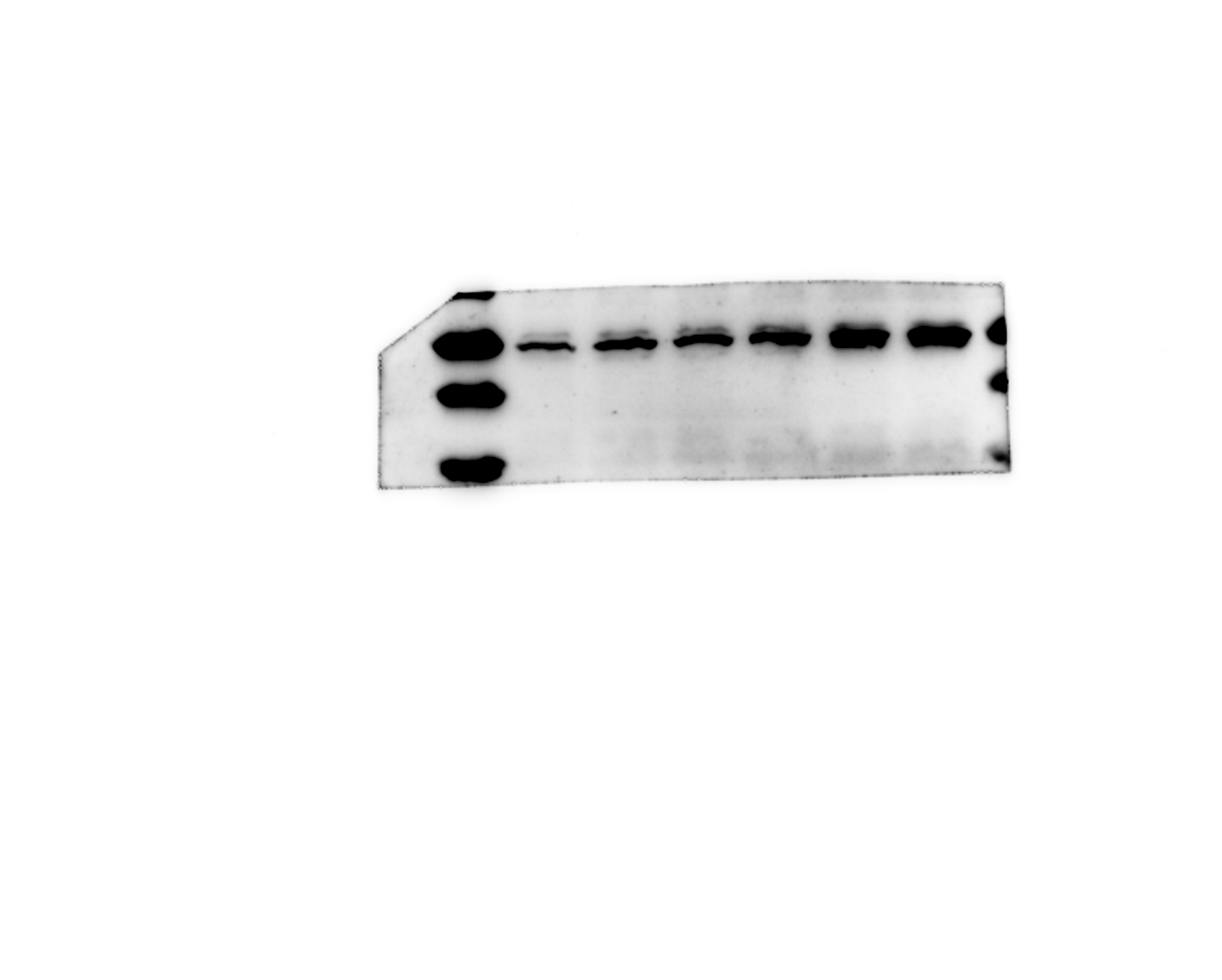


**Figure9 B: MCUB**

**Group: (1) NC (2) 4.0uM (3) 8.0uM (4) 12uM (5) 16uM (6) 20uM**


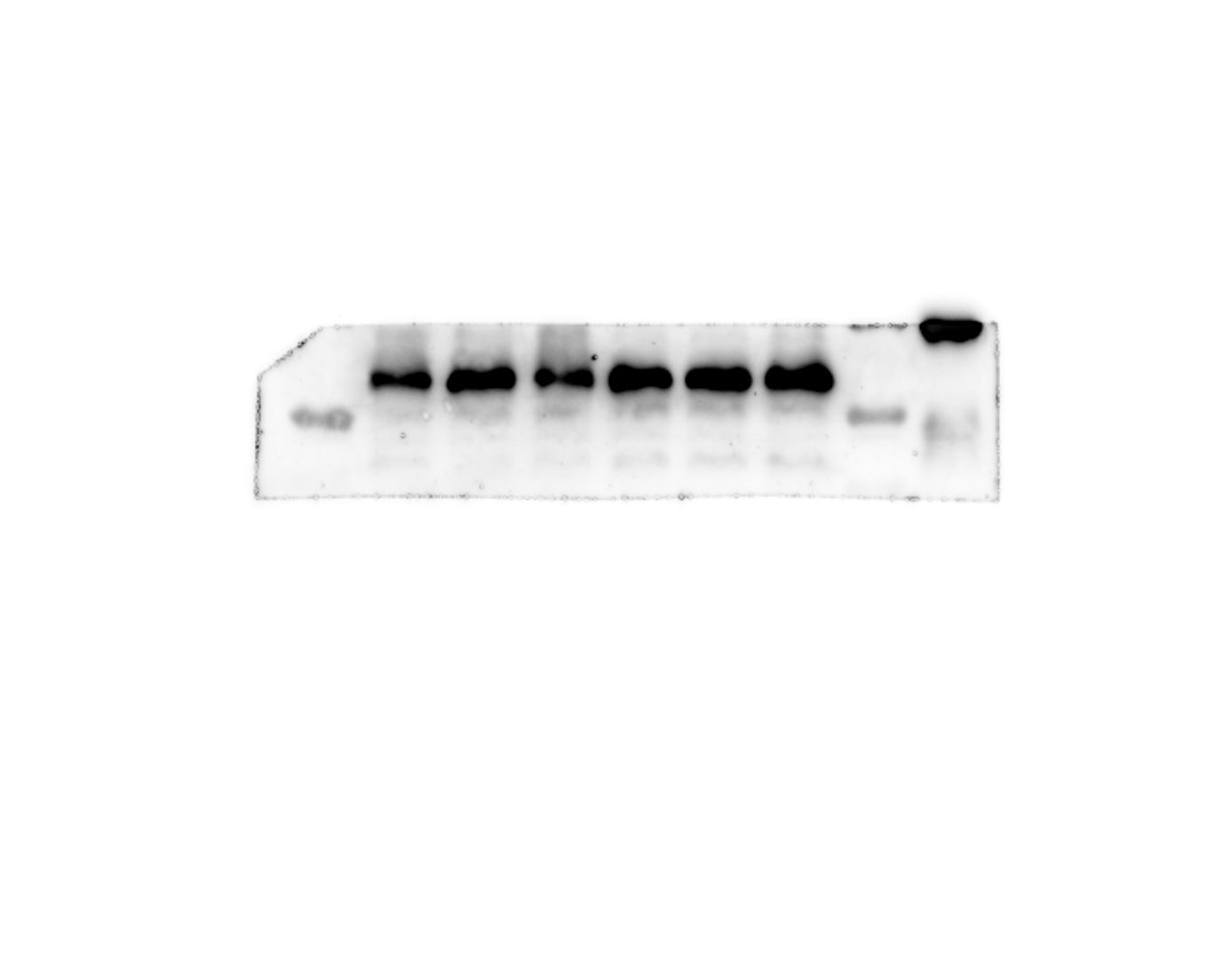


**Figure9 B: α-Tubulin**

**Group: (1) NC (2) 4.0uM (3) 8.0uM (4) 12uM (5) 16uM**


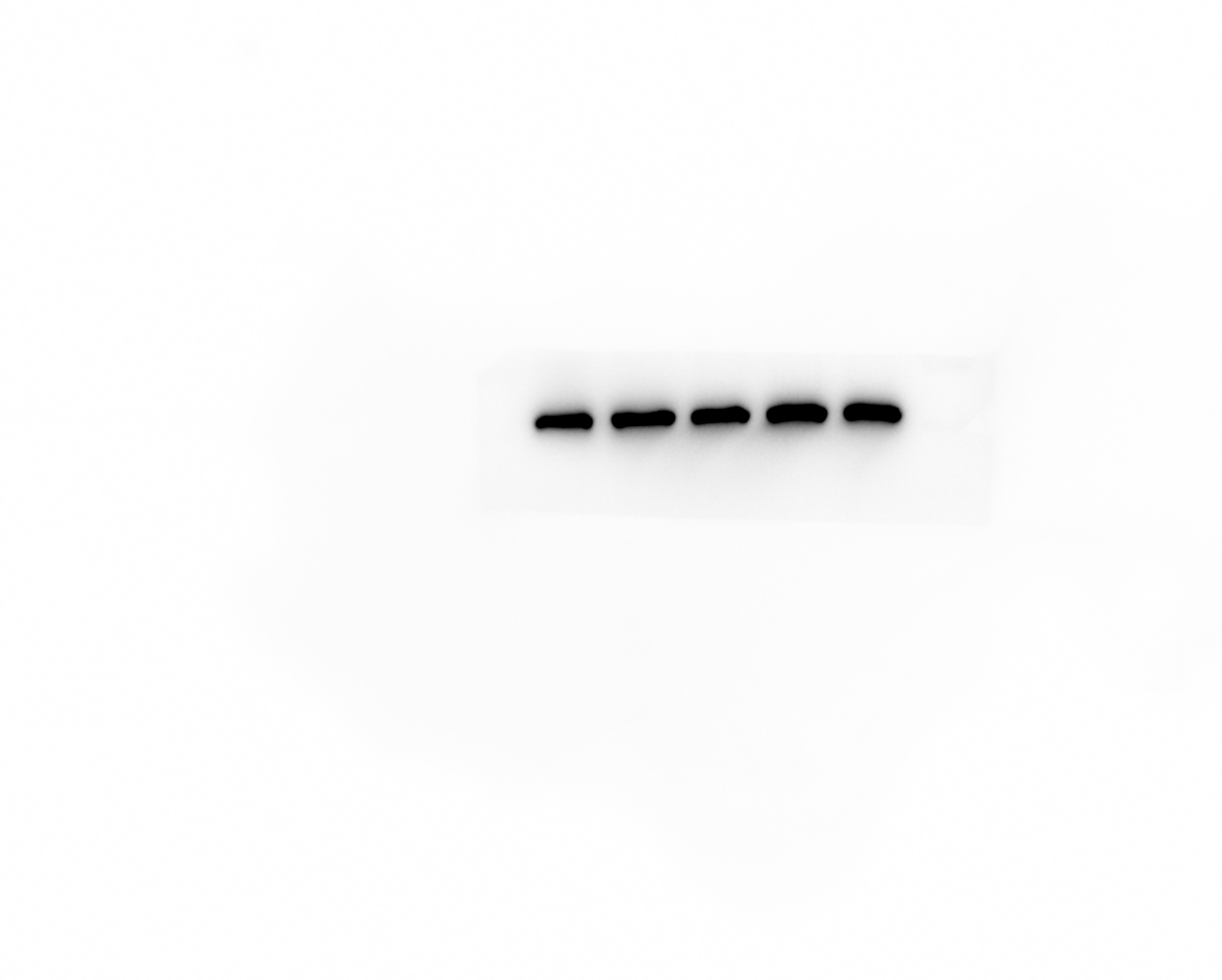

Supplement: Supplementary file 2 — Supporting Information [file ADVS-13-e14764-s002.zip › Figure9.docx]
