# Supplementary material for: MCUB Inhibits PRKN‐Dependent Mitophagic Degradation of PD‐L1 to Promote Immune Evasion in Bladder Cancer
Source: Adv Sci (Weinh). 2025 Nov 12;13(5):e14764. doi: 10.1002/advs.202514764 (PMC12849890; doi:10.1002/advs.202514764)
Supplement: Supplementary file 2 — Supporting Information [file ADVS-13-e14764-s002.zip › Figure10.docx]

**Figure10:**


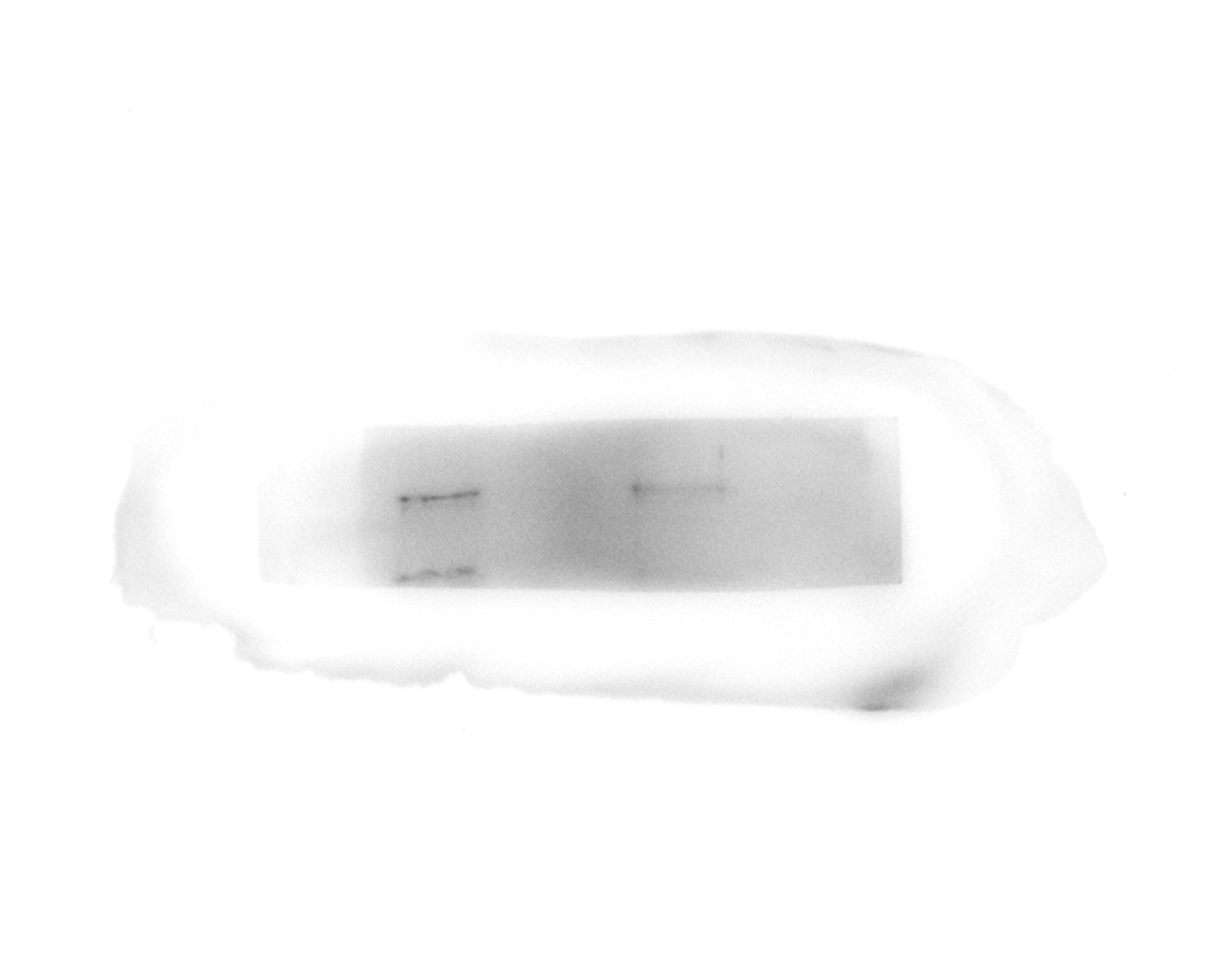
**Figure10 A: PRKN**


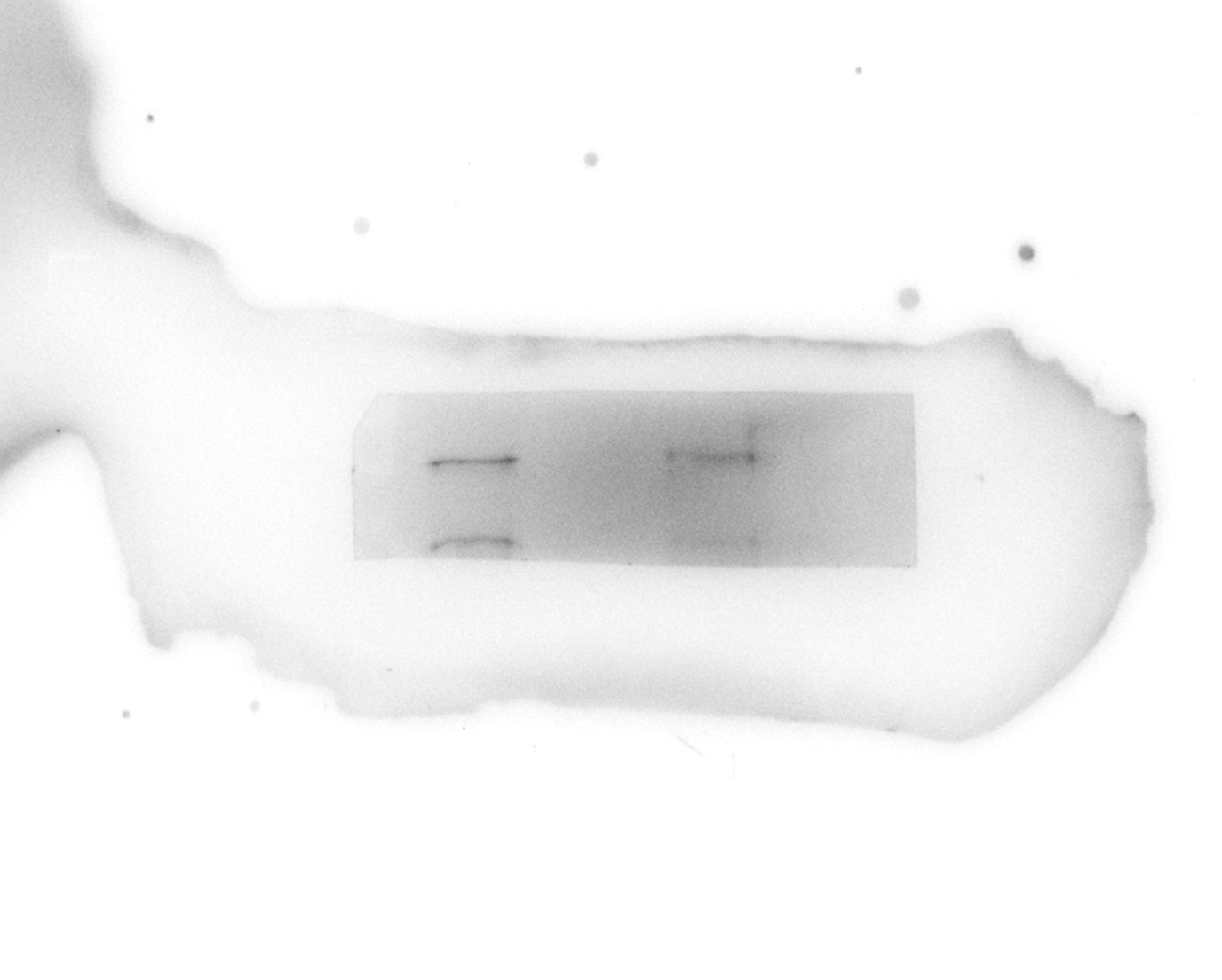
 **Figure10 A: MCUB**


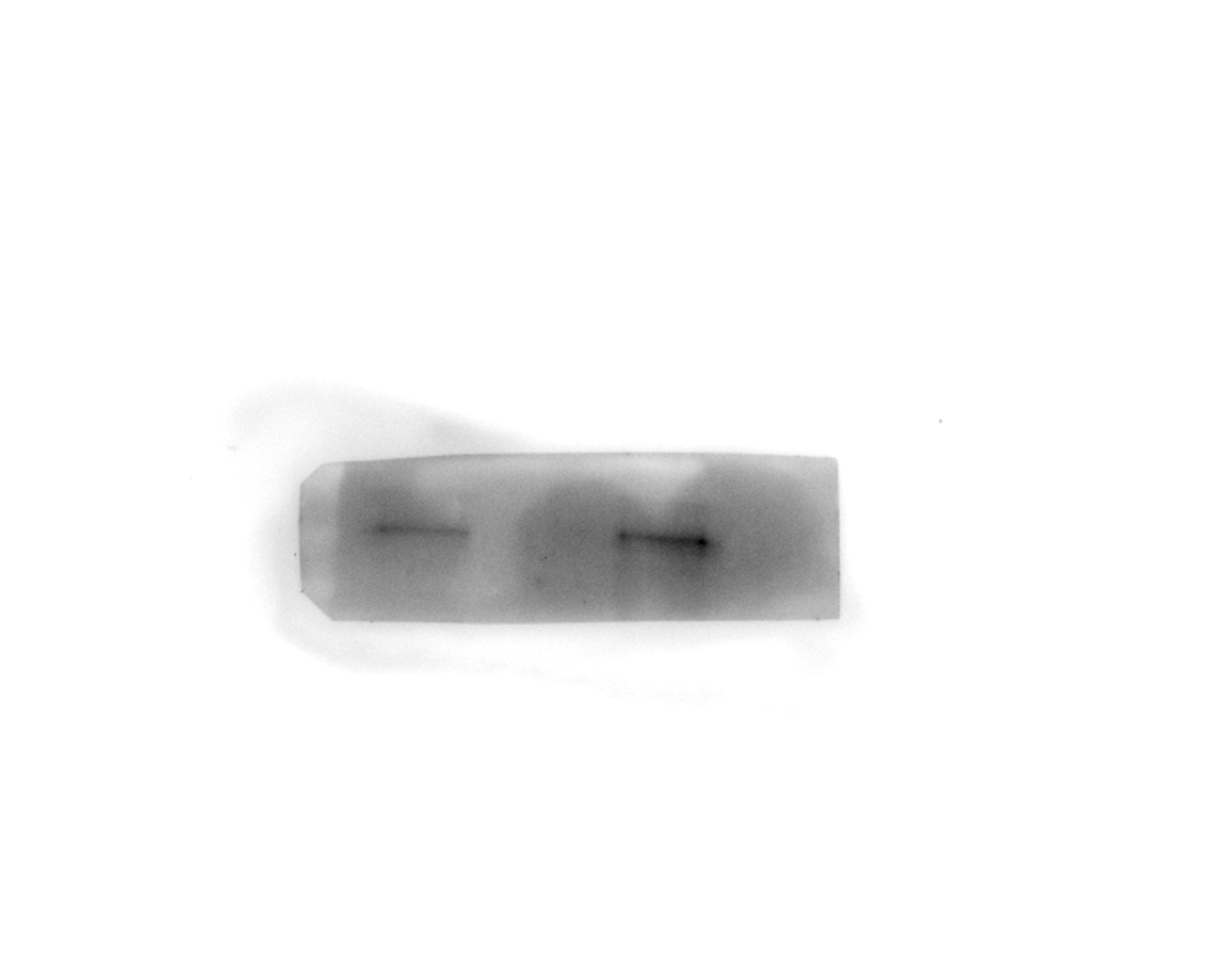
 **Figure10 A: MCUB**


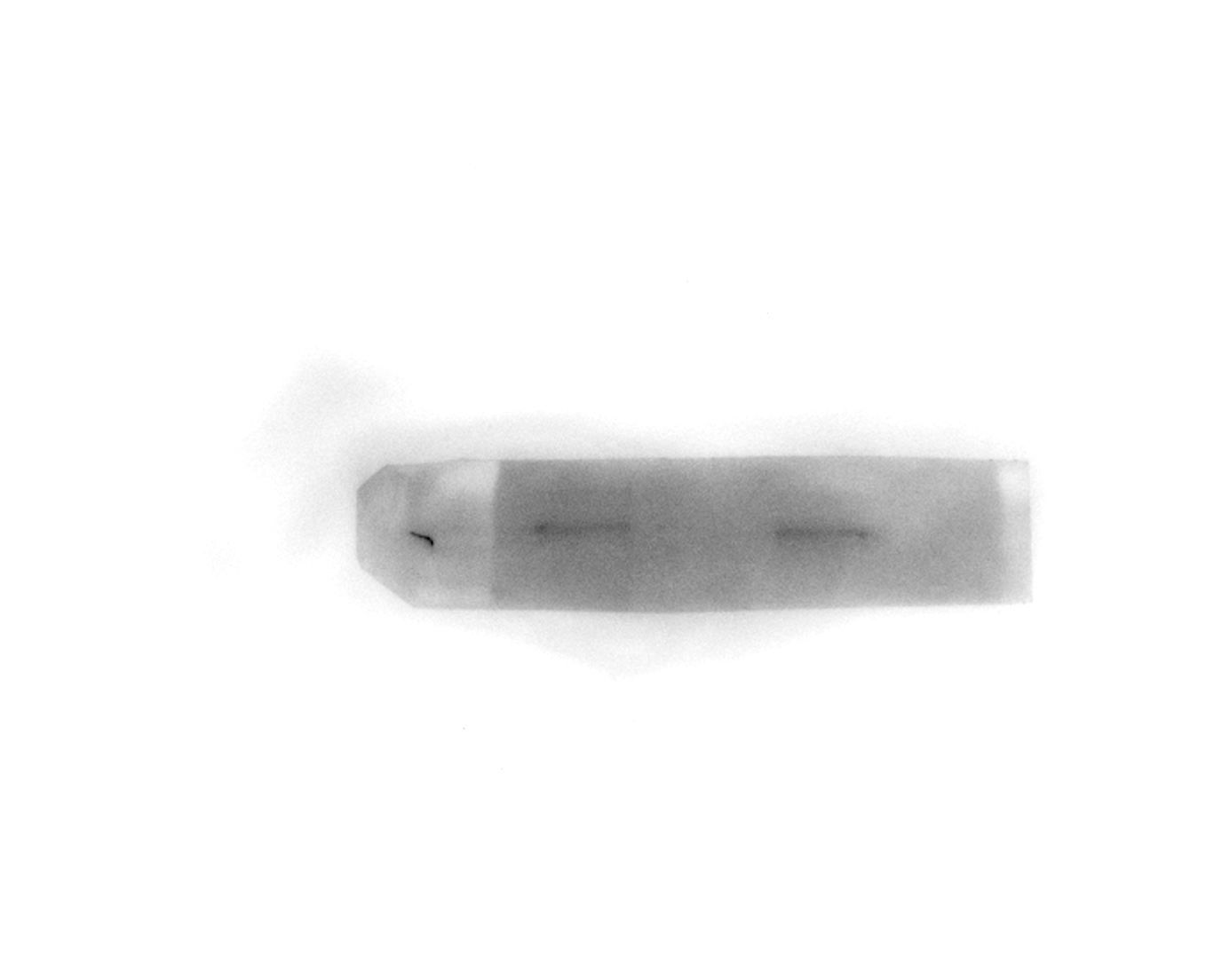
 **Figure10 A: PRKN**


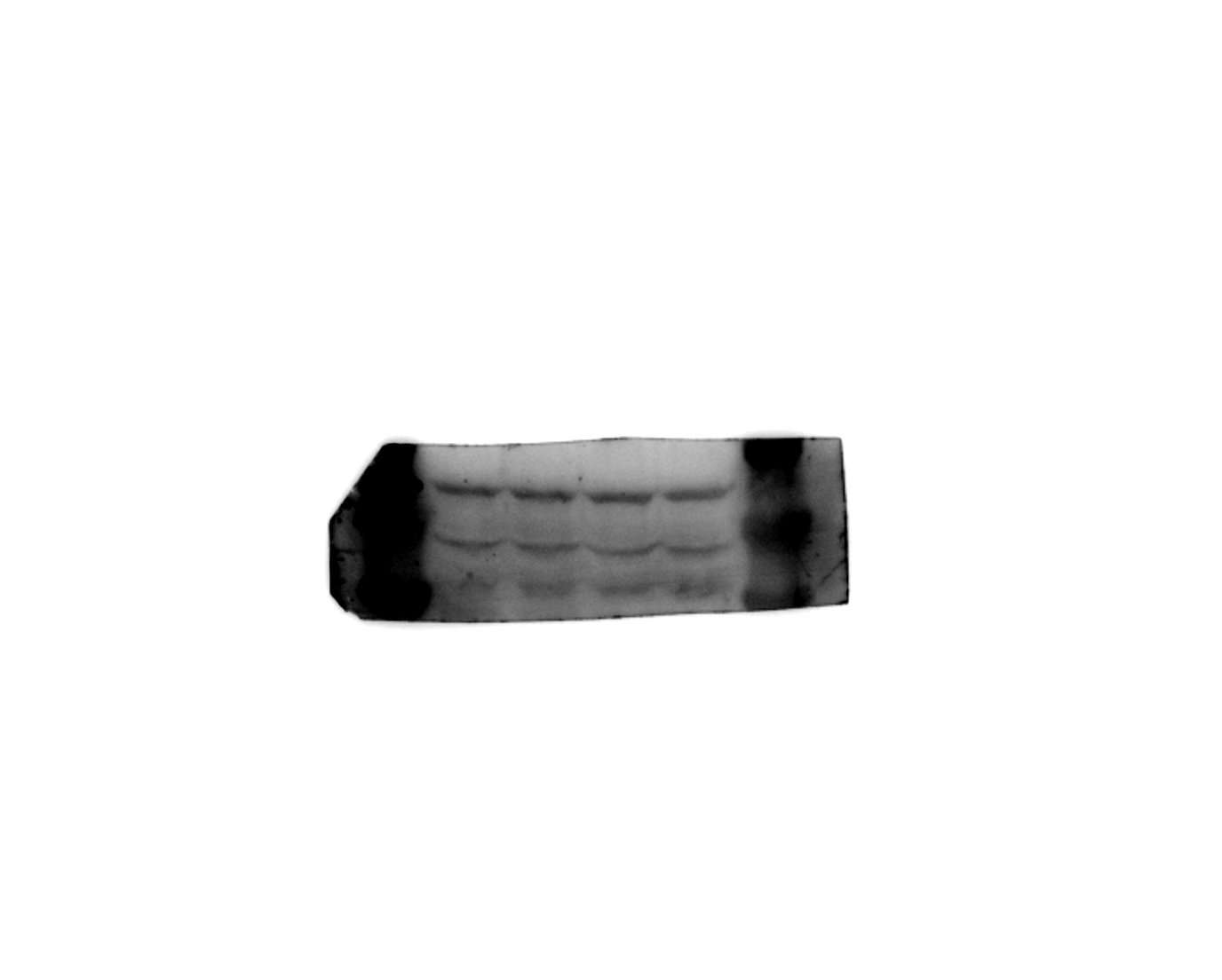
 **Figure10 C, Input: PD-L1**


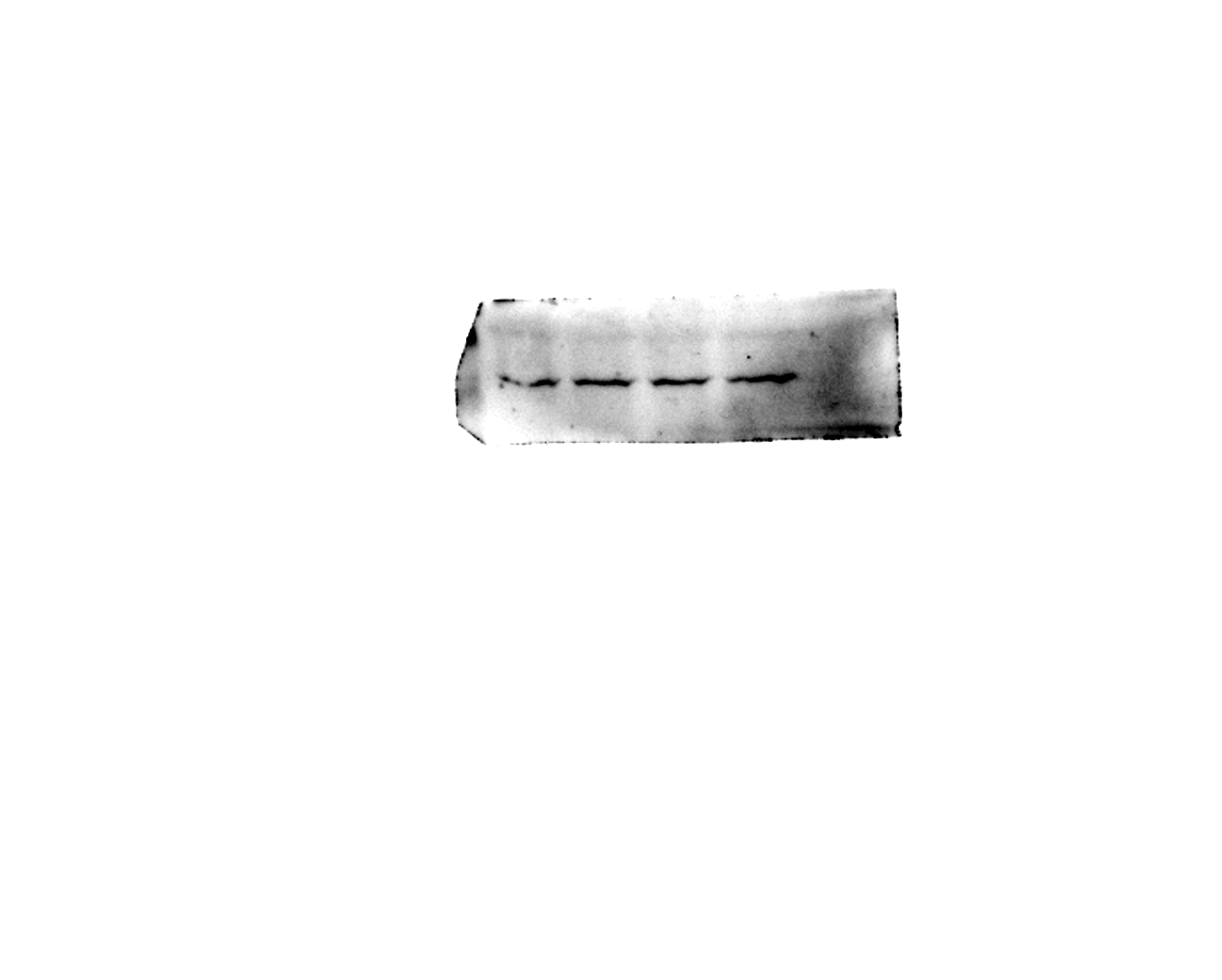
 **Figure10 C, Input: PINK1**


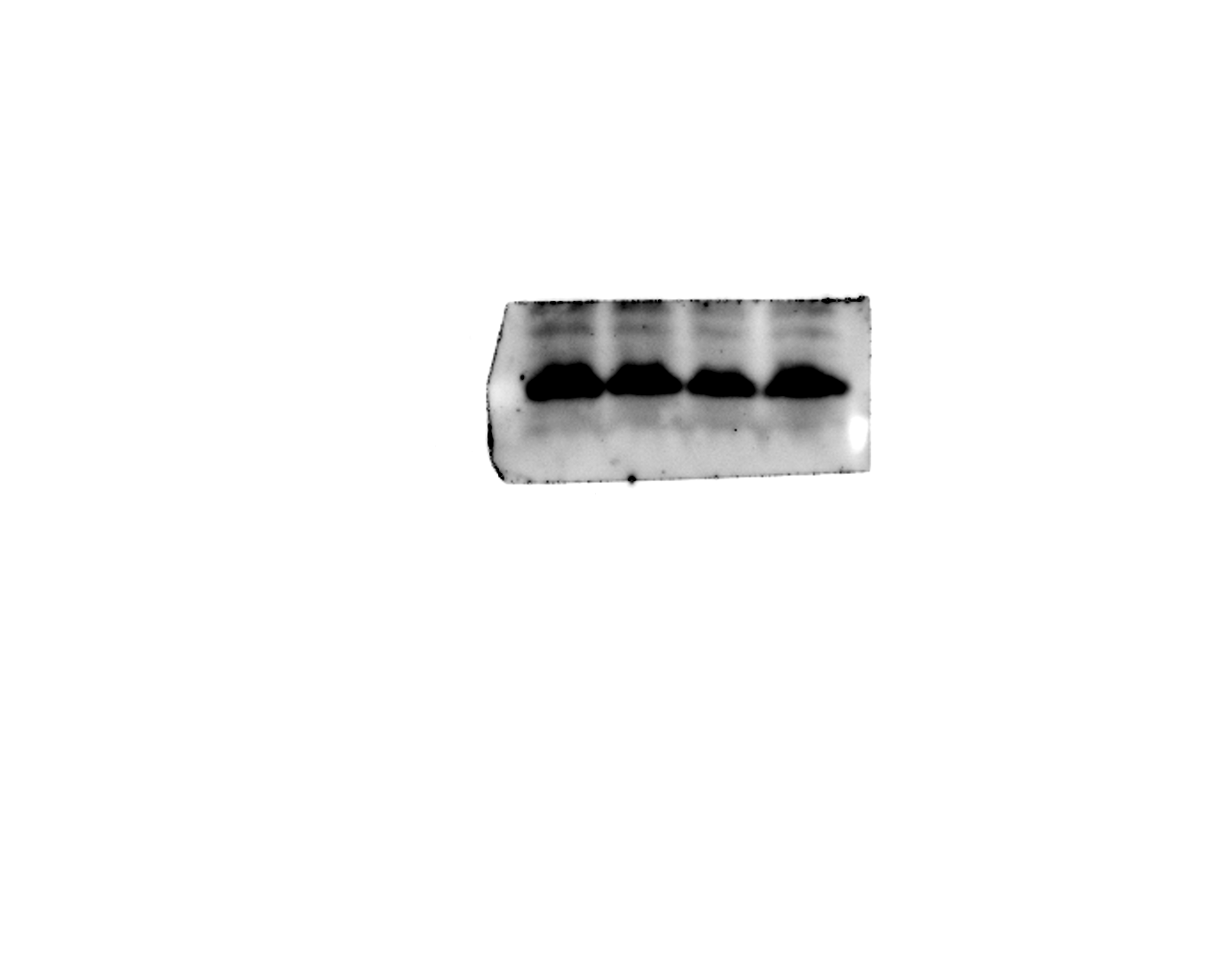
 **Figure10 C, Input: LC3**


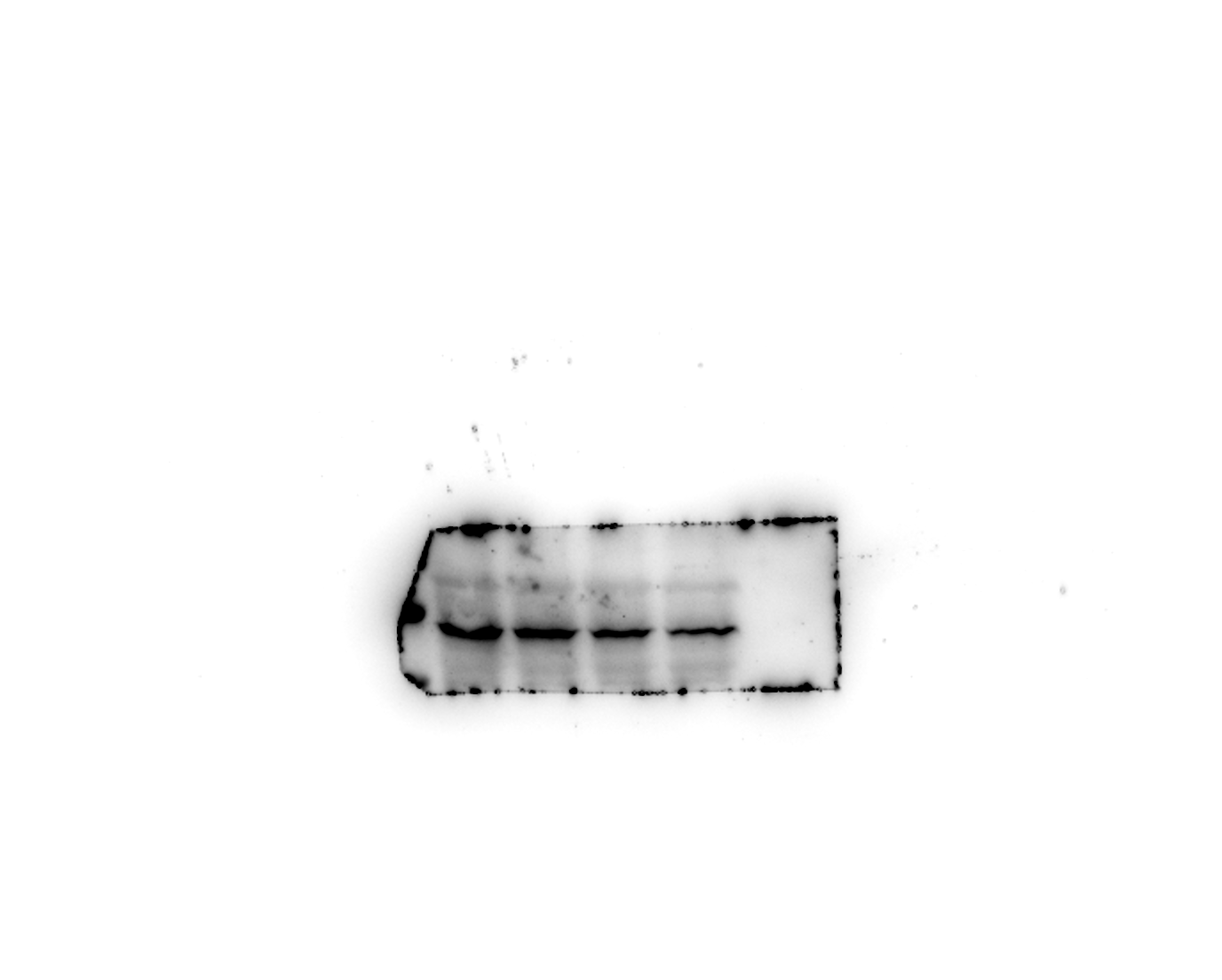
 **Figure10 C, Input: p-PPRKN**


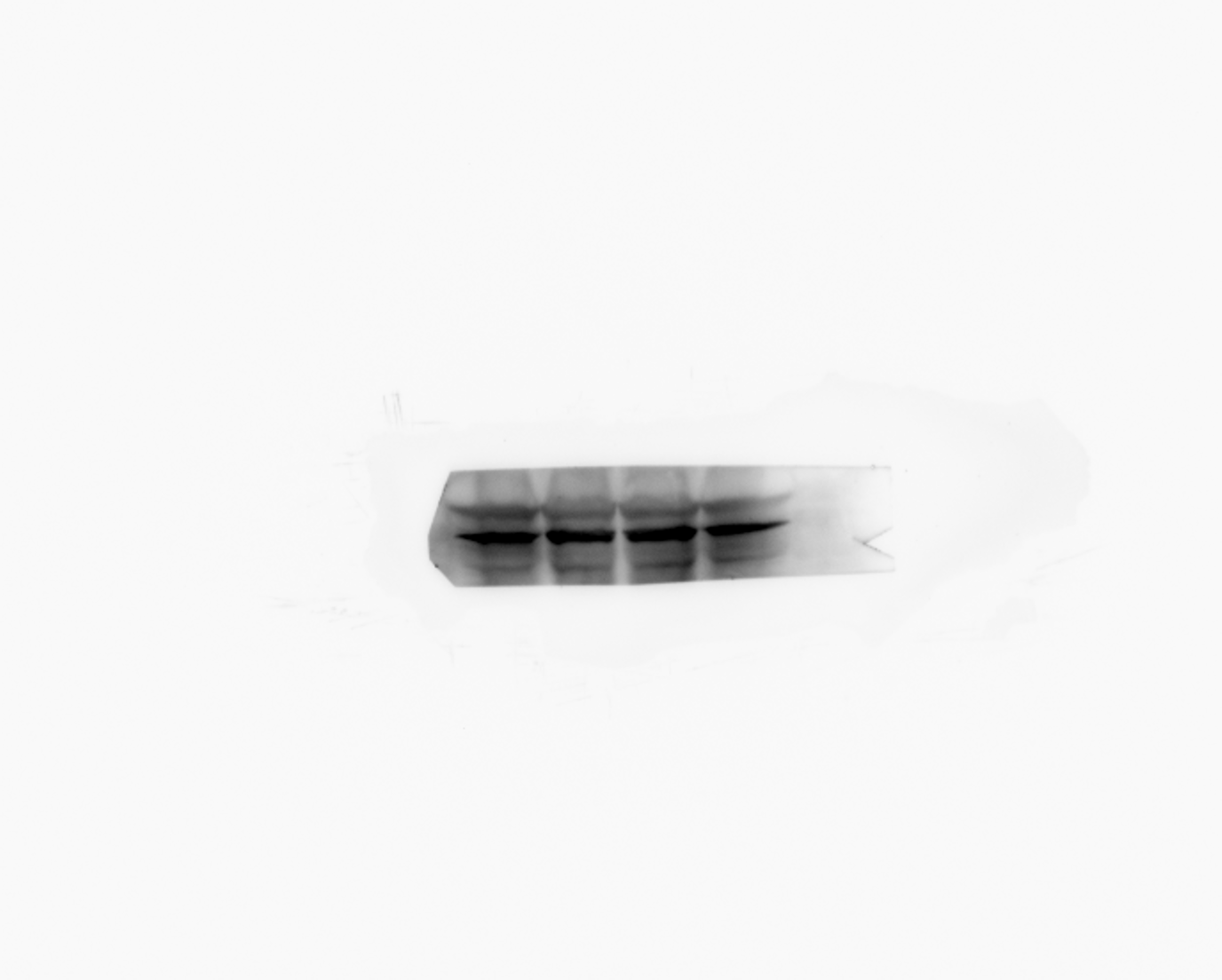
 **Figure10 C, Input: PRKN**


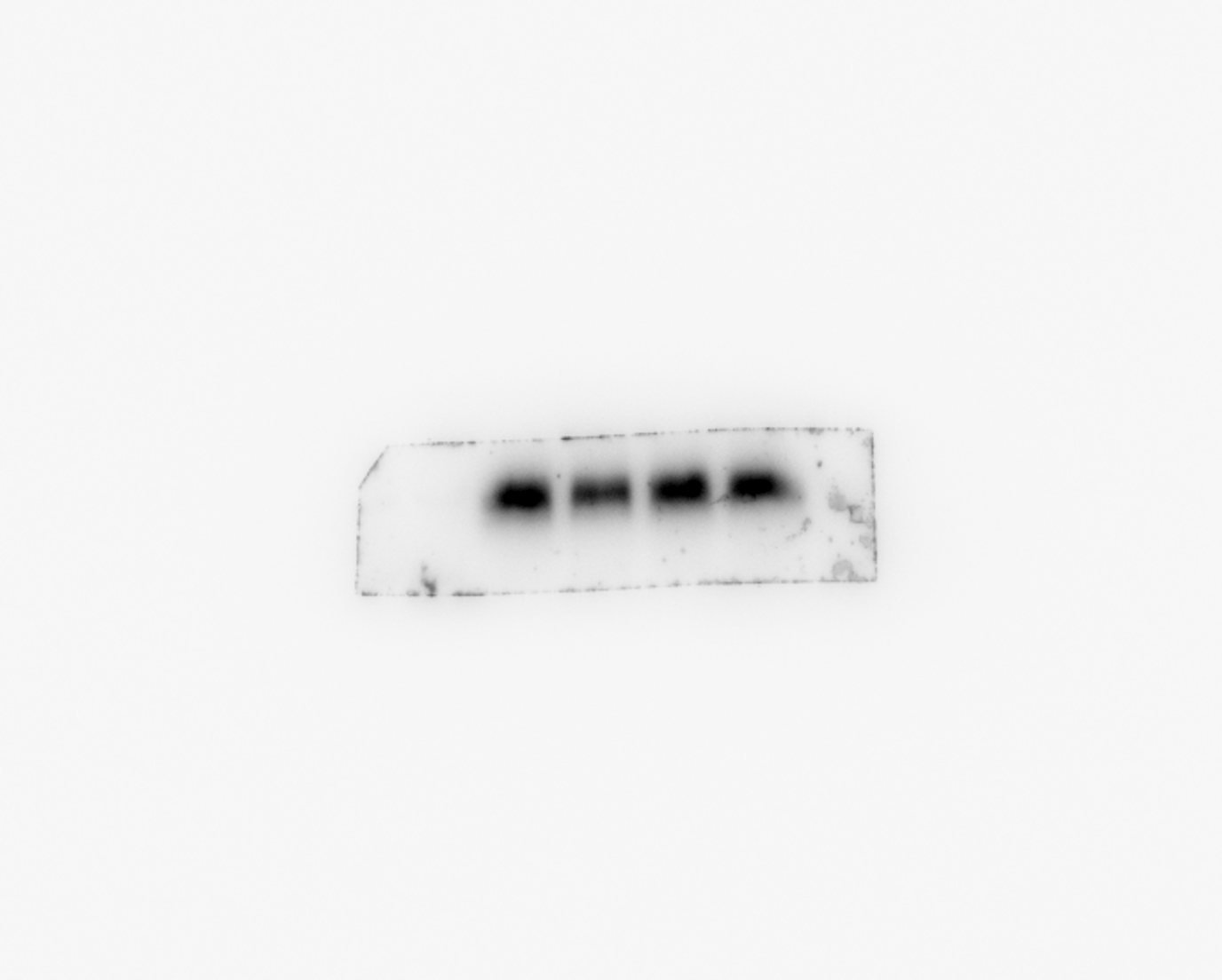
 **Figure10 C, IP: PRKN**


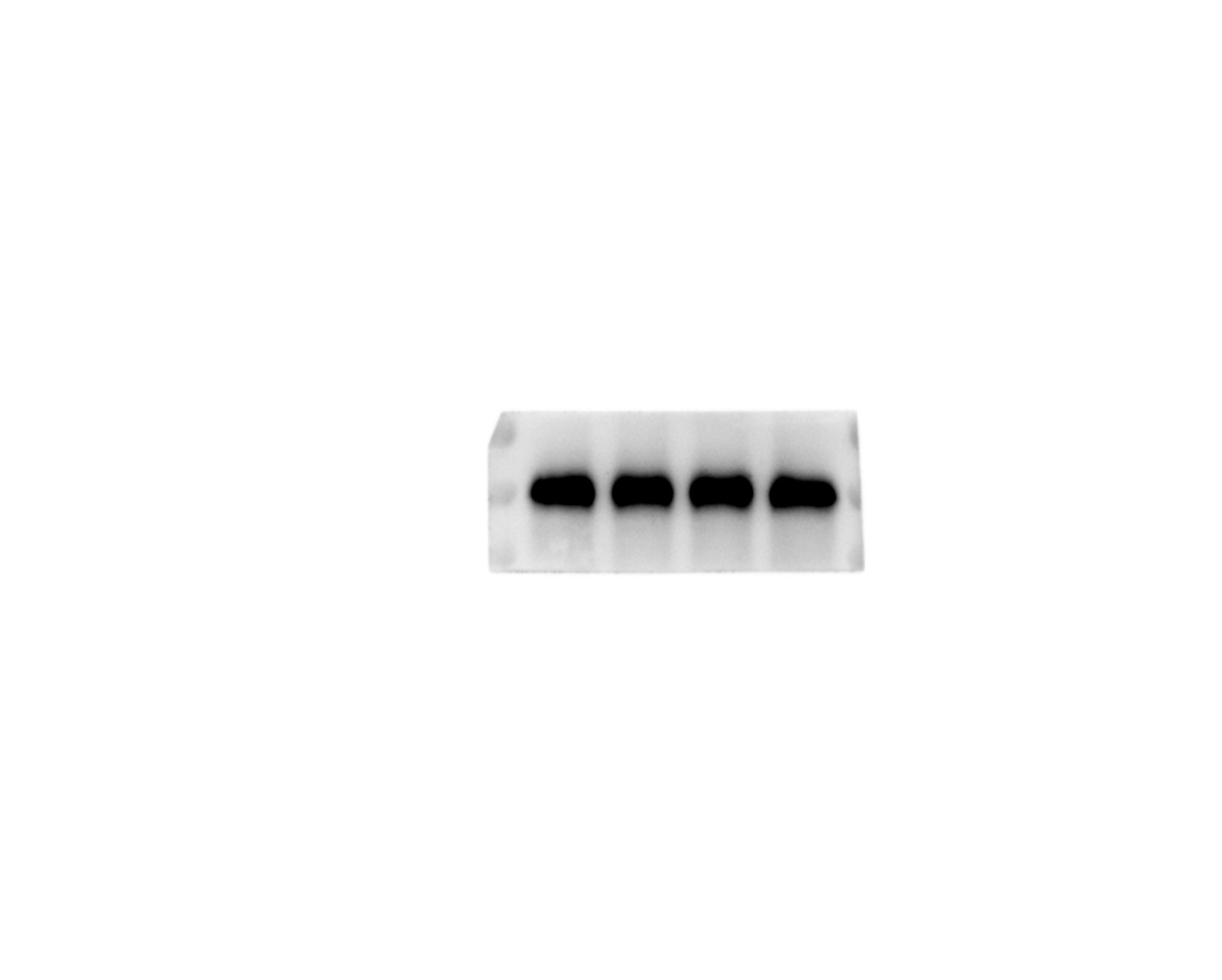
 **Figure10 C, IP: MCUB**


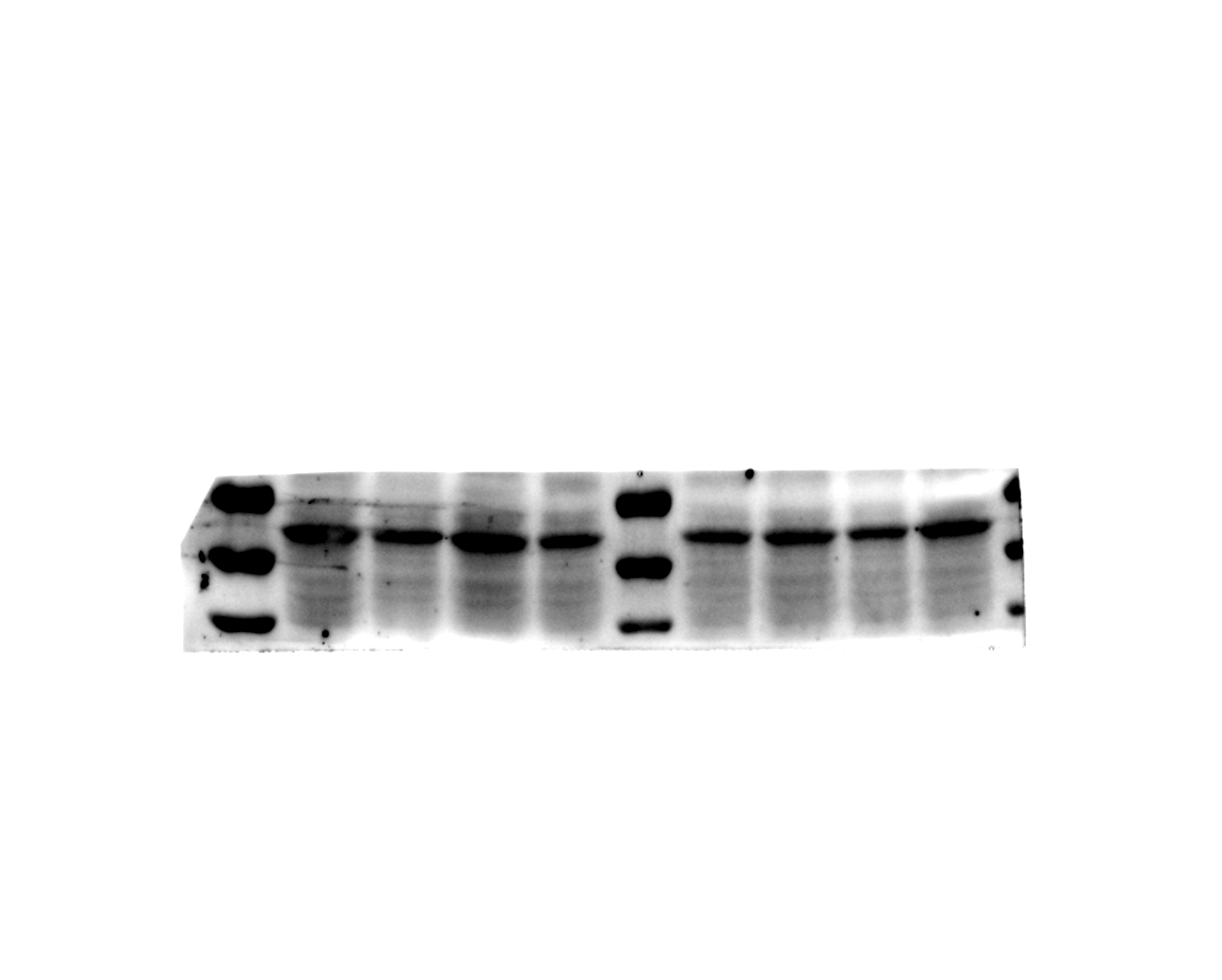
 **Figure10 D, E: MCUB**


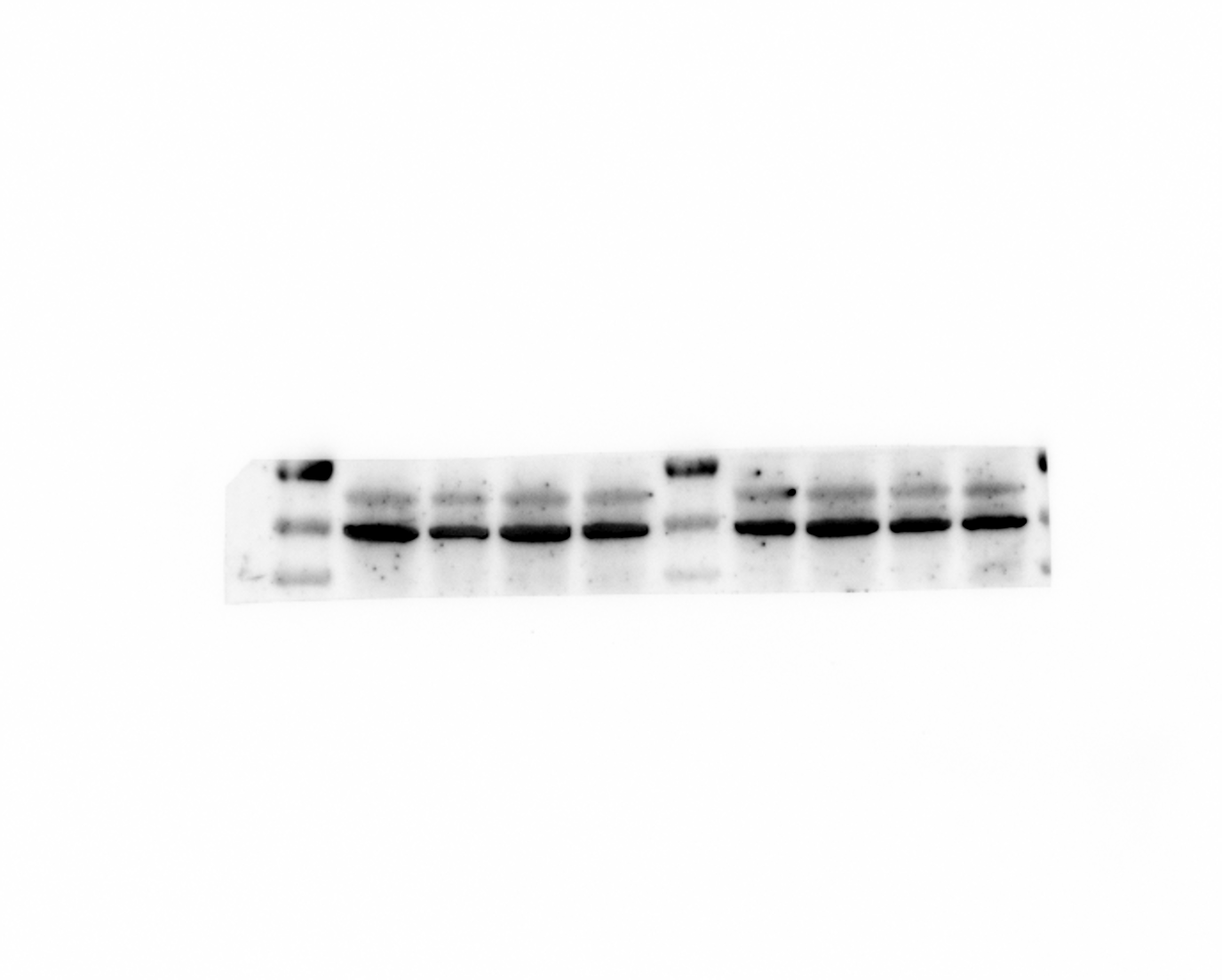
 **Figure10 D, E: PD-L1**


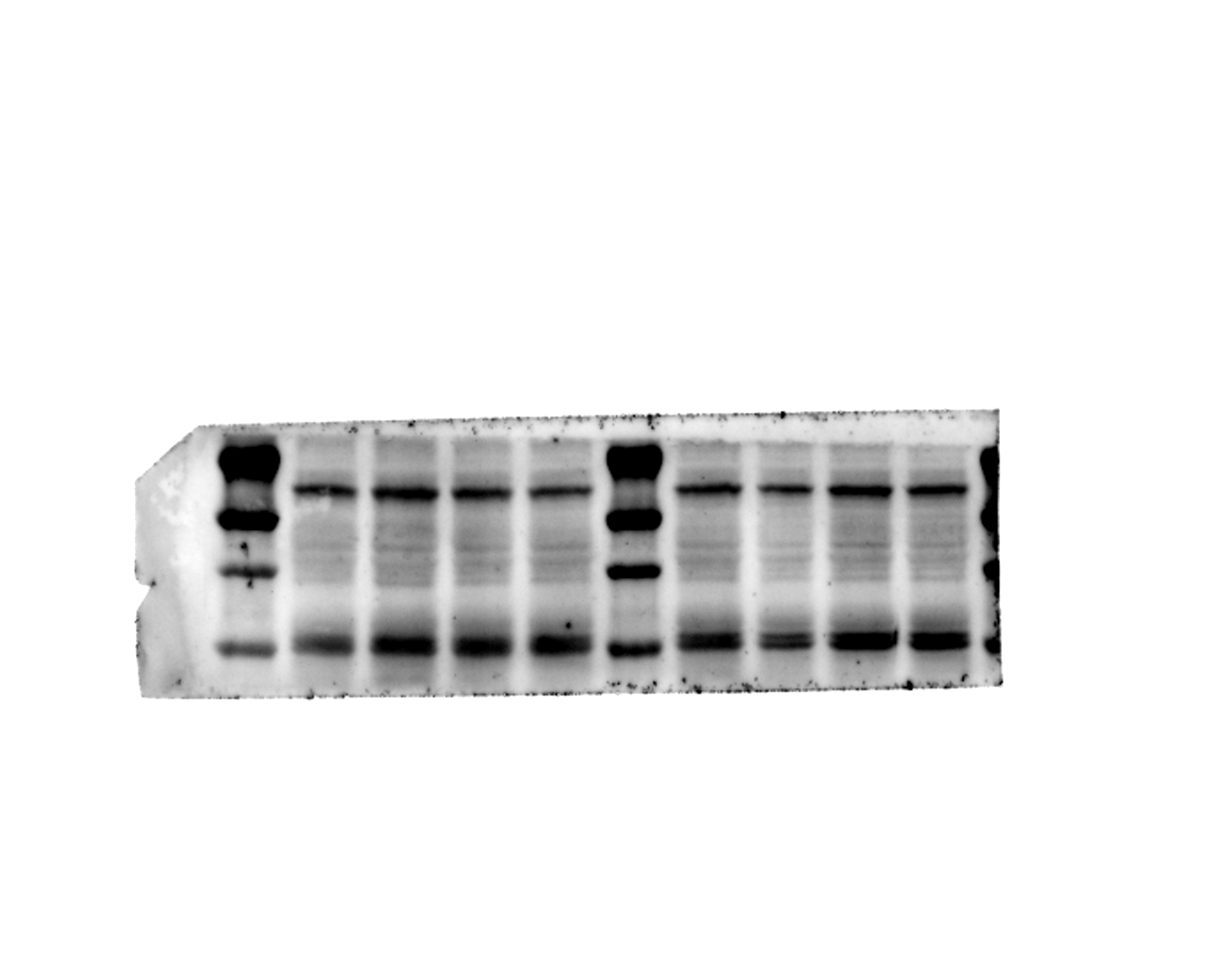
 **Figure10 D, E: p-PRKN**


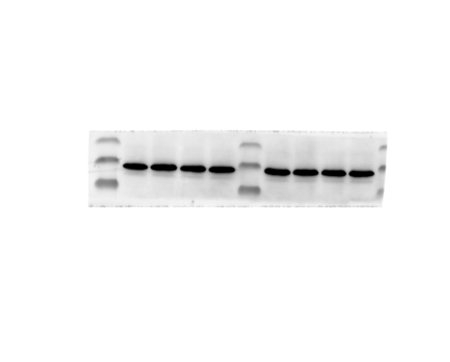
**Figure10 D, E: α-Tubulin**
