# Supplementary material for: MCUB Inhibits PRKN‐Dependent Mitophagic Degradation of PD‐L1 to Promote Immune Evasion in Bladder Cancer
Source: Adv Sci (Weinh). 2025 Nov 12;13(5):e14764. doi: 10.1002/advs.202514764 (PMC12849890; doi:10.1002/advs.202514764)
Supplement: Supplementary file 2 — Supporting Information [file ADVS-13-e14764-s002.zip › Figure11 C.pdf]

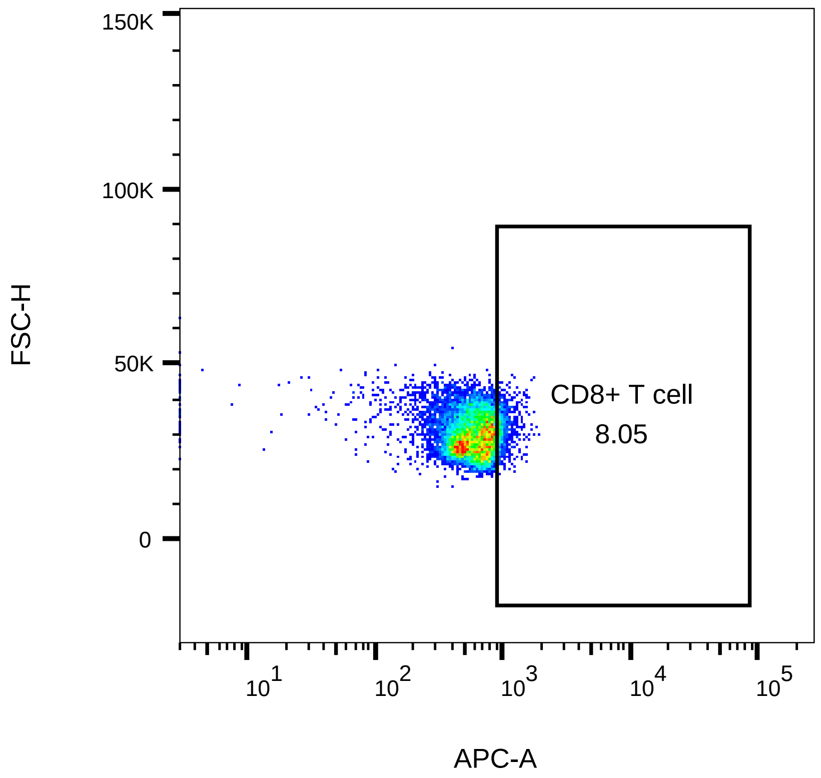

Specimen_001_wboty_003_004.fcs
Single Cells
12229

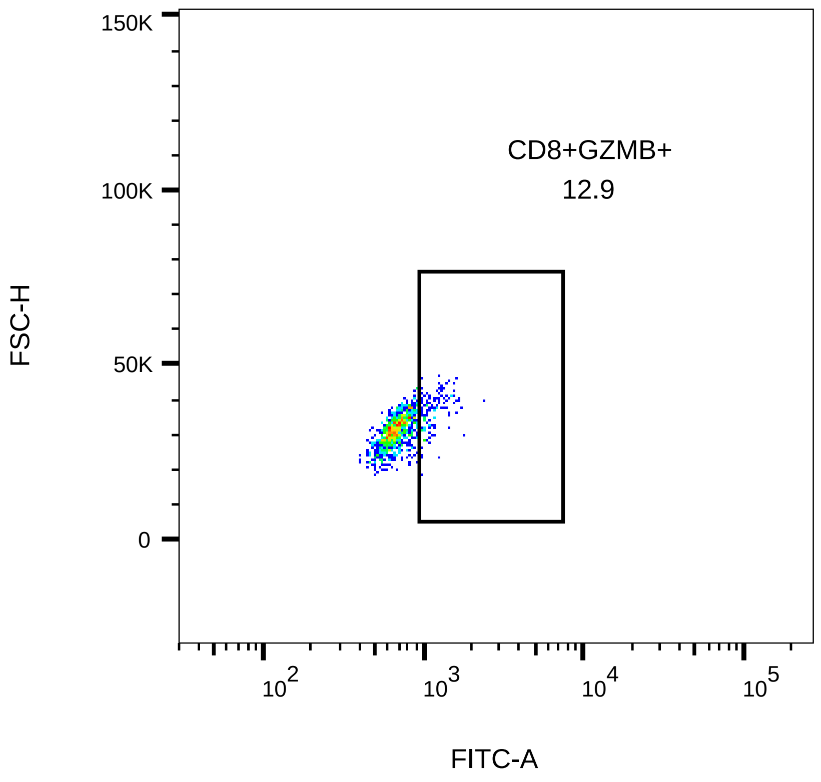

Specimen_001_wboty_003_004.fcs
CD8+ T cell
984

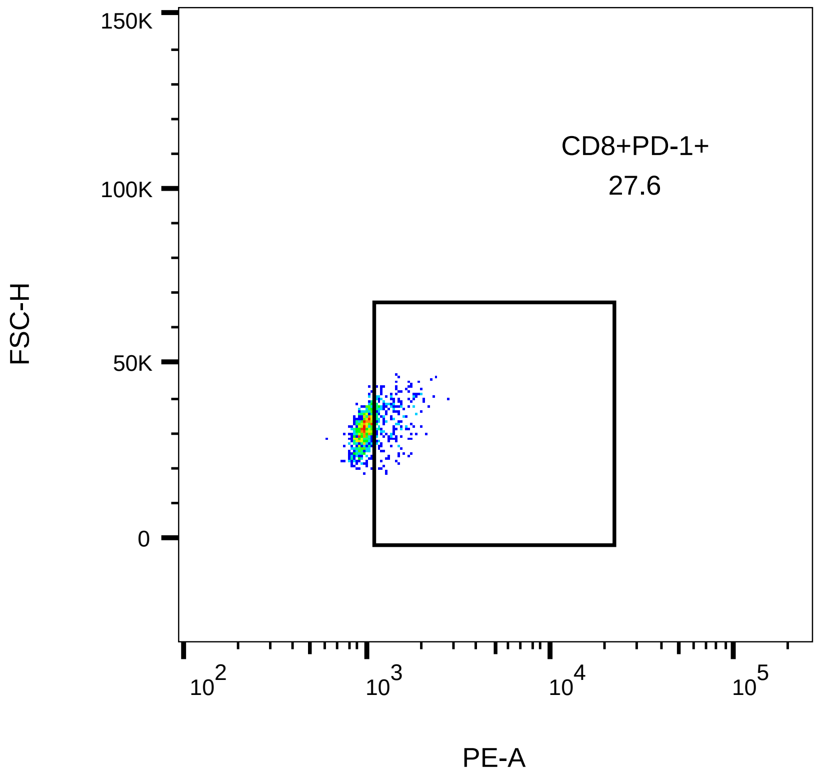

Specimen_001_wboty_003_004.fcs
CD8+ T cell
984

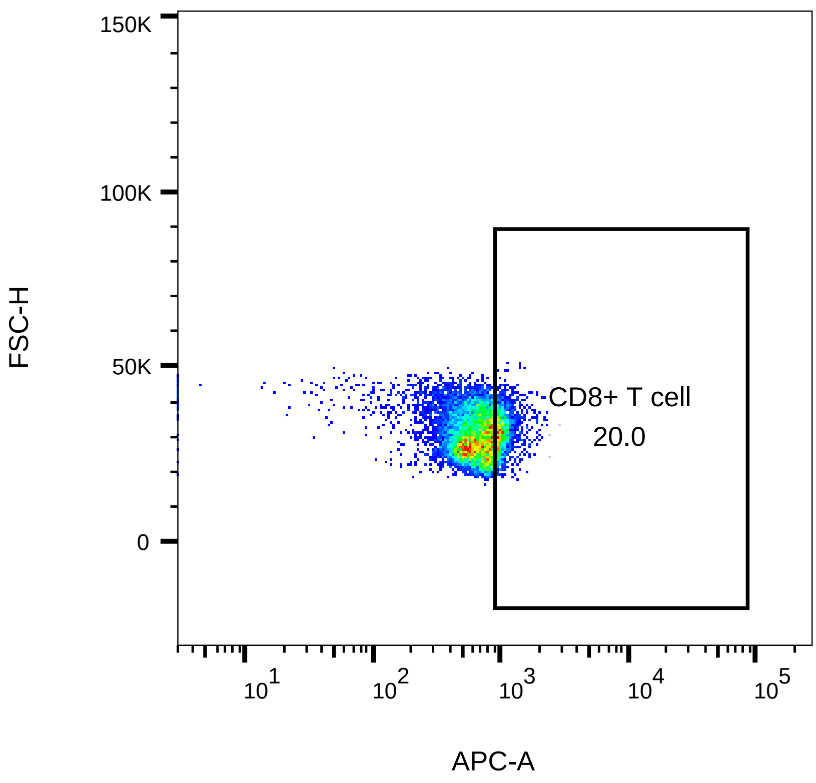

Specimen_001_wboty_007_008.fcs
Single Cells
12047

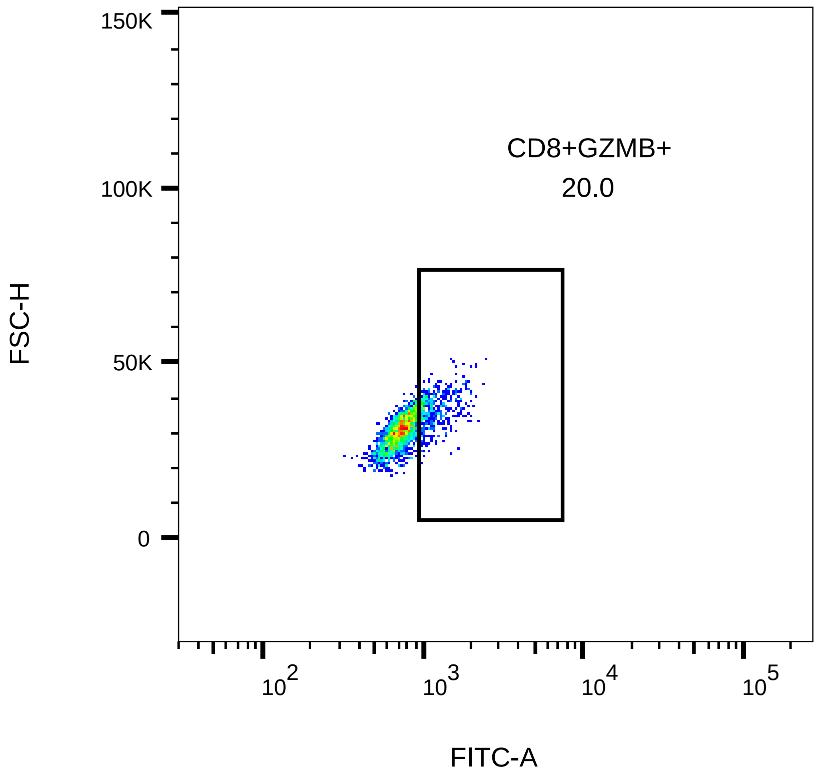

Specimen_001_wboty_007_008.fcs
CD8+ T cell
2408

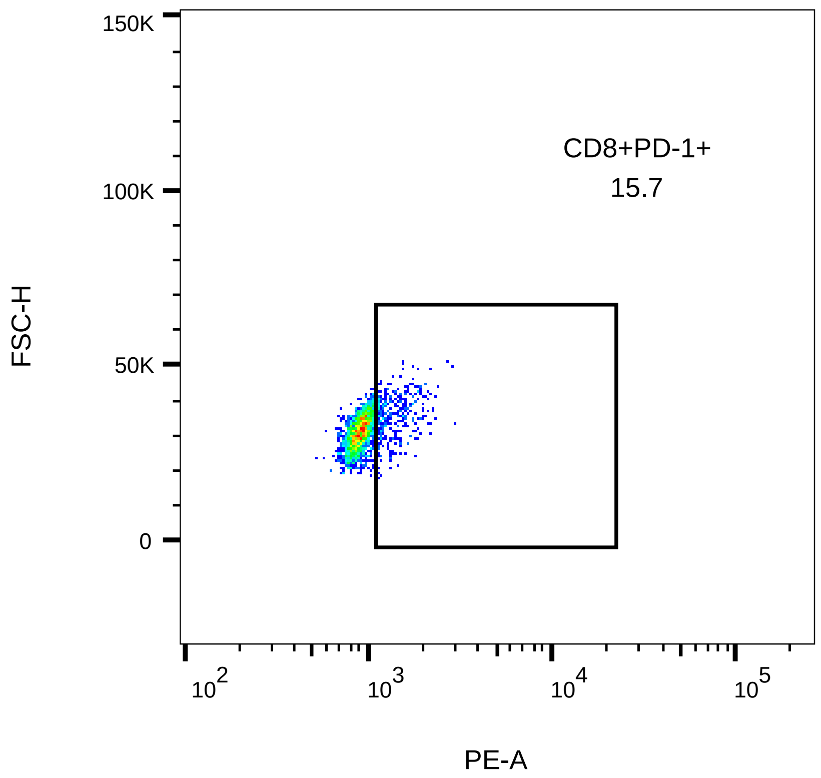

Specimen_001_wboty_007_008.fcs
CD8+ T cell
2408

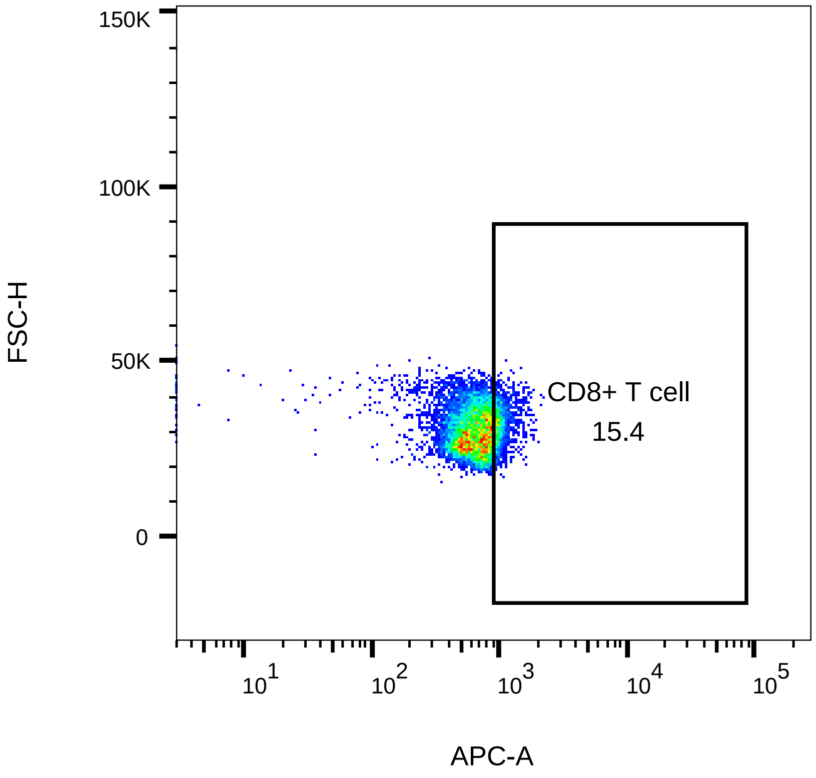

Specimen_001_wboty_014_015.fcs
Single Cells
12122

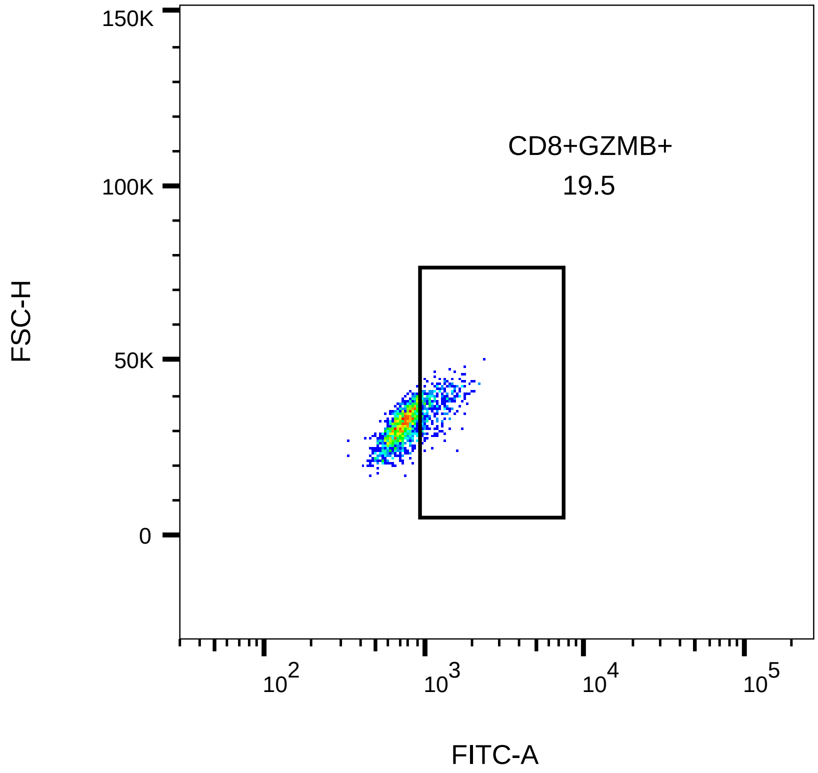

Specimen_001_wboty_014_015.fcs
CD8+ T cell
1870

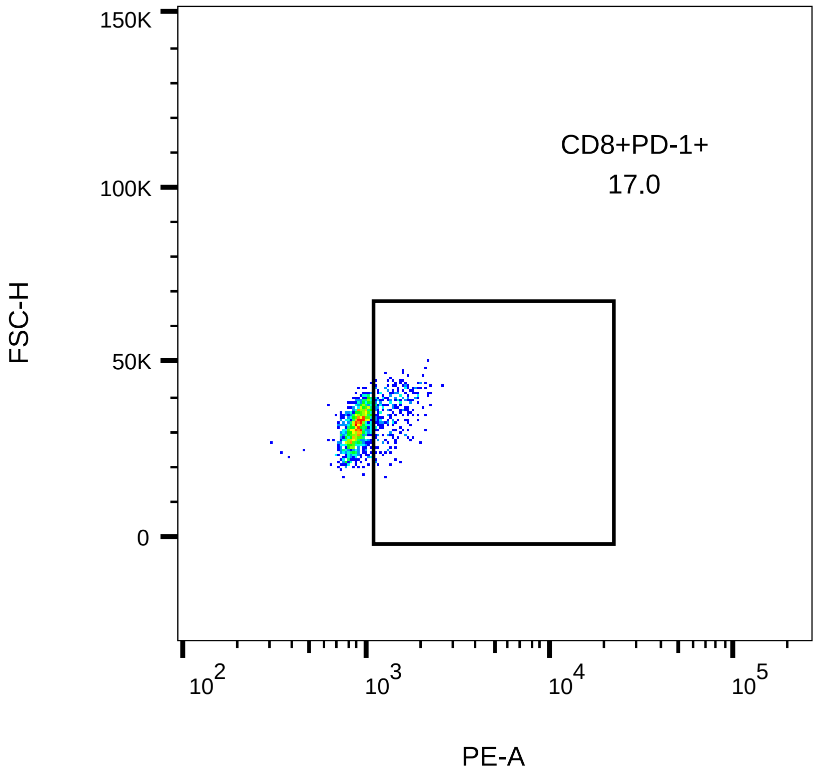

Specimen_001_wboty_014_015.fcs
CD8+ T cell
1870

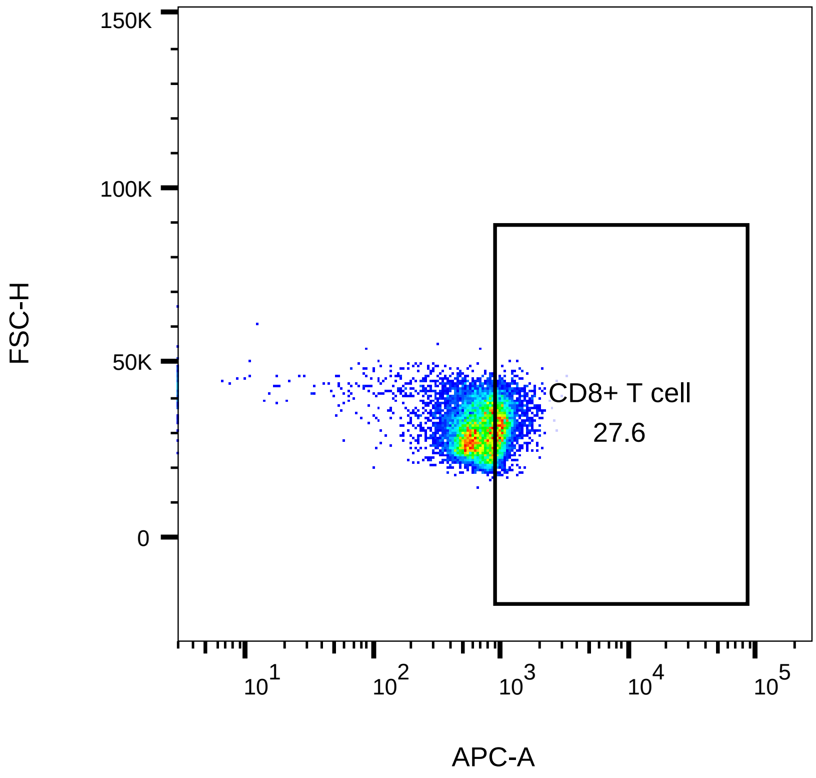

Specimen_001_wboty_028_029.fcs
Single Cells
12590

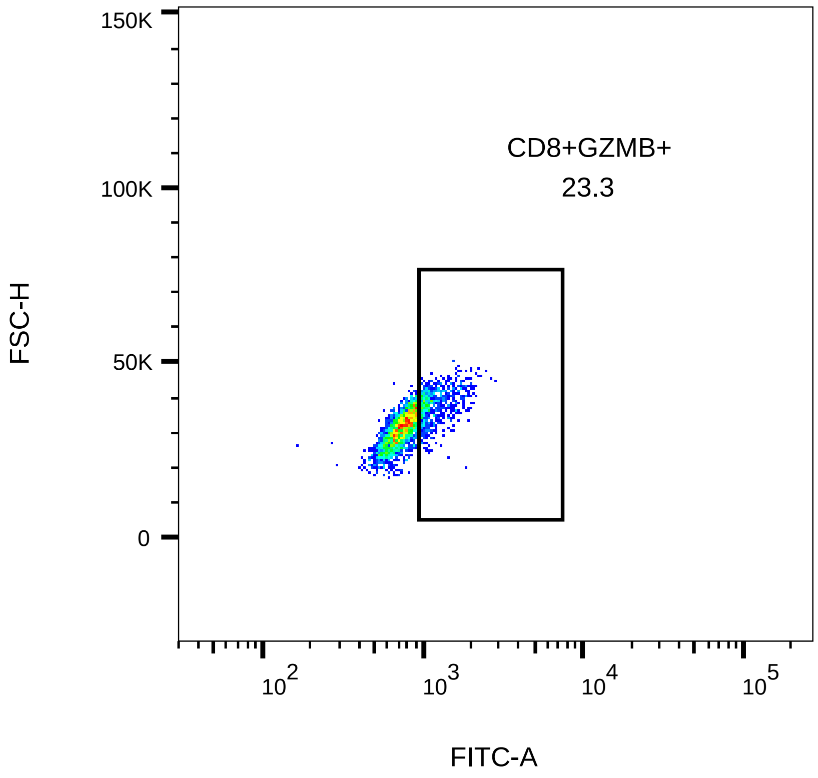

Specimen_001_wboty_028_029.fcs
CD8+ T cell
3471

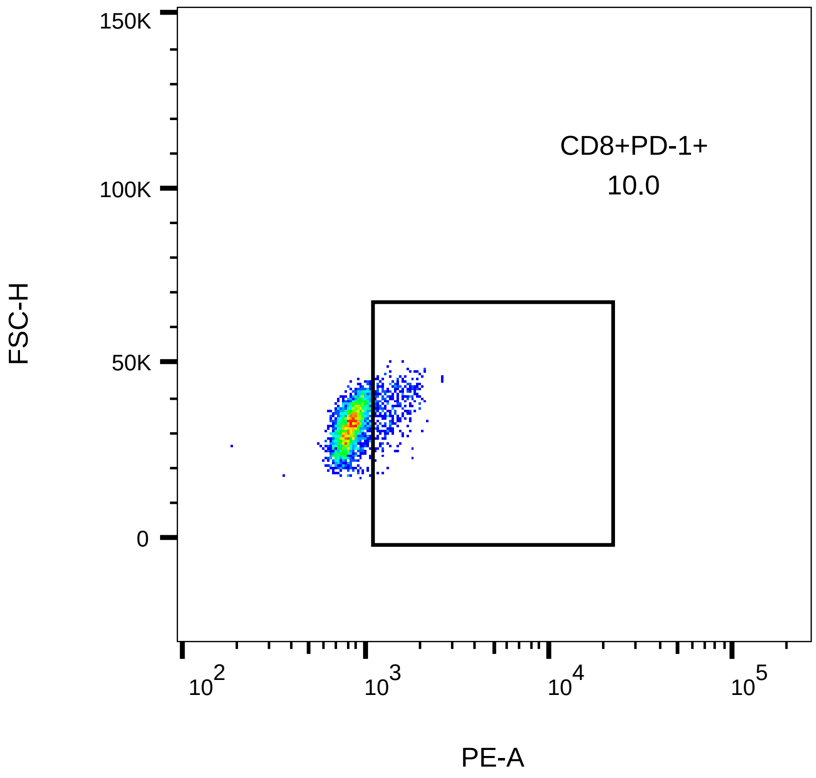

Specimen_001_wboty_028_029.fcs
CD8+ T cell
3471
