# Supplementary material for: MCUB Inhibits PRKN‐Dependent Mitophagic Degradation of PD‐L1 to Promote Immune Evasion in Bladder Cancer
Source: Adv Sci (Weinh). 2025 Nov 12;13(5):e14764. doi: 10.1002/advs.202514764 (PMC12849890; doi:10.1002/advs.202514764)
Supplement: Supplementary file 2 — Supporting Information [file ADVS-13-e14764-s002.zip › Figure11.docx]

**Figure11:**

**Figure11 A, left Figure11 A, right**


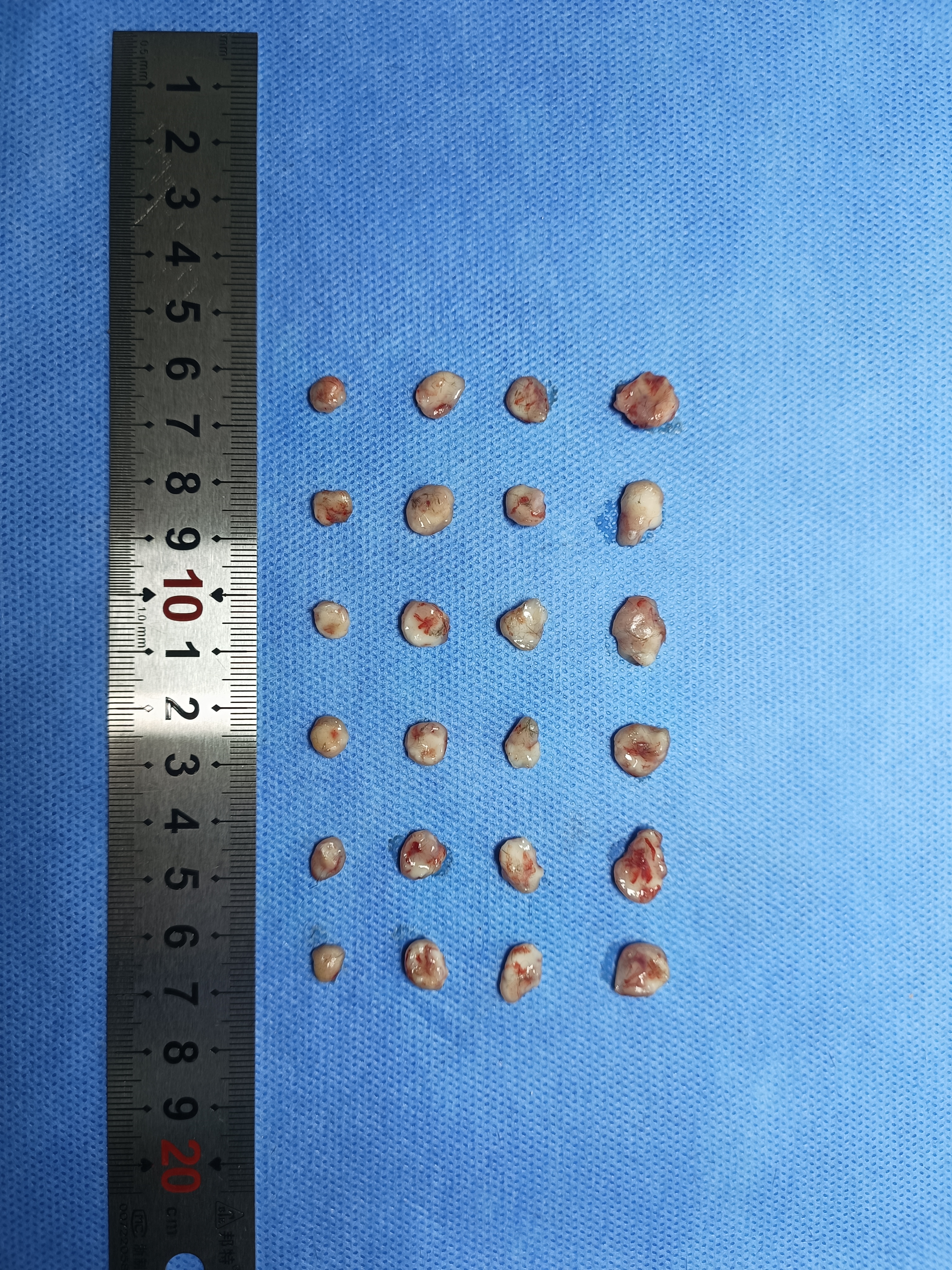

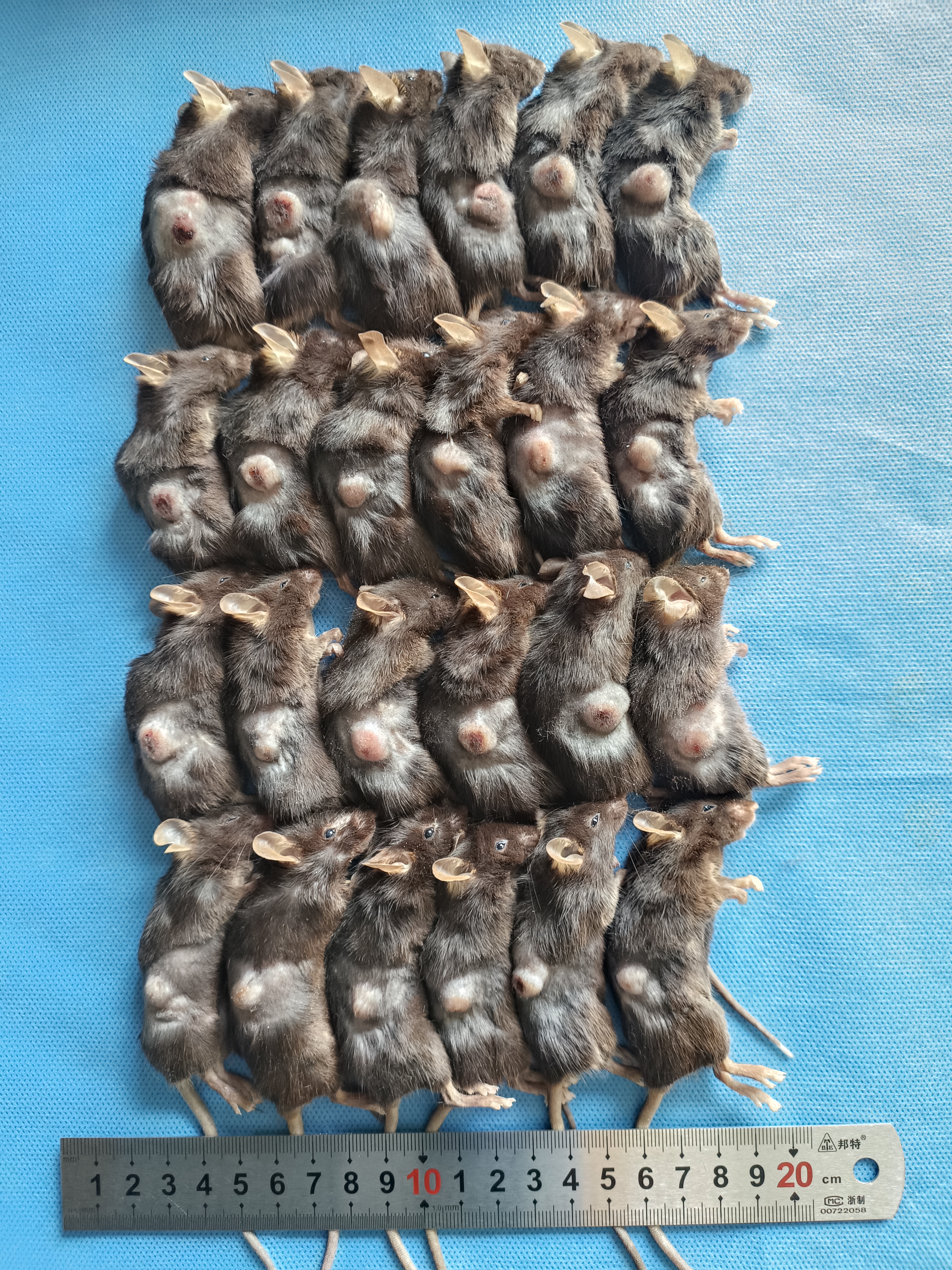


**Figure11 C**

**The details were presented in the attached file “*Figure11 C.pdf”.***

**Figure11 C, shNC: CD8+T cell**

**
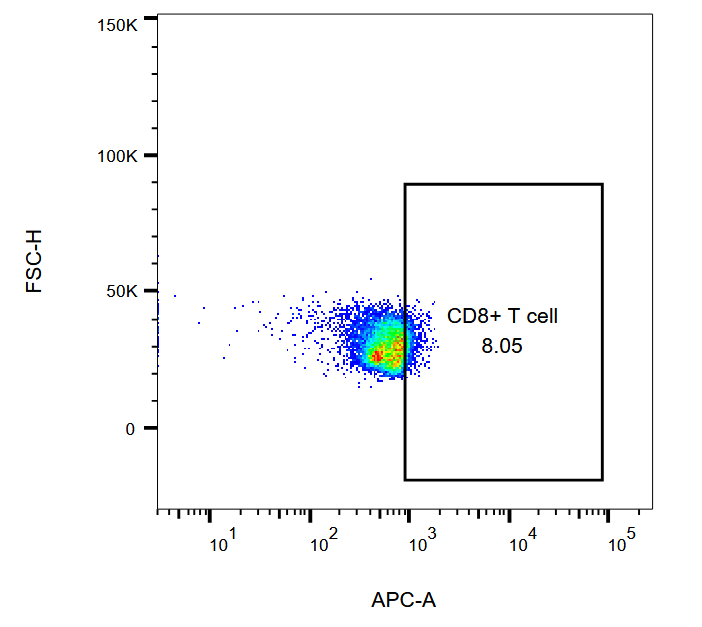
**

**Figure11 D, shNC: CD8+GZMB+**

**
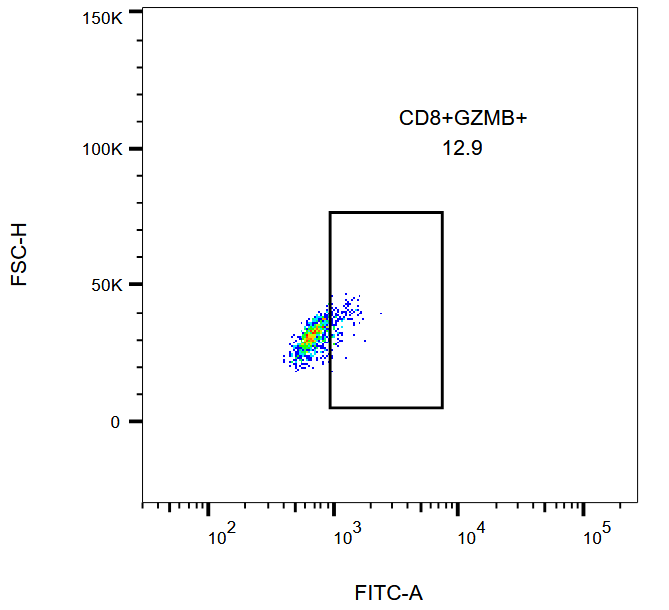
**

**Figure11 E, shNC: CD8+PD-1+**

**
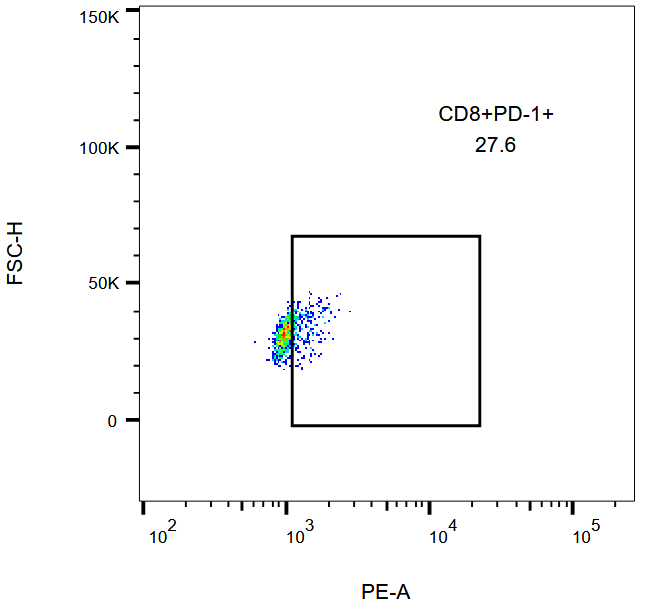
**

**Figure11 C, shMCUB: CD8+ T cell**

**
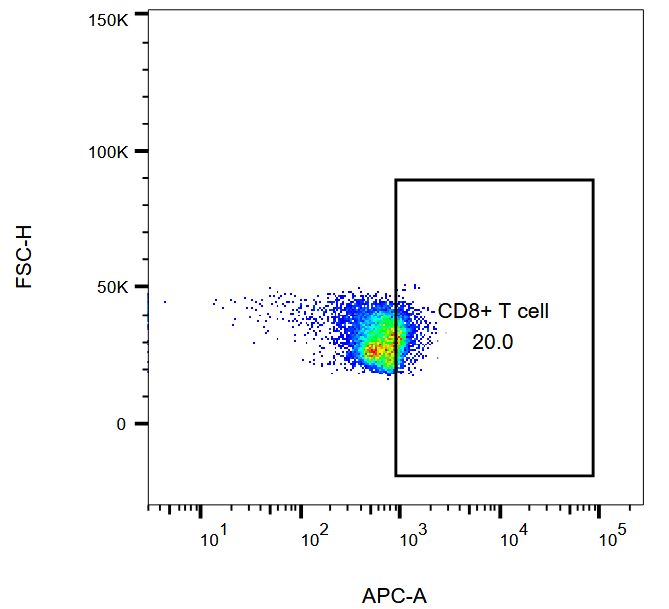
**

**Figure11 D, shMCUB: CD8+GZMB+**

**
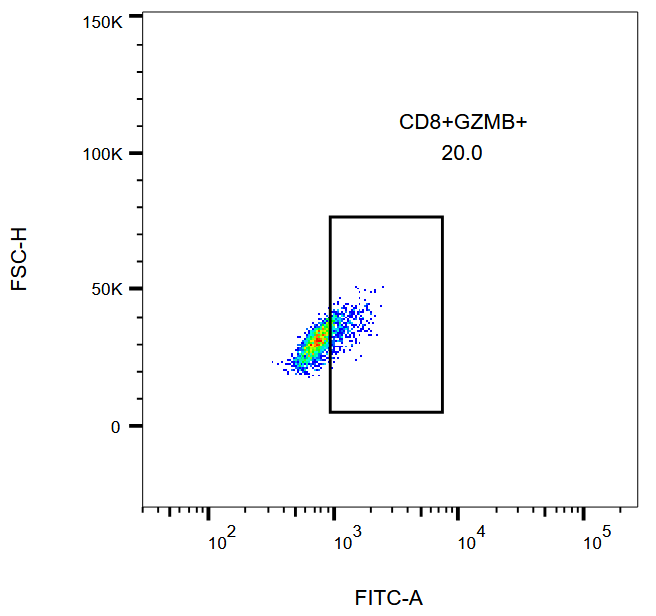
**

**Figure11 E, shMCUB: CD8+PD-1+**

**
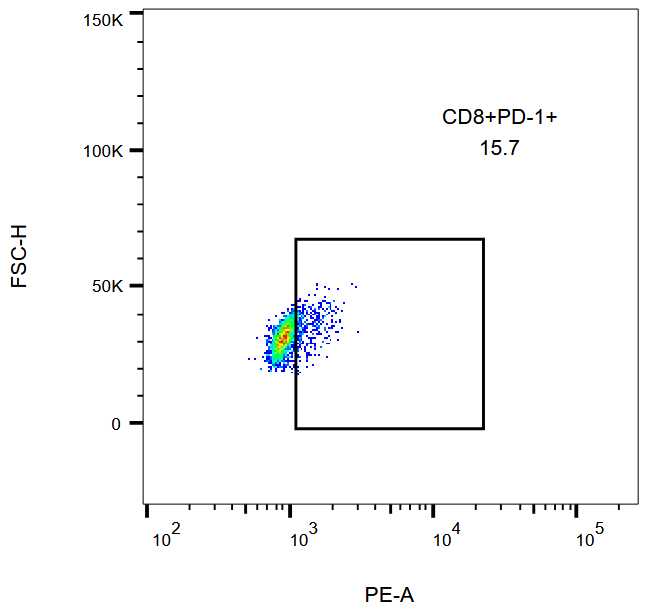
**

**Figure11 C, anti-PD-1: CD8+ T cell**

**
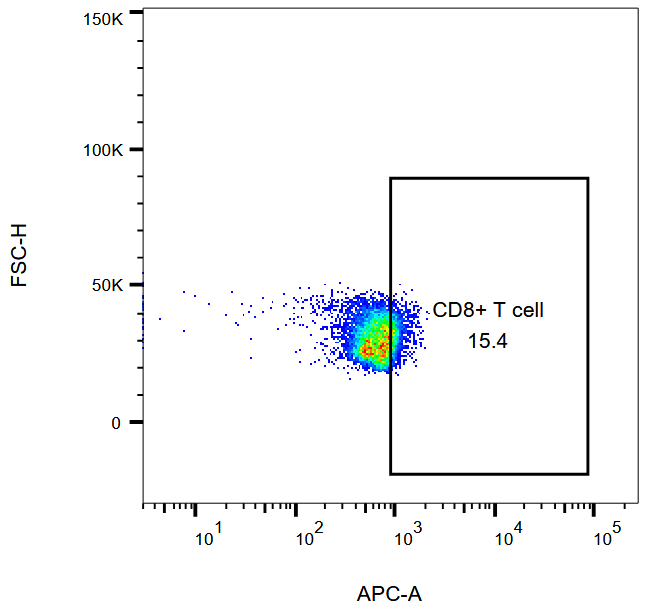
**

**Figure11 D, anti-PD-1: CD8+GZMB+**

**
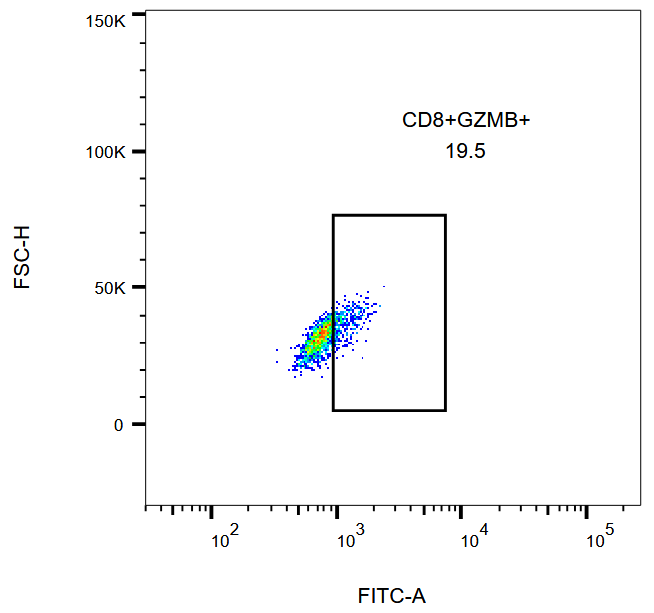
**

**Figure11 E, anti-PD-1: CD8+PD-1+**

**
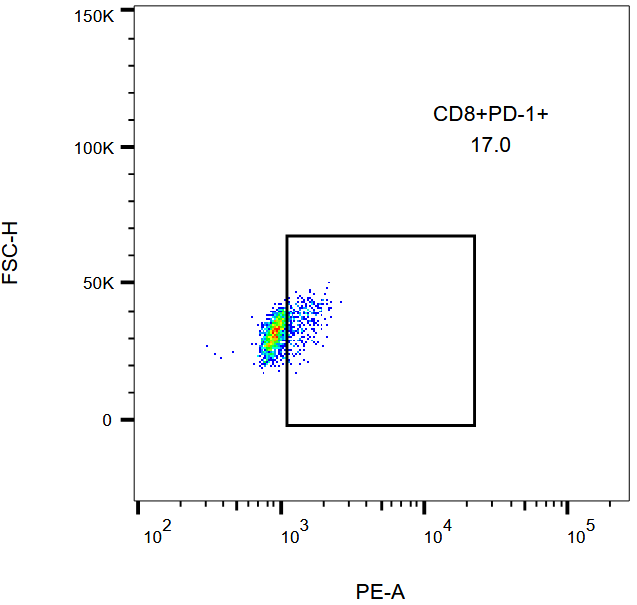
**

**Figure11 C, shMCUB+anti-PD-1: CD8+ T cell**

**
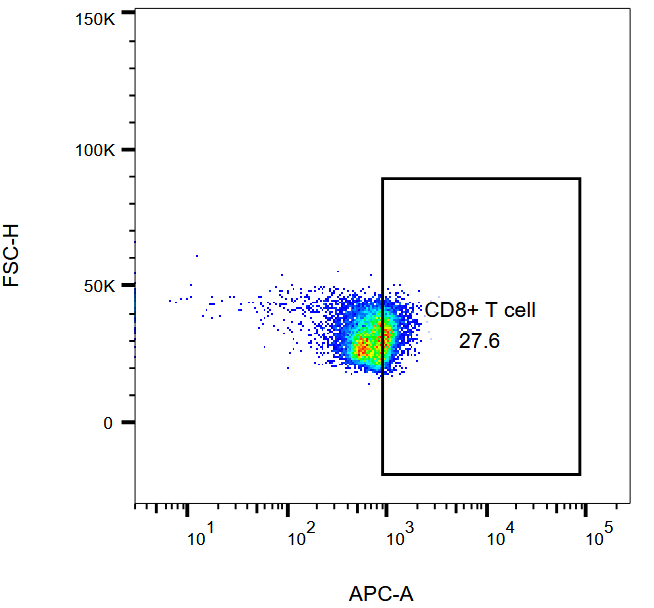
**

**Figure11 D, shMCUB+anti-PD-1: CD8+GZMB+**

**
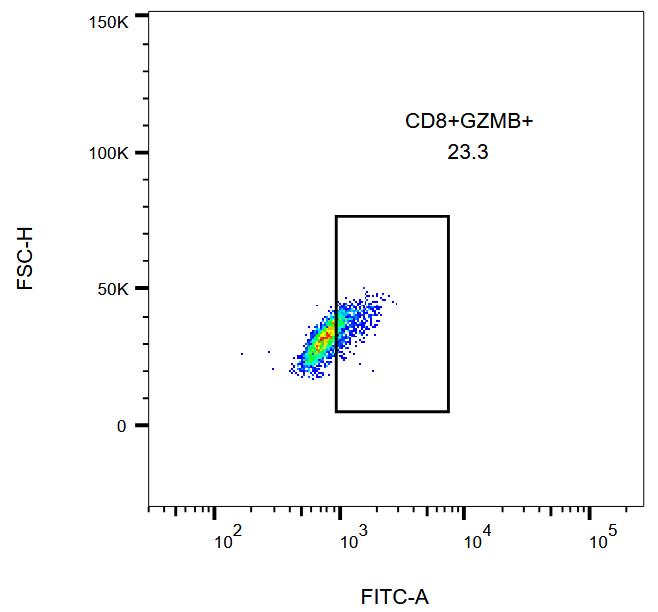
**

**Figure11 E, shMCUB+anti-PD-1: CD8+PD-1+**

**
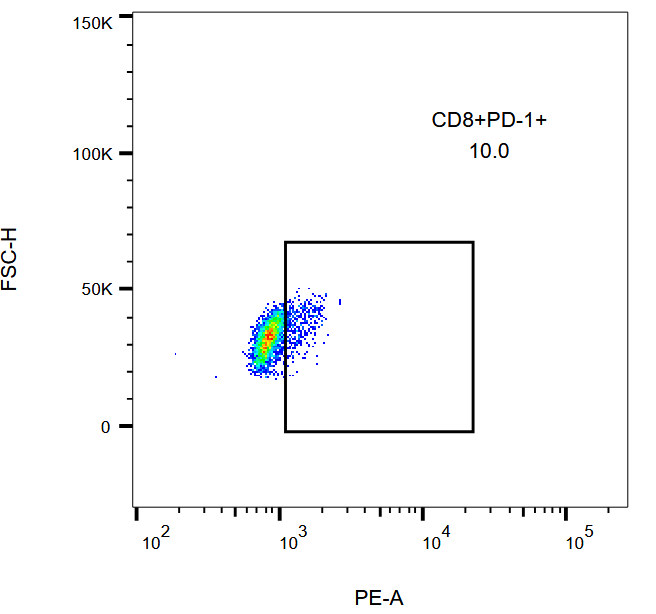
**
