# Supplementary figures and images for: MCUB Inhibits PRKN‐Dependent Mitophagic Degradation of PD‐L1 to Promote Immune Evasion in Bladder Cancer
Source: Adv Sci (Weinh). 2025 Nov 12;13(5):e14764. doi: 10.1002/advs.202514764 (PMC12849890; doi:10.1002/advs.202514764)

**Figure S1:**


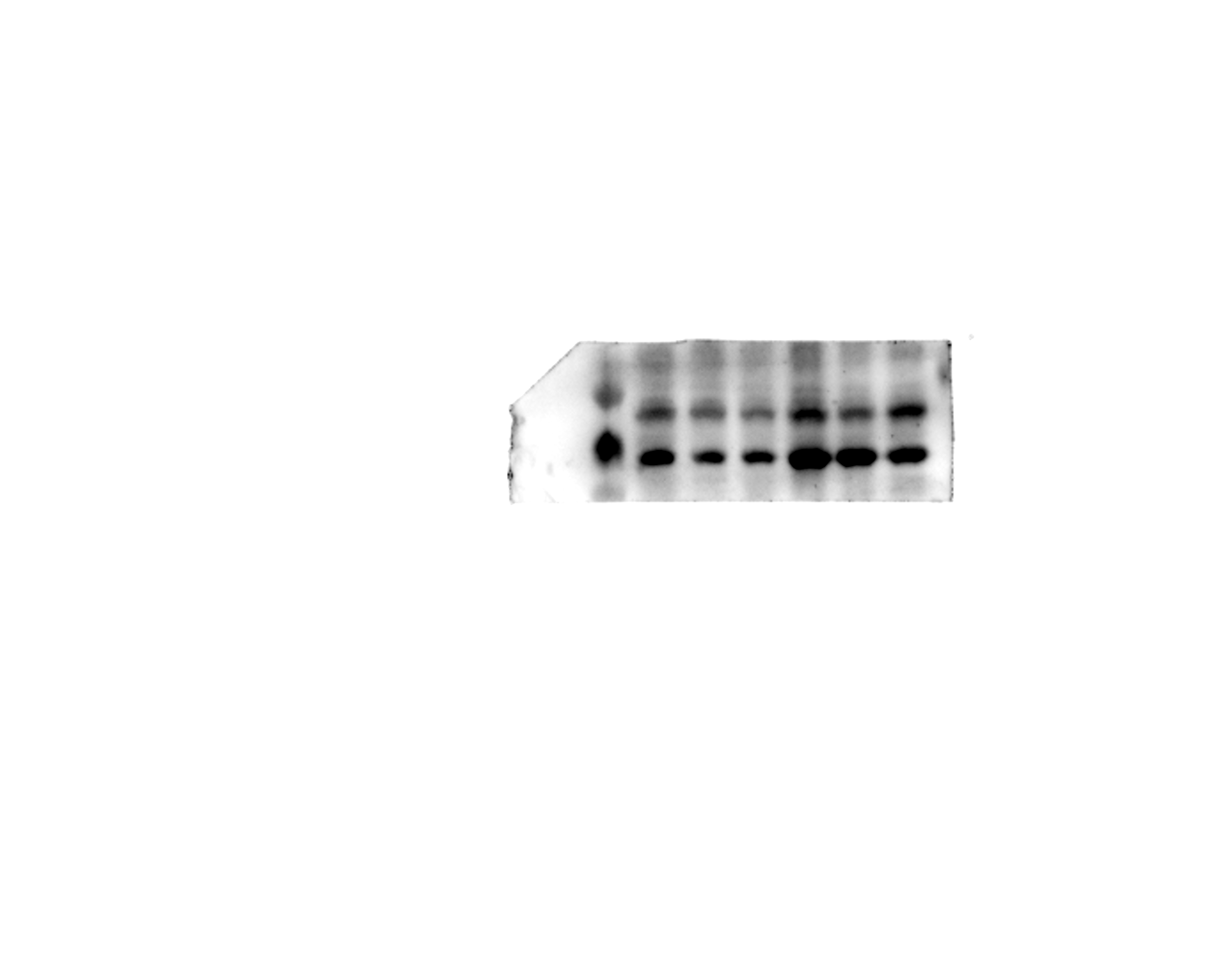
 **Figure S1F: MCUB**


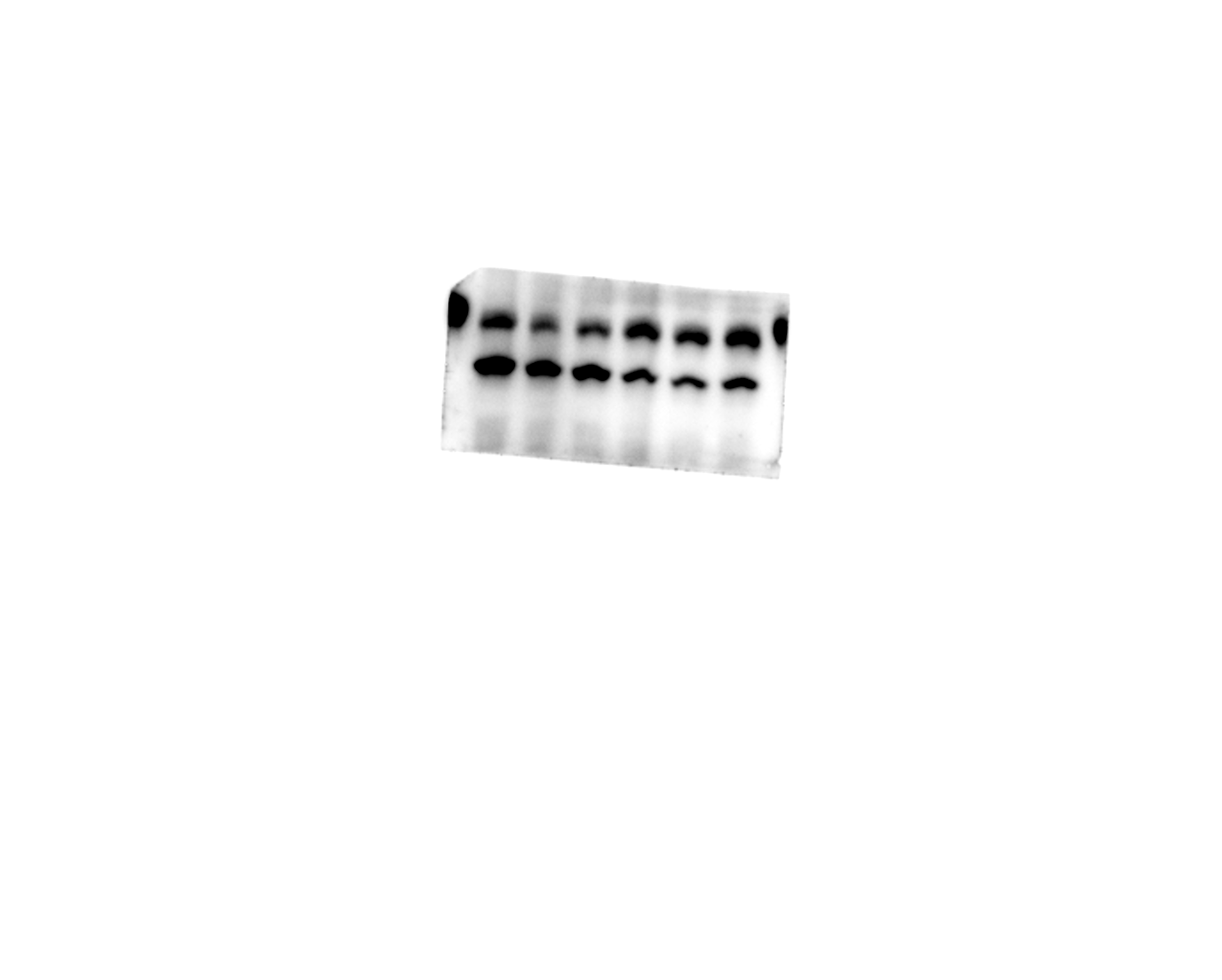
**Figure S1F: PD-L1**


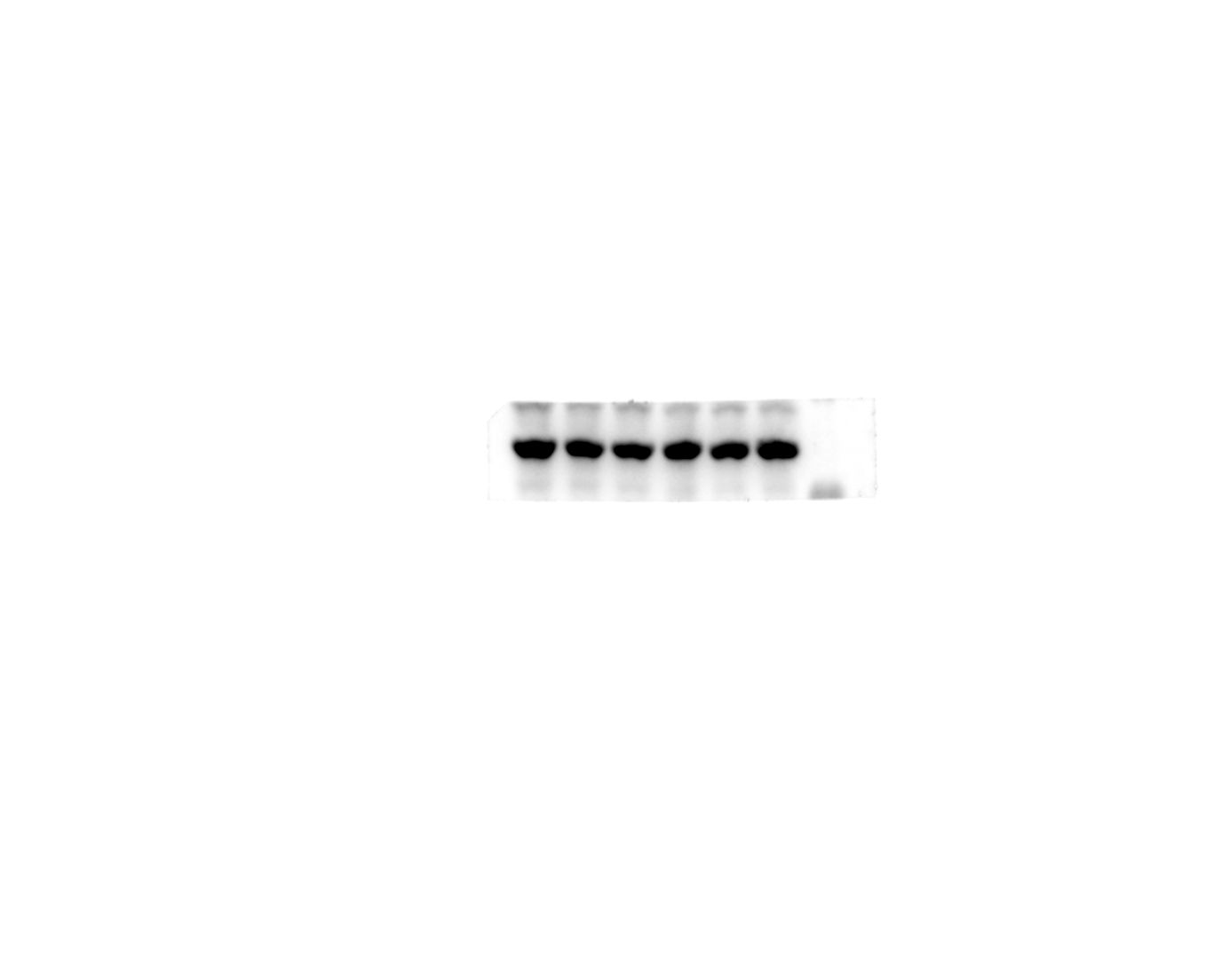
 **Figure S1F: β-Actin**


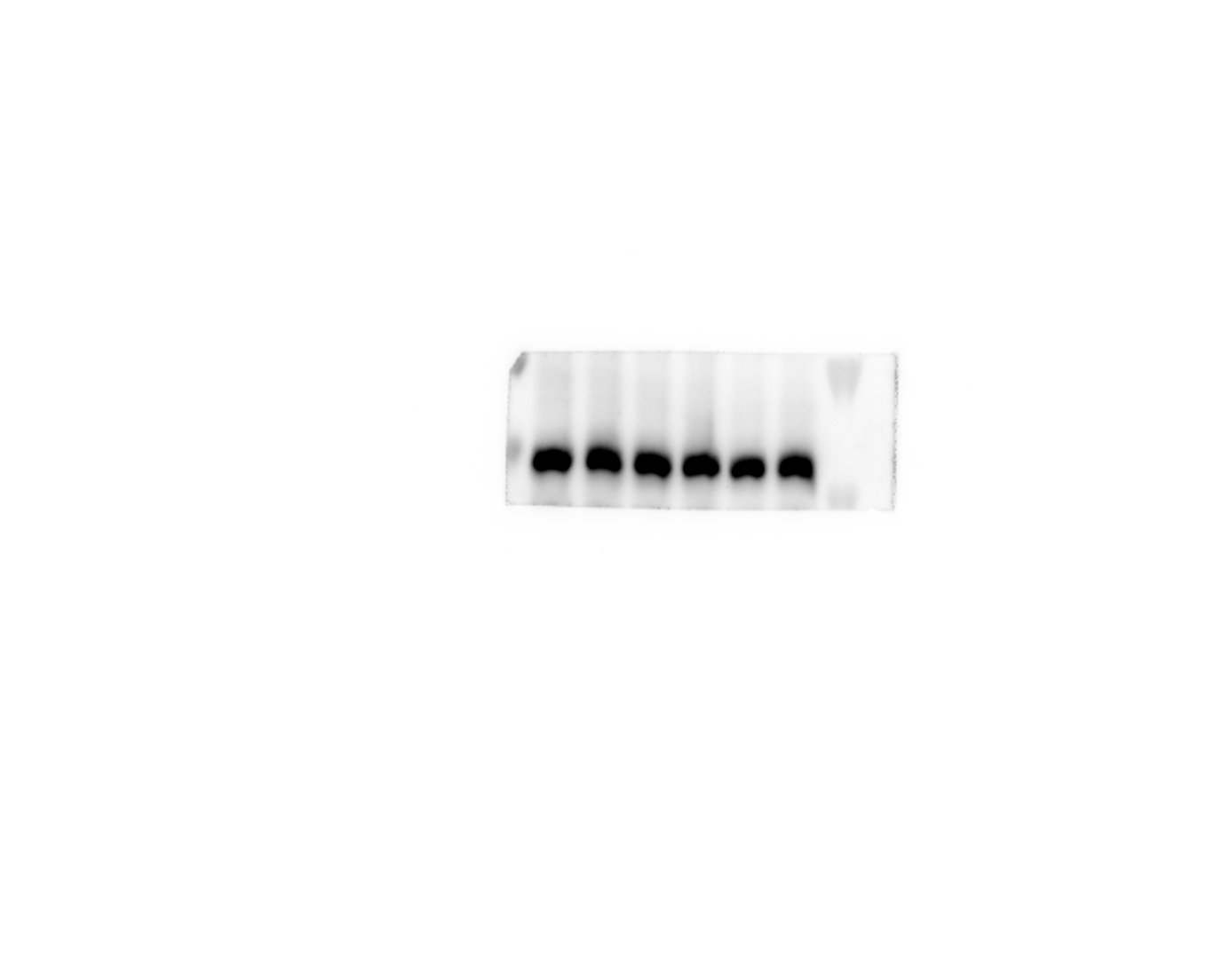
 **Figure S1F: α-Tubulin**

Supplement: Supplementary file 2 — Supporting Information [file ADVS-13-e14764-s002.zip › DataFile.docx]
